# Chiral spirocyclic phosphoric acid-catalyzed enantioselective synthesis of heterotriarylmethanes bearing an amino acid moiety†

**DOI:** 10.1039/d3ra03480a

**Published:** 2023-06-22

**Authors:** Jin Jiaping, Alemayehu Gashaw Woldegiorgis, Xufeng Lin

**Affiliations:** a Center of Chemistry for Frontier Technologies, Department of Chemistry, Zhejiang University Hangzhou 310058 China lxfok@zju.edu.cn

## Abstract

We present herein an enantioselective protocol for the chiral phosphoric acid-catalyzed addition of 3-arylisoxazol-5-amines to highly reactive 3-methide-3*H*-pyrroles to provide a diverse range of heterotriarylmethanes bearing an amino acid moiety in good yields (up to 97%) and high enantioselectivities (up to 93% ee) under mild conditions. The chiral spirocyclic phosphoric acid is crucial in converting the initial 1*H*-pyrrol-3-yl carbinols into reactive 3-methide-3*H*-pyrroles and obtaining the good enantiocontrol, thereby facilitating the desired enantioselective transformation.

## Abstract 2

Chiral spirocyclic phosphoric acid-catalyzed enantioselective synthesis of heterotriarylmethanes bearing an amino acid moiety has been developed.

## Graphical abstract

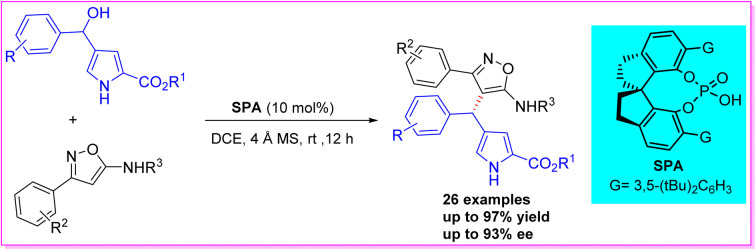

Triarylmethanes are incredibly interesting, with incredible potential for various applications in fields including medicinal chemistry, materials science and organic synthesis.^1^ Among these applications, several triarylmethanes have emerged with promising importance in organic functional materials,^2^ dyes^3^ and biologically active compounds with different pharmacological activities such as anti-tuberculosis,^4^ antibacterial,^5^ anti-viral, anti-cancer^6^ and cytotoxic activities against MCF-7 cells (Fig. 1).^7^ Given the numerous benefits of triarylmethanes, the synthetic chemistry community has focused on creating novel synthetic strategies, including the development of methods for their asymmetric synthesis.

**Fig. 1:**
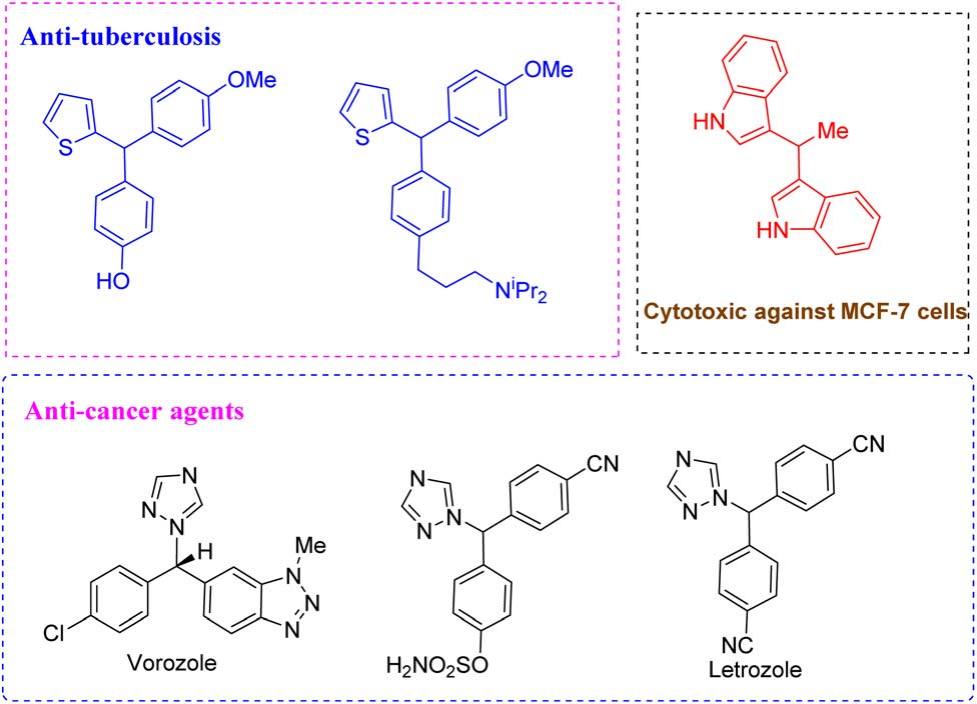
Heterotriarylmethanes with different pharmacologi-cal activities.

Over the past few decades, various methods have been reported for the synthesis of triarylmethanes, including transition metal catalyzed cross-coupling,^1*a*,8^ transition-metal-free sequential cross-coupling reaction,^9^ visible light-induced thiourea photoacids catalyze C–C bond-forming reactions,^10^ and Brønsted acidic ionic liquid [bsmim][NTf_2_] catalyzed three-component Friedel–Crafts reaction.^1*b*^ In addition, there have been developments in the organocatalytic synthesis of triaryl methanes bearing different groups including triphenyl, diphenylheteroaryl, and diheteroaryl phenyl groups (with similar heteroaryls). These methods include organocatalytic transfer hydrogenation of *para*-quinone methides,^11^ oxidative cross-coupling of racemic 2,2-diarylacetonitriles with electron-rich (hetero)arenes,^12^ and regio- and enantioselective Friedel–Crafts alkylation of aniline derivatives with *para*-quinone methides.^13^ Other successful methods include Friedel–Crafts reaction of indoles and phenols with *in situ*-generated *ortho*-quinone methides,^14^ Brønsted acid catalyzed reaction of *in situ* generated aza-o-QM with 2-substituted indoles,^15^ 2-indolylmethanols with 3-alkylindoles^16^ and indoles,^17^ and from racemic tertiary alcohols with indoles were also reported to synthesize enantioenriched triarylmethanes.^18^ However, the organocatalytic asymmetric synthesis of triarylmethanes bearing three different aryl/heteroaryl scaffolds has long been a challenge due to the lack of sufficient steric difference between the aryl rings, and remains rare.^1*a*,19^

In 2014, Zhang and co-workers achieved the chiral imidodiphosphoric acid-catalyzed enantioselective synthesis of triarylmethanes bearing two different heteroaryl groups (indole and pyrrole) and a phenyl group in high yields and enantioselectivities (Scheme 1a).^6*c*^ Liao and co-workers also developed an efficient enantioselective construction of structurally diverse C_2_-substituted triarylmethane derivatives by catalytic enantioselective 1,4-addition reaction of 3-substituted indoles, pyrroles, and furans with azadienes by using a chiral phosphoric acid (Scheme 1b).^4*c*^ However, these strategies have drawn more attention to the synthesis of chiral triarylmethanes bearing heteroarenes such as indoles, pyrroles and furans or a phenyl ring, and the asymmetric versions of chiral triarylmethanes containing heteroaromatic structures such as isoxazole and pyrrole rings together have not been disclosed yet.

**Scheme 1:**
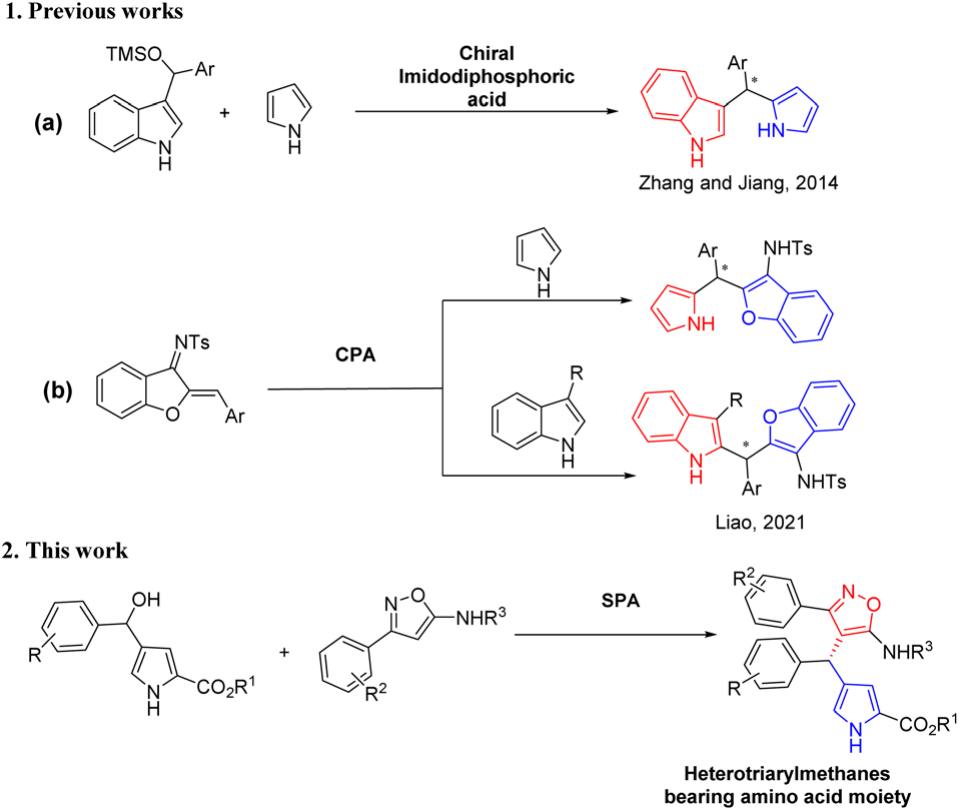
Asymmetric synthesis of heterotriarylmethanes bearing two different heteroaryl skeletons.

Medicinal chemists place great importance on the availability of a diverse range of functionalized heterocyclic scaffolds and their use in the asymmetric synthesis of various compounds.^20^ In this regard, five-membered heterocycles containing N and O atoms, such as isoxazoles, play a vital role in many naturally occurring compounds with significant medicinal and pharmaceutical applications.^21^ Isoxazoles exhibit diverse pharmacological properties, including anti-inflammatory,^22^ anti-cancer,^23^ anti-viral,^24^ anti-bacterial^25^ and antidepressant activities,^26^ and they are used as pesticides and insecticides in agrochemicals.^21*b*^ As a result, the synthesis of enantioenriched compounds with isoxazole moieties has garnered attention from chemical researchers. Recently, asymmetric synthesis of compounds bearing isoxazole was synthesized *via* organocatalytic asymmetric 1,6-addition of pyrazol-5-ones to 3-methyl-4-nitro-5-alkenylisoxazoles^27^ and enantioselective addition of 5-amino-isoxazoles with β,γ-alkynyl-α-ketimino esters in high yields and enantioselectivities.^28^

Recently, 1*H*-pyrrol-3-yl carbinol has emerged as an active class of reactants in catalytic asymmetric transformations. Upon dehydration of 1*H*-pyrrol-3-yl carbinol in the presence of an acid catalyst, highly reactive 3-methide-3*H*-pyrroles are formed. However, due to the challenging preparation and synthetic handling, their application in organic synthesis has been limited and received little attention from chemists in the research field of asymmetric catalysis. So far, only Schneider's group has described the highly stereoselective (3 + 2)-cycloannulation of cyclic enamides to *in situ*-generated 3-methide-3*H*-pyrroles^29^ and [6 + 2]-cycloaddition of 3-methide-3*H*-pyrroles with 2-vinylindoles in the presence of chiral phosphoric acid.^30^ In continuation of our efforts to explore the chiral phosphoric acid catalyzed asymmetric synthesis of enantioenriched compounds,^31^ herein, we report the asymmetric construction of heterotriarylmethanes bearing amino acid moiety from the reaction of 5-aminoisoxazole and 3-methide-3*H*-pyrroles generated *in situ via* chiral spirocyclic phosphoric acid catalysis developed by our group.^32^

Initially, we examined the reaction between ethyl 4-(hydroxy(*m*-tolyl)methyl)-1*H*-pyrrole-2-carboxylate (1a) and 3-phenylisoxazol-5-amine (2a) in dichloroethane (DCE), using 10 mol% of chiral spirocyclic phosphoric acids for 12 hours at room temperature (Table 1). First, chiral phosphoric acid (*S*)-4.1a was used in the reaction and afforded the desired product, heterotriarylmethane 3a in moderate yield (60%) and enantioselectivity (42% ee) (Table 1, entry 1). With this promising outcome, other chiral spirocyclic phosphoric acids (*S*)-4.1b – (*S*)-4.1i were also tested, and it was discovered that the catalyst (*S*)-4.1i performed well in the reaction, yielding the corresponding product 3a with high yield (86%) and enantioselectivity (72% ee) (Table 2, entry 9).

**Table 1:** Optimization of the reaction conditions^a^

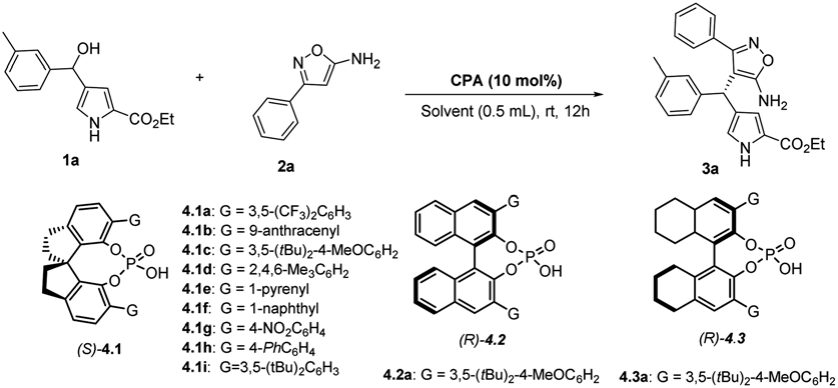				
Entry	Catalyst	Solvent	Yield^b^ (%)	ee^c^ (%)
1	(*S*)-4.1a	DCE	60	42
2	(*S*)-4.1b	DCE	73	38
3	(*S*)-4.1c	DCE	80	66
4	(*S*)-4.1d	DCE	64	2
5	(*S*)-4.1e	DCE	81	30
6	(*S*)-4.1f	DCE	47	18
7	(*S*)-4.1g	DCE	89	63
8	(*S*)-4.1h	DCE	71	58
9	(*S*)-4.1i	DCE	86	72
10	(*R*)-4.2a	DCE	72	32
11	(*R*)-4.3a	DCE	75	34
12	(*S*)-4.1i	DCM	79	64
13	(*S*)-4.1i	CHCl_3_	83	64
14	(*S*)-4.1i	Et_2_O	59	52
15	(*S*)-4.1i	EA	74	59
16	(*S*)-4.1i	1,4-Dioxane	80	32
17^d^	(S)-4.1i	DCE	86	86
**18** e	**(*S*)-**4.1i	**DCE**	**88**	**87**
19^f^	(*S*)-4.1i	DCE	56	79

a Reaction conditions: 1a (0.05 mmol), 2a (0.06 mmol) and catalyst (10 mol%) in DCE (0.5 mL) at room temperature for 12 h.

b Isolated yields.

c Determined by chiral HPLC analysis.

d 3 Å MS.

e 4 Å MS.

f At 0 °C.

**Table 2:** The scope of the reaction with respect to both substrates^a^

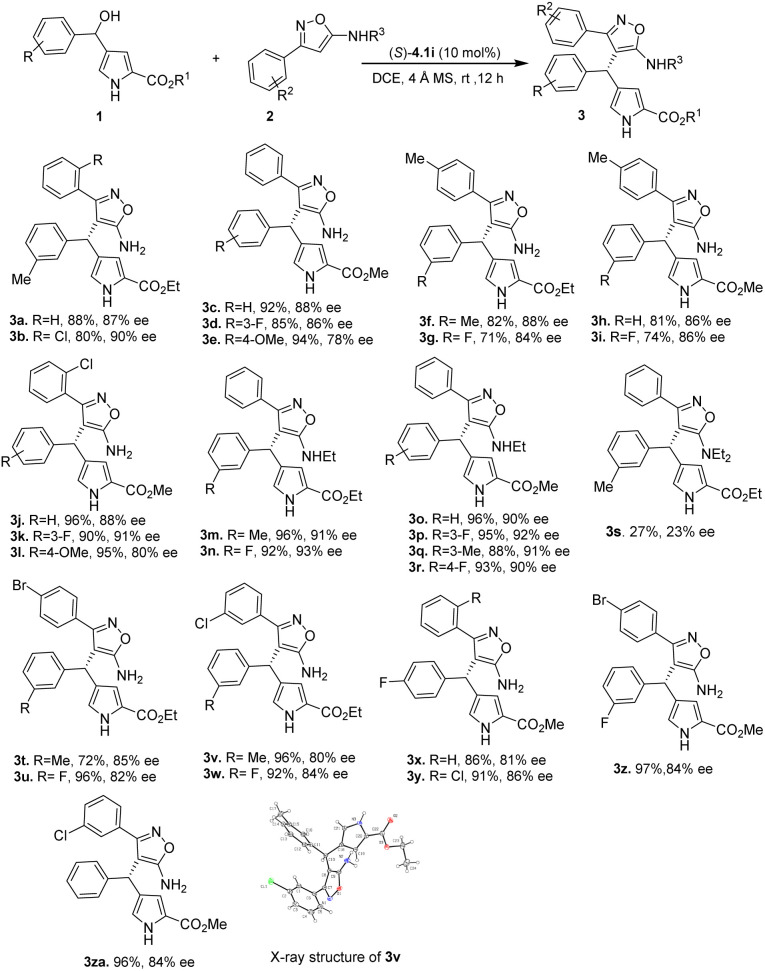

a Reaction conditions: 1 (0.05 mmol), 2 (0.06 mmol) and (*S*)-4.1i (10 mol%) using 4 Å MS in DCE (0.5 mL) at room temperature for 12 h. Isolated yields. The ee values of 3 were determined by HPLC analysis with a chiral stationary phase.

In an attempt to enhance the yield and enantioselectivity, alternative chiral phosphoric acid catalysts,^33^ including BINOL and H8-BINOL with different groups, were evaluated. Nonetheless, these catalysts demonstrated lower yields and enantioselectivities (Table 1, entries 10–11 and ESI†). Furthermore, different solvents such as dichloromethane, chloroform, diethyl ether, ethyl acetate, and 1,4-dioxane were examined while using (*S*)-4.1i as a catalyst. However, no significant improvement in either yield or enantioselectivity was observed (Table 1, entries 12–16). Gratifyingly, after investigating several additives, it was discovered that 4 Å MS was the most effective in terms of both yield and enantioselectivity (Table 1, entry 18). Moreover, the reaction was assessed at a lower temperature of 0 °C but resulted in a lower yield and enantioselectivity of the corresponding product 3a (Table 1, entry 19).

With the optimized conditions in hand, we explored the scope of reactions involving several derivatives of 4-(hydroxy(phenyl)methyl)-1*H*-pyrrole-2-carboxylate (1) with 3-phenylisoxazol-5-amine derivatives (2) (Table 2). The utilization of 3-phenylisoxazol-5-amine 2a and 2b containing electron neutral and electron-withdrawing group, like a chloro group in the *ortho* position of the phenyl ring, resulted in heterotriarylmethane products 3a and 3b in high yields with good enantioselectivities (3a: 88%, 87% ee; 3b: 80%, 90% ee). The methyl 4-(hydroxy(phenyl)methyl)-1*H*-pyrrole-2-carboxylates 1c–1e, which have electron neutral, electron withdrawing (F) and donating (OMe) groups at different positions, were also tolerated and provided the corresponding products 3c–3e in high yields (85–94%) and enantioselectivities (78–88% ee).

Furthermore, when 4-(hydroxy(phenyl)methyl)-1*H*-pyrrole-2-carboxylate derivatives bearing different groups at the *meta* position of phenyl ring 1a–1d, were treated with 3-(*p*-tolyl)isoxazol-5-amine 2b, the desired heterotriarylmethanes 3f–3i were obtained in good yields (71–82%) and high enantioselectivities (84–88% ee). Similarly, when 3-(2-chlorophenyl)isoxazol-5-amine was reacted with methyl 4-(hydroxy(phenyl)methyl)-1*H*-pyrrole-2-carboxylates bearing electron-withdrawing or electron-donating groups at the *para* and *meta* positions of the phenyl group, the corresponding products 3j–3l were resulted in excellent yields (90–96%) and good enantioselectivities (80–91% ee). It is interesting to note that substituting one of the hydrogen atoms in the amino group of 3-phenylisoxazol-5-amine with an ethyl group resulted in improved enantioselectivities of the products. For instance, when *N*-ethyl-3-phenylisoxazol-5-amine 2d was treated with 1a and 1b, the desired heterotriarylmethanes 3m–3n were obtained in excellent yields (92–96%) and enantioselectivities (91–93% ee). Even substrates 1c–1d and 1f–1g with electron donating and withdrawing groups were compatible with *N*-ethyl-3-phenylisoxazol-5-amine 2d, yielding products 3o–3r with high yields (88–96%) and enantioselectivities (90–92% ee). However, replacing both hydrogen atoms of the amino group with ethyl groups resulted in lower yields and enantioselectivities (3s: 27%, 23% ee), indicating that the NH of the amino group is crucial in the reaction as it forms hydrogen bonding with chiral phosphoric acid catalyst to increase reactivity and enantioselectivity.

High yields (72–96%) and enantioselectivities (80–85% ee) were obtained for the products 3t–3w when 3-(4-bromophenyl)-isoxazol-5-amine 2e and 3-(3-chlorophenyl)isoxazol-5-amine 2f were reacted with ethyl 4-(hydroxy(phenyl)methyl)-1*H*-pyrrole-2-carboxylates bearing electron-donating (–Me) and withdrawing groups (–F). Additionally, heterotriarylmethanes 3x–3za were produced in good yields (86–97%) and enantioselectivities (81–86% ee) when 3-phenylisoxazol-5-amine substrates bearing electron-withdrawing groups on the *ortho*, *meta*, and *para* positions of the phenyl ring were smoothly reacted with methyl 4-(hydroxy(phenyl)methyl)-1*H*-pyrrole-2-carboxylate derivatives. X-ray crystallographic analysis was used to determine the absolute stereochemistry of 3v (CCDC 2258261) to be *S*, while the configurations of other products were tentatively assigned similarly.

Next, we conducted a scale-up synthesis of 3b and 3m and synthetic conversion of compound 3k (Scheme 2). In order to explore the practicability of the reaction, the product 3b and 3m were synthesized efficiently on the 1 mmol scale with good yields and nearly the same enantioselectivities, making further derivatization feasible (Scheme 2a). The hydrolysis of 3k using NaOH in THF/MeOH/H_2_O (2/2/0.5) resulted in the removal of the methyl group to give heterotriarylmethane bearing amino acid moiety 4a in high yield (96%) and enantioselectivity (92% ee) (Scheme 2b).

**Scheme 2:**
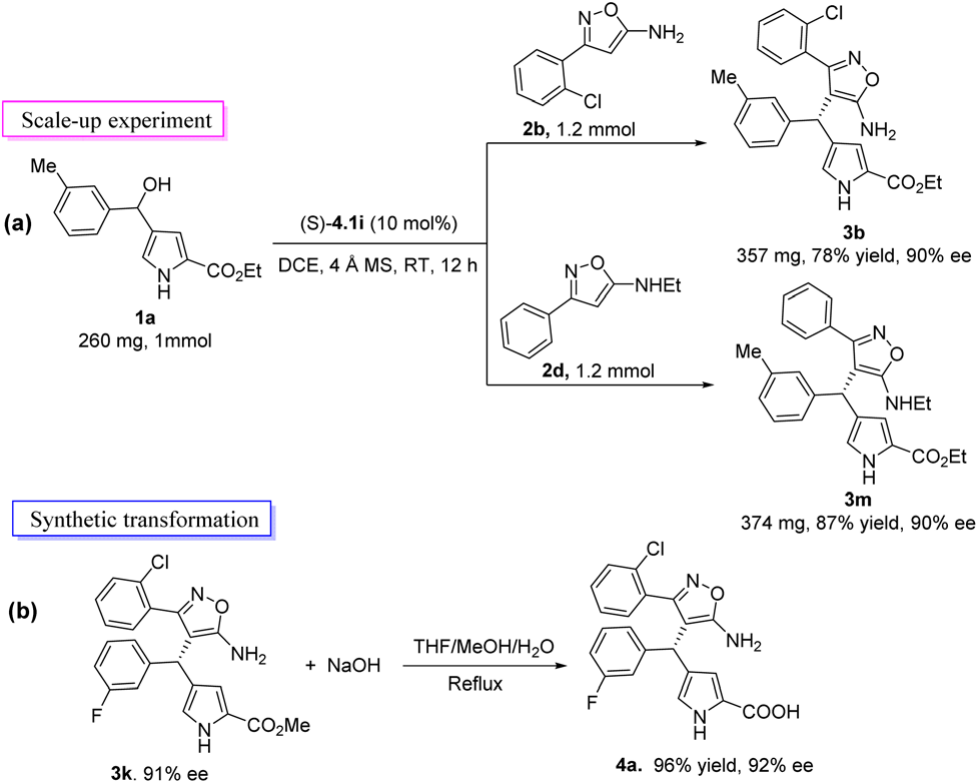
Scale-up experiment and synthetic transformations.

According to the literature,^29,30^ and our experimental results, a possible reaction pathway was proposed and presented in Scheme 3. Initially, a highly reactive 3-methide-3*H*-pyrrole 1I was generated *in situ* from 1*H*-pyrrol-3-yl carbinol 1 through dehydration in the presence of chiral phosphoric acid, which activated the electrophilicity of 1*H*-pyrrol-3-yl carbinol *via* hydrogen bonding. Then, the substrate 3-arylisoxazol-5-amine 2 underwent Friedel–Crafts reaction with 3-methide-3*H*-pyrroles 1I on the Si face by forming dual hydrogen bonds with the catalyst to produce the corresponding products 3 upon the regeneration of catalyst (*S*)-4.1i.

**Scheme 3:**
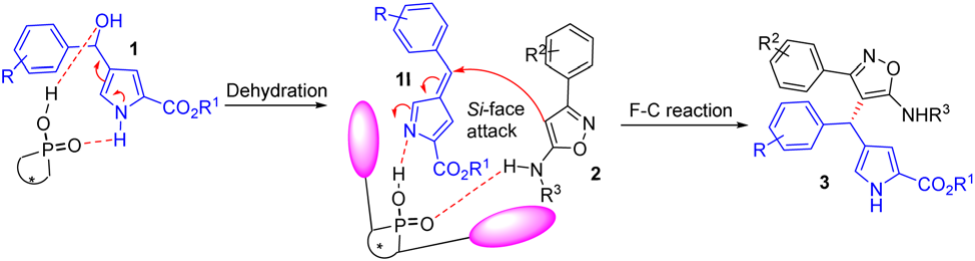
The proposed reaction mechanism.

## Conclusions

In summary, we successfully developed a chiral phosphoric acid catalyzed Friedel–Crafts reaction of nucleophilic 5-aminoisoxazoles with *in situ*-generated 3-methide-3*H*-pyrrole. This reaction produced a diverse range of chiral heterotriarylmethane bearing amino acid moiety in high yields and good enantioselectivities (up to 97% yield, 93% ee). This reaction method is not only an efficient way to construct biologically important heterotriarylmethanes bearing amino acid moiety in an enantioselective form, but also promotes the development of 1*H*-pyrrol-3-yl carbinol-involved catalytic enantioselective construction of structurally diverse enantioenriched triarylmethanes and other asymmetric transformations. Enantioselectivity was preserved during the successful conduct of certain synthetic transformation, without any erosion.

## Experimental

### General procedure to synthesize compound 3

1*H*-pyrrol-3-yl carbinol 1 (0.1 mmol), 3-arylisoxazol-5-amine 2 (0.12 mmol), chiral phosphoric acid (*S*)-4.1i (3.4 mg, 10 mol%, 0.01 mmol) and 4 Å MS (50 mg) were added to a dried tube. Then, dichloroethane (0.5 mL) was added to the reaction mixture, which was stirred at 25 °C for 12 h. After the completion of the reaction which was indicated by TLC, the reaction mixture was directly purified by column chromatography using PE : EA (5 : 1) as eluent to afford the pure product 3.

#### (*S*)-Ethyl 4-((5-amino-3-phenylisoxazol-4-yl)(*m*-tolyl)methyl)-1*H*-pyrrole-2-carboxylate (3a)

White solid (35.2 mg, 88%); MP = 236–238 °C; the enantiomeric excess was determined to be 87% by HPLC analysis on Daicel Chiralcel AD-H column (*n*-hexane/i-PrOH = 70/30, flow rate 1.0 mL min^−1^, *T* = 25 °C), UV 254 nm; *t*_major_ = 9.706 min, *t*_minor_ = 6.877 min; [*α*]^20^_D_ = −27.6° (*c* = 0.19, CH_2_Cl_2_); ^1^H NMR (400 MHz, DMSO) *δ* 11.77 (s, 1H), 7.43–7.35 (m, 3H), 7.30 (dd, *J* = 8.0, 1.6 Hz, 2H), 7.17 (t, *J* = 7.8 Hz, 1H), 7.01 (d, *J* = 7.6 Hz, 1H), 6.92 (d, *J* = 7.4 Hz, 2H), 6.58 (d, *J* = 2.0 Hz, 1H), 6.46 (t, *J* = 1.9 Hz, 1H), 5.91 (s, 2H), 5.09 (s, 1H), 4.19 (q, *J* = 7.1 Hz, 2H), 2.23 (s, 3H), 1.24 (t, *J* = 7.1 Hz, 3H) ppm. ^13^C NMR (101 MHz, DMSO) *δ* 167.8, 163.3, 160.8, 143.4, 137.8, 130.7, 129.5, 129.0, 128.9, 128.8, 128.7, 127.4, 126.1, 125.6, 123.1, 122.5, 115.2, 92.5, 60.0, 37.2, 21.6, 14.9 ppm. HRMS (ESI-TOF) *m*/*z*: [M + Na]^+^ calcd for C_24_H_23_N_3_O_3_Na: 424.1739; found: 424.1631. IR (KBr, cm^−1^): 3451, 3315, 2976, 2894, 1694, 1635, 1479, 1439, 1196, 1103, 764.

#### (*S*)-Ethyl 4-((5-amino-3-(2-chlorophenyl)isoxazol-4-yl)(*m*-tolyl)methyl)-1*H*-pyrrole-2-carboxylate (3b)

Yellow solid (34.9 mg, 80%); MP = 107–109 °C; the enantiomeric excess was determined to be 90% by HPLC analysis on Daicel Chiralcel AD-H column (*n*-hexane/i-PrOH = 85/15, flow rate 1.0 mL min^−1^, *T* = 25 °C), UV 254 nm; *t*_major_ = 20.800 min, *t*_minor_ = 18.521 min; [*α*]^20^_D_ = −41.5° (*c* = 0.375, CH_2_Cl_2_); ^1^H NMR (400 MHz, DMSO) *δ* 11.69 (s, 1H), 7.47 (dd, *J* = 8.1, 1.0 Hz, 1H), 7.40 (td, *J* = 7.7, 1.7 Hz, 1H), 7.27 (td, *J* = 7.5, 1.2 Hz, 1H), 7.12–7.02 (m, 2H), 6.95 (d, *J* = 7.5 Hz, 1H), 6.88–6.79 (t, 2H), 6.55 (d, *J* = 1.9 Hz, 1H), 6.39 (t, *J* = 1.9 Hz, 1H), 6.09 (s, 2H), 4.79 (s, 1H), 4.19 (q, *J* = 7.1 Hz, 2H), 2.18 (s, 3H), 1.25 (t, *J* = 7.1 Hz, 3H) ppm. ^13^C NMR (101 MHz, DMSO) *δ* 167.2, 161.9, 160.8, 143.1, 137.5, 133.3, 132.0, 131.1, 129.9, 129.8, 129.0, 128.4, 127.3, 127.2, 125.7, 125.5, 123.0, 122.3, 115.2, 93.6, 59.9, 37.5, 21.6, 14.9 ppm. HRMS (ESI-TOF) *m*/*z*: [M + Na]^+^ calcd for C_24_H_22_ClN_3_O_3_Na: 458.1350; found: 458.1241. IR (KBr, cm^−1^): 3445, 3319, 3055, 2981, 1693, 1634, 1471, 1104, 1023, 761.

#### (*S*)-Methyl 4-((5-amino-3-phenylisoxazol-4-yl)(phenyl)methyl)-1*H*-pyrrole-2-carboxylate (3c)

White solid (34.3 mg, 92%); MP = 106–108 °C; the enantiomeric excess was determined to be 88% by HPLC analysis on Daicel Chiralcel AD-H column (*n*-hexane/i-PrOH = 60/40, flow rate 1.0 mL min^−1^, *T* = 25 °C), UV 254 nm; *t*_major_ = 8.294 min, *t*_minor_ = 5.563 min; [*α*]^20^_D_ = −64.4° (*c* = 0.225, CH_2_Cl_2_); ^1^H NMR (400 MHz, DMSO) *δ* 11.84 (s, 1H), 7.43–7.34 (m, 3H), 7.29 (dd, *J* = 13.4, 4.4 Hz, 4H), 7.20 (t, *J* = 7.3 Hz, 1H), 7.14 (d, *J* = 7.5 Hz, 2H), 6.60 (s, 1H), 6.47 (s, 1H), 5.92 (s, 2H), 5.12 (s, 1H), 3.72 (s, 3H) ppm. ^13^C NMR (101 MHz, DMSO) *δ* 167.9, 163.3, 161.2, 143.4, 130.7, 129.6, 128.9, 128.8, 128.6, 128.4, 126.8, 126.0, 123.2, 122.2, 115.4, 92.4, 51.5, 37.2 ppm. HRMS (ESI-TOF) *m*/*z*: [M + Na]^+^ calcd for C_22_H_19_N_3_O_3_Na: 396.1426; found: 396.1318. IR (KBr, cm^−1^): 3446, 3323, 3060, 3025, 2951, 1698, 1635, 1478, 1438, 1203, 1105, 768.

#### (*S*)-Methyl 4-((5-amino-3-phenylisoxazol-4-yl)(3-fluorophenyl)-methyl)-1*H*-pyrrole-2-carboxylate (3d)

White solid (33.3 mg, 85%); MP = 99–101 °C; the enantiomeric excess was determined to be 86% by HPLC analysis on Daicel Chiralcel AD-H column (*n*-hexane/i-PrOH = 70/30, flow rate 1.0 mL min^−1^, *T* = 25 °C), UV 254 nm; *t*_major_ = 9.079 min, *t*_minor_ = 7.475 min; [*α*]^20^_D_ = −25.0° (*c* = 0.10, CH_2_Cl_2_); ^1^H NMR (400 MHz, DMSO) *δ* 11.87 (s, 1H), 7.43–7.35 (m, 3H), 7.34–7.26 (m, 3H), 7.01 (td, *J* = 8.5, 2.4 Hz, 1H), 6.95 (d, *J* = 7.9 Hz, 1H), 6.87 (d, *J* = 10.4 Hz, 1H), 6.64 (s, 1H), 6.49 (s, 1H), 6.09 (s, 2H), 5.14 (s, 1H), 3.72 (s, 3H) ppm. ^13^C NMR (101 MHz, DMSO) *δ* 167.9, 163.3, 162.6 (d, *J* = 244.42 Hz), 161.2, 146.5 (d, *J* = 6.6 Hz), 130.7, 130.6, 129.6, 129.0, 128.7, 125.4, 124.6, 123.2, 122.3, 115.3, 115.1 (d, *J* = 21.7 Hz), 113.5 (d, *J* = 21.0 Hz), 91.9, 51.5, 37.0 ppm. ^19^F NMR (376 MHz, DMSO) *δ* −113.3 (s) ppm. HRMS (ESI-TOF) *m*/*z*: [M + H]^+^ calcd for C_22_H_19_FN_3_O_3_: 392.1332; found: 392.1405. IR (KBr, cm^−1^): 3445, 3323, 3062, 2951, 1694, 1634, 1481, 1439, 1202, 1106, 764.

#### (*S*)-Methyl 4-((5-amino-3-phenylisoxazol-4-yl)(4-methoxyphenyl)-methyl)-1*H*-pyrrole-2-carboxylate (3e)

White solid (37.9 mg, 94%); MP = 84–86 °C; the enantiomeric excess was determined to be 78% by HPLC analysis on Daicel Chiralcel AD-H column (*n*-hexane/i-PrOH = 60/40, flow rate 1.0 mL min^−1^, *T* = 25 °C), UV 254 nm; *t*_major_ = 12.503 min, *t*_minor_ = 5.937 min; [*α*]^20^_D_ = −30.9° (*c* = 0.35, CH_2_Cl_2_); ^1^H NMR (400 MHz, DMSO) *δ* 11.80 (s, 1H), 7.35 (dd, *J* = 26.4, 6.7 Hz, 5H), 7.05 (d, *J* = 8.3 Hz, 2H), 6.85 (d, *J* = 8.4 Hz, 2H), 6.58 (s, 1H), 6.46 (s, 1H), 5.83 (s, 2H), 5.05 (s, 1H), 3.72 (s, 6H) ppm. ^13^C NMR (101 MHz, DMSO) *δ* 167.8, 163.2, 161.2, 158.1, 135.3, 130.7, 129.5, 129.4, 128.9, 128.6, 126.5, 123.1, 122.2, 115.3, 114.2, 92.7, 55.5, 51.5, 36.5 ppm. HRMS (ESI-TOF) *m*/*z*: [M + Na]^+^ calcd for C_23_H_21_N_3_O_4_Na: 426.1532; found: 426.1419. IR (KBr, cm^−1^): 3459, 3325, 2971, 2952, 2837, 1698, 1635, 1509, 1478, 1248, 1106, 1033, 767.

#### (*S*)-Ethyl 4-((5-amino-3-(*p*-tolyl)isoxazol-4-yl)(*m*-tolyl)methyl)-1*H*-pyrrole-2-carboxylate (3f)

White solid (35.9 mg, 82%); MP = 121–123 °C; the enantiomeric excess was determined to be 88% by HPLC analysis on Daicel Chiralcel AD-H column (*n*-hexane/i-PrOH = 50/50, flow rate 1.0 mL min^−1^, *T* = 25 °C), UV 254 nm; *t*_major_ = 9.984 min, *t*_minor_ = 6.110 min; [*α*]^20^_D_ = −28.5° (*c* = 0.39, CH_2_Cl_2_); ^1^H NMR (400 MHz, CDCl_3_) *δ* 9.30 (s, 1H), 7.34 (d, *J* = 8.0 Hz, 2H), 7.20 (dd, *J* = 16.2, 8.0 Hz, 3H), 7.07 (d, *J* = 7.6 Hz, 1H), 7.00 (d, *J* = 5.8 Hz, 2H), 6.70 (s, 1H), 6.59 (s, 1H), 5.12 (s, 1H), 4.30 (q, *J* = 7.1 Hz, 2H), 3.96 (s, 2H), 2.37 (s, 3H), 2.32 (s, 3H), 1.34 (t, *J* = 7.1 Hz, 3H) ppm. ^13^C NMR (101 MHz, CDCl_3_) *δ* 165.1, 162.6, 160.1, 141.1, 138.2, 137.3, 128.1, 127.8, 127.4, 127.2, 126.5, 125.7, 125.6, 124.2, 122.3, 120.8, 113.8, 92.9, 59.4, 36.3, 20.4, 20.2, 13.3 ppm. HRMS (ESI-TOF) *m*/*z*: [M + Na]^+^ calcd for C_25_H_25_N_3_O_3_Na: 438.1896; found: 438.1789. IR (KBr, cm^−1^): 3425, 3295, 2964, 1694, 1633, 1471, 1104, 1020, 796.

#### (*S*)-Ethyl 4-((5-amino-3-(*p*-tolyl)isoxazol-4-yl)(3-fluorophenyl)-methyl)-1*H*-pyrrole-2-carboxylate (3g)

White solid (29.8 mg, 71%); MP = 80–82 °C; the enantiomeric excess was determined to be 84% by HPLC analysis on Daicel Chiralcel AD-H column (*n*-hexane/i-PrOH = 50/50, flow rate 1.0 mL min^−1^, *T* = 25 °C), UV 254 nm; *t*_major_ = 7.995 min, *t*_minor_ = 5.761 min; [*α*]^20^_D_ = −38.5° (*c* = 0.275, CH_2_Cl_2_); ^1^H NMR (400 MHz, CDCl_3_) *δ* 11.82 (s, 1H), 7.32 (dd, *J* = 14.3, 7.8 Hz, 1H), 7.20–7.15 (m, 4H), 7.02 (td, *J* = 8.6, 2.3 Hz, 1H), 6.95 (d, *J* = 7.8 Hz, 1H), 6.88 (d, *J* = 10.4 Hz, 1H), 6.62 (s, 1H), 6.46 (s, 1H), 6.05 (s, 2H), 5.13 (s, 1H), 4.19 (q, *J* = 7.1 Hz, 2H), 2.30 (s, 3H), 1.24 (t, *J* = 7.1 Hz, 3H) ppm. ^13^C NMR (101 MHz, CDCl_3_) *δ* 167.8, 163.2, 162.7 (d, *J* = 244.42 Hz), 160.8, 146.6 (d, *J* = 6.6 Hz), 139.1, 130.6 (d, *J* = 8.3 Hz), 129.5, 128.5, 127.7, 125.4, 124.6, 123.1, 122.6, 115.12, 115.1 (d, *J* = 21.21 Hz), 113.5 (d, *J* = 21.0 Hz), 91.9, 60.0, 37.0, 21.3, 14.9 ppm. ^19^F NMR (376 MHz, CDCl_3_) *δ* −113.2 (s) ppm. HRMS (ESI-TOF) *m*/*z*: [M + H]^+^ calcd for C_24_H_23_FN_3_O_3_: 420.1645; found: 420.1716. IR (KBr, cm^−1^): 3448, 3307, 3054, 2983, 2924, 1682, 1633, 1470, 1022, 764.

#### (*S*)-Methyl 4-((5-amino-3-(*p*-tolyl)isoxazol-4-yl)(phenyl)methyl)-1*H*-pyrrole-2-carboxylate (3h)

White solid (31.4 mg, 81%); MP = 207–209 °C; the enantiomeric excess was determined to be 86% by HPLC analysis on Daicel Chiralcel AD-H column (*n*-hexane/i-PrOH = 50/50, flow rate 1.0 mL min^−1^, *T* = 25 °C), UV 254 nm; *t*_major_ = 10.180 min, *t*_minor_ = 5.550 min; [*α*]^20^_D_ = −43.0° (*c* = 0.20, CH_2_Cl_2_); ^1^H NMR (400 MHz, DMSO) *δ* 11.84 (s, 1H), 7.29 (t, *J* = 7.5 Hz, 2H), 7.23–7.17 (m, 5H), 7.15 (t, *J* = 6.4 Hz, 2H), 6.59 (s, 1H), 6.47 (s, 1H), 5.87 (s, 2H), 5.11 (s, 1H), 3.72 (s, 3H), 2.30 (s, 3H) ppm. ^13^C NMR (101 MHz, DMSO) *δ* 167.8, 163.2, 161.2, 143.5, 139.0, 129.5, 128.8, 128.5, 128.4, 127.8, 126.8, 126.1, 123.2, 122.2, 115.4, 92.4, 51.5, 37.3, 21.3 ppm. HRMS (ESI-TOF) *m*/*z*: [M + H]^+^ calcd for C_23_H_22_N_3_O_3_: 388.1583; found: 388.1653. IR (KBr, cm^−1^): 3454, 3329, 3024, 2951, 2922, 1698, 1634, 1475, 1442, 1203, 1105, 769.

#### (*S*)-Methyl 4-((5-amino-3-(*p*-tolyl)isoxazol-4-yl)(3-fluorophenyl)-methyl)-1*H*-pyrrole-2-carboxylate (3i)

White solid (30.1 mg, 74%); MP = 86–88 °C; the enantiomeric excess was determined to be 86% by HPLC analysis on Daicel Chiralcel AD-H column (*n*-hexane/i-PrOH = 50/50, flow rate 1.0 mL min^−1^, *T* = 25 °C), UV 254 nm; *t*_major_ = 6.935 min, *t*_minor_ = 5.557 min; [*α*]^20^_D_ = −60.9° (*c* = 0.225, CH_2_Cl_2_); ^1^H NMR (400 MHz, DMSO) *δ* 11.87 (s, 1H), 7.36–7.27 (m, 1H), 7.18 (s, 4H), 7.03 (td, *J* = 8.6, 2.4 Hz, 1H), 6.96 (d, *J* = 7.8 Hz, 1H), 6.88 (d, *J* = 10.4 Hz, 1H), 6.64 (d, *J* = 2.0 Hz, 1H), 6.49 (t, *J* = 1.9 Hz, 1H), 6.05 (s, 2H), 5.13 (s, 1H), 3.73 (s, 3H), 2.30 (s, 3H) ppm. ^13^C NMR (101 MHz, DMSO) *δ* 167.8, 163.2, 162.7 (d, *J* = 244.42 Hz), 161.2, 146.6 (d, *J* = 6.6 Hz), 139.1, 130.6 (d, *J* = 8.3 Hz), 129.6, 128.5, 127.7, 125.4, 124.6, 123.2, 122.3, 115.3, 115.1 (d, *J* = 21.8 Hz), 113.5 (d, *J* = 20.8 Hz), 91.9, 51.5, 37.0, 21.3 ppm. ^19^F NMR (376 MHz, DMSO) *δ* −113.2 (s) ppm. HRMS (ESI-TOF) *m*/*z*: [M + H]^+^ calcd for C_23_H_21_FN_3_O_3_: 406.4294; found: 406.1559. IR (KBr, cm^−1^): 3457, 3329, 2952, 1701, 1637, 1481, 1202, 1105, 769.

#### (*S*)-Methyl 4-((5-amino-3-(2-chlorophenyl)isoxazol-4-yl)(phenyl)-methyl)-1*H*-pyrrole-2-carboxylate (3j)

White solid (39.1 mg, 96%); MP = 226–228 °C; the enantiomeric excess was determined to be 88% by HPLC analysis on Daicel Chiralcel AD-H column (*n*-hexane/i-PrOH = 70/30, flow rate 1.0 mL min^−1^, *T* = 25 °C), UV 254 nm; *t*_major_ = 8.281 min, *t*_minor_ = 6.887 min; [*α*]^20^_D_ = −60.0° (*c* = 0.35, CH_2_Cl_2_); ^1^H NMR (400 MHz, DMSO) *δ* 11.73 (s, 1H), 7.51–7.45 (dd, 1H), 7.41 (td, *J* = 7.8, 1.5 Hz, 1H), 7.28 (td, *J* = 7.5, 1.0 Hz, 1H), 7.21 (t, *J* = 7.2 Hz, 2H), 7.15 (t, *J* = 7.2 Hz, 1H), 7.11–7.02 (m, 3H), 6.55 (s, 1H), 6.41 (s, 1H), 6.04 (s, 2H), 4.79 (s, 1H), 3.71 (s, 3H) ppm. ^13^C NMR (101 MHz, DMSO) *δ* 167.2, 162.0, 161.2, 143.1, 133.3, 132.0, 131.2, 129.8, 128.6, 128.3, 127.3, 126.6, 125.7, 123.1, 122.1, 115.3, 100.0, 93.6, 51.4, 37.5 ppm. HRMS (ESI-TOF) *m*/*z*: [M + H]^+^ calcd for C_22_H_19_ClN_3_O_3_: 408.1037; found: 408.1104. IR (KBr, cm^−1^): 3461, 3313, 2972, 2894, 1701, 1637, 1474, 1203, 1105, 761.

#### (*S*)-Methyl 4-((5-amino-3-(2-chlorophenyl)isoxazol-4-yl)(3-fluoro-phenyl)methyl)-1*H*-pyrrole-2-carboxylate (3k)

White solid (38.3 mg, 90%); MP = 112–114 °C; the enantiomeric excess was determined to be 91% by HPLC analysis on Daicel Chiralcel AD-H column (*n*-hexane/i-PrOH = 85/15, flow rate 1.0 mL min^−1^, *T* = 25 °C), UV 254 nm; *t*_major_ = 22.040 min, *t*_minor_ = 18.943 min; [*α*]^20^_D_ = −65.8° (*c* = 019, CH_2_Cl_2_); ^1^H NMR (400 MHz, DMSO) *δ* 11.79 (s, 1H), 7.47 (dd, *J* = 8.1, 1.1 Hz, 1H), 7.41 (td, *J* = 7.7, 1.6 Hz, 1H), 7.32–7.26 (m, 1H), 7.26–7.20 (m, 1H), 7.12 (dd, *J* = 7.6, 1.4 Hz, 1H), 6.97 (td, *J* = 8.5, 2.4 Hz, 1H), 6.89 (d, *J* = 7.8 Hz, 1H), 6.79 (d, *J* = 10.5 Hz, 1H), 6.60 (s, 1H), 6.43 (s, 1H), 6.25 (s, 2H), 4.83 (s, 1H), 3.72 (s, 3H) ppm. ^13^C NMR (101 MHz, DMSO) *δ* 167.3, 162.5 (d, *J* = 244.42 Hz), 161.9, 161.2, 146.1 (d, *J* = 6.6 Hz), 131.3, 130.4 (d, *J* = 8.3 Hz), 129.8, 129.7, 127.3, 125.1, 124.6, 123.1, 122.1, 115.3, 115.0 (d, *J* = 21.8 Hz), 113.4 (d, *J* = 21.0 Hz), 93.0, 51.5, 37.2 ppm. ^19^F NMR (376 MHz, DMSO) *δ* −113.5 (s) ppm. HRMS (ESI-TOF) *m*/*z*: [M + Na]^+^ calcd for C_22_H_17_ClFN_3_O_3_Na: 448.0942; found: 448.0834. IR (KBr, cm^−1^): 3447, 3320, 2972, 2895, 1697, 1636, 1476, 1106, 772.

#### (*S*)-Methyl 4-((5-amino-3-(2-chlorophenyl)isoxazol-4-yl)(4-meth-oxyphenyl)methyl)-1*H*-pyrrole-2-carboxylate (3l)

White solid (41.5 mg, 95%); MP = 96–98 °C; the enantiomeric excess was determined to be 80% by HPLC analysis on Daicel Chiralcel AD-H column (*n*-hexane/i-PrOH = 60/40, flow rate 1.0 mL min^−1^, *T* = 25 °C), UV 254 nm; *t*_major_ = 7.531 min, *t*_minor_ = 5.738 min; [*α*]^20^_D_ = −28.4° (*c* = 0.32, CH_2_Cl_2_); ^1^H NMR (400 MHz, DMSO) *δ* 11.70 (s, 1H), 7.48 (dd, *J* = 8.0, 0.9 Hz, 1H), 7.46–7.35 (m, 1H), 7.29 (t, *J* = 7.5 Hz, 1H), 7.16–7.05 (t, 1H), 6.97 (d, *J* = 6.9 Hz, 2H), 6.77 (d, *J* = 8.3 Hz, 2H), 6.54 (s, 1H), 6.42 (s, 1H), 5.97 (s, 2H), 4.74 (s, 1H), 3.72 (s, 3H), 3.70 (s, 3H) ppm. ^13^C NMR (101 MHz, DMSO) *δ* 167.1, 161.9, 161.2, 158.0, 135.0, 133.3, 132.0, 131.1, 129.9, 129.8, 129.3, 127.3, 126.2, 123.0, 122.0, 115.3, 113.9, 94.0, 55.4, 51.4, 36.7 ppm. HRMS (ESI-TOF) *m*/*z*: [M + Na]^+^ calcd for C_23_H_20_ClN_3_O_4_Na: 460.1142; found: 460.1031. IR (KBr, cm^−1^): 3445, 3329, 3002, 2952, 1698, 1635, 1510, 1248, 1106, 761.

#### (*S*)-Ethyl 4-((5-(ethylamino)-3-phenylisoxazol-4-yl)(*m*-tolyl)-methyl)-1*H*-pyrrole-2-carboxylate (3m)

White solid (41.2 mg, 96%); MP = 72–74 °C; the enantiomeric excess was determined to be 91% by HPLC analysis on Daicel Chiralcel AD-H column (*n*-hexane/i-PrOH = 60/40, flow rate 1.0 mL min^−1^, *T* = 25 °C), UV 254 nm; *t*_major_ = 6.473 min, *t*_minor_ = 4.180 min; [*α*]^20^_D_ = −23.8° (*c* = 0.26, CH_2_Cl_2_); ^1^H NMR (400 MHz, DMSO) *δ* 11.76 (s, 1H), 7.42–7.33 (m, 3H), 7.27 (dd, *J* = 8.0, 1.4 Hz, 2H), 7.16 (t, *J* = 7.8 Hz, 1H), 7.01 (d, *J* = 7.6 Hz, 1H), 6.91 (d, *J* = 7.2 Hz, 2H), 6.57 (s, 1H), 6.43 (t, *J* = 1.8 Hz, 1H), 5.55 (t, *J* = 6.0 Hz, 1H), 5.10 (s, 1H), 4.19 (q, *J* = 7.1 Hz, 2H), 3.21 (p, *J* = 7.0 Hz, 2H), 2.22 (s, 3H), 1.24 (t, *J* = 7.1 Hz, 3H), 1.02 (t, *J* = 7.1 Hz, 3H) ppm. ^13^C NMR (101 MHz, DMSO) *δ* 167.2, 163.4, 160.8, 143.3, 137.8, 130.7, 129.5, 129.0, 128.8, 128.75, 128.6, 127.4, 126.0, 125.6, 123.1, 122.5, 115.3, 92.4, 60.0, 37.9, 37.1, 21.6, 16.1, 14.9 ppm. HRMS (ESI-TOF) *m*/*z*: [M + H]^+^ calcd for C_26_H_28_N_3_O_3_: 430.2052; found: 430.2123. IR (KBr, cm^−1^): 3388, 3302, 3054, 1704, 1622, 1488, 1101, 1024, 765.

#### (*S*)-Ethyl 4-((5-(ethylamino)-3-phenylisoxazol-4-yl)(3-fluorophe-nyl)methyl)-1*H*-pyrrole-2-carboxylate (3n)

White solid (49.9 mg, 92%); MP = 74–76 °C; the enantiomeric excess was determined to be 93% by HPLC analysis on Daicel Chiralcel AD-H column (*n*-hexane/i-PrOH = 60/40, flow rate 1.0 mL min^−1^, *T* = 25 °C), UV 254 nm; *t*_major_ = 6.697 min, *t*_minor_ = 4.509 min; [*α*]^20^_D_ = −45.5° (*c* = 0.36, CH_2_Cl_2_); ^1^H NMR (400 MHz, DMSO) *δ* 11.80 (s, 1H), 7.42–7.33 (m, 3H), 7.32–7.27 (t, 1H), 7.24 (d, *J* = 7.1 Hz, 2H), 7.01 (t, *J* = 8.1 Hz, 1H), 6.94 (d, *J* = 7.7 Hz, 1H), 6.84 (d, *J* = 10.4 Hz, 1H), 6.61 (s, 1H), 6.45 (s, 1H), 5.84 (t, *J* = 5.8 Hz, 1H), 5.16 (s, 1H), 4.19 (q, *J* = 7.0 Hz, 2H), 3.21 (dd, *J* = 13.4, 6.6 Hz, 2H), 1.24 (t, *J* = 7.1 Hz, 3H), 1.04 (t, *J* = 7.0 Hz, 3H) ppm. ^13^C NMR (101 MHz, DMSO) *δ* 167.19, 163.49, 162.62 (d, *J* = 244.42 Hz), 160.8, 146.4 (d, *J* = 6.5 Hz), 130.6, 130.5, 129.6, 128.9, 128.7, 125.2, 124.6, 123.1, 122.6, 115.2, 115.0, 113.5 (d, *J* = 21.0 Hz), 91.8, 60.0, 37.9, 36.9, 16.1, 14.8 ppm. ^19^F NMR (376 MHz, DMSO) *δ* −113.3 (s) ppm. HRMS (ESI-TOF) *m*/*z*: [M + H]^+^ calcd for C_25_H_25_FN_3_O_3_: 434.1802; found: 434.1868. IR (KBr, cm^−1^): 3389, 3302, 3061, 2978, 1704, 1622, 1488, 1192, 1103, 771.

#### (*S*)-Methyl 4-((5-(ethylamino)-3-phenylisoxazol-4-yl)(phenyl)-methyl)-1*H*-pyrrole-2-carboxylate (3o)

White solid (38.5 mg, 96%); MP = 87–89 °C; the enantiomeric excess was determined to be 90% by HPLC analysis on Daicel Chiralcel AD-H column (*n*-hexane/i-PrOH = 50/50, flow rate 1.0 mL min^−1^, *T* = 25 °C), UV 254 nm; *t*_major_ = 6.009 min, *t*_minor_ = 4.076 min; [*α*]^20^_D_ = −20.9° (*c* = 0.105, CH_2_Cl_2_); ^1^H NMR (400 MHz, DMSO) *δ* 11.82 (s, 1H), 7.41–7.31 (m, 3H), 7.27 (t, *J* = 7.3 Hz, 4H), 7.19 (t, *J* = 7.2 Hz, 1H), 7.12 (d, *J* = 7.4 Hz, 2H), 6.58 (s, 1H), 6.45 (t, *J* = 1.8 Hz, 1H), 5.54 (t, *J* = 6.0 Hz, 1H), 5.14 (s, 1H), 3.71 (s, 3H), 3.20 (p, *J* = 7.0 Hz, 2H), 1.00 (t, *J* = 7.1 Hz, 3H) ppm. ^13^C NMR (101 MHz, DMSO) *δ* 167.2, 163.4, 161.2, 143.4, 130.7, 129.5, 128.9, 128.8, 128.7, 128.4, 126.8, 125.9, 123.2, 122.2, 115.5, 92.3, 51.5, 37.9, 37.1, 16.1 ppm. HRMS (ESI-TOF) *m*/*z*: [M + H]^+^ calcd for C_24_H_24_N_3_O_3_: 402.1739; found: 402.1814. IR (KBr, cm^−1^): 3389, 3303, 3060, 3025, 2973, 1706, 1624, 1491, 1202, 1105, 1028, 769.

#### (*S*)-Methyl 4-((5-(ethylamino)-3-phenylisoxazol-4-yl)(3-fluoro phenyl)methyl)-1*H*-pyrrole-2-carboxylate (3p)

White solid (39.8 mg, 95%); MP = 64–66 °C; the enantiomeric excess was determined to be 92% by HPLC analysis on Daicel Chiralcel AD-H column (*n*-hexane/i-PrOH = 50/50, flow rate 1.0 mL min^−1^, *T* = 25 °C), UV 254 nm; *t*_major_ = 4.975 min, *t*_minor_ = 4.004 min; [*α*]^20^_D_ = −39.7° (*c* = 0.305, CH_2_Cl_2_); ^1^H NMR (400 MHz, DMSO) *δ* 11.85 (s, 1H), 7.42–7.22 (m, 6H), 7.06–6.78 (m, 3H), 6.62 (s, 1H), 6.47 (s, 1H), 5.83 (s, 1H), 5.15 (s, 1H), 3.72 (d, *J* = 1.5 Hz, 3H), 3.29–3.07 (m, 2H), 1.03 (t, *J* = 6.1 Hz, 3H) ppm. ^13^C NMR (101 MHz, DMSO) *δ* 166.5, 162.8, 161.9 (d, *J* = 244.42 Hz), 160.5, 145.7 (d, *J* = 6.3 Hz), 129.9, 129.87, 128.9, 128.2, 128.0, 124.6, 123.9, 122.5, 121.6, 114.7, 114.4 (d, *J* = 21.8 Hz), 112.8 (d, *J* = 20.7 Hz), 91.1, 50.8, 37.2, 36.2, 15.4 ppm. ^19^F NMR (376 MHz, DMSO) *δ* −113.3 (s) ppm. HRMS (ESI-TOF) *m*/*z*: [M + H]^+^ calcd for C_24_H_23_FN_3_O_3_: 420.1645; found: 420.1716. IR (KBr, cm^−1^): 3390, 3305, 3060, 2963, 1704, 1622, 1487, 1201, 1105, 1028, 773.

#### (*S*)-Methyl 4-((5-(ethylamino)-3-phenylisoxazol-4-yl)(*m*-tolyl)me-thyl)-1*H*-pyrrole-2-carboxylate (3q)

White solid (37.0 mg, 88%); MP = 107–109 °C; the enantiomeric excess was determined to be 91% by HPLC analysis on Daicel Chiralcel AD-H column (*n*-hexane/i-PrOH = 70/30, flow rate 1.0 mL min^−1^, *T* = 25 °C), UV 254 nm; *t*_major_ = 7.098 min, *t*_minor_ = 4.801 min; [*α*]^20^_D_ = −23.5° (*c* = 0.40, CH_2_Cl_2_); ^1^H NMR (400 MHz, DMSO) ^1^H NMR (400 MHz, DMSO) *δ* 11.81 (s, 1H), 7.37 (m, *J* = 16.0, 7.7, 2.4 Hz, 3H), 7.30–7.23 (m, 2H), 7.16 (t, *J* = 7.8 Hz, 1H), 7.01 (d, *J* = 7.5 Hz, 1H), 6.91 (d, *J* = 7.0 Hz, 2H), 6.58 (s, 1H), 6.45 (s, 1H), 5.54 (t, *J* = 6.0 Hz, 1H), 5.10 (s, 1H), 3.72 (s, 3H), 3.20 (q, *J* = 13.6, 6.6 Hz, 2H), 2.22 (s, 3H), 1.02 (t, *J* = 7.1 Hz, 3H) ppm. ^13^C NMR (101 MHz, DMSO) *δ* 167.2, 163.4, 161.2, 143.2, 137.8, 130.7, 129.5, 129.0, 128.8, 128.7, 128.6, 127.4, 126.1, 125.6, 123.2, 122.2, 115.4, 92.4, 51.5, 37.9, 37.1, 21.6, 16.1 ppm. HRMS (ESI-TOF) *m*/*z*: [M + H]^+^ calcd for C_25_H_26_N_3_O_3_: 416.1896; found: 416.1967. IR (KBr, cm^−1^): 3389, 3300, 2951, 1712, 1622, 1488, 1435, 1386, 1201, 1104, 767.

#### (*S*)-Methyl 4-((5-(ethylamino)-3-phenylisoxazol-4-yl)(4-fluoro-phenyl)methyl)-1*H*-pyrrole-2-carboxylate (3r)

White solid (39.0 mg, 93%); MP = 176–178 °C; the enantiomeric excess was determined to be 90% by HPLC analysis on Daicel Chiralcel AD-H column (*n*-hexane/i-PrOH = 60/40, flow rate 1.0 mL min^−1^, *T* = 25 °C), UV 254 nm; *t*_major_ = 7.066 min, *t*_minor_ = 4.333 min; [*α*]^20^_D_ = −48.9° (*c* = 0.48, CH_2_Cl_2_); ^1^H NMR (400 MHz, DMSO) *δ* 11.82 (s, 1H), 7.37 (td, *J* = 14.2, 6.8 Hz, 3H), 7.25 (d, *J* = 7.2 Hz, 2H), 7.18–7.02 (m, 4H), 6.59 (s, 1H), 6.45 (s, 1H), 5.68 (t, *J* = 5.8 Hz, 1H), 5.13 (s, 1H), 3.72 (s, 3H), 3.31–3.11 (m, 2H), 1.03 (t, *J* = 7.0 Hz, 3H) ppm. ^13^C NMR (101 MHz, DMSO) *δ* 167.2, 163.4, 161.2, 161.16 (d, *J* = 243.41 Hz), 139.4, 130.6, 130.2 (d, *J* = 7.9 Hz), 129.5, 128.9, 128.7, 125.8, 123.2, 122.3, 115.5, 115.3 (d, *J* = 9.8 Hz), 92.2, 51.5, 37.9, 36.4, 16.1 ppm. ^19^F NMR (376 MHz, DMSO) *δ* −116.99 (s) ppm. HRMS (ESI-TOF) *m*/*z*: [M + H]^+^ calcd for C_24_H_23_FN_3_O_3_: 420.1645; found: 420.1716. IR (KBr, cm^−1^): 3388, 3303, 2952, 2928, 1704, 1622, 1505, 1388, 1262, 1106, 767.

#### (*S*)-Ethyl 4-((5-(diethylamino)-3-phenylisoxazol-4-yl)(*m*-tolyl)me-thyl)-1*H*-pyrrole-2-carboxylate(3s)

White solid (30.4 mg, 27%); MP = 153–155 °C; the enantiomeric excess was determined to be 23% by HPLC analysis on Daicel Chiralcel AD-H column (*n*-hexane/i-PrOH = 90/10, flow rate 1.0 mL min^−1^, *T* = 25 °C), UV 254 nm; *t*_major_ = 24.447 min, *t*_minor_ = 18.525 min; [*α*]^20^_D_ = −22.4° (*c* = 0.30, CH_2_Cl_2_); ^1^H NMR (400 MHz, CDCl_3_) *δ* 9.37 (s, 1H), 7.33–7.26 (m, 2H), 7.24 (dd, *J* = 11.7, 4.5 Hz, 3H), 7.13 (t, *J* = 7.5 Hz, 1H), 7.04–6.92 (t, 3H), 6.71 (s, 1H), 6.58 (s, 1H), 5.29 (s, 1H), 4.31 (q, *J* = 7.1 Hz, 2H), 3.18 (ddd, *J* = 14.0, 7.0, 3.3 Hz, 4H), 2.26 (s, 3H), 1.35 (t, *J* = 7.1 Hz, 3H), 0.99 (t, *J* = 7.1 Hz, 6H) ppm. ^13^C NMR (101 MHz, CDCl_3_) *δ* 168.3, 165.7, 161.4, 142.4, 137.6, 130.7, 129.2, 128.9, 128.8, 128.0, 127.98, 127.1, 127.0, 125.6, 122.7, 122.3, 115.7, 97.5, 60.4, 44.5, 38.0, 29.7, 21.5, 14.5, 13.3 ppm. HRMS (ESI-TOF) *m*/*z*: [M + H]^+^ calcd for C_28_H_32_N_3_O_3_: 458.2365; found: 458.2435. IR (KBr, cm^−1^): 3388, 3305, 2960, 1710, 1622, 1492, 1388, 1201, 1106, 767.

#### (*S*)-Ethyl 4-((5-amino-3-(4-bromophenyl)isoxazol-4-yl)(*m*-tolyl)-methyl)-1*H*-pyrrole-2-carboxylate (3t)

White solid (34.6 mg, 72%); MP = 146–148 °C; the enantiomeric excess was determined to be 85% by HPLC analysis on Daicel Chiralcel AD-H column (*n*-hexane/i-PrOH = 50/50, flow rate 1.0 mL min^−1^, *T* = 25 °C), UV 254 nm; *t*_major_ = 8.066 min, *t*_minor_ = 5.952 min; [*α*]^20^_D_ = −31.5° (*c* = 0.445, CH_2_Cl_2_); ^1^H NMR (400 MHz, DMSO) *δ* 11.76 (s, 1H), 7.61–7.48 (m, 2H), 7.22–7.17 (m, 2H), 7.15 (d, *J* = 7.8 Hz, 1H), 7.00 (d, *J* = 7.5 Hz, 1H), 6.90 (d, *J* = 1.7 Hz, 2H), 6.65–6.51 (m, 1H), 6.42 (t, *J* = 1.9 Hz, 1H), 6.00 (s, 2H), 5.09 (s, 1H), 4.19 (q, *J* = 7.1 Hz, 2H), 2.22 (s, 3H), 1.24 (t, *J* = 7.1 Hz, 3H) ppm. ^13^C NMR (101 MHz, DMSO) *δ* 168.0, 162.4, 160.8, 143.3, 137.8, 131.9, 130.7, 130.0, 129.0, 128.7, 127.5, 125.9, 125.6, 123.1, 123.0, 122.5, 115.2, 92.5, 60.0, 37.1, 21.6, 14.9 ppm. HRMS (ESI-TOF) *m*/*z*: [M + Na]^+^ calcd for C_24_H_22_BrN_3_O_3_Na: 502.0845; found: 502.0720. IR (KBr, cm^−1^): 3455, 3311, 2975, 2925, 1693, 1634, 1473, 1196, 1104, 1013, 766.

#### (*S*)-Ethyl 4-((5-amino-3-(4-bromophenyl)isoxazol-4-yl)(3-fluoro-phenyl)methyl)-1*H*-pyrrole-2-carboxylate (3u)

Yellow solid (46.4 mg, 96%); MP = 98–100 °C; the enantiomeric excess was determined to be 82% by HPLC analysis on Daicel Chiralcel AD-H column (*n*-hexane/i-PrOH = 60/40, flow rate 1.0 mL min^−1^, *T* = 25 °C), UV 254 nm; *t*_major_ = 9.937 min, *t*_minor_ = 8.193 min; [*α*]^20^_D_ = −37.1° (*c* = 0.21, CH_2_Cl_2_); ^1^H NMR (400 MHz, DMSO) *δ* 11.81 (s, 1H), 7.64–7.50 (m, 2H), 7.31 (td, *J* = 8.0, 6.4 Hz, 1H), 7.24–7.17 (m, 2H), 7.02 (td, *J* = 8.5, 2.4 Hz, 1H), 6.96 (d, *J* = 7.8 Hz, 1H), 6.88 (d, *J* = 10.4 Hz, 1H), 6.70–6.55 (t, 1H), 6.45 (t, *J* = 1.9 Hz, 1H), 6.18 (s, 2H), 5.16 (s, 1H), 4.20 (q, *J* = 7.1 Hz, 2H), 1.25 (t, *J* = 7.1 Hz, 3H) ppm. ^13^C NMR (101 MHz, DMSO) *δ* 168.1, 162.6 (d, *J* = 244.42 Hz), 162.4, 160.8, 146.4 (d, *J* = 6.6 Hz), 131.9, 130.7, 130.6, 129.9, 125.2, 124.6, 123.1, 123.1, 122.7, 115.2, 115.1 (d, *J* = 22.22 Hz), 113.6 (d, *J* = 20.9 Hz), 91.9, 60.1, 36.9, 14.9 ppm. ^19^F NMR (376 MHz, DMSO) *δ* −113.20 (s). HRMS (ESI-TOF) *m*/*z*: [M + Na]^+^ calcd for C_23_H_19_BrFN_3_O_3_Na: 507.0845; found: 507.0471. IR (KBr, cm^−1^): 3452, 3322, 2976, 2930, 1694, 1634, 1480, 1198, 1104, 772.

#### (*S*)-Ethyl 4-((5-amino-3-(3-chlorophenyl)isoxazol-4-yl)(*m*-tolyl)-methyl)-1*H*-pyrrole-2-carboxylate (3v)

White solid (41.8 mg, 96%); MP = 221–223 °C; the enantiomeric excess was determined to be 80% by HPLC analysis on Daicel Chiralcel AD-H column (*n*-hexane/i-PrOH = 50/50, flow rate 1.0 mL min^−1^, *T* = 25 °C), UV 254 nm; *t*_major_ = 5.057 min, *t*_minor_ = 4.288 min; [*α*]^20^_D_ = −26.3° (*c* = 0.255, CH_2_Cl_2_); ^1^H NMR (400 MHz, DMSO) *δ* 11.78–11.61 (s, 1H), 7.45 (ddd, *J* = 8.1, 2.0, 1.1 Hz, 1H), 7.38 (t, *J* = 7.8 Hz, 1H), 7.25–7.20 (m, 1H), 7.16 (dd, *J* = 13.1, 4.9 Hz, 2H), 7.01 (d, *J* = 7.5 Hz, 1H), 6.92 (d, *J* = 1.8 Hz, 2H), 6.56 (s, 1H), 6.41 (s, 1H), 6.09 (d, *J* = 2.9 Hz, 2H), 5.12 (s, 1H), 4.19 (q, *J* = 7.0 Hz, 2H), 2.23 (s, 3H), 1.24 (t, *J* = 7.1 Hz, 3H) ppm. ^13^C NMR (101 MHz, DMSO) *δ* 168.1, 162.1, 160.8, 143.3, 137.8, 133.4, 132.8, 130.7, 129.4, 129.0, 128.7, 128.5, 127.5, 127.4, 125.9, 125.6, 123.1, 122.5, 115.2, 92.6, 60.0, 37.1, 21.6, 14.9 ppm. HRMS (ESI-TOF) *m*/*z*: [M + Na]^+^ calcd for C_24_H_22_ClN_3_O_3_Na: 458.1350; found: 458.1242. IR (KBr, cm^−1^): 3445, 3319, 2968, 2925, 1693, 1634, 1477, 1441, 1196, 1103, 1022, 793.

#### (*S*)-Ethyl 4-((5-amino-3-(3-chlorophenyl)isoxazol-4-yl)(3-fluoro-phenyl)methyl)-1*H*-pyrrole-2-carboxylate (3w)

Yellow solid (40.5 mg, 92%); MP = 169–171 °C; the enantiomeric excess was determined to be 84% by HPLC analysis on Daicel Chiralcel AD-H column (*n*-hexane/i-PrOH = 80/20, flow rate 1.0 mL min^−1^, *T* = 25 °C), UV 254 nm; *t*_major_ = 15.854 min, *t*_minor_ = 12.522 min; [*α*]^20^_D_ = −42.1° (*c* = 0.34, CH_2_Cl_2_); ^1^H NMR (400 MHz, DMSO) *δ* 11.81 (s, 1H), 7.46 (ddd, *J* = 8.0, 1.9, 1.0 Hz, 1H), 7.39 (t, *J* = 7.8 Hz, 1H), 7.32 (dd, *J* = 14.3, 7.9 Hz, 1H), 7.22 (d, *J* = 7.7 Hz, 1H), 7.15 (t, *J* = 1.6 Hz, 1H), 7.03 (td, *J* = 8.6, 2.4 Hz, 1H), 6.96 (d, *J* = 7.8 Hz, 1H), 6.86 (d, *J* = 10.5 Hz, 1H), 6.61 (d, *J* = 1.9 Hz, 1H), 6.44 (t, *J* = 1.9 Hz, 1H), 6.25 (s, 2H), 5.19 (s, 1H), 4.19 (q, *J* = 7.0 Hz, 2H), 1.24 (t, *J* = 7.1 Hz, 3H) ppm. ^13^C NMR (101 MHz, DMSO) *δ* 168.2, 162.6 (d, *J* = 244.42 Hz), 162.1, 160.8, 146.4 (d, *J* = 6.5 Hz), 133.5, 132.7, 130.8, 130.6 (d, *J* = 8.3 Hz), 129.4, 128.5, 127.4, 125.2, 124.6, 123.1, 122.7, 115.12 (d, *J* = 22.22 Hz), 115.11, 113.6 (d, *J* = 20.9 Hz), 92.0, 60.0, 36.8, 14.8 ppm. ^19^F NMR (376 MHz, DMSO) *δ* −113.23 (s). HRMS (ESI-TOF) *m*/*z*: [M + Na]^+^ calcd for C_23_H_19_ClFN_3_O_3_Na: 462.1099; found: 462.0992. IR (KBr, cm^−1^): 3445, 3320, 2982, 2933, 1698, 1633, 1485, 1441, 1196, 1104, 776.

#### (*S*)-Methyl 4-((5-amino-3-phenylisoxazol-4-yl)(4-fluorophenyl)-methyl)-1*H*-pyrrole-2-carboxylate (3x)

White solid (33.7 mg, 86%); MP = 158–160 °C; the enantiomeric excess was determined to be 81% by HPLC analysis on Daicel Chiralcel AD-H column (*n*-hexane/i-PrOH = 60/40, flow rate 1.0 mL min^−1^, *T* = 25 °C), UV 254 nm; *t*_major_ = 7.508 min, *t*_minor_ = 4.992 min; [*α*]^20^_D_ = −75.8° (*c* = 0.215, CH_2_Cl_2_); ^1^H NMR (400 MHz, DMSO) *δ* 11.86 (s, 1H), 7.43–7.35 (m, 3H), 7.29 (dd, *J* = 7.9, 1.6 Hz, 2H), 7.16–7.07 (m, 4H), 6.67–6.58 (t, 1H), 6.49 (t, *J* = 1.9 Hz, 1H), 6.00 (s, 2H), 5.12 (s, 1H), 3.73 (s, 3H) ppm. ^13^C NMR (101 MHz, DMSO) *δ* 167.8, 163.3, 161.2, 161.1 (d, *J* = 243.41 Hz), 139.5 (d, *J* = 2.6 Hz), 130.7, 130.2 (d, *J* = 8.0 Hz), 129.6, 128.9, 128.6, 126.0, 123.2, 122.3, 115.5, 115.3, 92.3, 51.5, 36.5 ppm. ^19^F NMR (376 MHz, DMSO) *δ* −116.96 (s). HRMS (ESI-TOF) *m*/*z*: [M + Na]^+^ calcd for C_22_H_18_FN_3_O_3_Na: 414.1332; found: 414.1225. IR (KBr, cm^−1^): 3451, 3323, 2972, 2894, 1698, 1634, 1479, 1439, 1106, 1014, 768.

#### (*S*)-Methyl 4-((5-amino-3-(2-chlorophenyl)isoxazol-4-yl)(4-fluoro-phenyl)methyl)-1*H*-pyrrole-2-carboxylate (3y)

White solid (41.5 mg, 91%); MP = 107–109 °C; the enantiomeric excess was determined to be 86% by HPLC analysis on Daicel Chiralcel AD-H column (*n*-hexane/i-PrOH = 70/30, flow rate 1.0 mL min^−1^, *T* = 25 °C), UV 254 nm; *t*_major_ = 7.563 min, *t*_minor_ = 6.242 min; [*α*]^20^_D_ = −89.7° (*c* = 0.59, CH_2_Cl_2_); ^1^H NMR (400 MHz, DMSO) *δ* 11.74 (s, 1H), 7.46 (d, *J* = 7.2 Hz, 1H), 7.40 (td, *J* = 7.8, 1.5 Hz, 1H), 7.28 (td, *J* = 7.4, 1.0 Hz, 1H), 7.12–6.97 (m, 5H), 6.56 (s, 1H), 6.41 (s, 1H), 6.13 (s, 2H), 4.81 (s, 1H), 3.71 (s, 3H) ppm. ^13^C NMR (101 MHz, DMSO) *δ* 167.2, 161.9, 161.2, 161.1 (d, *J* = 243.41 Hz), 139.2, 133.4, 132.0, 131.2, 130.2, 130.1, 129.8, 127.3, 125.7, 123.0, 122.2, 115.3, 115.1, 93.5, 51.5, 36.8 ppm. ^19^F NMR (376 MHz, DMSO) *δ* −117.16 (s). HRMS (ESI-TOF) *m*/*z*: [M + H]^+^ calcd for C_22_H_18_ClFN_3_O_3_: 426.0942; found: 426.1020. IR (KBr, cm^−1^): 3450, 3326, 2977, 2895, 1699, 1636, 1474, 1390, 1221, 1106, 764.

#### (*S*)-Methyl 4-((5-amino-3-(4-bromophenyl)isoxazol-4-yl)(3-fluoro-phenyl)methyl)-1*H*-pyrrole-2-carboxylate (3z)

Yellow solid (47.7 mg, 97%); MP = 230–232 °C; the enantiomeric excess was determined to be 84% by HPLC analysis on Daicel Chiralcel AD-H column (*n*-hexane/i-PrOH = 60/40, flow rate 1.0 mL min^−1^, *T* = 25 °C), UV 254 nm; *t*_major_ = 8.902 min, *t*_minor_ = 7.556 min; [*α*]^20^_D_ = −53.6° (*c* = 0.345, CH_2_Cl_2_); ^1^H NMR (400 MHz, DMSO) *δ* 11.85 (s, 1H), 7.56 (d, *J* = 8.4 Hz, 2H), 7.30 (dd, *J* = 14.3, 7.9 Hz, 1H), 7.20 (d, *J* = 8.4 Hz, 2H), 7.02 (td, *J* = 8.5, 2.3 Hz, 1H), 6.94 (d, *J* = 7.8 Hz, 1H), 6.86 (d, *J* = 10.4 Hz, 1H), 6.63 (s, 1H), 6.46 (s, 1H), 6.15 (d, *J* = 2.6 Hz, 2H), 5.14 (s, 1H), 3.72 (s, 3H) ppm. ^13^C NMR (101 MHz, DMSO) *δ* 168.1, 162.6 (d, *J* = 244.42 Hz), 162.4, 161.2, 146.3 (d, *J* = 6.6 Hz), 131.9, 130.7, 130.6, 129.9, 125.3, 124.6, 123.2, 123.1, 122.3, 115.3, 115.1 (d, *J* = 21.9 Hz), 113.6 (d, *J* = 21.0 Hz), 91.9, 51.5, 36.8 ppm. ^19^F NMR (376 MHz, DMSO) *δ* −113.23 (s). HRMS (ESI-TOF) *m*/*z*: [M + Na]^+^ calcd for C_22_H_17_BrFN_3_O_3_Na: 492.0437; found: 492.0331. IR (KBr, cm^−1^): 3449, 3315, 2952, 2926, 1700, 1635, 1474, 1439, 1201, 1105, 771.

#### (*S*)-Methyl 4-((5-amino-3-(3-chlorophenyl)isoxazol-4-yl)(phenyl)-methyl)-1*H*-pyrrole-2-carboxylate (3za)

White solid (39.4 mg, 96%); MP = 168–170 °C; the enantiomeric excess was determined to be 84% by HPLC analysis on Daicel Chiralcel AD-H column (*n*-hexane/i-PrOH = 50/50, flow rate 1.0 mL min^−1^, *T* = 25 °C), UV 254 nm; *t*_major_ = 8.911 min, *t*_minor_ = 7.148 min; [*α*]^20^_D_ = −44.0° (*c* = 0.25, CH_2_Cl_2_); ^1^H NMR (400 MHz, DMSO) *δ* 11.83 (s, 1H), 7.44 (ddd, *J* = 8.1, 2.1, 1.1 Hz, 1H), 7.37 (t, *J* = 7.8 Hz, 1H), 7.31–7.24 (m, 3H), 7.22 (dt, *J* = 9.4, 1.7 Hz, 1H), 7.19–7.16 (t, 1H), 7.13 (d, *J* = 7.4 Hz, 2H), 6.65–6.51 (m, 1H), 6.44 (t, *J* = 1.9 Hz, 1H), 6.09 (s, 2H), 5.17 (s, 1H), 3.71 (s, 4H) ppm. ^13^C NMR (101 MHz, DMSO) *δ* 168.2, 162.1, 161.2, 143.3, 133.5, 132.8, 130.7, 129.4, 128.8, 128.5, 128.4, 127.3, 126.8, 125.9, 123.2, 122.2, 115.4, 92.5, 51.5, 37.1 ppm. HRMS (ESI-TOF) *m*/*z*: [M + Na]^+^ calcd for C_22_H_18_ClN_3_O_3_Na: 430.1037; found: 430.0928. IR (KBr, cm^−1^): 3446, 3323, 3026, 2970, 2895, 1704, 1633, 1479, 1440, 1203, 1104, 773.

### Procedure for the 1.0 mmol scale reaction

1*H*-Pyrrol-3-yl carbinol 1a (260 mg, 1 mmol), 3-arylisoxazol-5-amine 2b or 2d (1.2 mmol), chiral phosphoric acid (*S*)-4.1i (34 mg, 10 mol%, 0.1 mmol) and 4 Å MS (500 mg) were added to a dried tube. Then, dichloroethane (5 mL) was added to the reaction mixture, which was stirred at 25 °C for 12 h. After the completion of the reaction which was indicated by TLC, the reaction mixture was directly purified by column chromatography using PE : EA (5 : 1) as eluent to afford the pure product 3b (357 mg, 78% yield, 90% ee) or 3m (374 mg, 87% yield, 90% ee).

### Procedure for the derivatization experiment

3k (127.5 mg, 0.3 mmol) and NaOH (58.5 mg, 1.5 mmol) were stirred in THF : MeOH : H_2_O = 2 : 2 : 0.5 (15 mL) at reflux temperature for 5 hours. Adjust the pH of the reaction solution to 2 using dilute hydrochloric acid solution (1 M). The reaction mixture was quenched with water and extracted with EtOAc, and then the organic layer was washed with brine and dried with anhydrous sodium sulfate and evaporated under reduced pressure. The crude product was purified by column chromatography (EA : PE : MeOH = 1 : 1 : 0.5) to afford compound 4a.

#### (*S*)-4-((5-Amino-3-(2-chlorophenyl)isoxazol-4-yl)(3-fluorophenyl)-methyl)-1*H*-pyrrole-2-carboxylic acid (4a)

White solid (118.6 mg, 96%); MP = 167–169 °C; the enantiomeric excess was determined to be 92% by HPLC analysis on Daicel Chiralcel AD-H column (*n*-hexane/i-PrOH = 80/20, flow rate 1.0 mL min^−1^, *T* = 25 °C), UV 254 nm; *t*_major_ = 30.01 min, *t*_minor_ = 22.823 min; [*α*]^20^_D_ = −77.436° (*c* = 0.39, CH_2_Cl_2_); ^1^H NMR (400 MHz, DMSO) *δ* 11.48 (s, 1H), 7.55–7.34 (m, 2H), 7.34–7.15 (m, 2H), 7.10 (d, *J* = 6.4 Hz, 1H), 7.00–6.82 (m, 2H), 6.77 (d, *J* = 9.5 Hz, 1H), 6.47 (s, 1H), 6.35 (s, 1H), 6.12 (s, 2H), 4.81 (s, 1H) ppm. ^13^C NMR (101 MHz, DMSO) *δ* 167.3, 163.0, 162.4 (d, *J* = 243.41 Hz), 161.9, 146.3 (d, *J* = 6.5 Hz), 133.3, 132.0, 131.2, 130.3 (d, *J* = 8.2 Hz), 129.8, 129.75, 127.3, 124.9, 124.6, 124.5, 121.6, 115.0 (d, *J* = 21.5 Hz), 114.1, 113.3 (d, *J* = 21.0 Hz), 93.2, 37.4 ppm. ^19^F NMR (376 MHz, DMSO) *δ* −113.64 (s) ppm. HRMS (ESI-TOF) *m*/*z*: [M + H]^+^ calcd for C_21_H_16_ClFN_3_O_3_: 411.0786; found: 412.0865. IR (KBr, cm^−1^): 3445, 3306, 3061, 2925, 1930, 1683, 1636, 1485, 1441, 1129, 773.

## Conflicts of interest

There are no conflicts to declare.

## Supplementary Material

RA-013-D3RA03480A-s001

RA-013-D3RA03480A-s002
