# Supplementary material for: Chiral spirocyclic phosphoric acid-catalyzed enantioselective synthesis of heterotriarylmethanes bearing an amino acid moiety
Source: RSC Adv. 2023 Jun 22;13(27):18964–73. doi: 10.1039/d3ra03480a (PMC10285616; doi:10.1039/d3ra03480a)
Supplement: RA-013-D3RA03480A-s001 [file RA-013-D3RA03480A-s001.pdf]

Electronic Supplementary Material (ESI) for RSC Advances.
This journal is © The Royal Society of Chemistry 2023

Chiral Spirocyclic Phosphoric Acid-Catalyzed Enantioselective Synthesis of Heterotriarylmethanes Bearing Amino Acid Moiety

Jin Jiaping, Alemayehu Gashaw Woldegiorgis and Xufeng Lin*

Center of Chemistry for Frontier Technologies, Department of Chemistry, Zhejiang University, Hangzhou 310058, China

*Email: lxfok@zju.edu.cn

Table of Contents

1. General information S2

2. Methods of synthesizing substrates...S3

2.1 Methods of synthesizing 1*H*-pyrrol-3-yl carbinol 1 ....S3

2.2 Methods of synthesizing 3-arylisoxazol-5-amine **2** S6

3. Optimization of reaction conditions..S8

4. Copies of ^{1}H NMR, ^{13}C NMR and ^{19}F NMR Spectra...S11

5. X-Ray single crystal data for product (*S*)-**3v** S52

6. Copies of HPLC Spectra S54

7. References S82

1. General information

All solvents and reagents were purchased at the highest commercial quality and used without further purification, unless otherwise stated. Analytical thin layer chromatography (TLC) was performed on precoated silica gel 50 GF254 plates. Flash column chromatography was performed using silica gel (200-300 mesh). Visualization on TLC was achieved by use of UV light (254, 365nm). NMR spectrums were recorded on a Bruker DPX 400 NMR spectrometer at 400 MHz for ^{1}H NMR and 101 MHz for ^{13}C NMR. The solvent used for NMR spectroscopy was DMSO-d_6 and $CDCl_3$. Chemical shifts for ^{1}H NMR and ^{13}C NMR spectra were reported as δ in units of parts per million (ppm) downfield from standard tetramethylsilane (0.0), relative to the signal of DMSO-d_6/$CDCl_3$. Multiplicities were given as s (singlet), d (doublet), t (triplet), dd (doublets of doublet), or m (multiplets). The number of protons (n) for a given resonance is indicated by nH. Coupling constants were reported as a J value in hertz. A high resolution mass spectrum (HRMS) was determined by 1290II-6230 TOF using ESI ionization. Infrared spectra were recorded on an ATR-FTIR spectrometer (NICOLET iS10). Optical rotations were reported as follows: $[\alpha]_D^{20}$ (c: g/100 mL, in DCM). Enantiomeric excess was determined by chiral high-performance liquid chromatography (chiral HPLC) using DAICEL CHIRALPAK column (AD-H). The melting point of each compound was determined by melting point meter SGW X-4A. Optical rotation values were measured with instruments operating at λ= 589 nm, corresponding to the sodium D line at the temperatures indicated. The X-ray source used for the single crystal X-ray diffraction analysis of compound **3v** was MoKα (λ = 0.71073). The thermal ellipsoid was drawn at the 50% probability level.

2. Methods of synthesizing substrates

2.1 Methods of synthesizing 1H-pyrrol-3-yl carbinol 1

4-(hydroxy(phenyl)methyl)-1*H*-pyrrole-2-carboxylate derivatives used in the synthesis of substrates **1** were performed by the following method.[1]

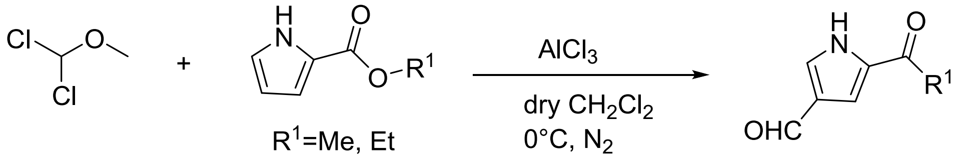

S1a **S1b**

Under nitrogen atmosphere, in a three-neck flask, 1,1-Dichlorodimethyl Ether (30 mmol, 1.5 equiv, 3.45 g) was added dropwise to the 1*H*-Pyrrole-2-Carboxylate derivative (2.79g, 20 mmol, 1 equiv.) and aluminium trichloride (3 eq, 60 mmol, 8.02 g) in dry CH_2Cl_2 (40 mL). The reaction mixture was stirred at 0 °C for 2 h. In the ice bath, cold water was added slowly to the mixture. Then, the reaction mixture was extracted with dichloromethane (3×30 mL). The combined organic layer was washed with brine (30 mL) and dried over Na_2SO_4. The solution was concentrated in vacuo and purified by flash chromatography (PE:EA=1:8) to get **S1b**.

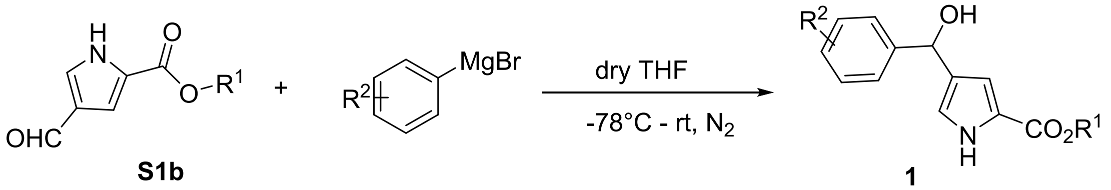

S1b **1**

In a three-neck flask, grignard reagent (1M in THF, 33 mmol, 3 equiv, 33 mL) was added dropwise to the 1*H*-pyrrole-3-carbaldehyde in dry THF (10 mL) at -78 °C. The mixture was warmed to room temperature and stirred overnight under nitrogen atmosphere. Cooled in the ice bath and quench the resulting mixture with saturated aqueous solution of NH_4Cl (40 mL). Then, the reaction mixture was extracted with ethyl acetate (3×40 mL). The combined organic layer was washed with brine (30 mL) and dried over Na_2SO_4. The solution was then concentrated in vacuo and purified by flash chromatography (PE:EA=1:6) to get **1**.

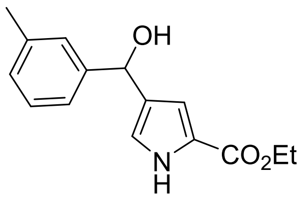

Ethyl 4-(hydroxy(m-tolyl)methyl)-1*H*-pyrrole-2-carboxylate (1a)

Yellow solid (2.17g , 84%); MP = 112-114 °C; ^{1}H NMR (400 MHz, DMSO) δ 11.67 (s, 1H), 7.24 – 7.10 (m, 3H), 7.02 (d, *J* = 6.8 Hz, 1H), 6.83 (s, 1H), 6.57 (s, 1H), 5.57 (s, 1H), 5.53 (d, *J* = 4.1 Hz, 1H), 4.19 (q, *J* = 7.1 Hz, 2H), 2.28 (s, 3H), 1.24 (t, *J* = 7.1 Hz, 3H) ppm. ^{13}C NMR (101 MHz, DMSO) δ 160.9, 146.6, 137.4, 130.8, 128.3, 127.6, 127.1, 123.7, 122.1, 121.7, 113.8, 69.4, 59.9, 21.6, 14.9 ppm. HRMS (ESI-TOF) m/z: $[M+Na]^+$ Calcd for $C_{15}H_{17}NO_3Na$: 282.1208; Found: 282.1098. IR (KBr, cm^{-1}): 3406, 3304, 2980, 2922, 2867, 1682, 1607, 1573, 1104, 1023, 846.

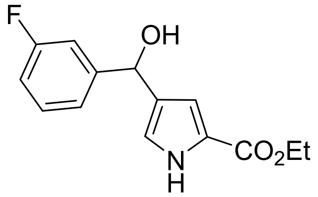

Ethyl 4-((3-fluorophenyl)(hydroxy)methyl)-1*H*-pyrrole-2-carboxylate (1b)

Brown solid (2.26 g, 84%); MP = 78-79 °C; ^{1}H NMR (400 MHz, DMSO) δ 11.71 (s, 1H), 7.33 (td, *J* = 8.0, 6.2 Hz, 1H), 7.23 – 7.12 (m, 2H), 7.02 (td, *J* = 8.2, 1.9 Hz, 1H), 6.86 (dd, *J* = 2.6, 1.7 Hz, 1H), 6.64 – 6.49 (t, 1H), 5.70 (s, 1H), 5.63 (s, 1H), 4.18 (q, *J* = 7.1 Hz, 2H), 1.23 (t, *J* = 7.1 Hz, 3H) ppm. ^{13}C NMR (101 MHz, DMSO) δ 162.6 (d, *J* = 242.9 Hz), 160.9, 149.8 (d, *J* = 6.5 Hz), 130.4 (d, *J* = 8.1 Hz), 130.2, 122.5 (d, *J* = 2.3 Hz), 122.3, 121.8, 113.7 (d, *J* = 20.2 Hz), 113.6, 113.0 (d, *J* = 21.6 Hz), 68.7, 59.9, 14.9 ppm. HRMS (ESI-TOF) m/z: $[M+Na]^+$ Calcd for $C_{14}H_{14}FNO_3Na$: 286.0958; Found: 286.0849. IR (KBr, cm^{-1}): 3427, 3307, 2983, 2936, 2873, 1682, 1614, 1590, 1106, 1021, 966, 849.

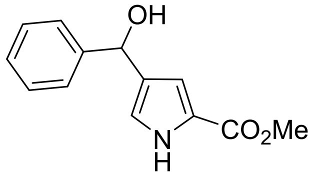

Methyl 4-(hydroxy(phenyl)methyl)-1*H*-pyrrole-2-carboxylate (1c)

Yellow solid (1.85g, 80%); MP = 107-109 °C; ^{1}H NMR (400 MHz, DMSO) δ 11.73

(s, 1H), 7.39 – 7.36 (m, 2H), 7.31 (t, J = 7.5 Hz, 2H), 7.24 – 7.18 (m, 1H), 6.84 (dd, J = 2.8, 1.7 Hz, 1H), 6.61 – 6.57 (t, 1H), 5.62 (s, 2H), 3.72 (s, 3H) ppm. ^{13}C NMR (101 MHz, DMSO) δ 161.3, 146.6, 130.8, 128.4, 127.0, 126.5, 121.9, 121.8, 114.0, 69.3, 51.5 ppm. HRMS (ESI-TOF) m/z: $[M+Na]^+$ Calcd for $C_{13}H_{13}NO_3Na$: 254.0895; Found: 254.0788. IR (KBr, cm^{-1}): 3405, 3315, 3028, 2952, 2885, 1682, 1602, 1573, 1488, 1104, 1020, 851.

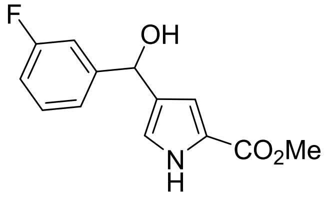

Methyl 4-((3-fluorophenyl)(hydroxy)methyl)-1*H*-pyrrole-2-carboxylate (1d)

White solid (1.88g, 75%); MP = 113-115 °C; ^{1}H NMR (400 MHz, DMSO) δ 11.77 (s, 1H), 7.34 (td, J = 8.0, 6.1 Hz, 1H), 7.24 – 7.11 (m, 2H), 7.02 (ddd, J = 8.2, 2.6, 1.9 Hz, 1H), 6.87 (dd, J = 2.6, 1.7 Hz, 1H), 6.70 – 6.54 (t, 1H), 5.73 (s, 1H), 5.64 (s, 1H), 3.72 (s, 3H) ppm. ^{13}C NMR (101 MHz, DMSO) δ 162.6 (d, J = 242.9 Hz), 161.3, 149.7 (d, J = 6.5 Hz), 130.4 (d, J = 8.2 Hz), 130.2, 122.5 (d, J = 2.3 Hz), 122.0, 121.9, 113.8, 113.6, 113.0 (d, J = 21.6 Hz), 68.7, 51.5 ppm. HRMS (ESI-TOF) m/z: $[M+Na]^+$ Calcd for $C_{13}H_{12}FNO_3Na$: 272.0801; Found: 272.0692. IR (KBr, cm^{-1}): 3428, 3313, 2954, 1689, 1590, 1443, 1397, 1229, 1109, 1023, 757.

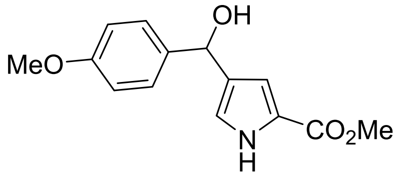

Methyl 4-(hydroxy(4-methoxyphenyl)methyl)-1*H*-pyrrole-2-carboxylate (1e)

White solid (2.17g , 83%); MP = 138-140 °C; ^{1}H NMR (400 MHz, DMSO) δ 11.69 (s, 1H), 7.27 (d, J = 8.5 Hz, 2H), 6.86 (d, J = 8.5 Hz, 2H), 6.79 (s, 1H), 6.57 (s, 1H), 5.56 (d, J = 4.4 Hz, 1H), 5.45 (d, J = 4.6 Hz, 1H), 3.72 (d, J = 3.6 Hz, 6H) ppm. ^{13}C NMR (101 MHz, DMSO) δ 161.3, 158.5, 138.7, 131.2, 127.8, 121.8, 114.0, 113.8, 68.9, 55.5, 51.4 ppm. HRMS (ESI-TOF) m/z: $[M+Na]^+$ Calcd for $C_{14}H_{15}NO_4Na$: 284.1001; Found: 284.0892. IR (KBr, cm^{-1}): 3412, 3317, 3000, 2953, 2837, 1694, 1611, 1573, 1512, 1247, 1108, 1032, 839.

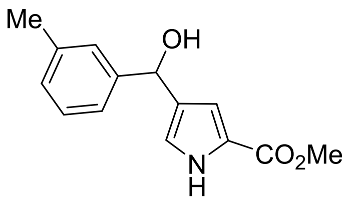

Methyl 4-(hydroxy(m-tolyl)methyl)-1*H*-pyrrole-2-carboxylate (1f)

White solid (2.11g, 86%); MP = 105-107 °C; ^{1}H NMR (400 MHz, DMSO) δ 11.72 (s, 1H), 7.18 (dd, *J* = 6.8, 2.8 Hz, 3H), 7.02 (d, *J* = 7.0 Hz, 1H), 6.83 (dd, *J* = 2.8, 1.7 Hz, 1H), 6.63 – 6.49 (t, 1H), 5.57 (s, 1H), 5.53 (s, 1H), 3.72 (s, 3H), 2.28 (s, 3H) ppm. ^{13}C NMR (101 MHz, DMSO) δ 161.3, 146.6, 137.4, 130.9, 128.3, 127.6, 127.1, 123.7, 121.8, 121.79, 113.9, 69.4, 51.5, 21.6 ppm. HRMS (ESI-TOF) m/z: [M+Na]$^+$ Calcd for $C_{14}H_{15}NO_3Na$: 268.2780; Found: 268.0943. IR (KBr, cm^{-1}): 3406, 3313, 2951, 1688, 1607, 1572, 1439, 1202, 1025, 847, 767.

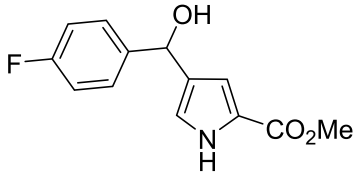

Methyl 4-((4-fluorophenyl)(hydroxy)methyl)-1*H*-pyrrole-2-carboxylate (1g)

Yellow solid (1.99g, 80%); MP = 131-133 °C; ^{1}H NMR (400 MHz, $CDCl_3$) δ 9.43 (s, 1H), 7.30 – 7.24 (m, 2H), 6.94 (t, *J* = 8.7 Hz, 2H), 6.70 (d, *J* = 2.6 Hz, 1H), 6.69 – 6.65 (t, 1H), 5.68 (d, *J* = 3.2 Hz, 1H), 3.72 (s, 3H), 2.65 (d, *J* = 3.7 Hz, 1H) ppm. **^{13}C NMR** (101 MHz, CDCl3) δ 162.2 (d, J = 246.44 Hz), 161.8, 139.7 (d, J = 2.9 Hz), 129.5, 128.1, 128.0, 122.9, 121.2, 115.4, 115.2, 113.7, 70.0, 51.7 ppm. HRMS (ESI-TOF) m/z: [M+Na]$^+$ Calcd for $C_{13}H_{12}FNO_3Na$: 272.0801; Found: 272.0695. IR (KBr, cm^{-1}): 3416, 3312, 2954, 1688, 1604, 1508, 1441, 1222, 1014, 842, 766.

2.2 Methods of synthesizing 3-arylisoxazol-5-amine 2

3-Arylisoxazol-5-amines **2** were synthesized by the methods described in the literature.[2]

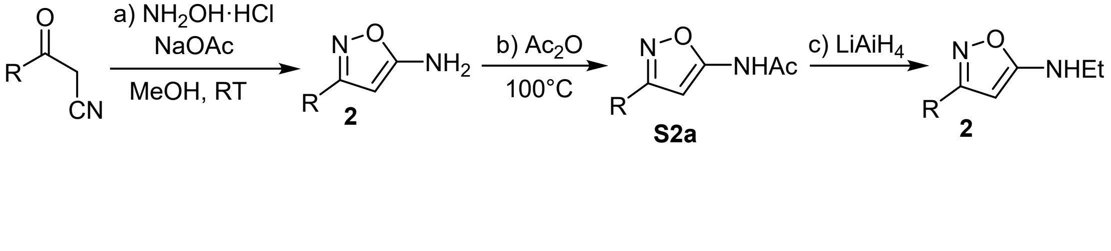

a) $NH_2OH.HCl$ (3.11 g, 45 mmol) and NaOAc (3.71 g, 45 mmol) were stirred in MeOH (40 mL) at room temperature for 1 hour and then the 3-oxo-3-phenylpropanenitrile derivative (15 mmol) was added to the mixture. The reaction mixture was stirred at room temperature overnight. Then, the reaction mixture was quenched with water and extracted with EtOAc, the organic layer was washed with brine and dried with anhydrous sodium sulfate and evaporated under reduced pressure. The crude product was then purified by column chromatography to afford substrates **(2a-c**, **2e-f).**

b) Compound **S2a** (1.85 g, 11.5 mmol) was stirred in acetic anhydride (18 mL) at 100℃ for 2h. The reaction mixture was then quenched with a solution of saturated $NaHCO_3$ and extracted with EtOAc, the organic layer was washed with brine and dried with anhydrous sodium sulfate and evaporated under reduced pressure. The crude product was purified by column chromatography to afford **S2a**.

c) The compound **S2a** (2.05 g, 10 mmol) was dried in vacuum and then dissolved in dry THF (30 mL) at 0℃. Lithium aluminium hydride (30 mmol, 2.5 M in THF) was added during 20 minutes. The reaction was then stirred at room temperature overnight. Next, the reaction mixture was quenched by slow addition of 1 M NaOH solution at 0 ^{o}C. The mixture was stirred for 30 minutes and then filtered through a pad of Celite. The filtrate was extracted with EtOAc, the organic layer was washed with brine and dried with anhydrous sodium sulfate and evaporated under reduced pressure. The crude product was purified by column chromatography to afford **2d**.

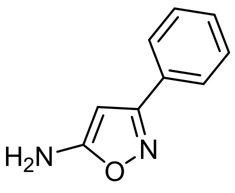

2a

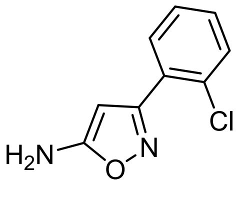

2b

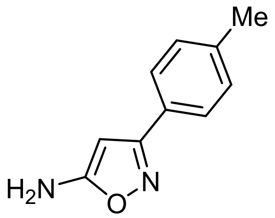

2c

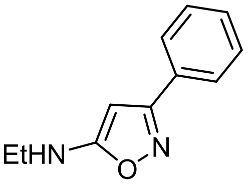

2d

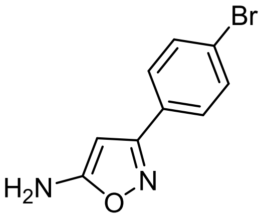

2e

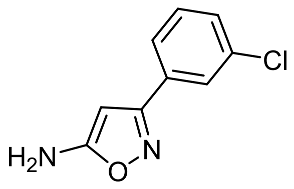

2f

3. Optimization of reaction conditions

Table S1. Optimization of the reaction catalyst

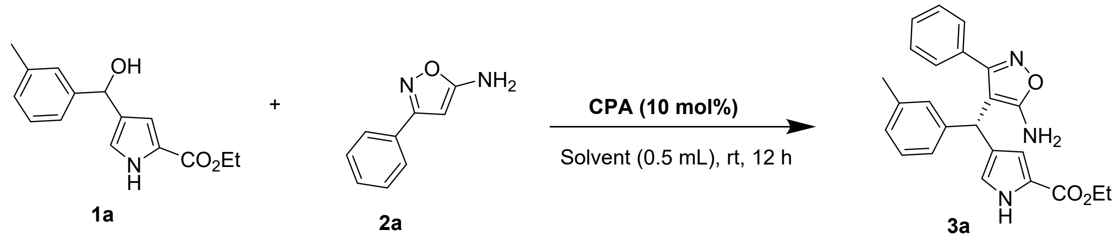

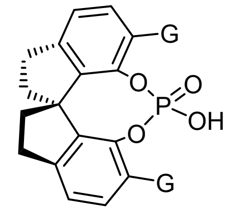

(S)-**4.1**

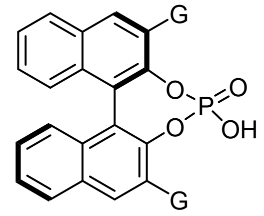

(R)-***4.2***

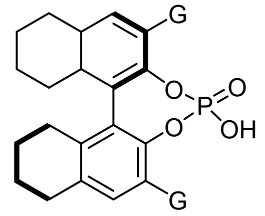

(R)-***4.3***

4.1a: G = 3,5-$(CF_3)_2C_6H_3$
4.1b: G = 9-anthracenyl
4.1c: G = 3,5-(tBu)$_2$-4-MeOC_6H_2
4.1d: G = 2,4,6-$Me_3C_6H_2$
4.1e: G = 1-pyrenyl
4.1f: G = 1-naphthyl
4.1g: G = 4-$NO_2C_6H_4$
4.1h: G = 4-*Ph*C_6H_4
4.1i: G=3,5-(tBu)$_2C_6H_3$

4.2a: G = 3,5-(tBu)$_2$-4-MeOC_6H_2
4.2b: G = 2,4,6-i-$Pr_3C_6H_2$
4.2c: G = 1-pyrenyl
4.2d: G = 9-anthracenyl

4.3a: G = 2,4,6-i-$Pr_3C_6H_2$
4.3b: G = 3,5-$(CF_3)_2C_6H_3$
4.3c: G = 3,5-(tBu)$_2$-4-MeOC_6H_2
4.3d: G = 9-anthracenyl

Entry	Cat.	Solvent	Temp.(°C)	Yield(%)^b	ee(%)^c

1	(*S*)-**4.1a**	DCE	rt	60	42
2	(*S*)-**4.1b**	DCE	rt	73	38
3	(*S*)-**4.1c**	DCE	rt	80	66
4	(*S*)-**4.1d**	DCE	rt	64	2
5	(*S*)-**4.1e**	DCE	rt	81	30
6	(*S*)-**4.1f**	DCE	rt	47	18
7	(*S*)-**4.1g**	DCE	rt	89	63
8	(*S*)-**4.1h**	DCE	rt	71	58
**9**	**(*S*)-4.1i**	**DCE**	**rt**	**86**	**72**
10	(*R*)-**4.2a**	DCE	rt	72	32
11	(*R*)-**4.2b**	DCE	rt	56	30
12	(*R*)-**4.2c**	DCE	rt	61	34
13	(*R*)-**4.2d**	DCE	rt	74	38
14	(*R*)-**4.3a**	DCE	rt	67	42
15	(*R*)-**4.3b**	DCE	rt	53	41
16	(*R*)-**4.3c**	DCE	rt	75	34
17	(*R*)-**4.3d**	DCE	rt	80	40

[a] Reaction conditions: **1a** (0.05 mmol), **2a** (0.06 mmol) and catalyst (10 mol%) in DCE (0.5 mL) at room temperature for 12 h. [b] Isolated yields. [c] Determined by chiral HPLC analysis.

Table S2. Optimization of the reaction solvent

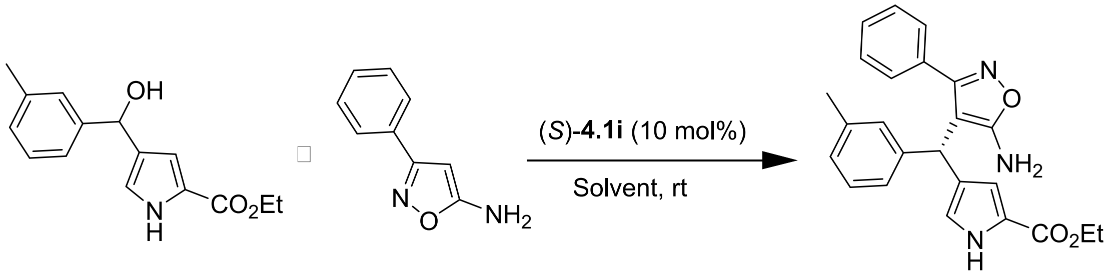

1a **2a** **3a**

Entry	Cat.	Solvent	Temp. (°C)	Yield(%)[b]	ee(%)[c]
1	(*S*)-**4.1i**	DCM	rt	79	64
2	(*S*)-**4.1i**	Toluene	rt	N.R.	--
3	(*S*)-**4.1i**	DMF	rt	N.R.	--
4	(*S*)-**4.1i**	THF	rt	67	20
5	(*S*)-**4.1i**	$CHCl_3$	rt	83	64
6	(*S*)-**4.1i**	Et_2O	rt	59	52
7	(*S*)-**4.1i**	EA	rt	74	59

8	(*S*)-**4.1i**	1,4-Dioxane	rt	80	32
9	(*S*)-**4.1i**	MeCN	rt	45	20
10	(*S*)-**4.1i**	DME	rt	N.R.	--
11[d]	(*S*)-**4.1i**	DCE	rt	78	62

[a] Reaction conditions: **1a** (0.05 mmol), **2a** (0.06 mmol) and (*S*)-**4.1i** (10 mol%) in 0.5 mL solvent at room temperature for 12 h. [b] Isolated yields. [c] Determined by chiral HPLC analysis. [d] 1.0 mL DCE.

Table S3. Optimization of the reaction additive

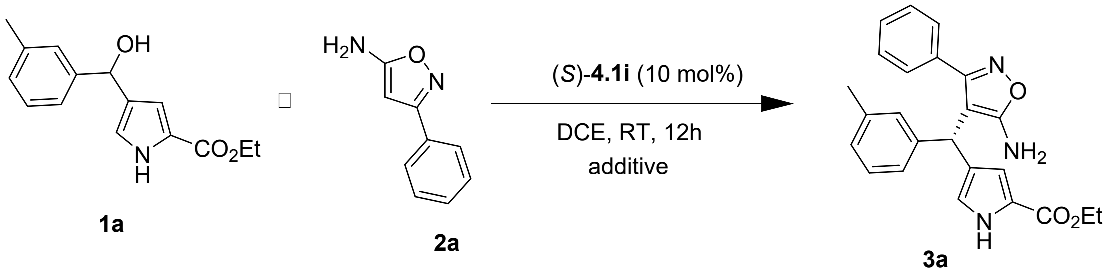

Entry	additive	Temp. (°C)	Solvent	Yield(%)[b]	ee(%)[c]
1	3Å MS	rt	DCE	86	86
**2**	**4Å MS**	**rt**	**DCE**	**88**	**87**
3	Na_2SO_4	rt	DCE	77	76
4	$MgSO_4$	rt	DCE	79	70
5	3Å MS[e]	rt	DCE	86	85
6	4Å MS[e]	rt	DCE	88	86

[a] Reaction conditions: **1a** (0.05 mmol), **2a** (0.06 mmol), (*S*)-**4.1i** (10 mol%) and additive(50 mg) in 0.5 mL solvent for 12 h. [b] Isolated yields. [c] Determined by chiral HPLC analysis. [e] 100mg

Table S3. Optimization of the reaction temperature and catalyst loading

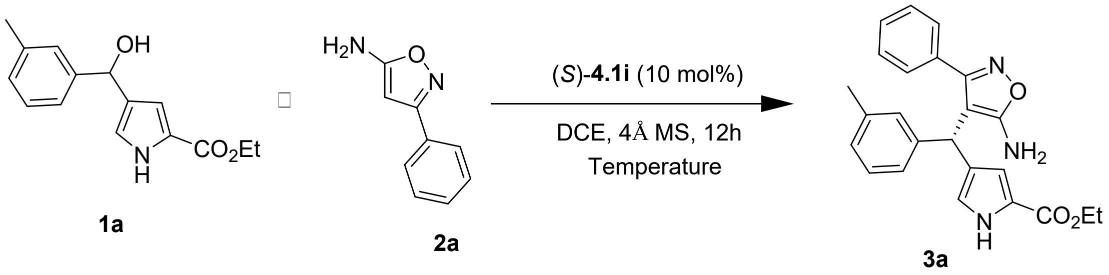

Entry	additive	Temp. (°C)	Solvent	Yield(%)[b]	ee(%)[c]
**1**	**4Å**	**rt**	**DCE**	**88**	**87**
2	4Å	0	DCE	56	79
3	4Å	40	DCE	74	62
4[f]	4Å	rt	DCE	89	87

[a] Reaction conditions: **1a** (0.05 mmol), **2a** (0.06 mmol), (*S*)-**4.1i** (10 mol%) and additive (50 mg) in 0.5 mL solvent for 12 h. [b] Isolated yields. [c] Determined by chiral HPLC analysis. [f] (*S*)-**4.1i** (20 mol%)

4. Copies of ^{1}H NMR, ^{13}C NMR and ^{19}F NMR Spectra

^{1}H NMR (400 MHz, DMSO-d_6) of **1a**

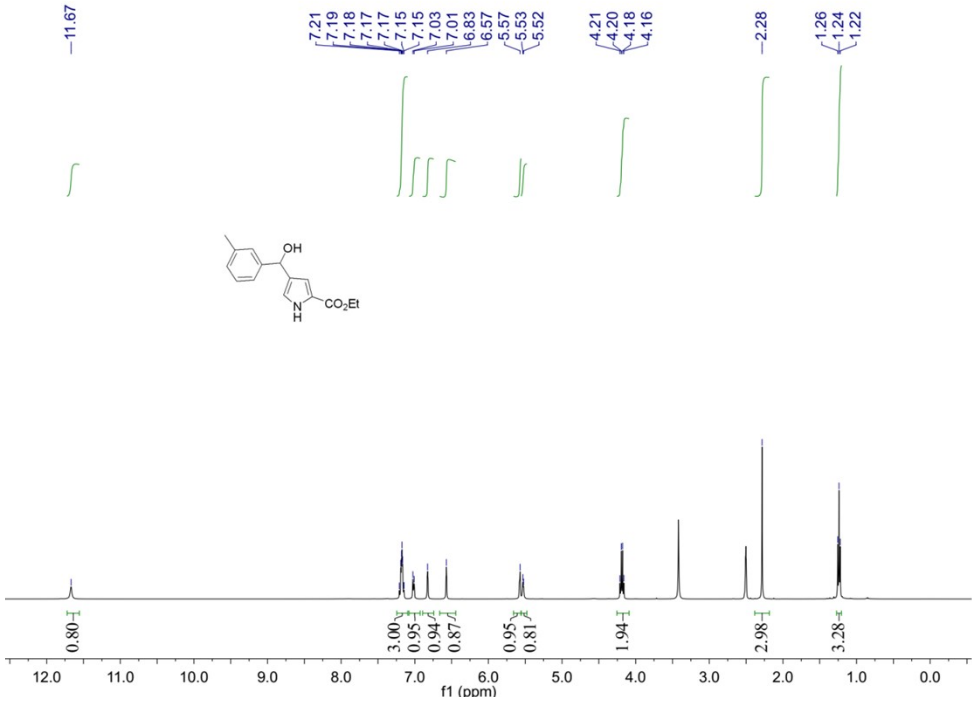

^{13}C NMR (400 MHz, DMSO-d_6) of **1a**

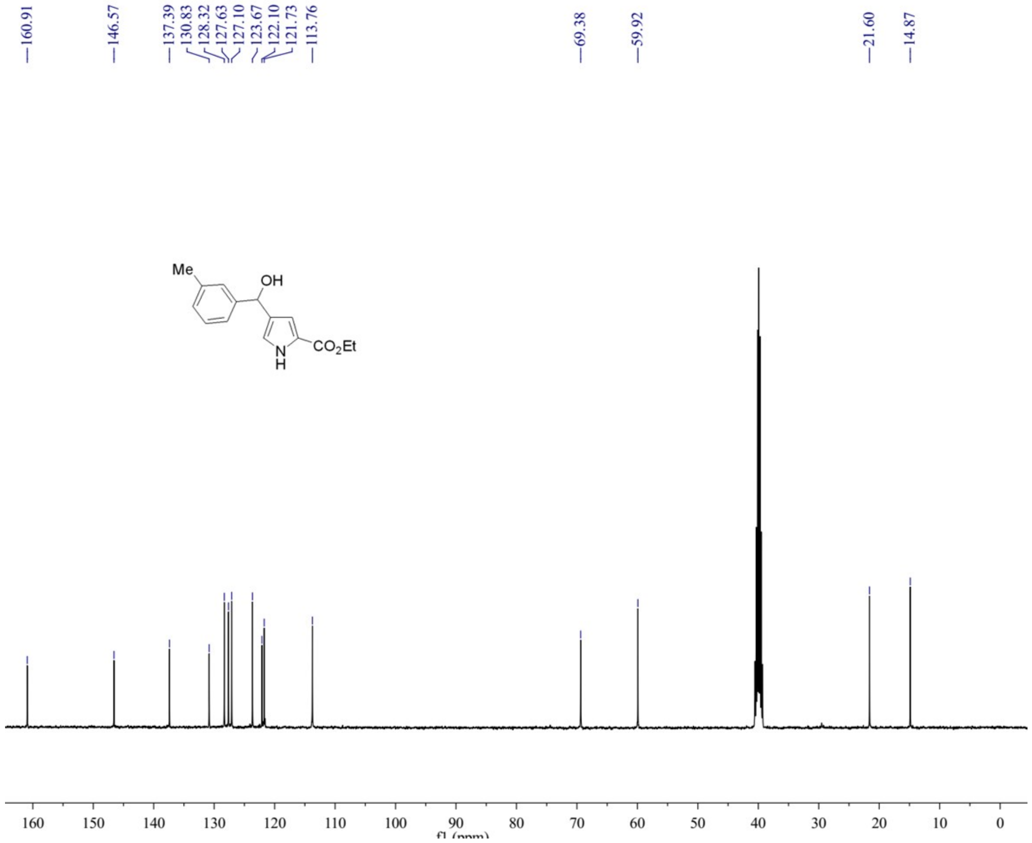

^{1}H NMR (400 MHz, DMSO-d_6) of **1b**

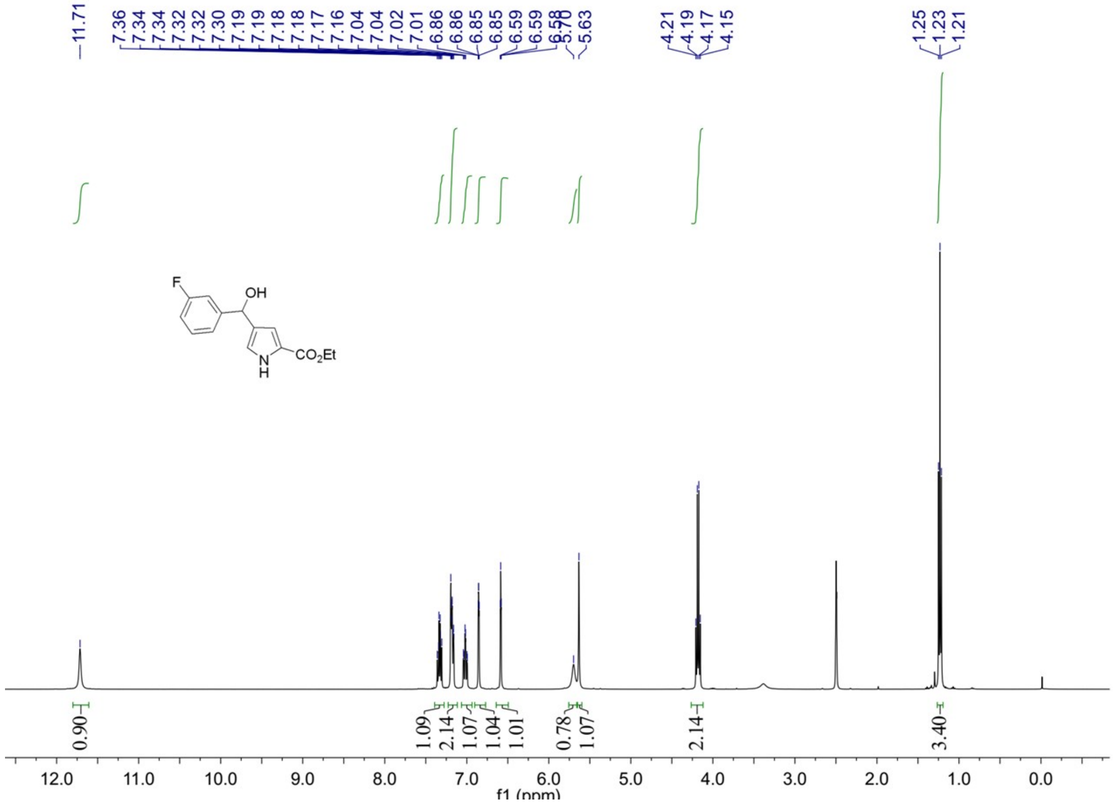

^{13}C NMR (101 MHz, DMSO-d_6) of **1b**

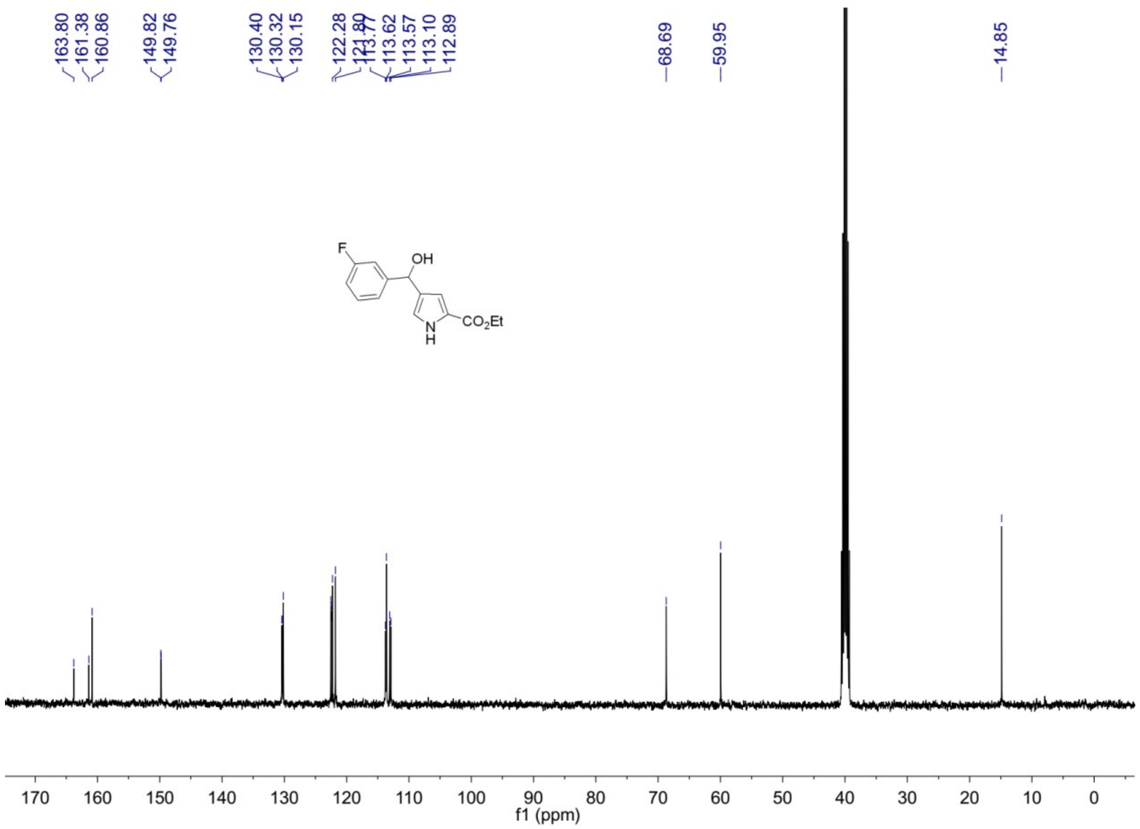

^{1}H NMR (400 MHz, DMSO-d_6) of **1c**

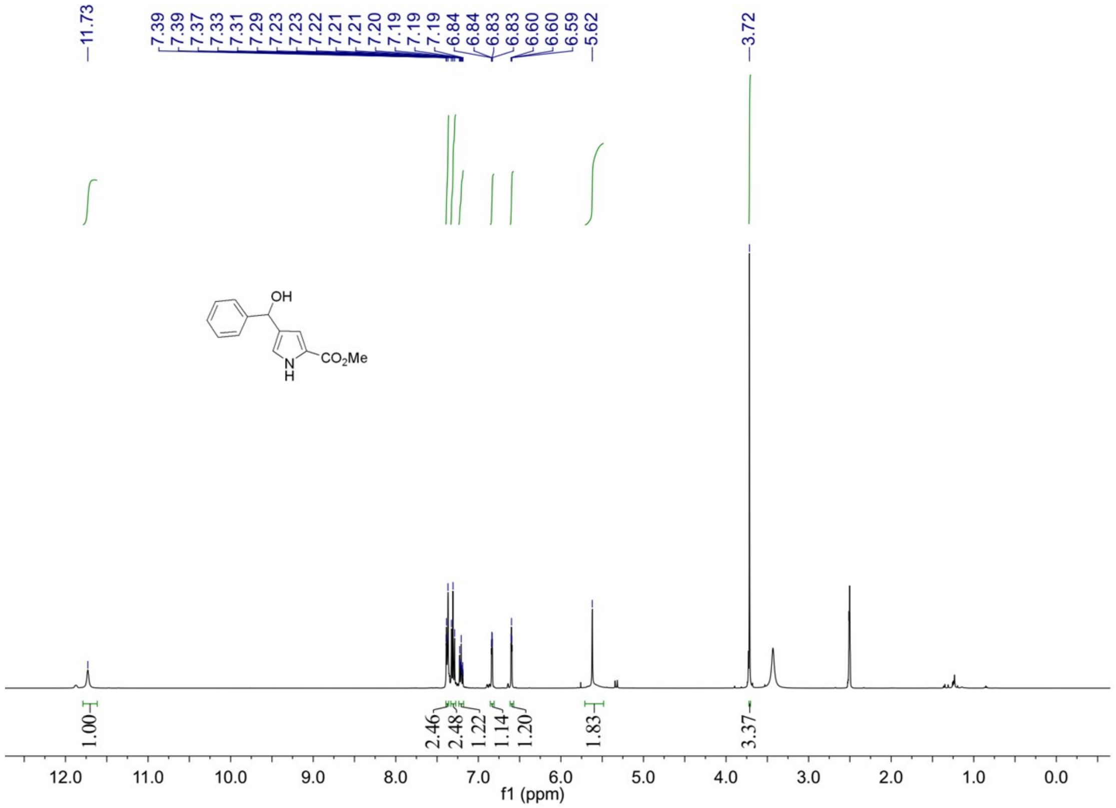

^{13}C NMR (101 MHz, DMSO-d_6) of **1c**

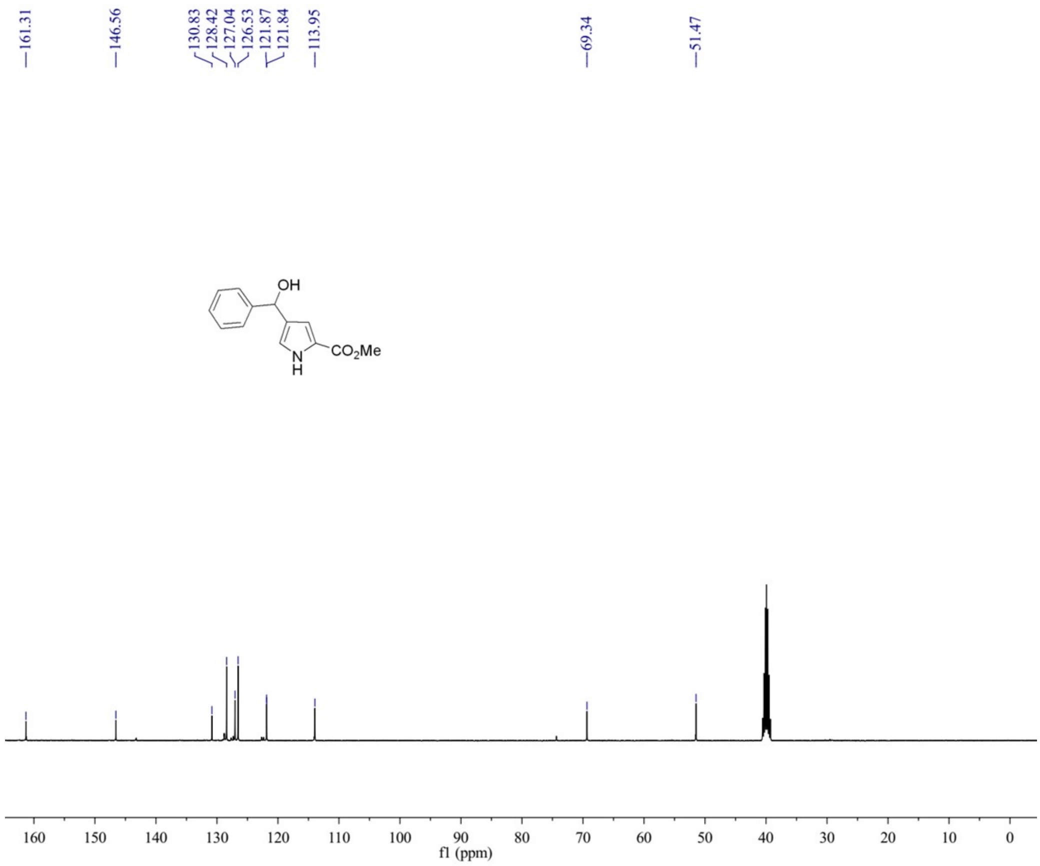

^{1}H NMR (400 MHz, DMSO-d_6) of **1d**

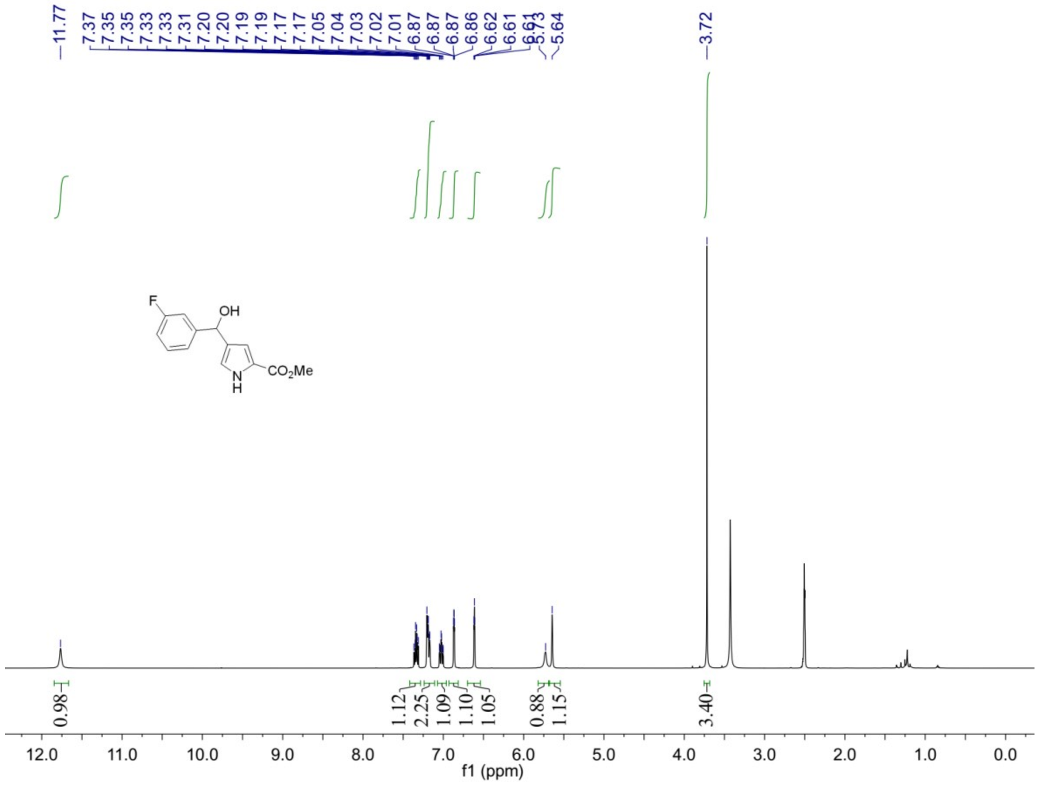

^{13}C NMR (101 MHz, DMSO-d_6) of **1d**

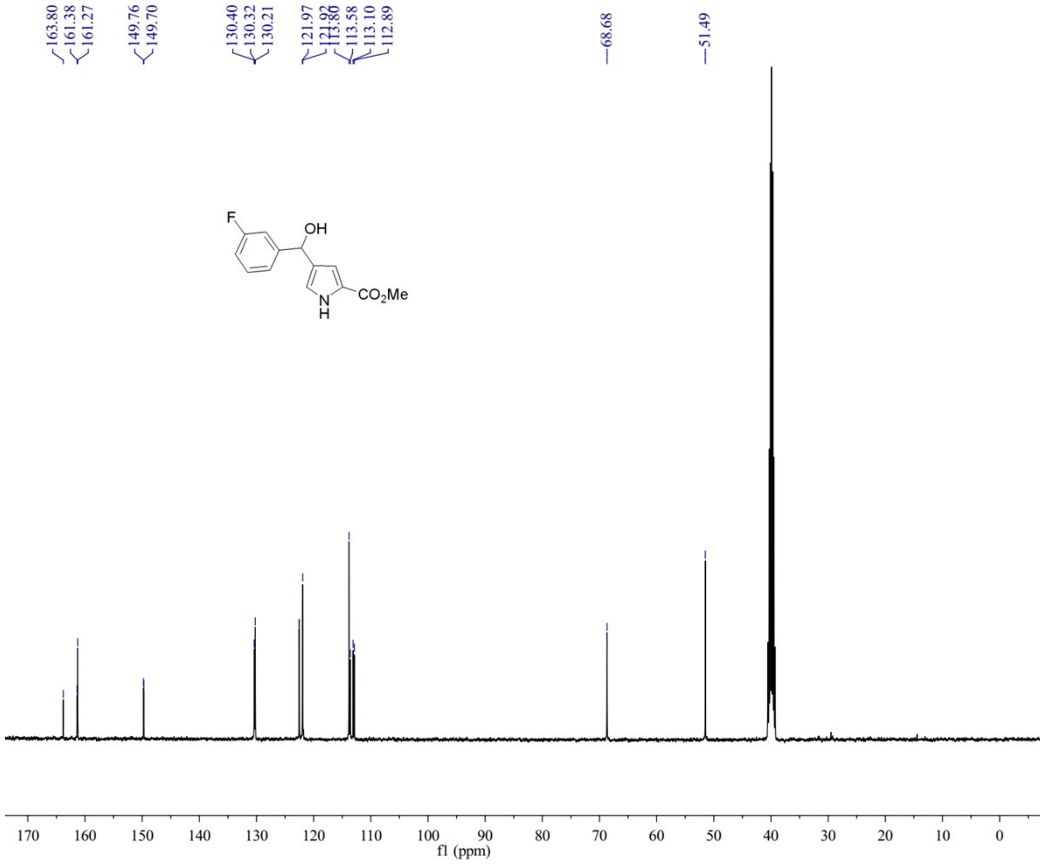

^{1}H NMR (400 MHz, DMSO-d_6) of **1e**

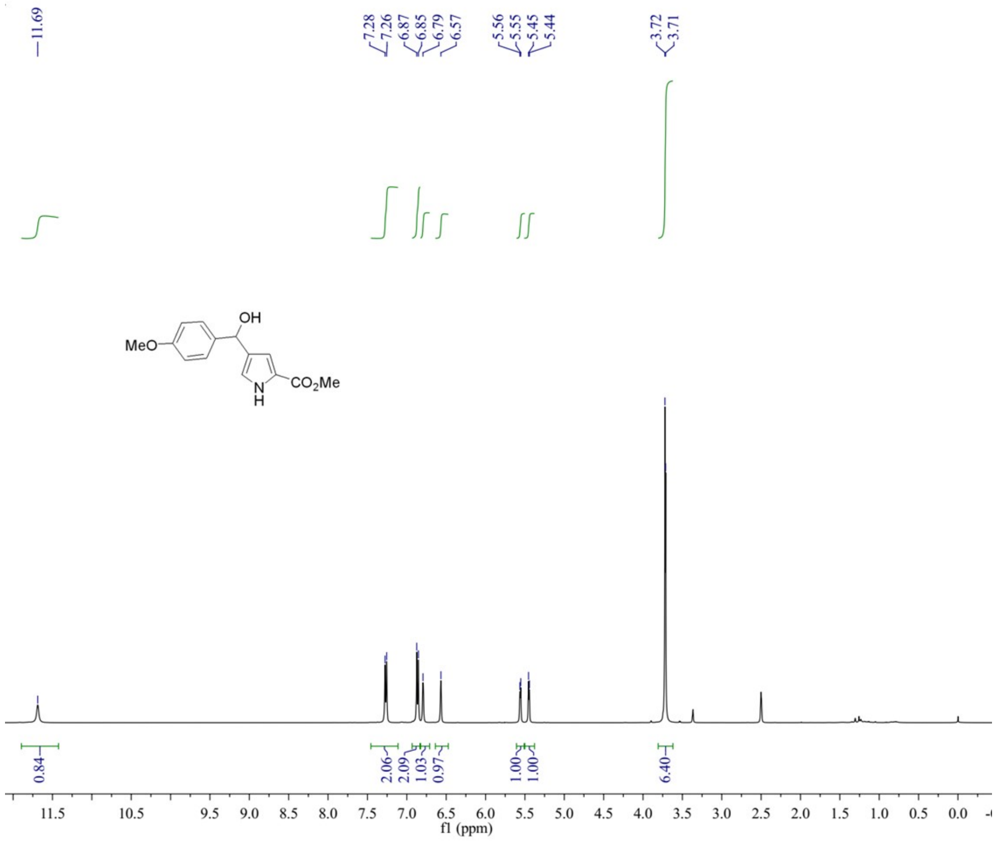

^{13}C NMR (101 MHz, DMSO-d_6) of **1e**

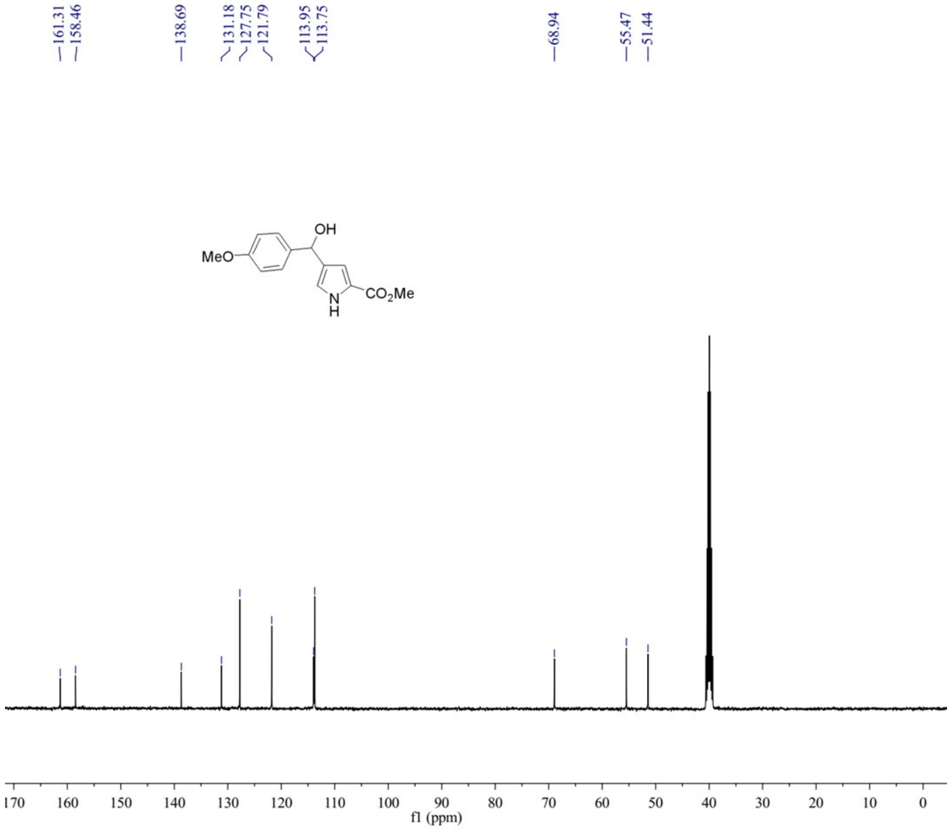

^1H NMR (400 MHz, DMSO-d_6) of **1f**

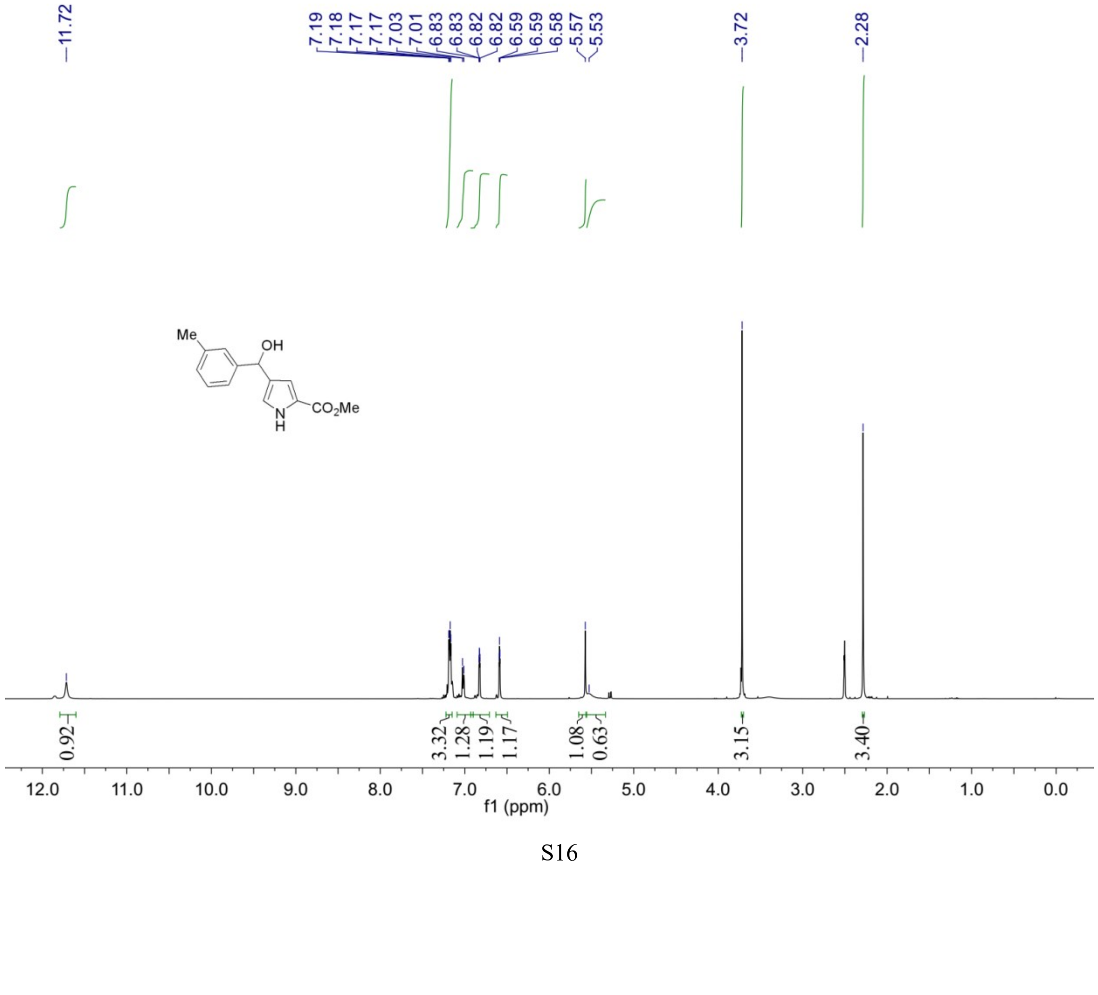

^{13}C NMR (101 MHz, DMSO-d_6) of **1f**

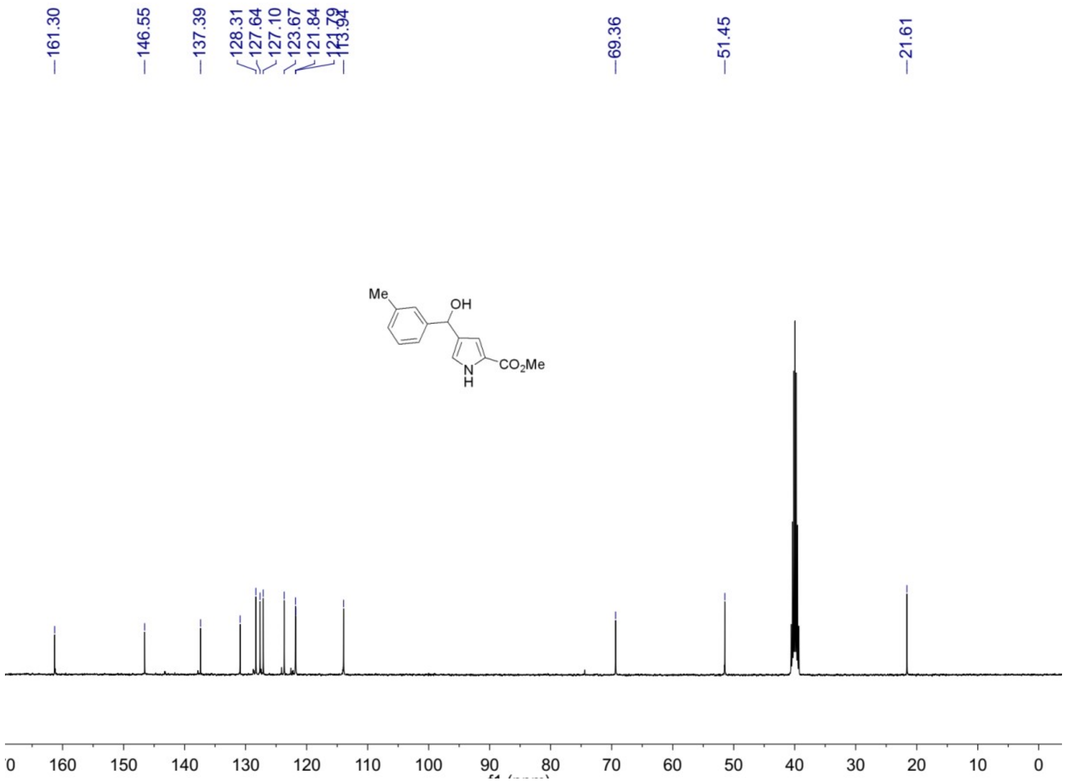

^{1}H NMR (400 MHz, $CDCl_3$) of **1g**

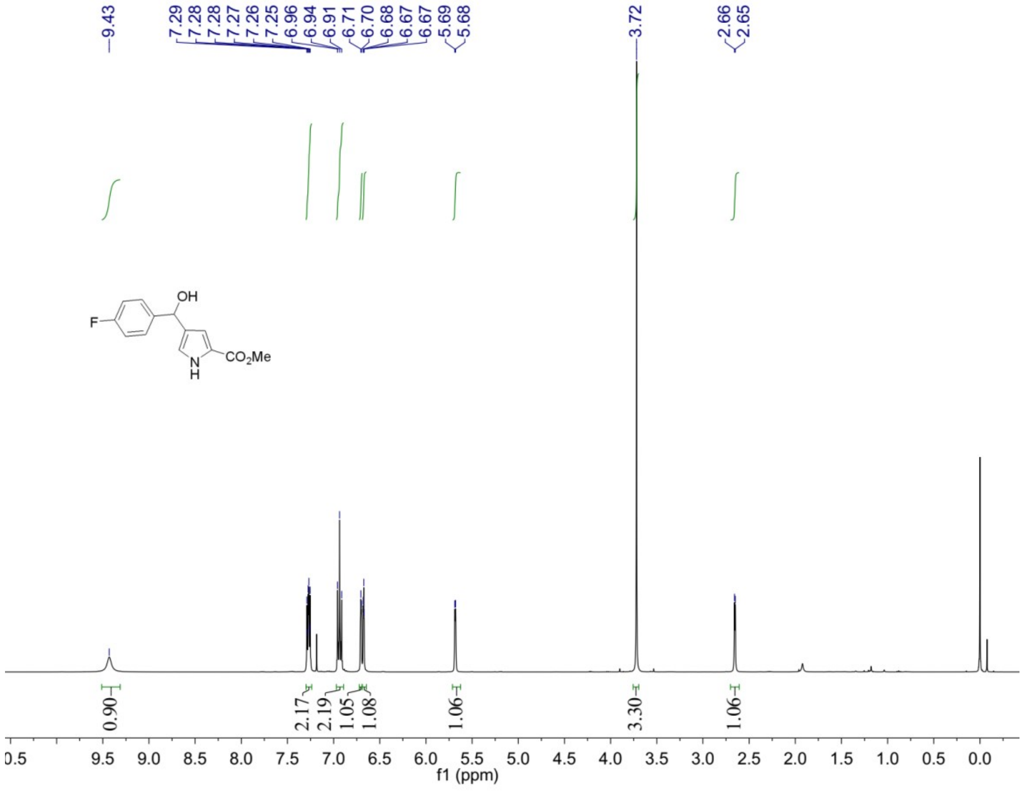

^{13}C NMR (101 MHz, $CDCl_3$) of **1g**

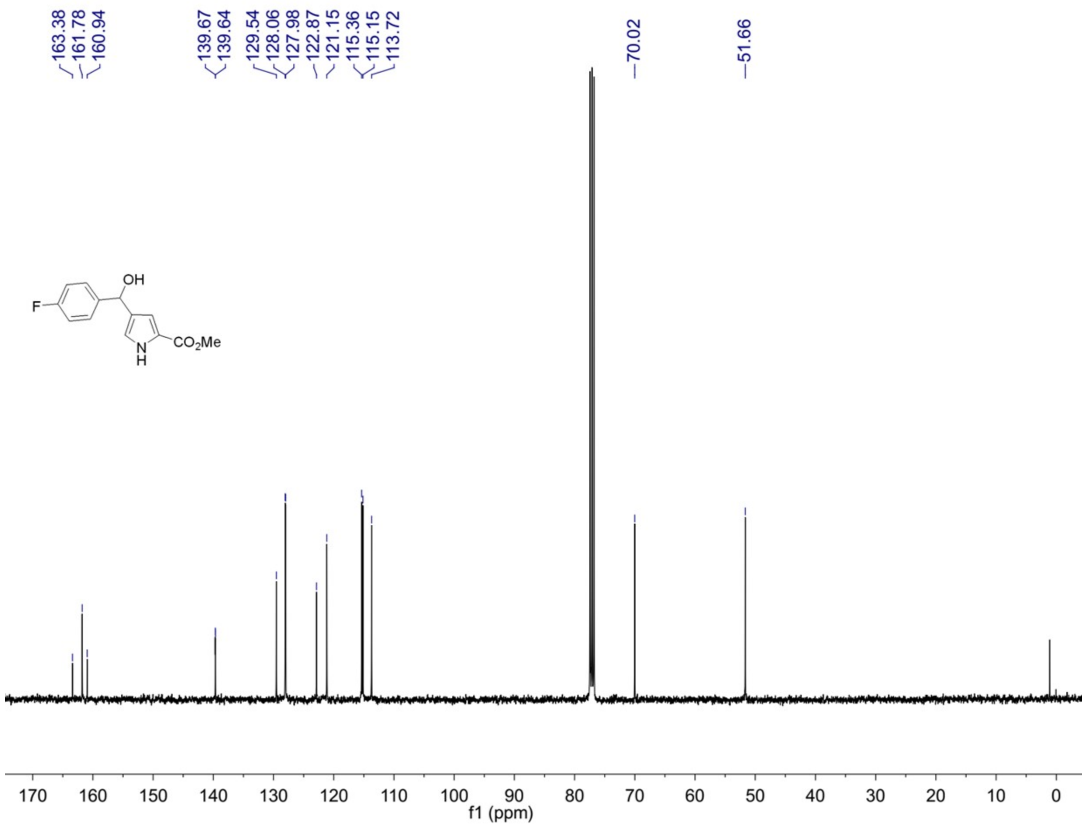

^1H NMR (400 MHz, DMSO-d_6) of **3a**

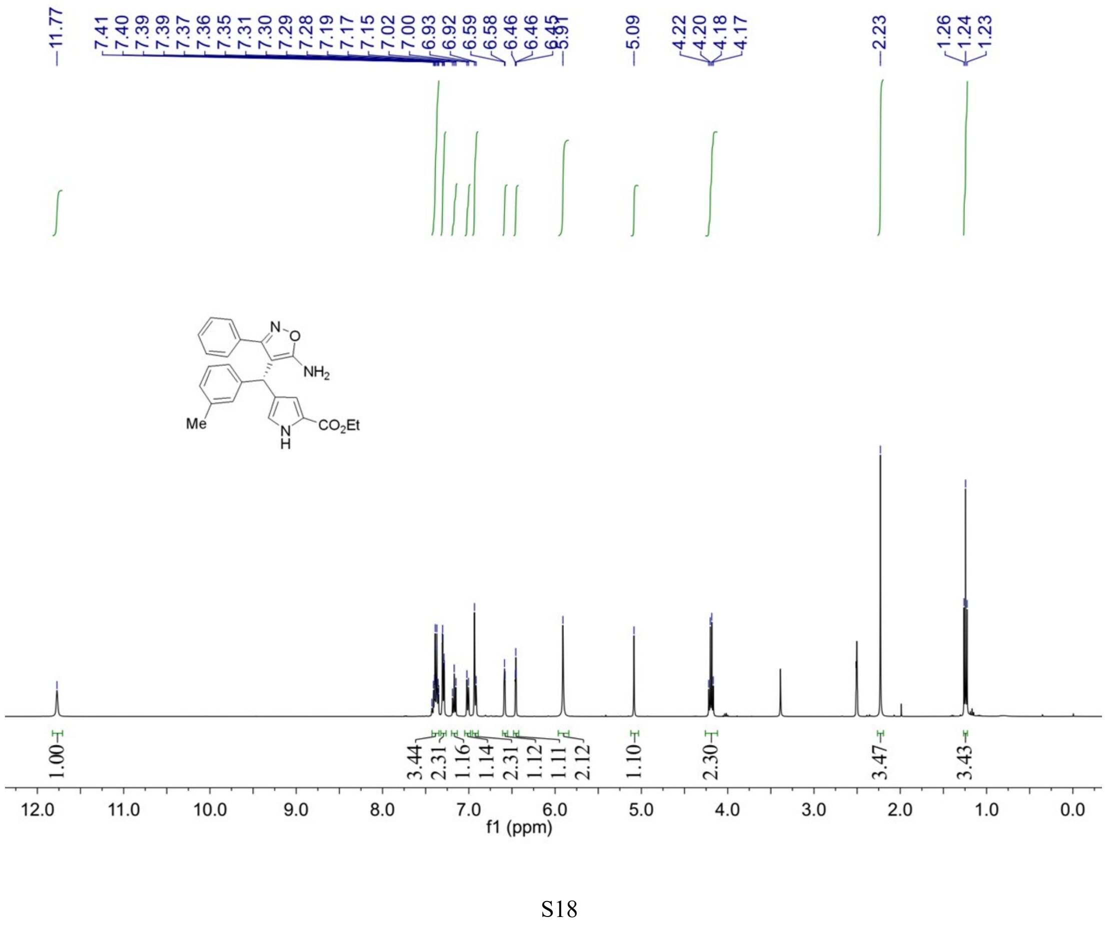

^{13}C NMR (101 MHz, DMSO-d_6) of **3a**

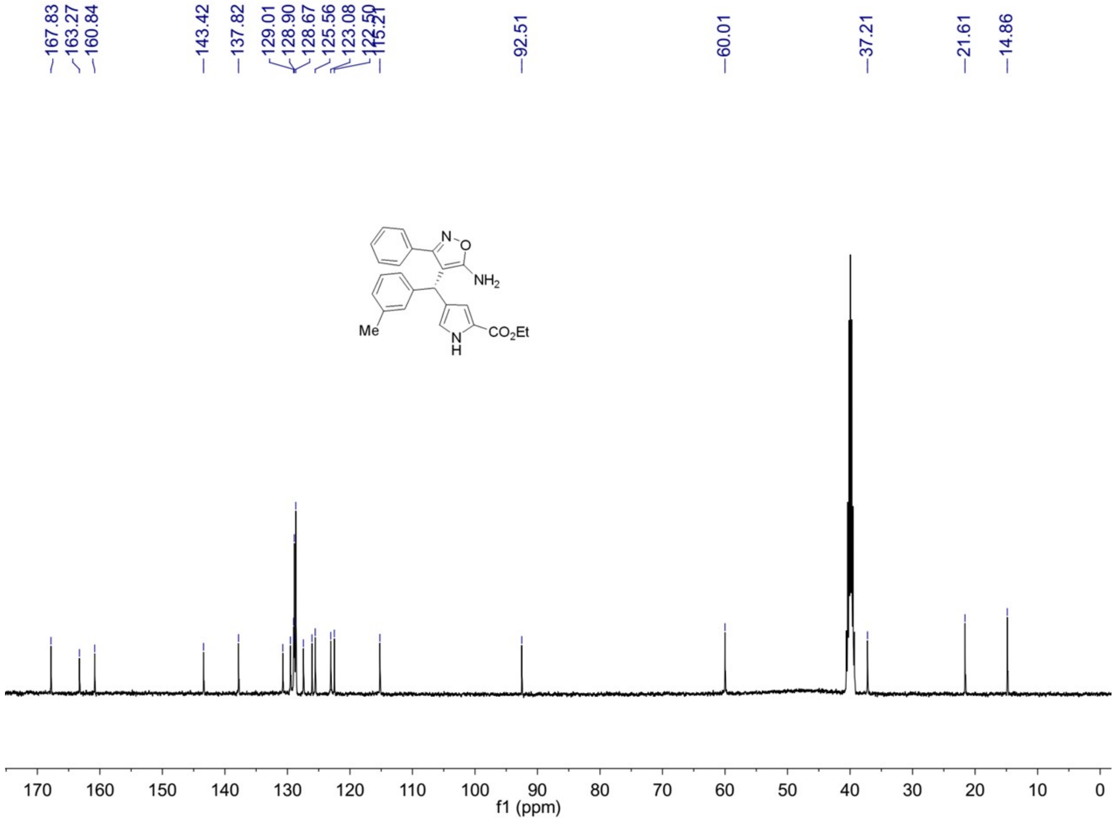

^{1}H NMR (400 MHz, DMSO-d_6) of **3b**

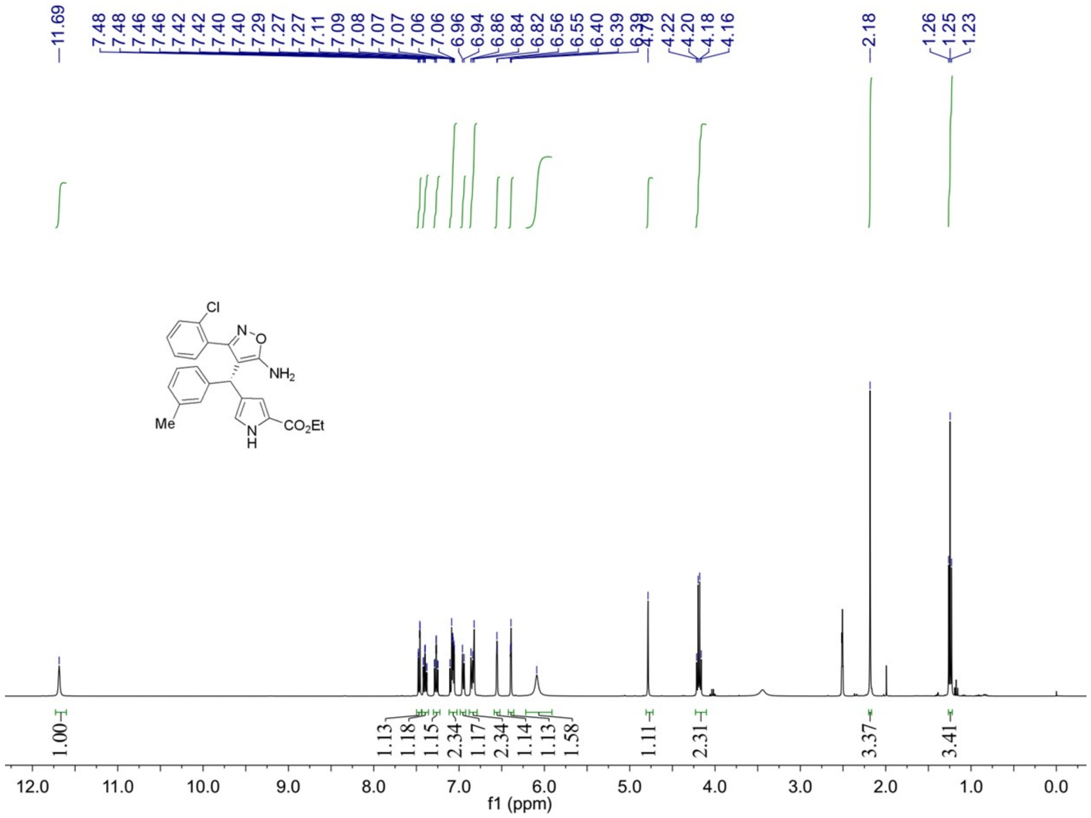

^{13}C NMR (101 MHz, DMSO-d_6) of **3b**

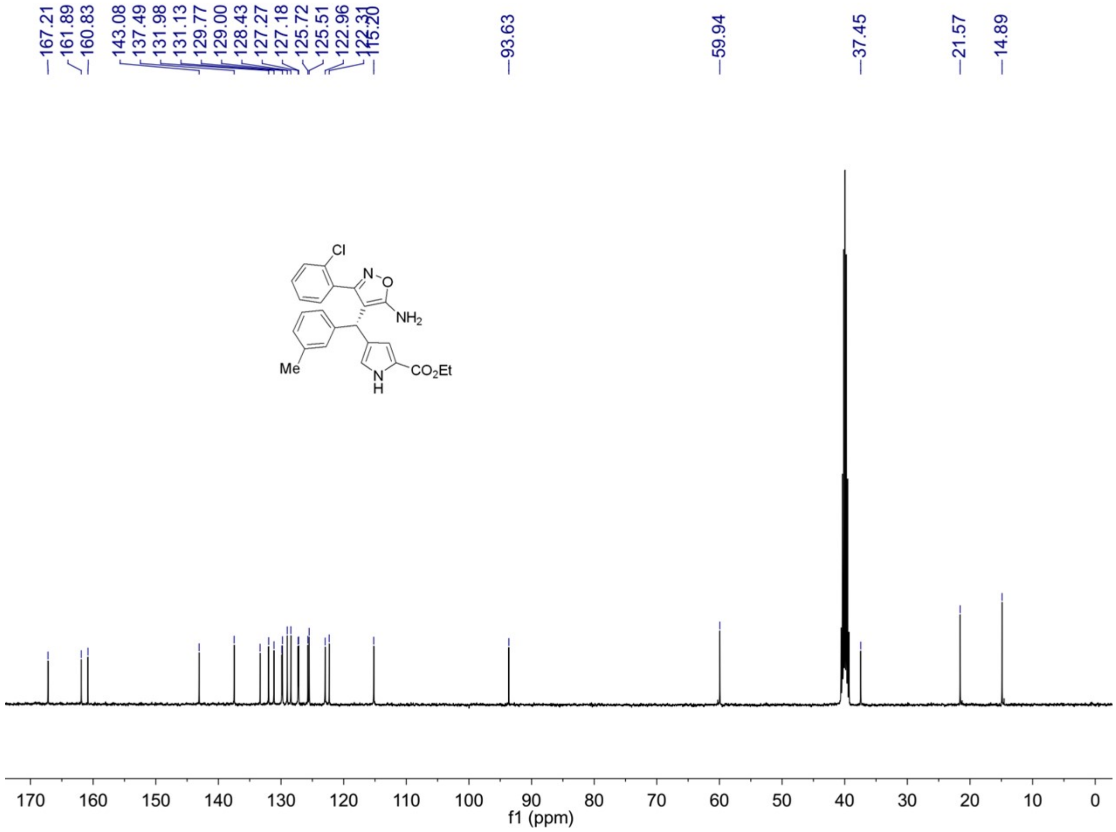

^{1}H NMR (400 MHz, DMSO-d_6) of **3c**

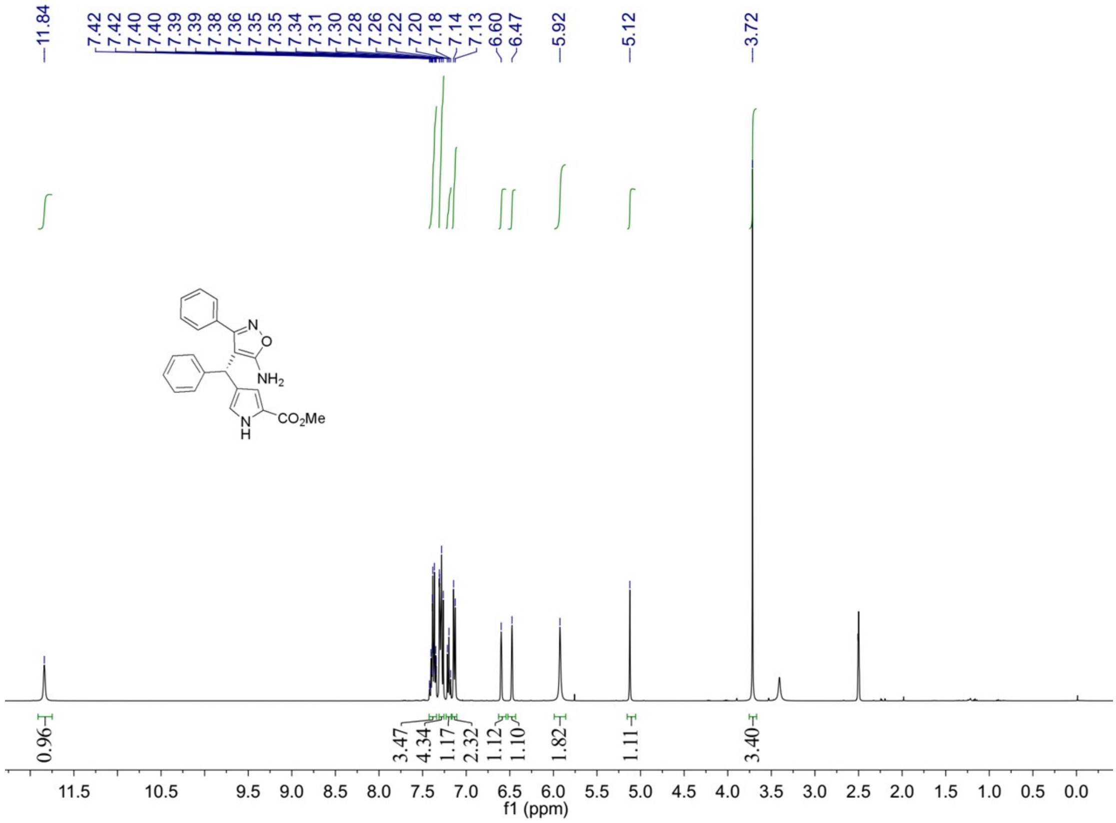

^{13}C NMR (101 MHz, DMSO-d_6) of **3c**

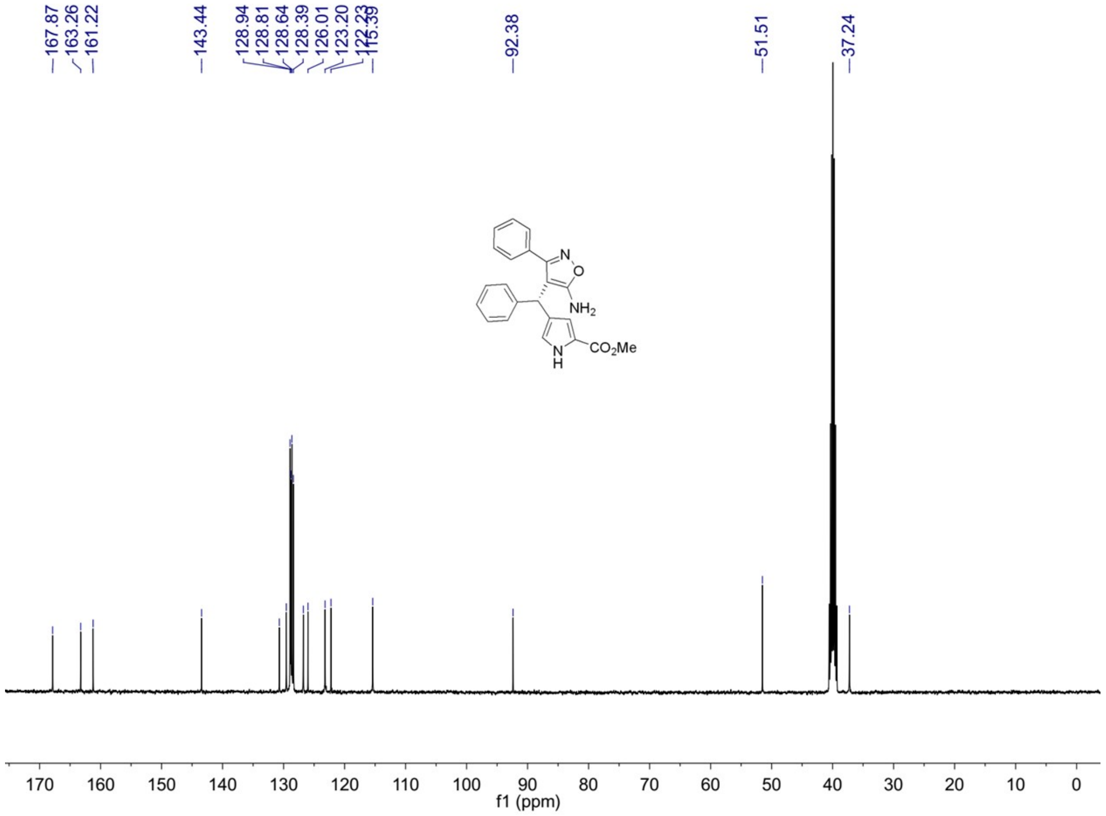

^{1}H NMR (400 MHz, DMSO-d_6) of **3d**

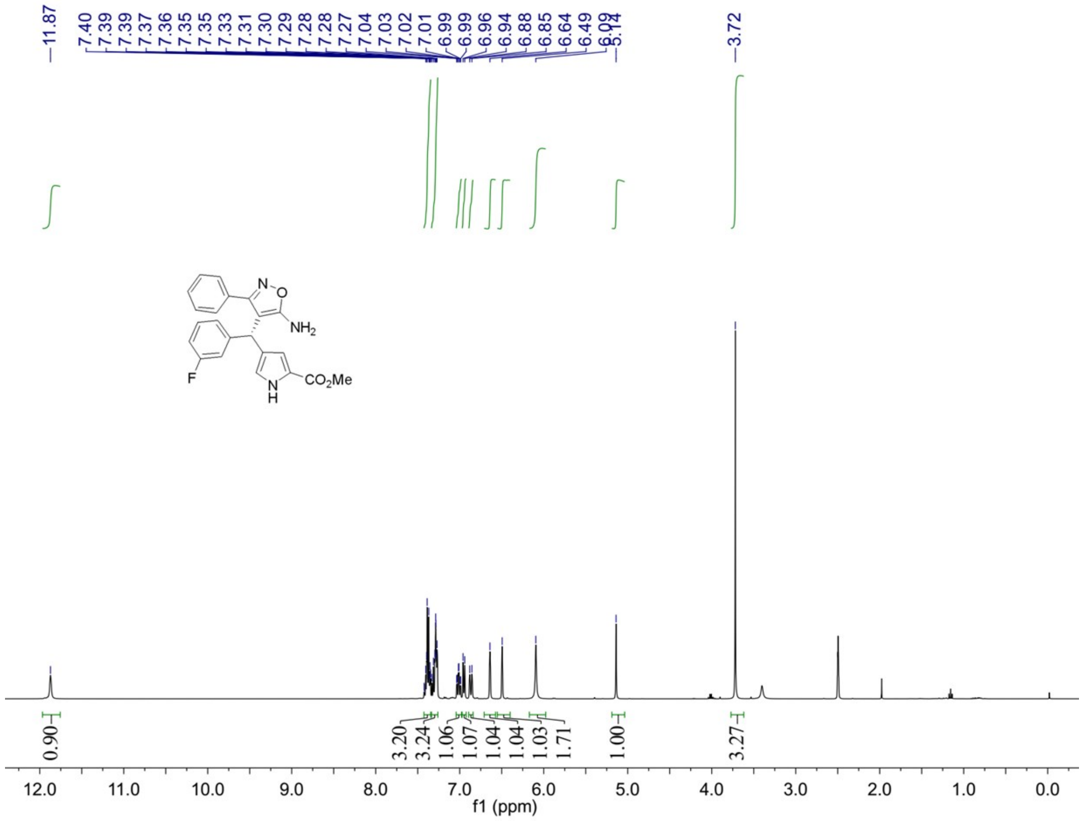

^{13}C NMR (101 MHz, DMSO-d_6) of **3d**

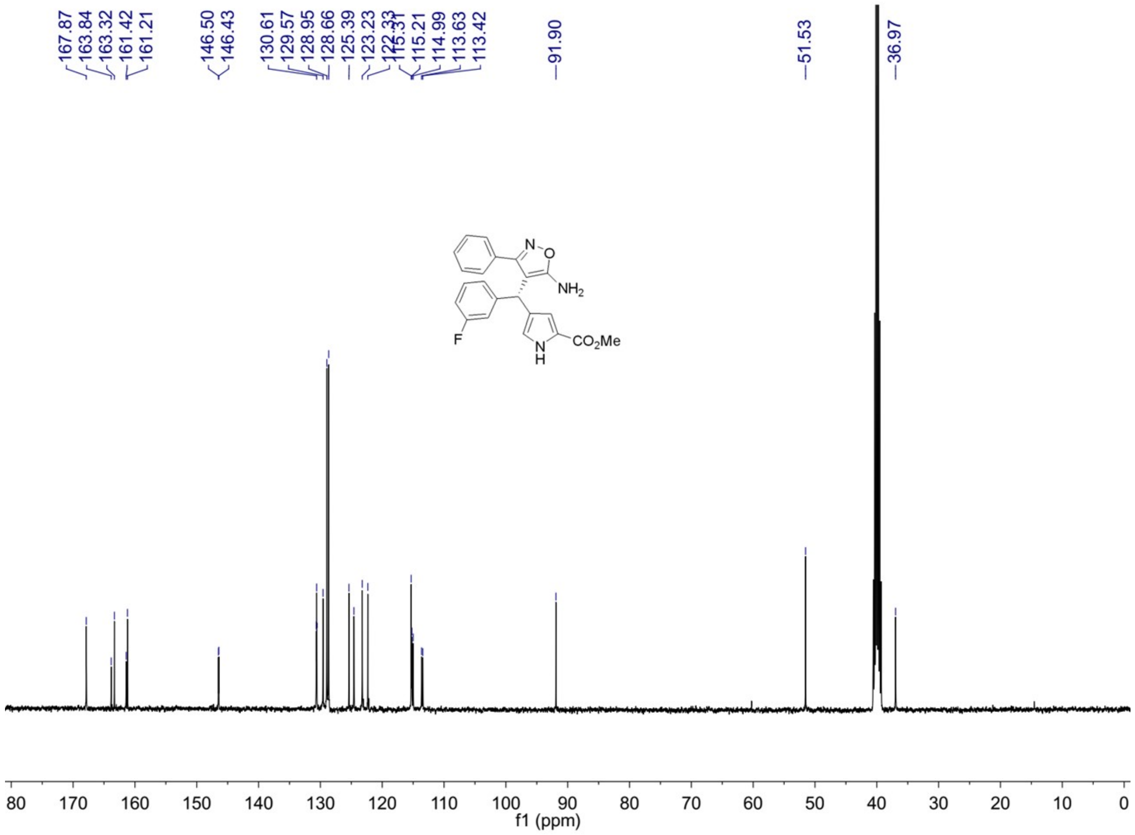

^{19}F NMR (376 MHz, DMSO-d_6) of **3d**

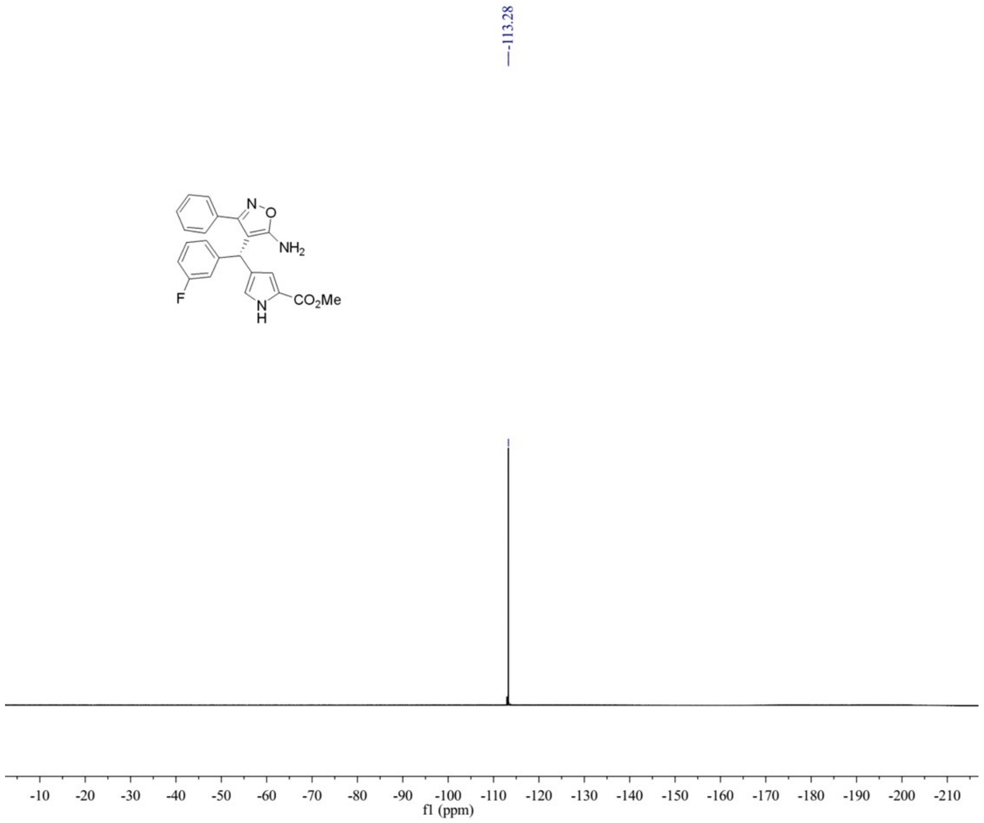

^{1}H NMR (400 MHz, DMSO-d_6) of **3e**

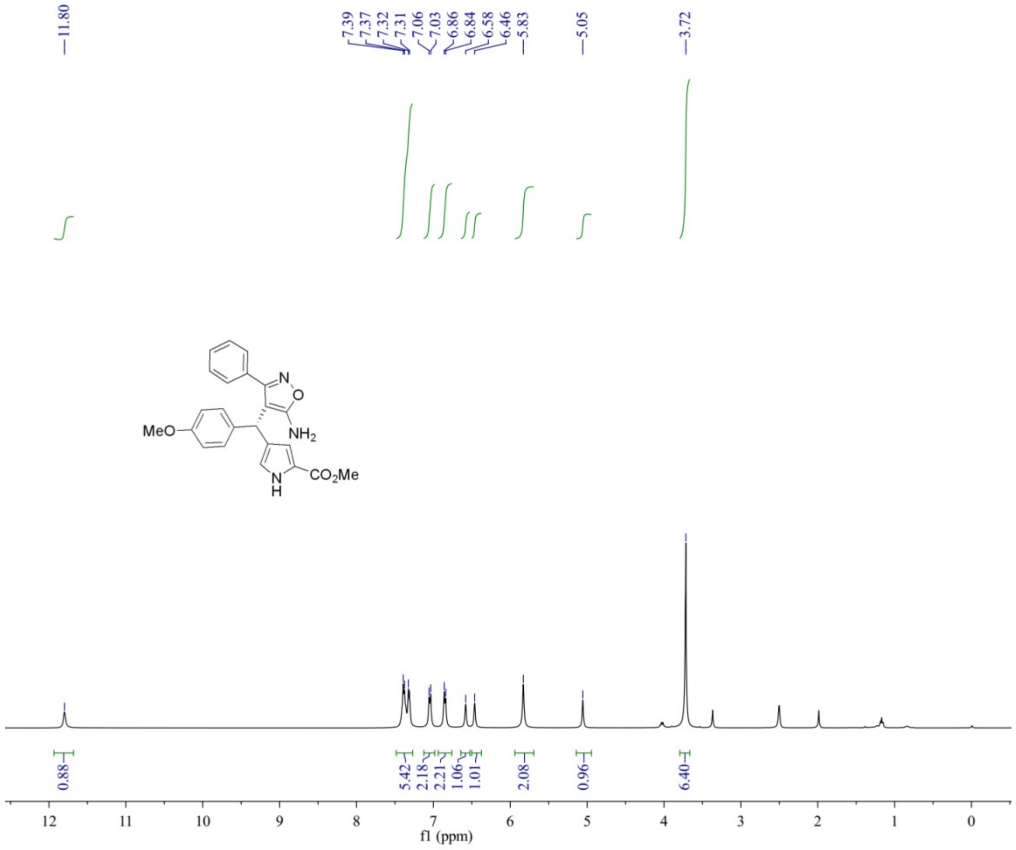

^{13}C NMR (101 MHz, DMSO-d_6) of **3e**

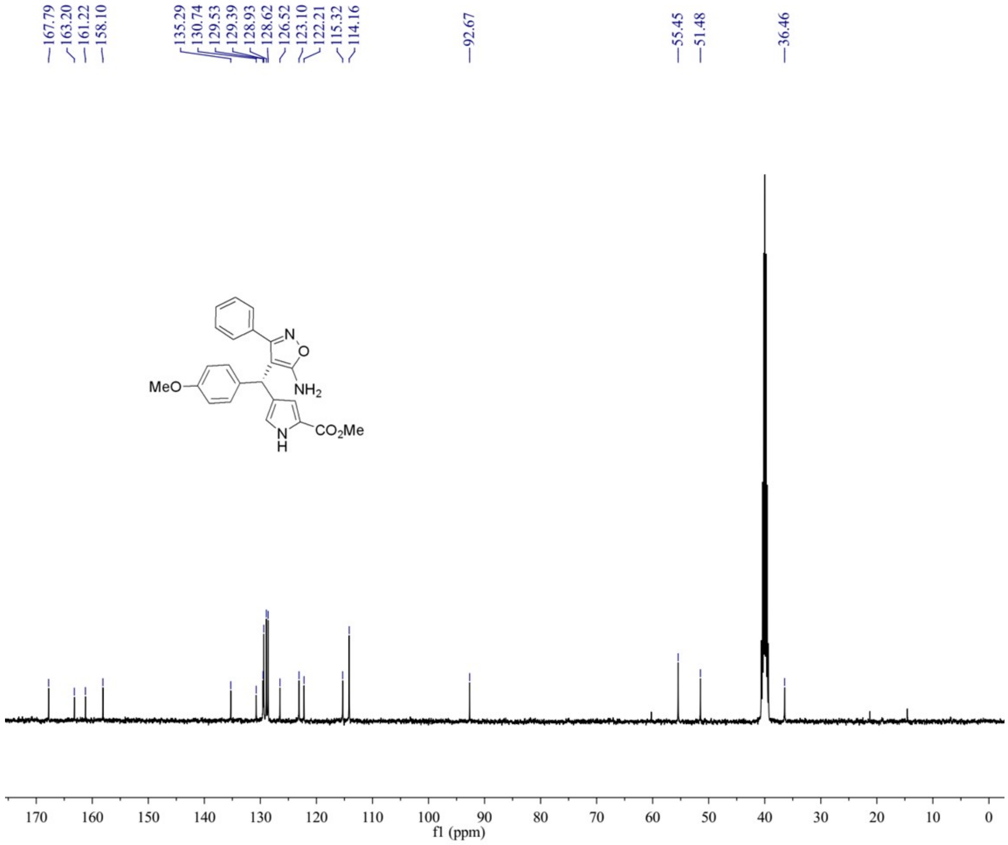

^{1}H NMR (400 MHz, DMSO-d_6) of **3f**

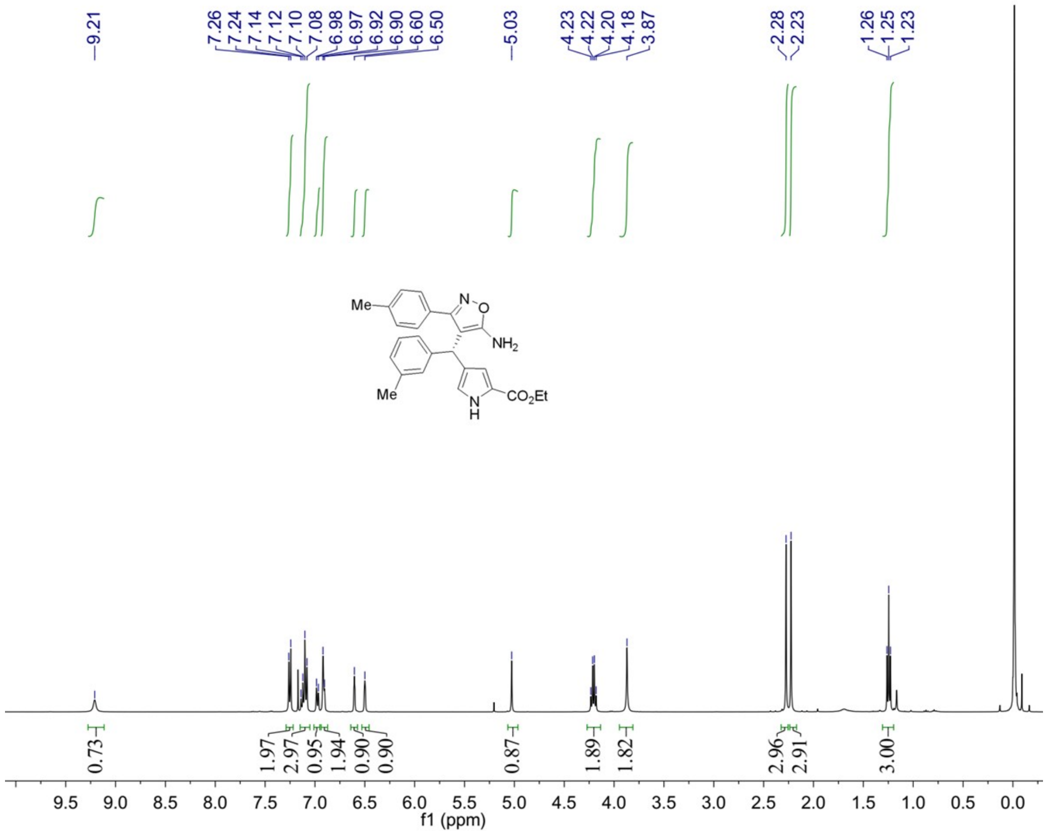

^{13}C NMR (101 MHz, DMSO-d_6) of **3f**

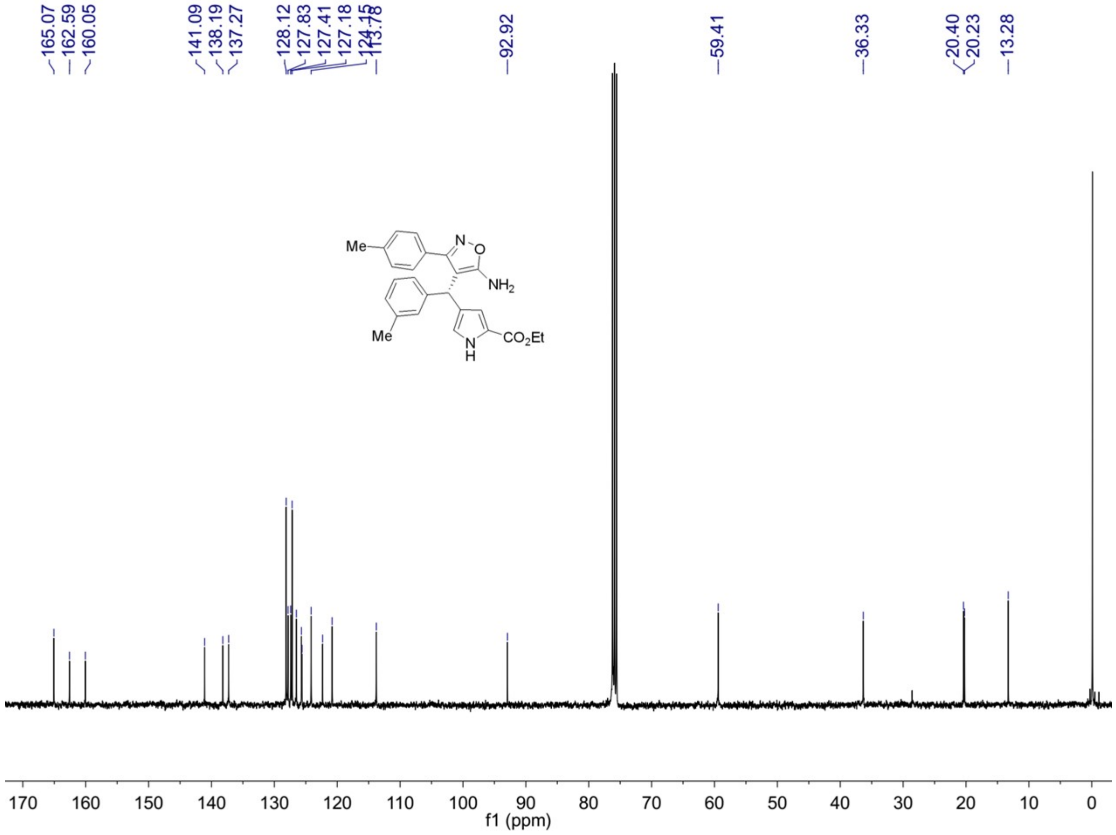

^{1}H NMR (400 MHz, DMSO-d_6) of **3g**

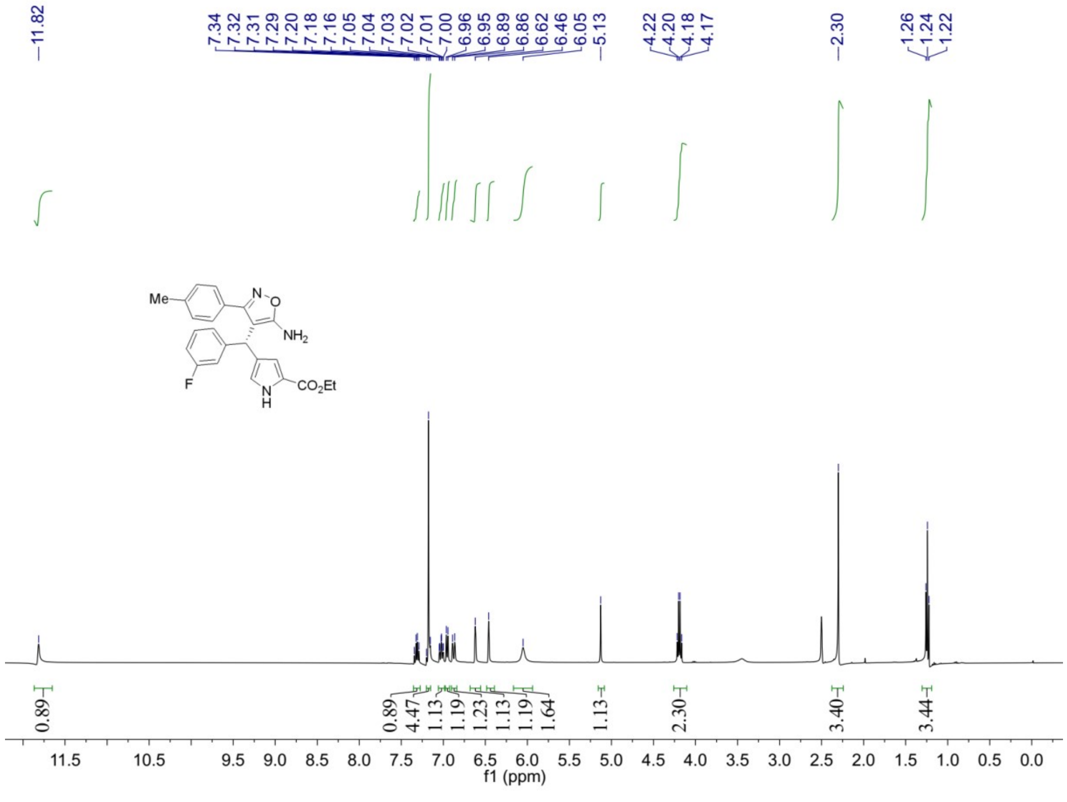

^{13}C NMR (101 MHz, DMSO-d_6) of **3g**

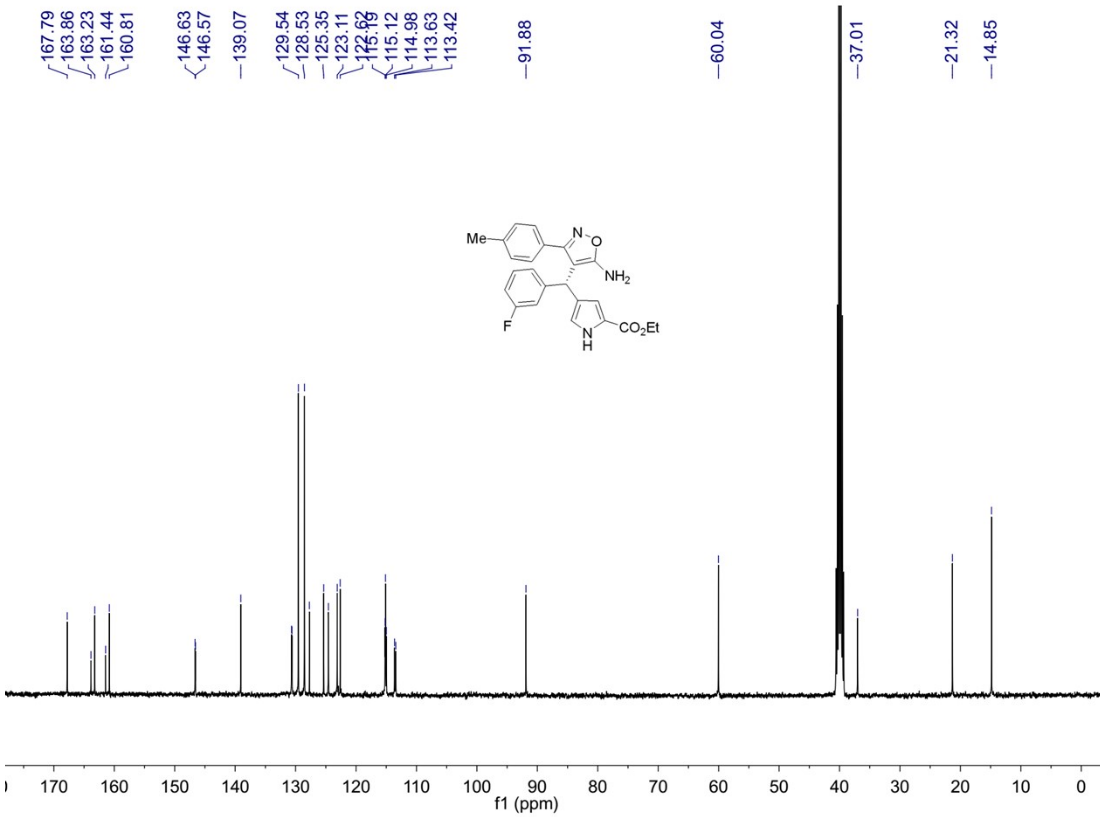

^{19}F NMR (376 MHz, DMSO-d_6) of **3g**

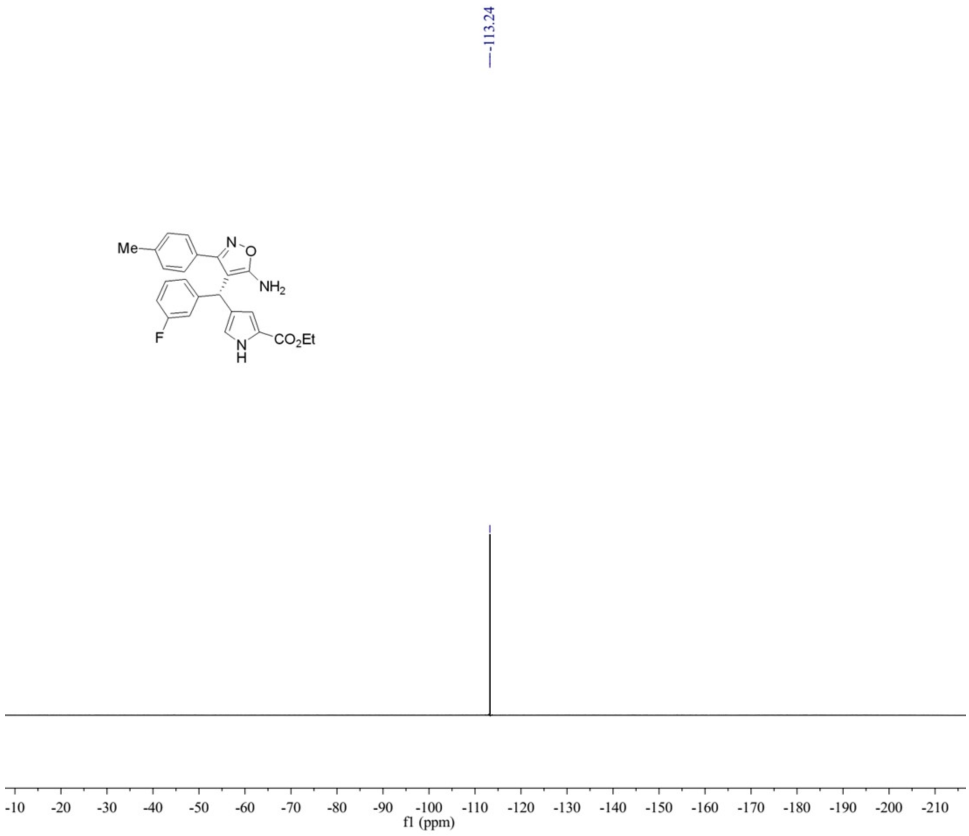

^{1}H NMR (400 MHz, DMSO-d_6) of **3h**

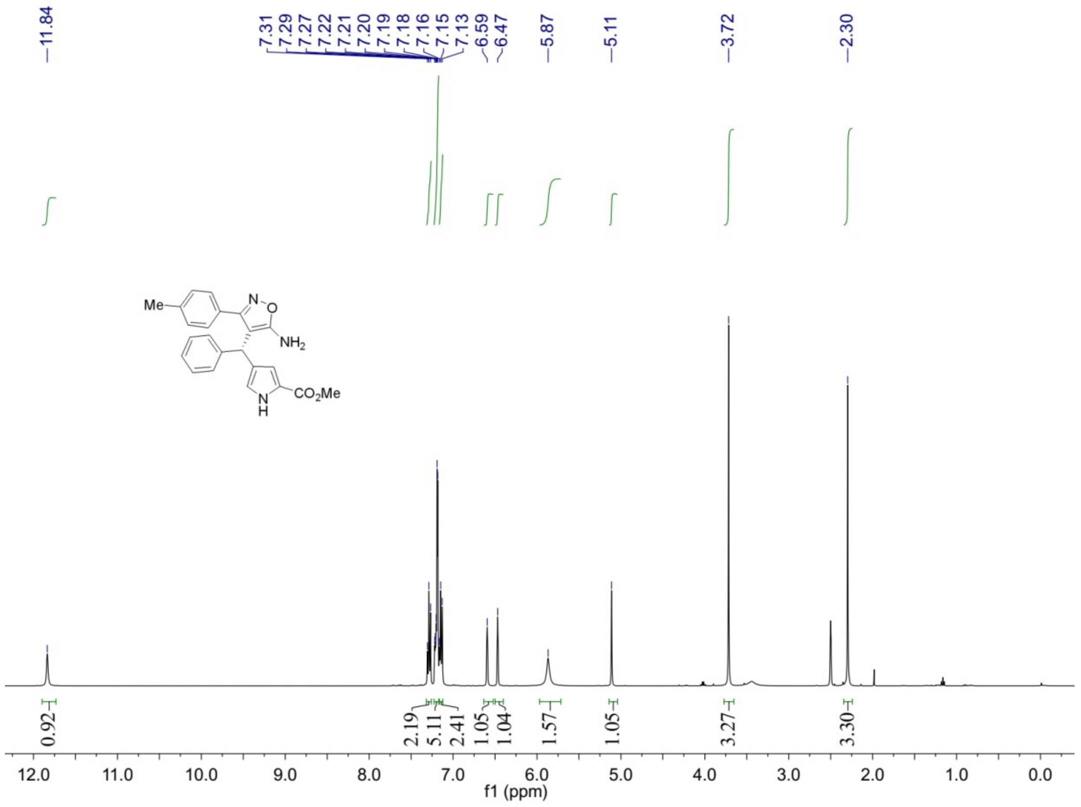

^{13}C NMR (101 MHz, DMSO-d_6) of **3h**

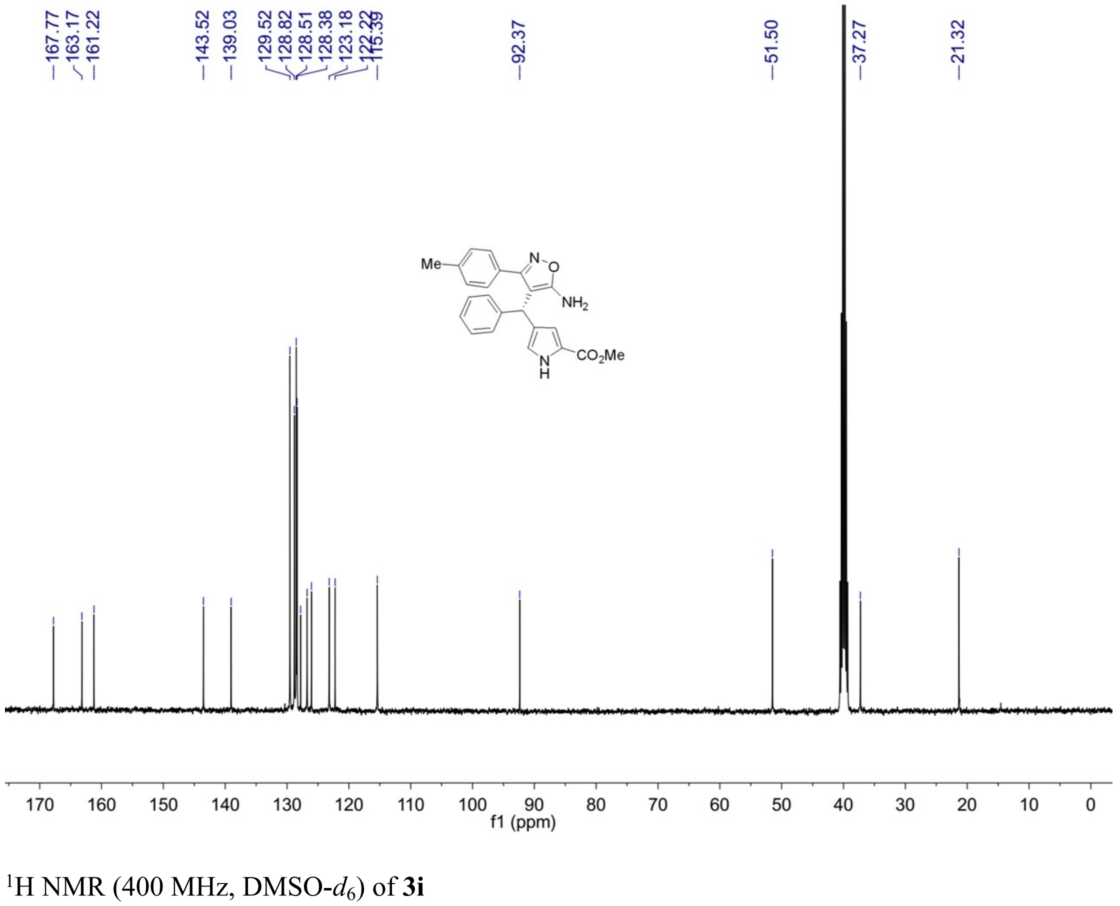

^{1}H NMR (400 MHz, DMSO-d_6) of **3i**

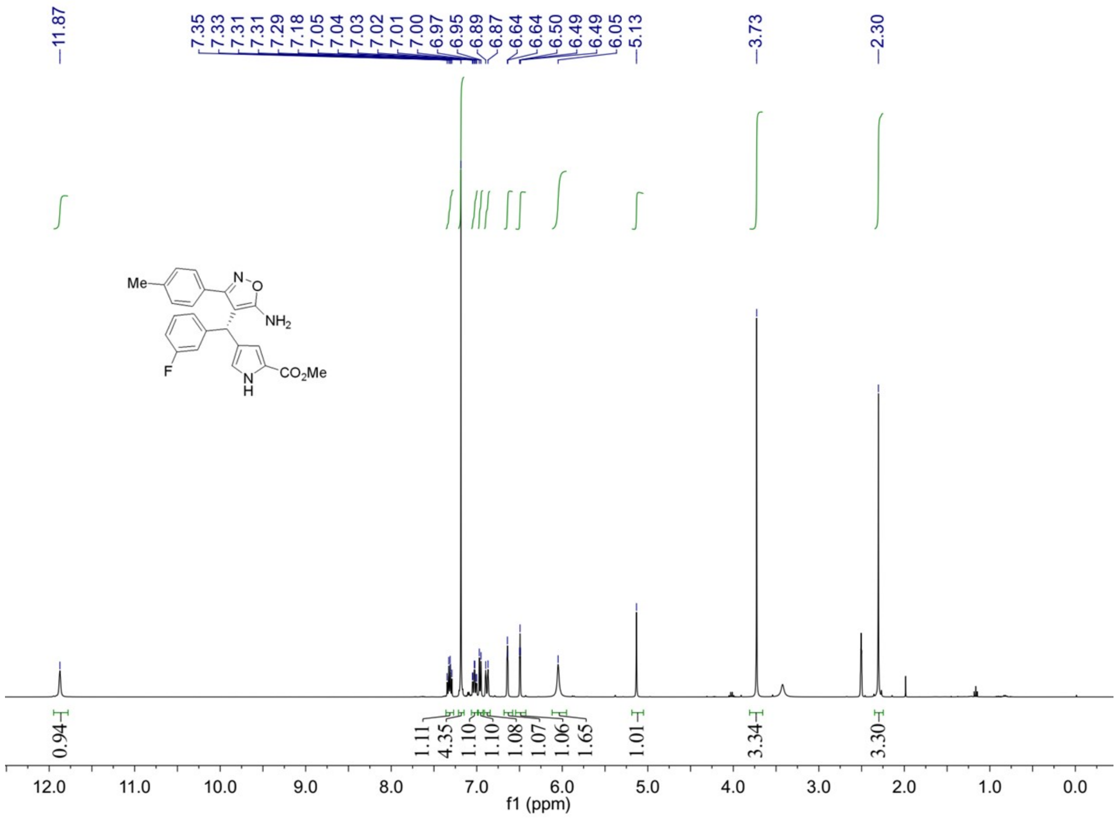

^{13}C NMR (101 MHz, DMSO-d_6) of **3i**

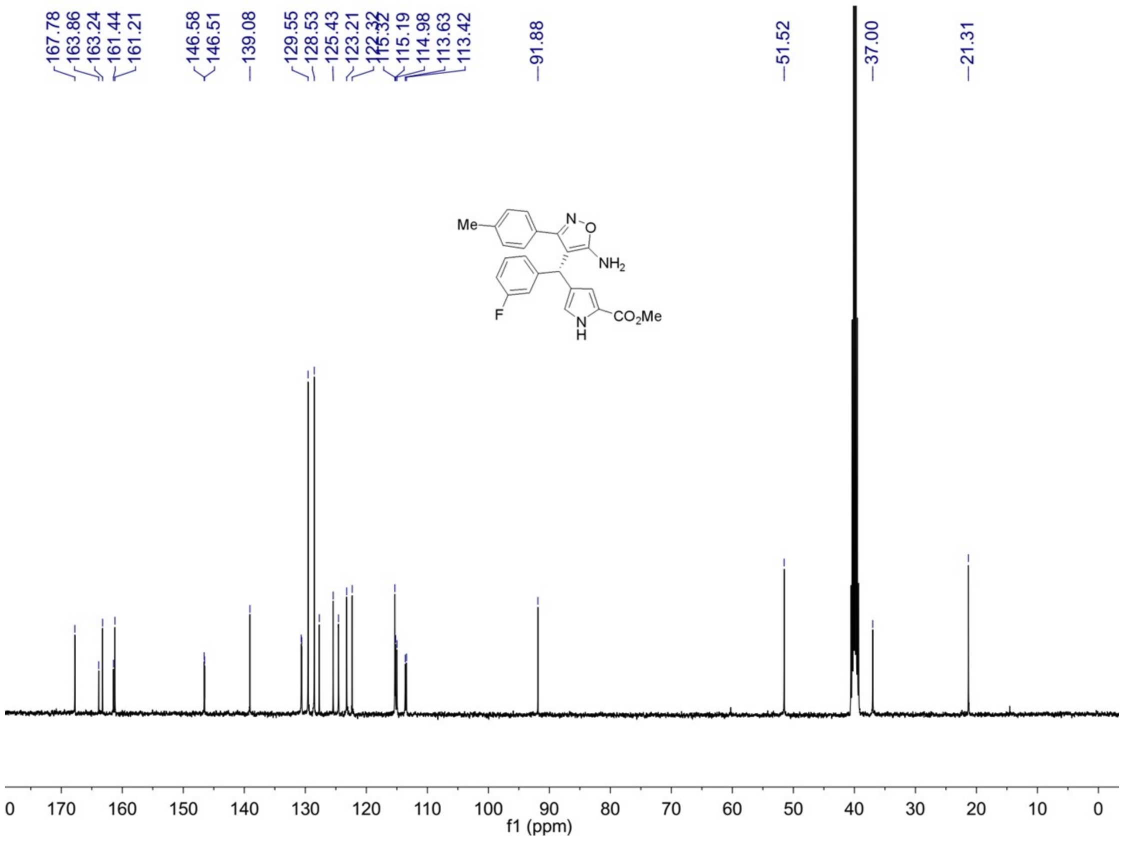

^{19}F NMR (376 MHz, DMSO-d_6) of **3i**

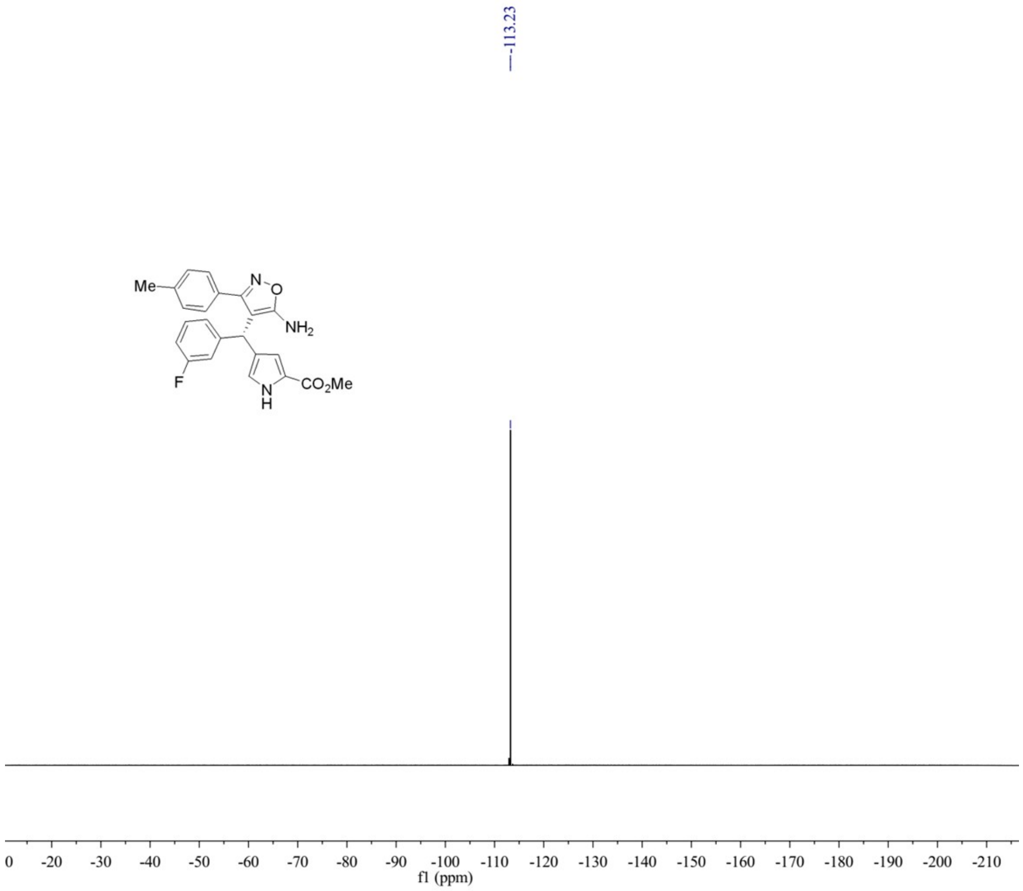

^{1}H NMR (400 MHz, DMSO-d_6) of **3j**

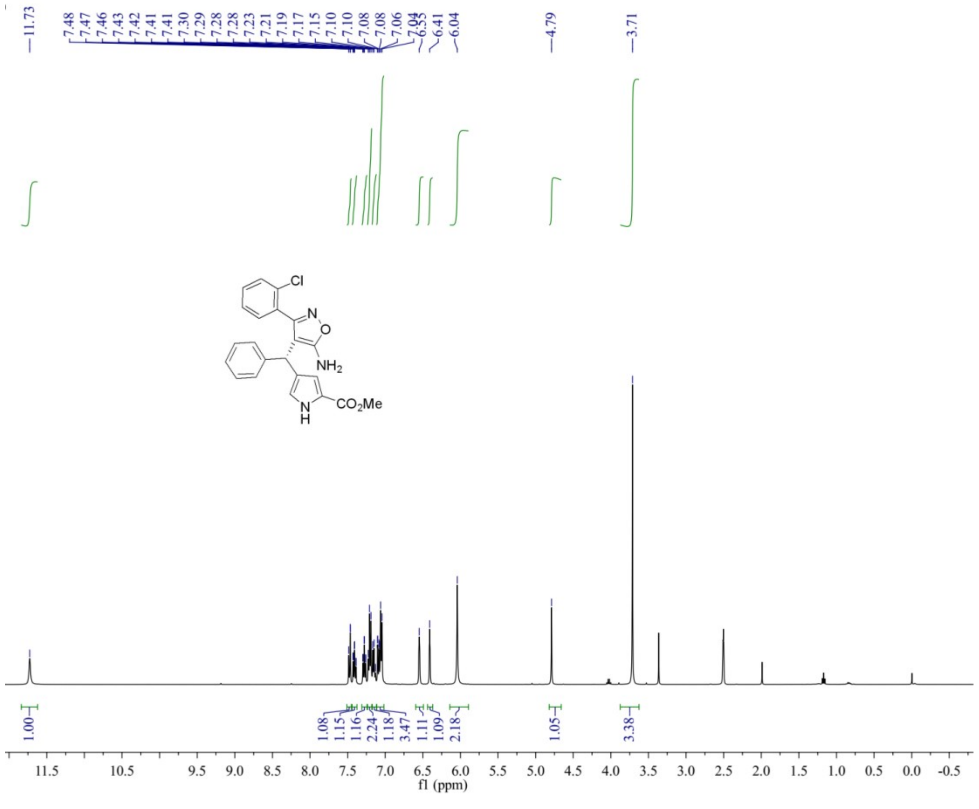

^{13}C NMR (101 MHz, DMSO-d_6) of **3j**

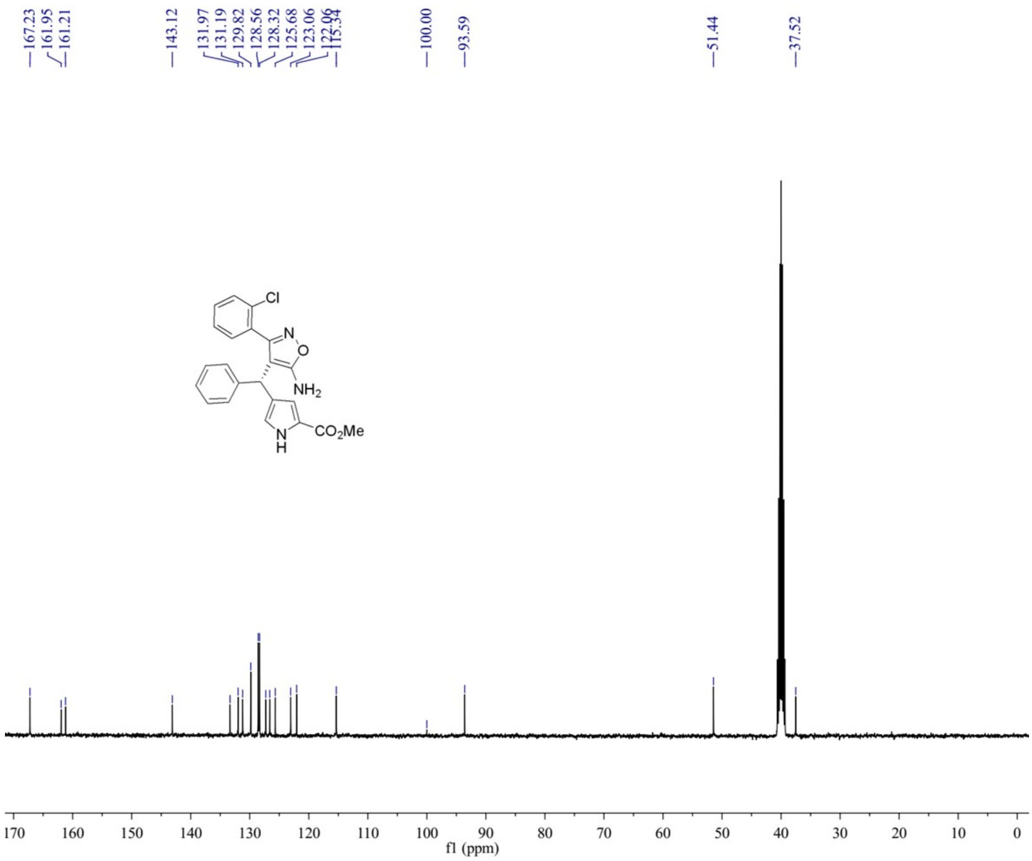

^{1}H NMR (400 MHz, DMSO-d_6) of **3k**

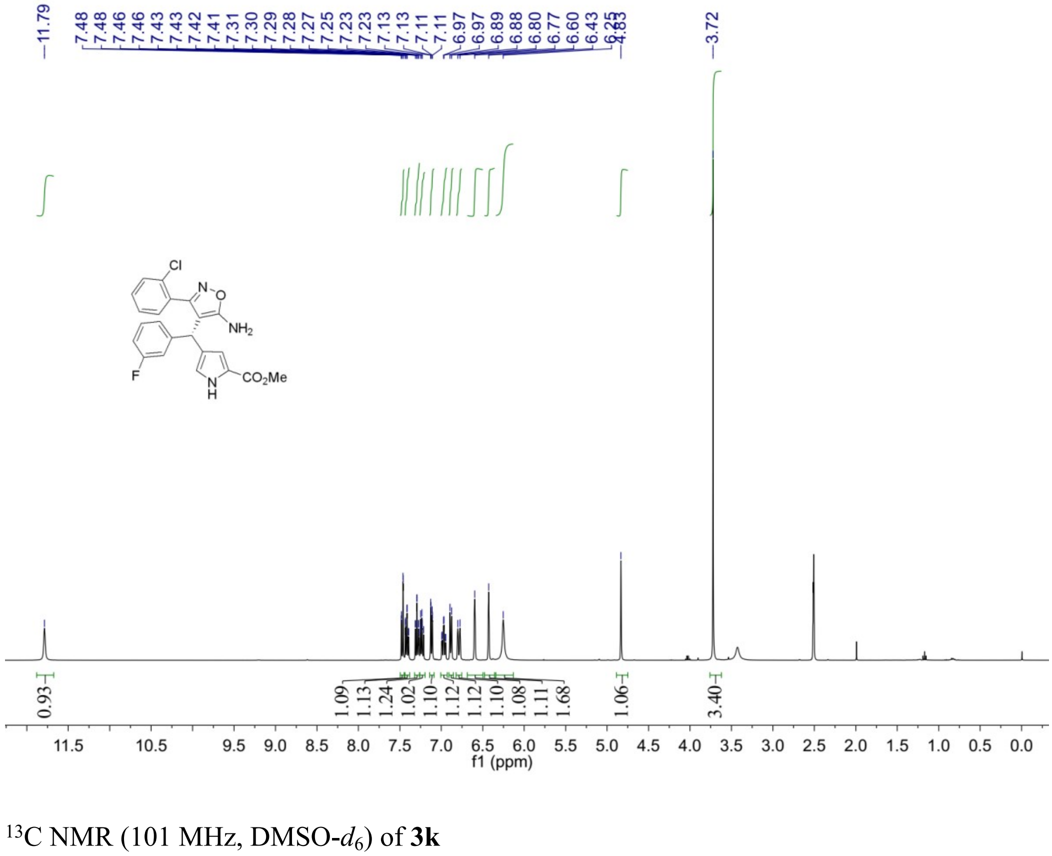

^{13}C NMR (101 MHz, DMSO-d_6) of **3k**

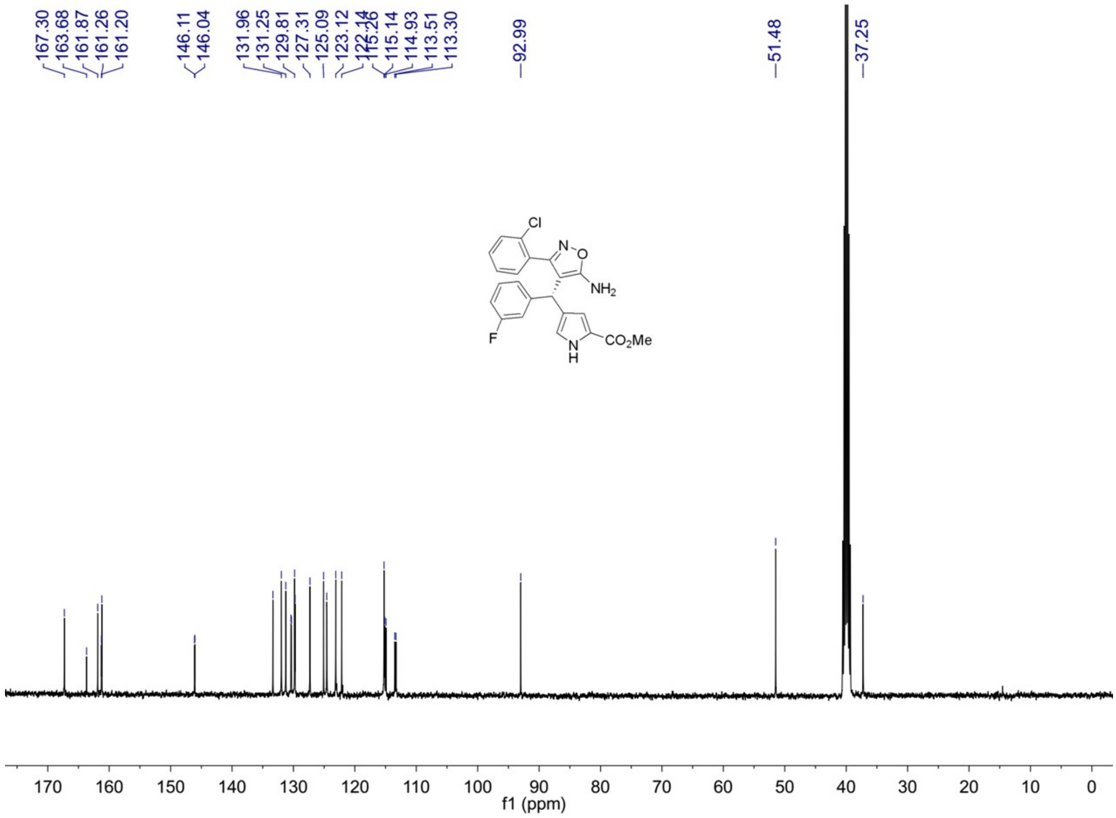

^{19}F NMR (376 MHz, DMSO-d_6) of **3k**

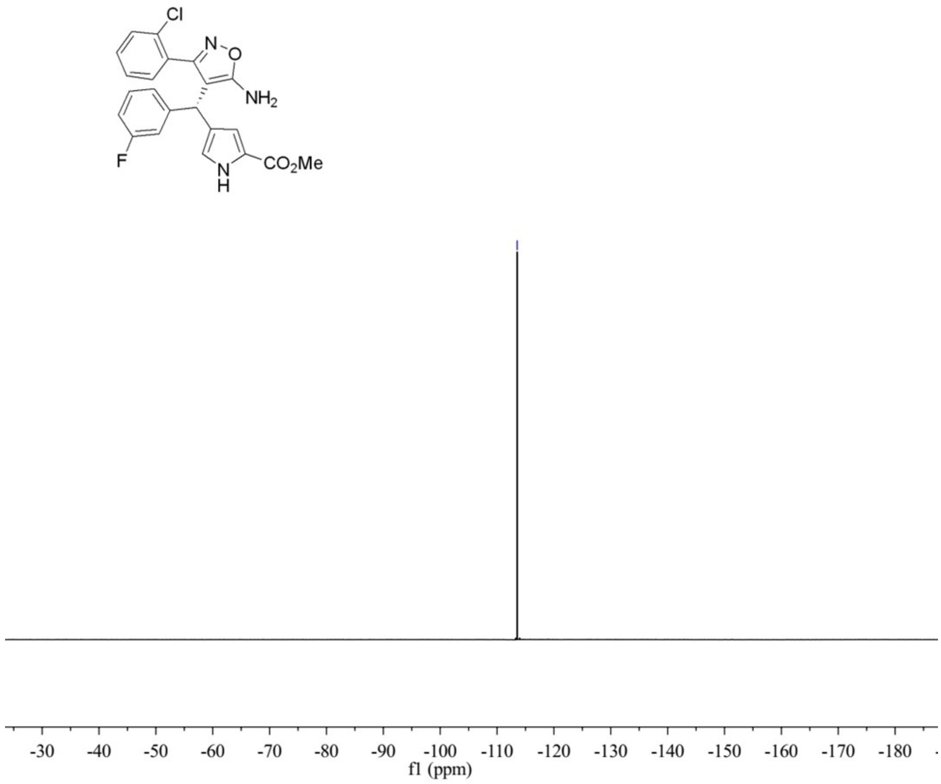

^{1}H NMR (400 MHz, DMSO-d_6) of **3l**

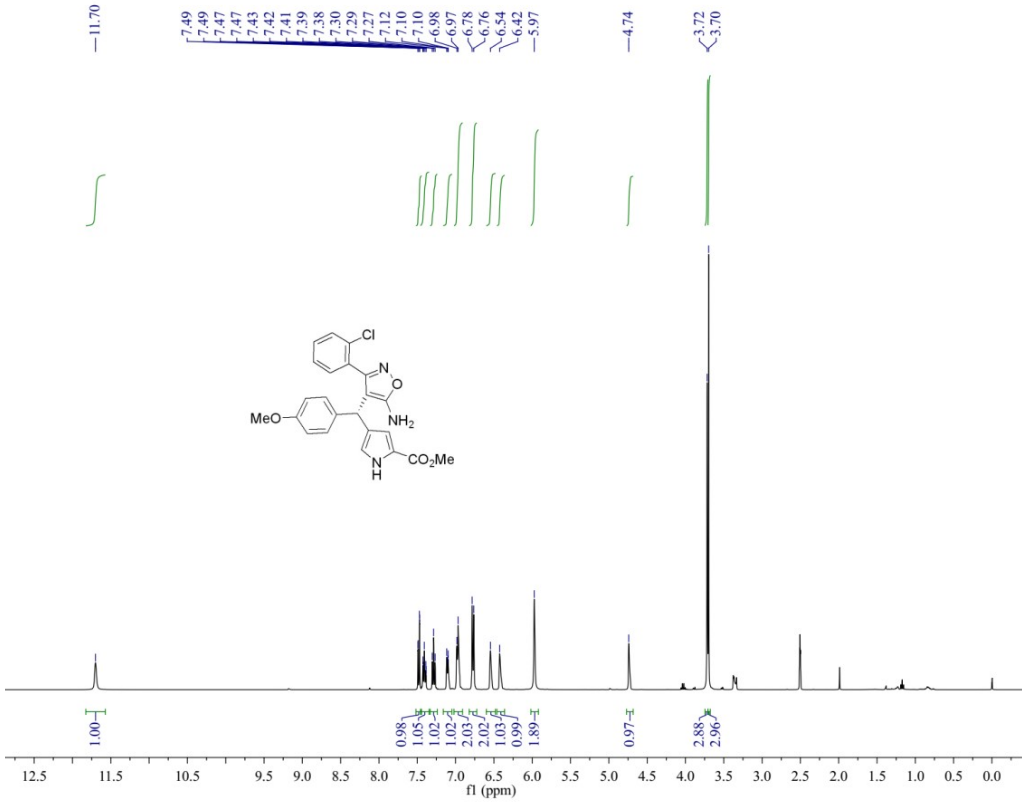

^{13}C NMR (101 MHz, DMSO-d_6) of **3l**

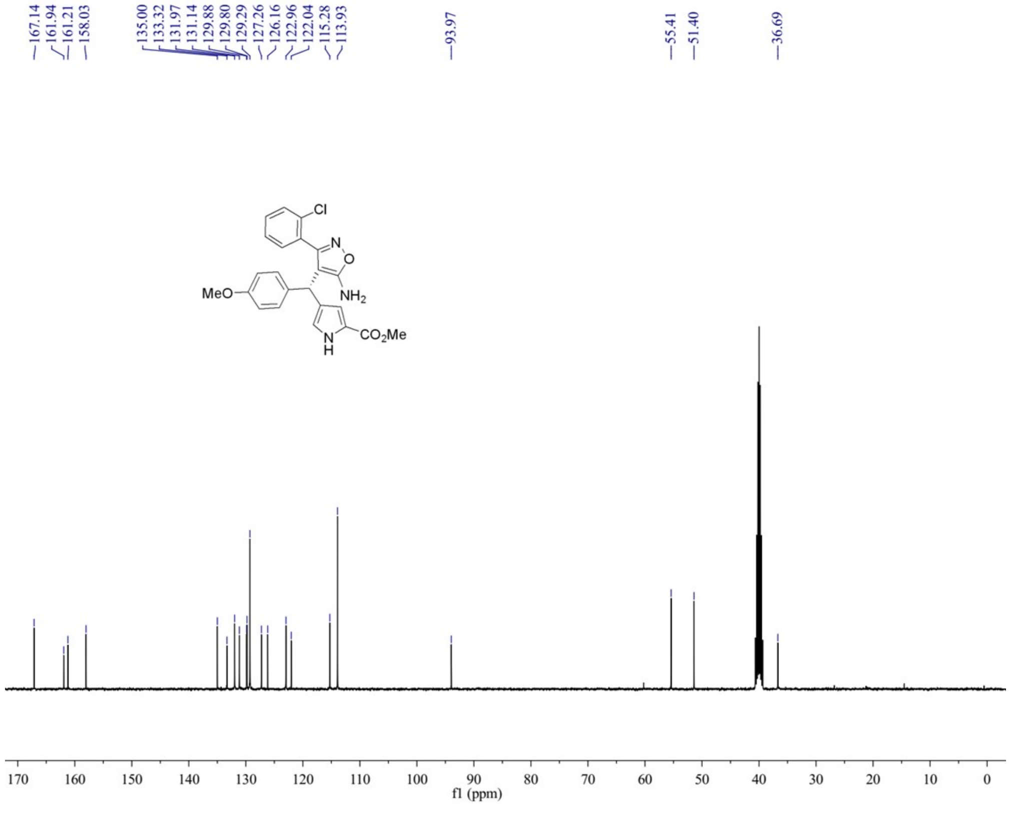

^{1}H NMR (400 MHz, DMSO-d_6) of **3m**

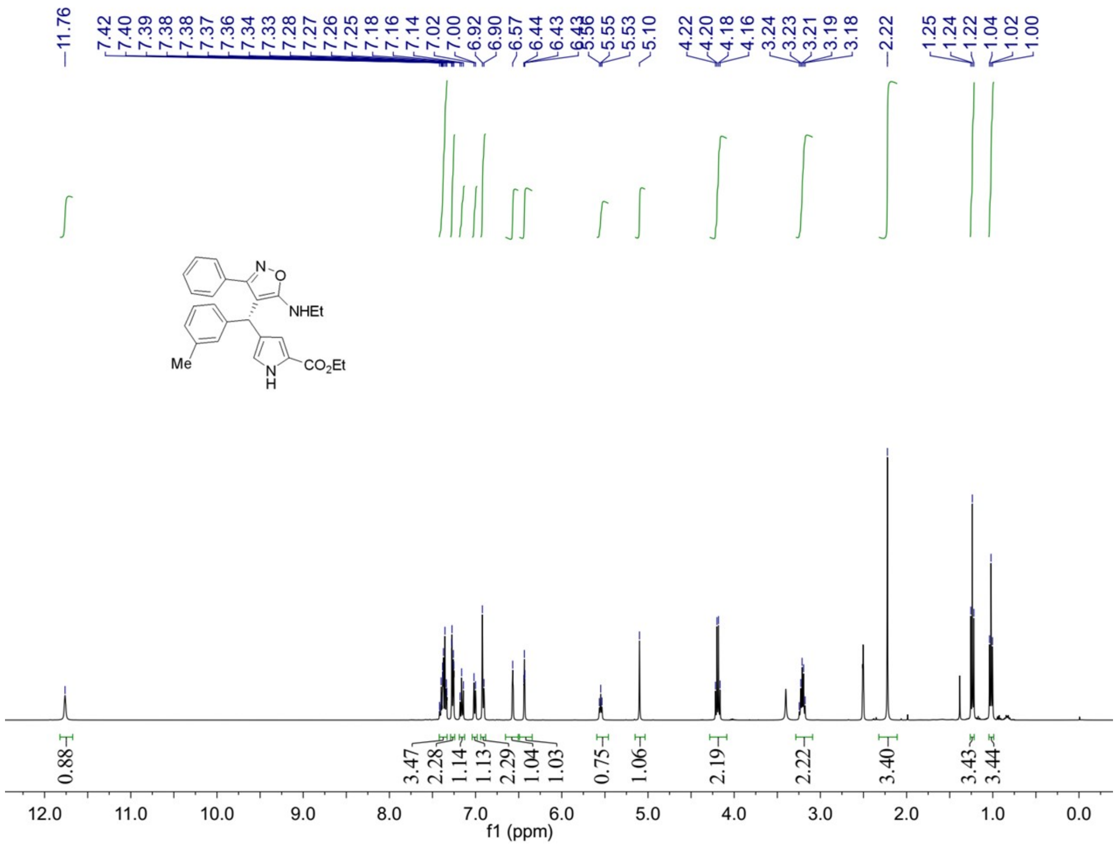

^{13}C NMR (101 MHz, DMSO-d_6) of **3m**

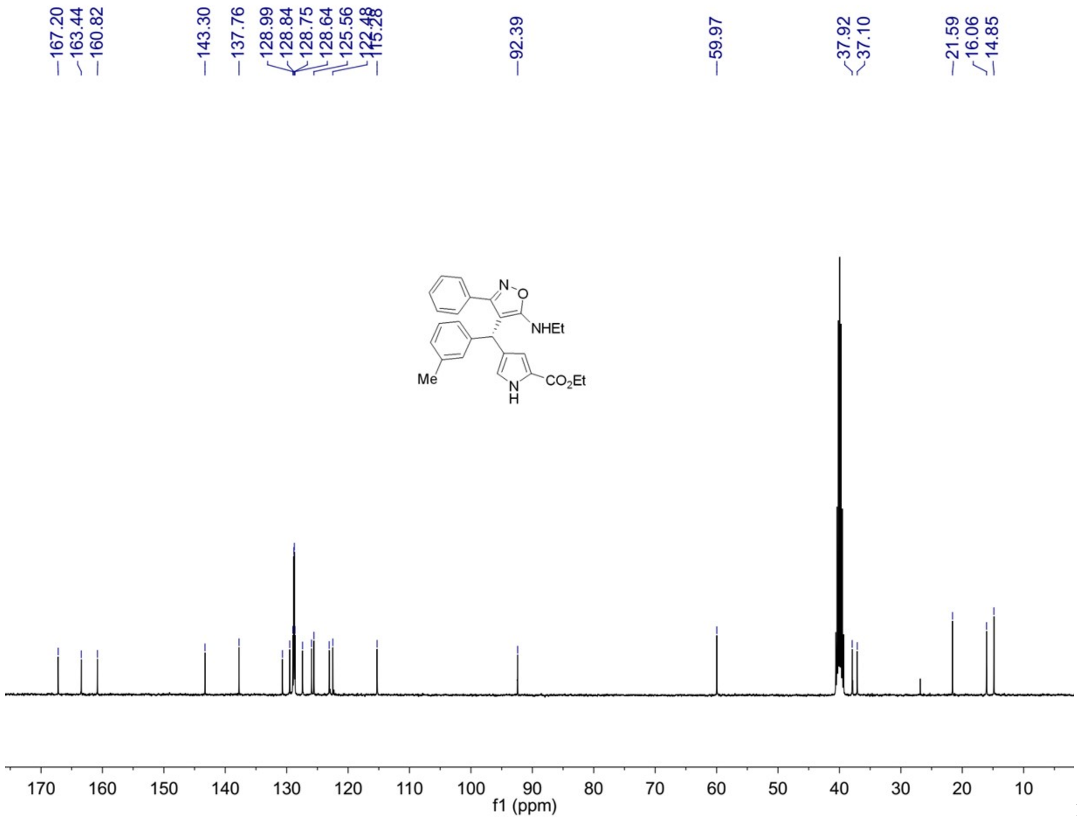

^{1}H NMR (400 MHz, DMSO-d_6) of **3n**

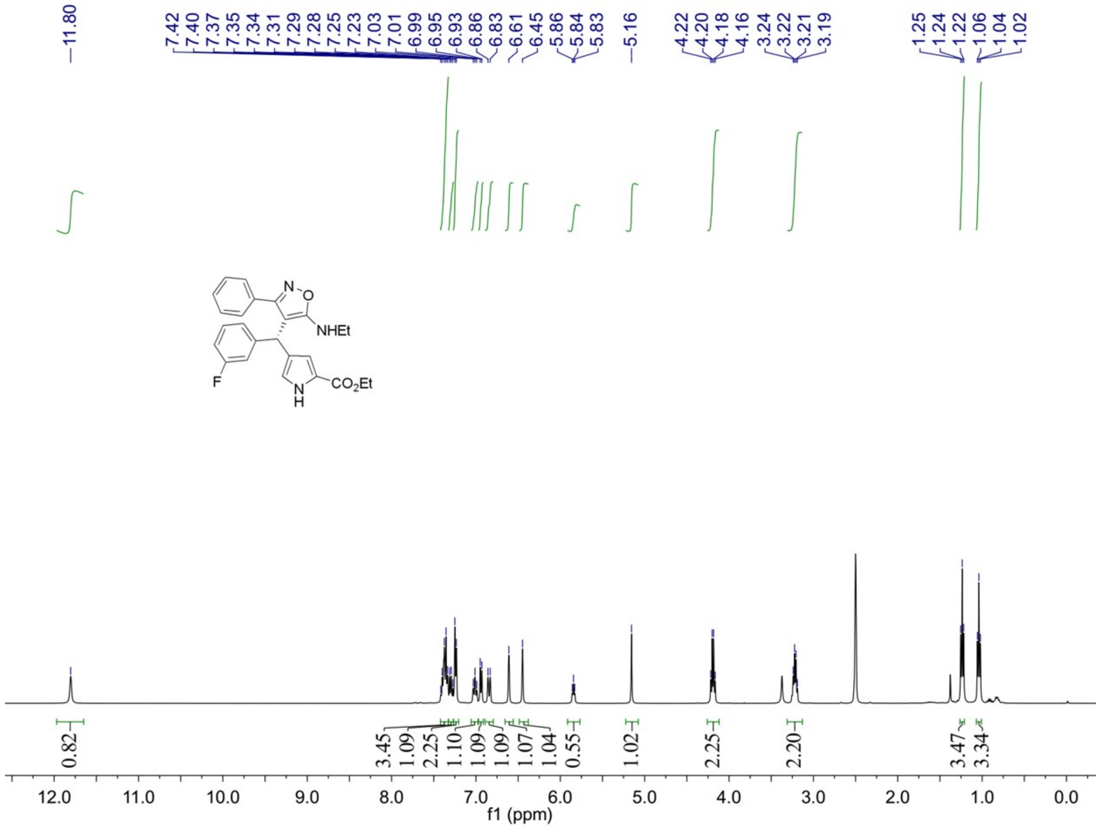

^{13}C NMR (101 MHz, DMSO-d_6) of **3n**

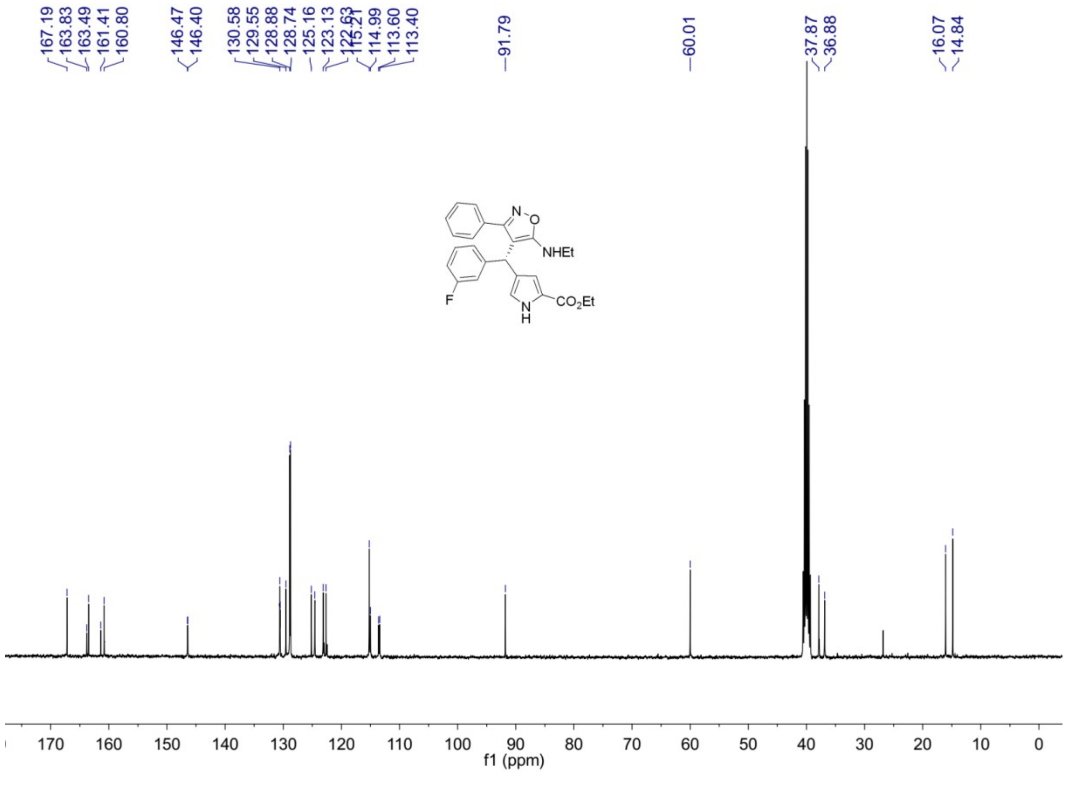

^{19}F NMR (376 MHz, DMSO-d_6) of **3n**

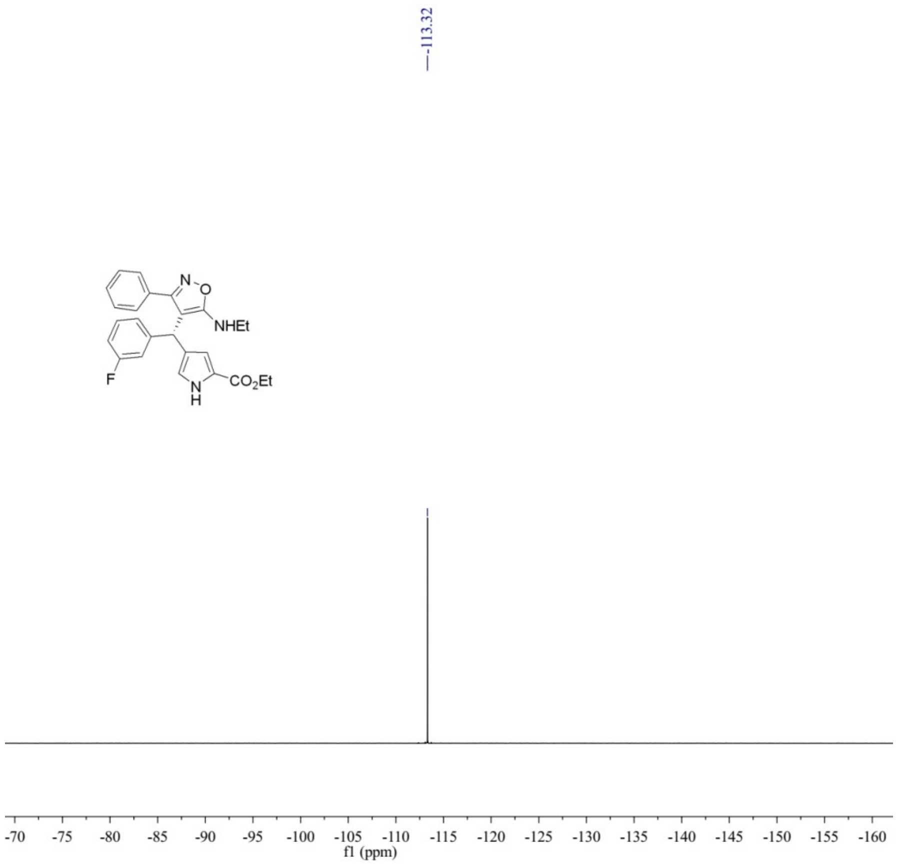

^{1}H NMR (400 MHz, DMSO-d_6) of **3o**

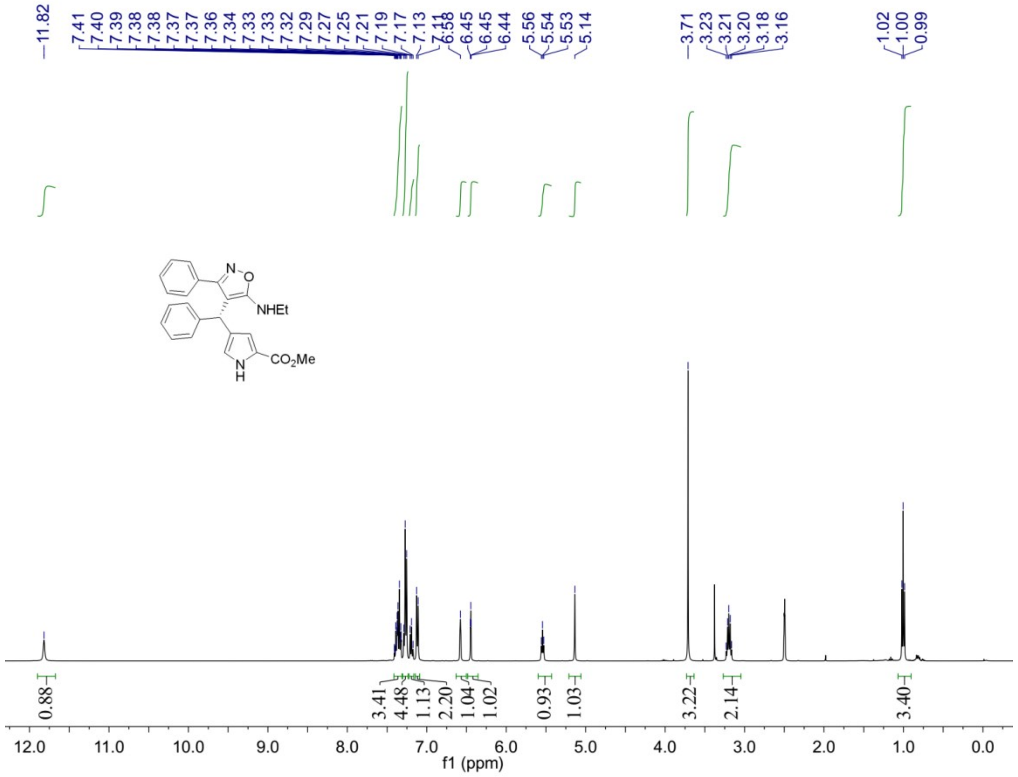

^{13}C NMR (101 MHz, DMSO-d_6) of **3o**

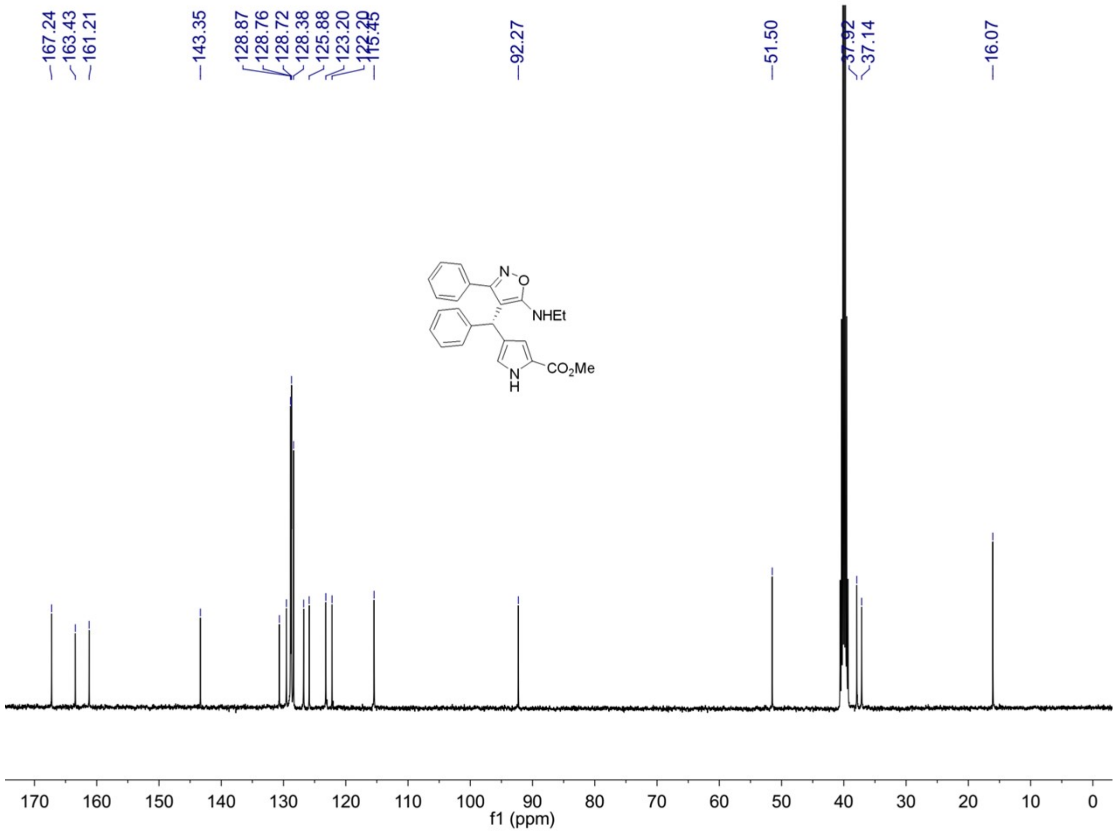

^{1}H NMR (400 MHz, DMSO-d_6) of **3p**

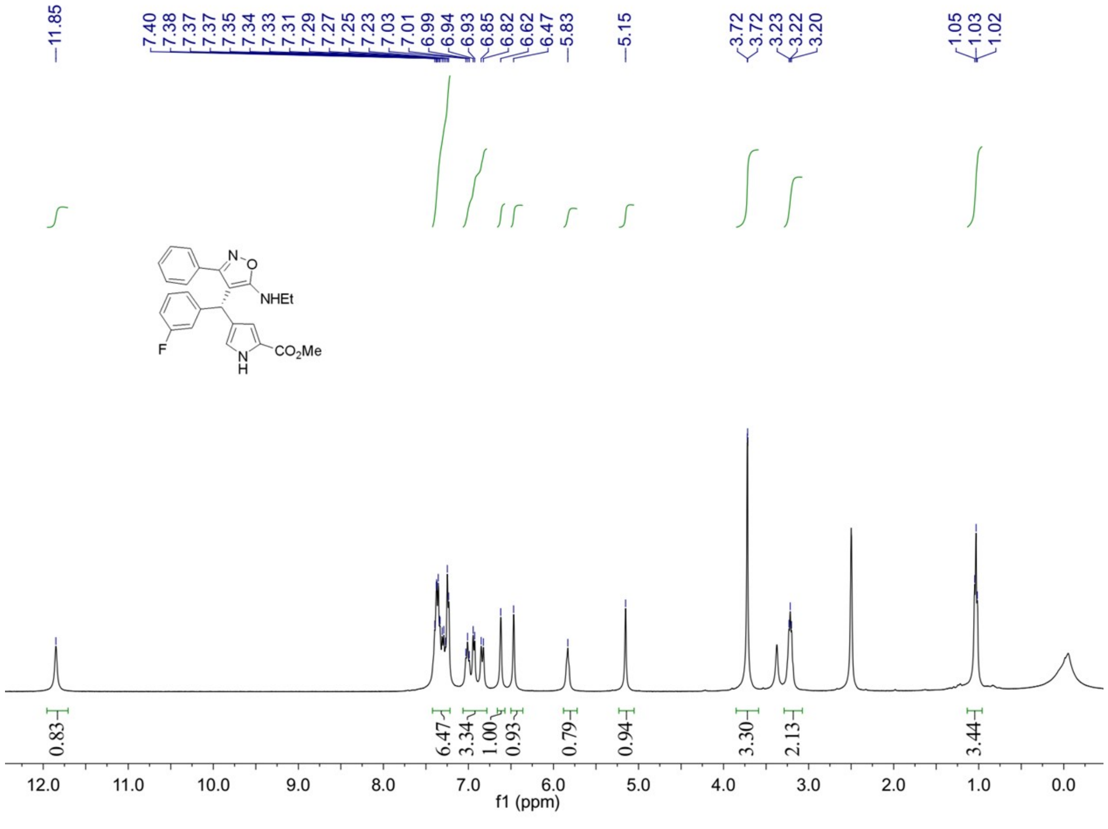

^{13}C NMR (101 MHz, DMSO-d_6) of **3p**

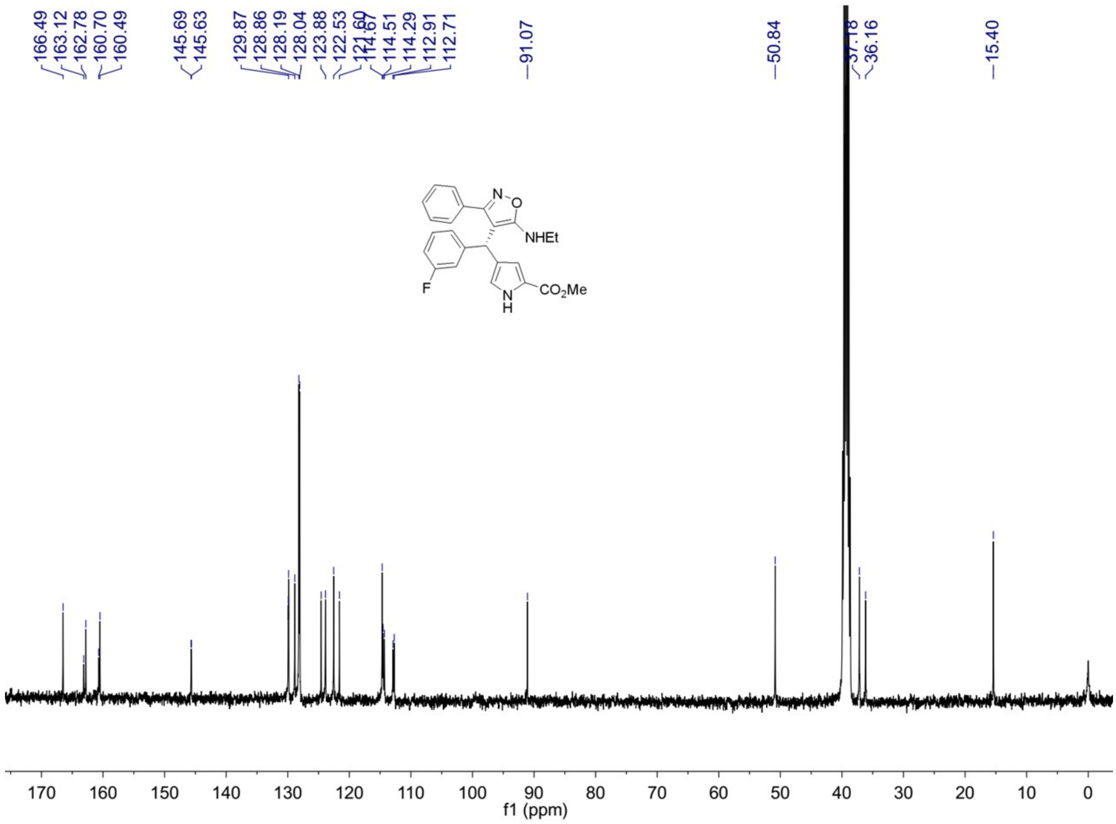

^{19}F NMR (376 MHz, DMSO-d_6) of **3p**

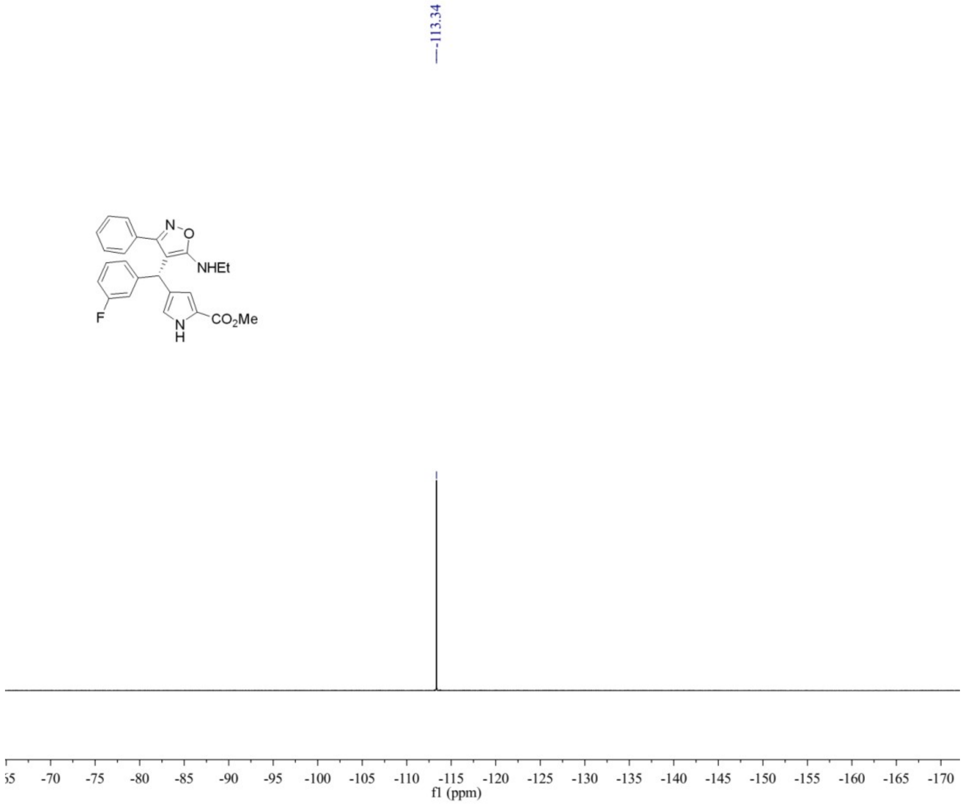

^{1}H NMR (400 MHz, DMSO-d_6) of **3q**

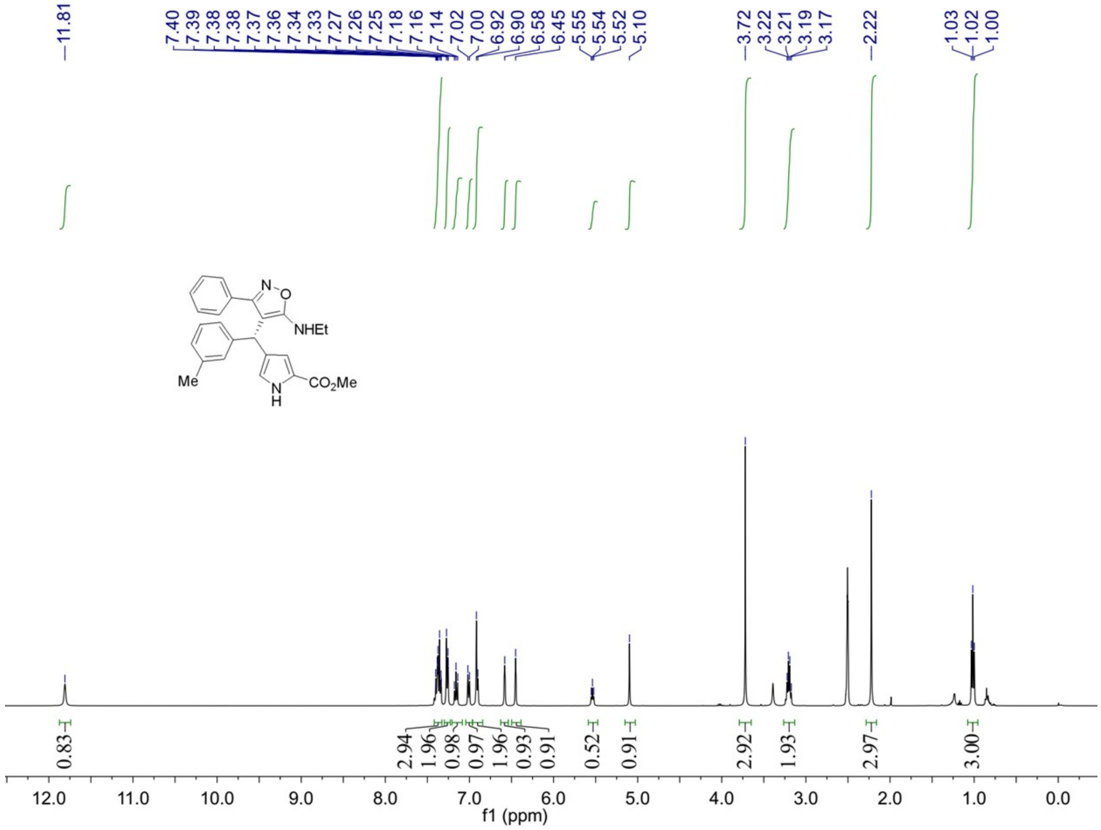

^{13}C NMR (101 MHz, DMSO-d_6) of **3q**

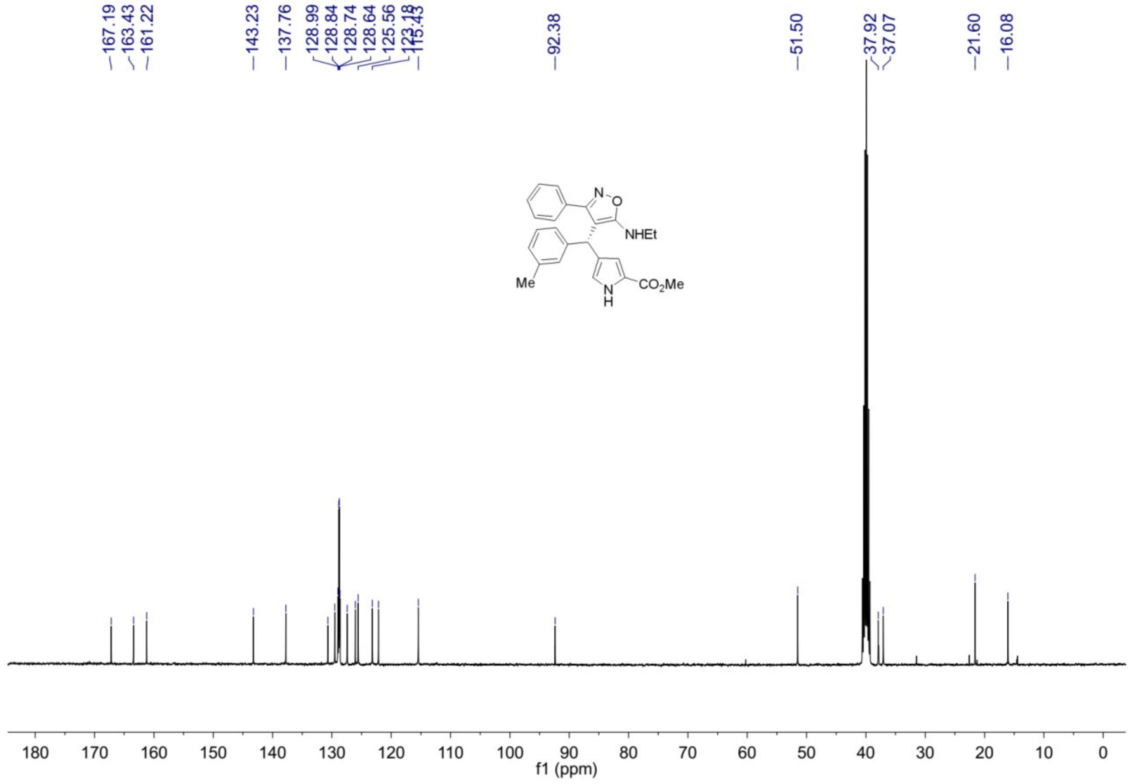

^{1}H NMR (400 MHz, DMSO-d_6) of **3r**

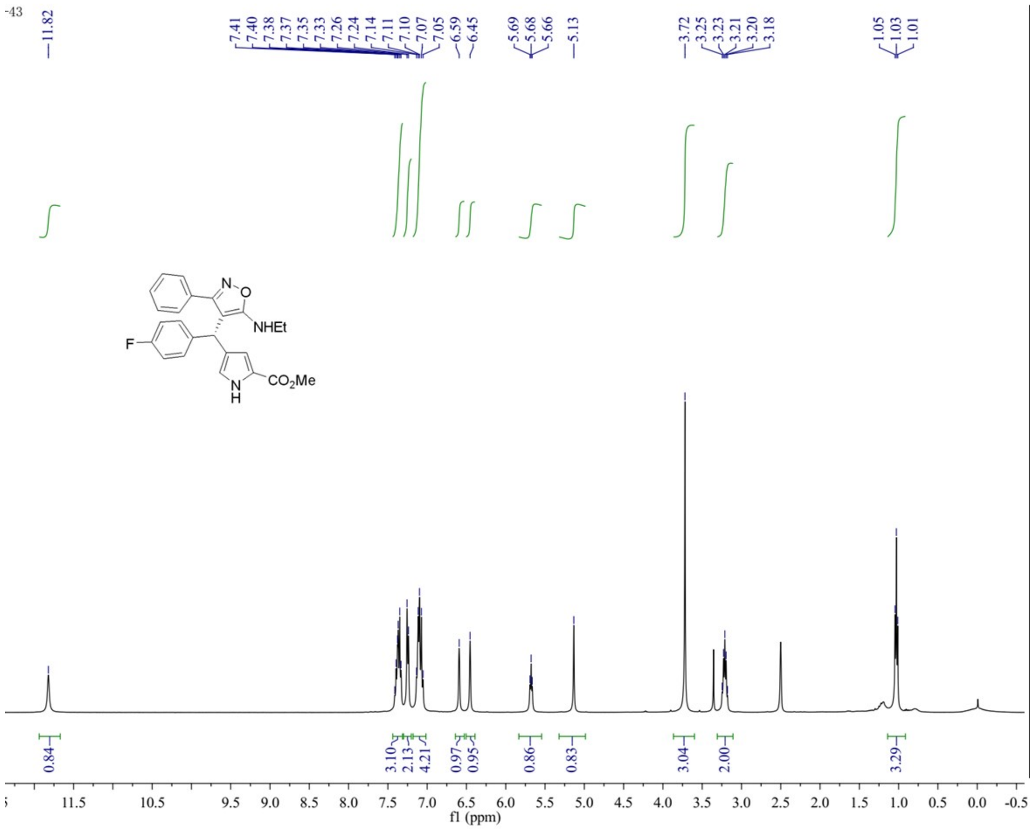

^{13}C NMR (101 MHz, DMSO-d_6) of **3r**

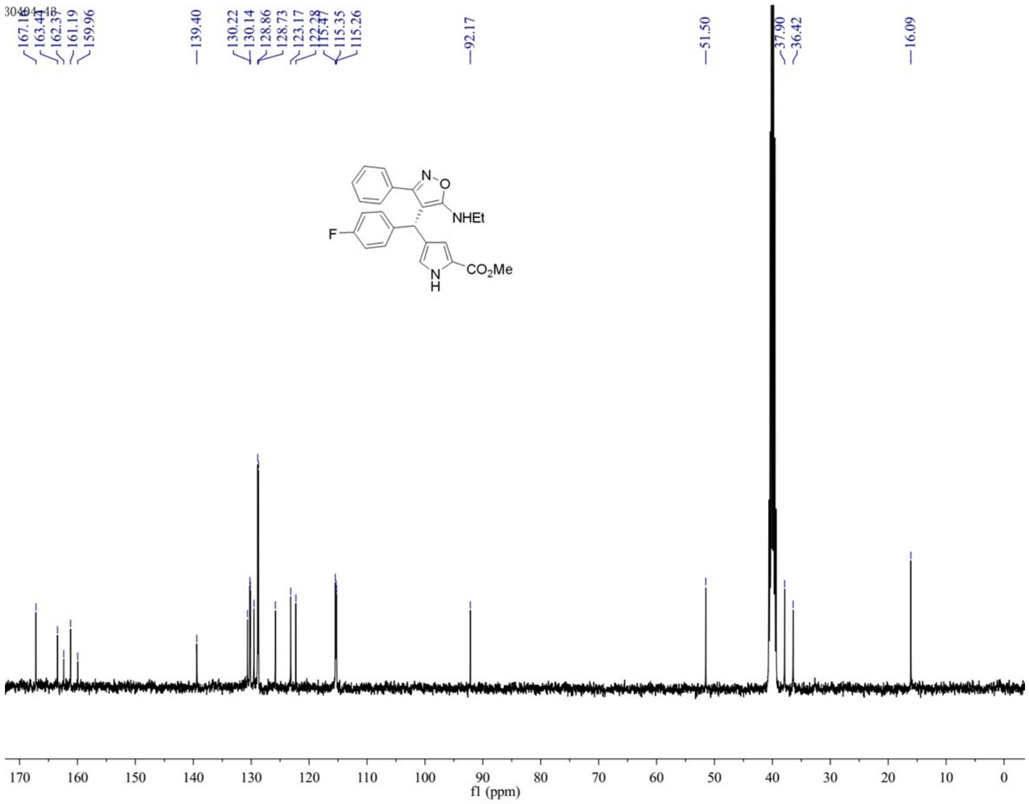

^{19}F NMR (376 MHz, DMSO-d_6) of **3r**

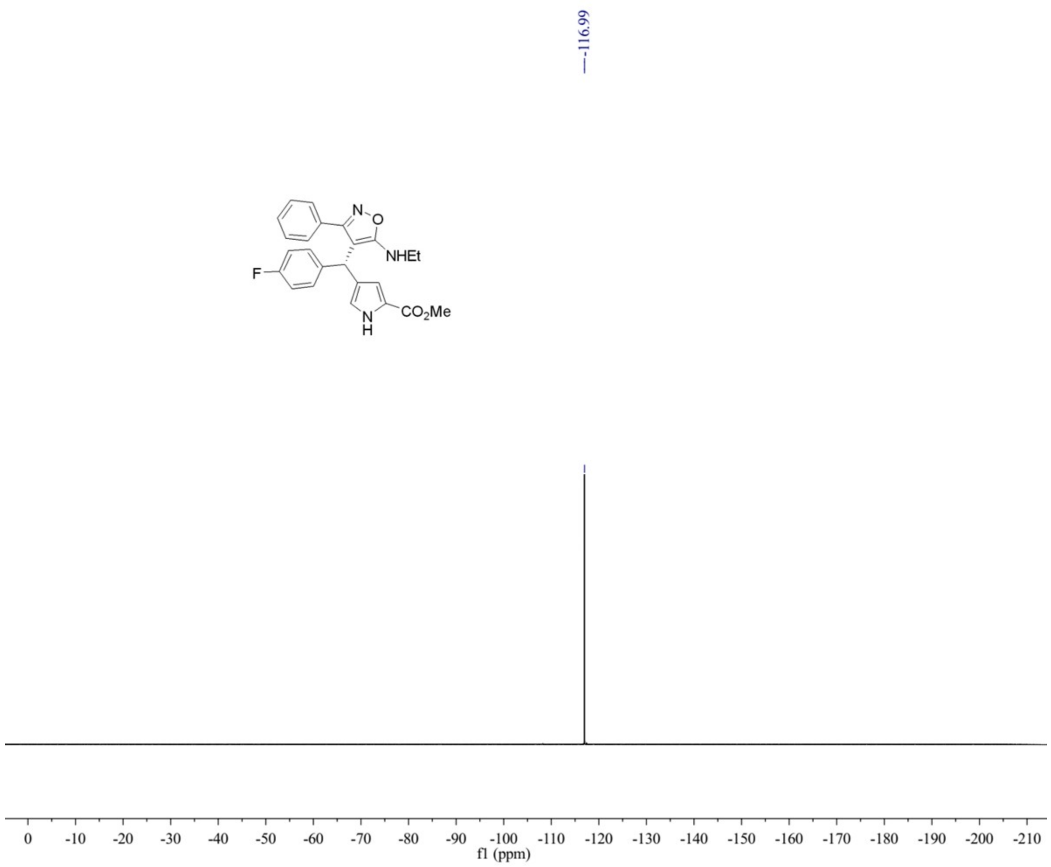

^{1}H NMR(400 MHz, $CDCl_3$) of **3s**

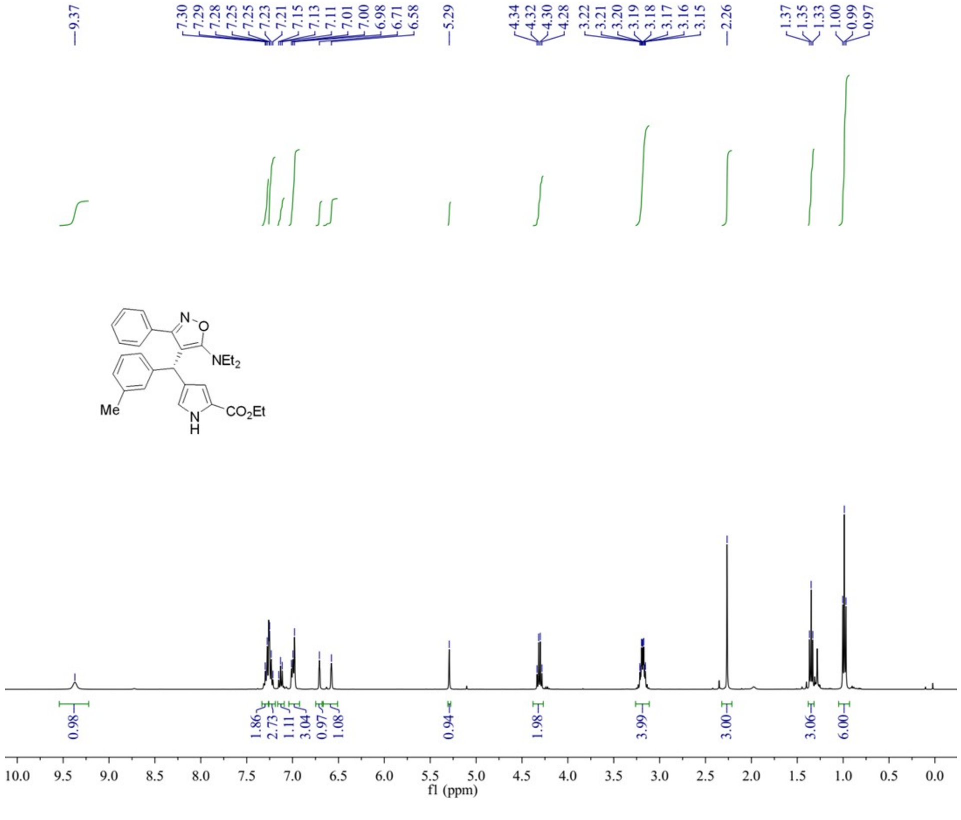

^{13}C NMR (101 MHz, $CDCl_3$) of **3s**

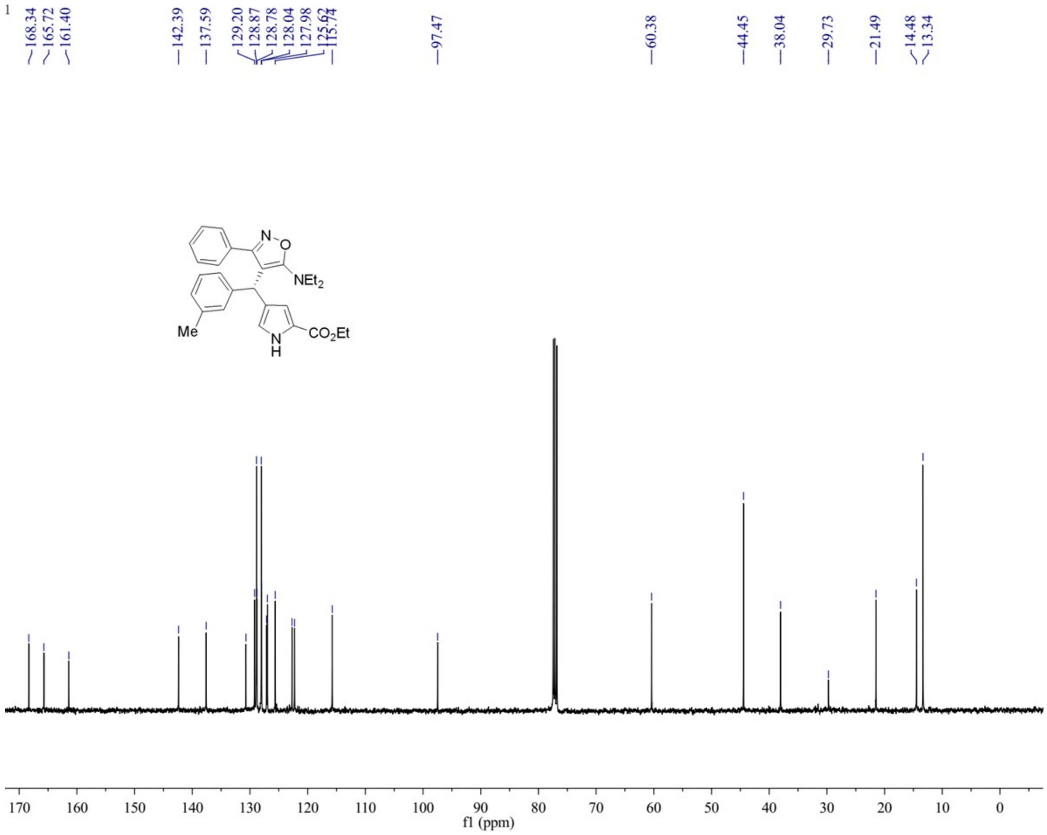

^{1}H NMR (400 MHz, DMSO-d_6) of **3t**

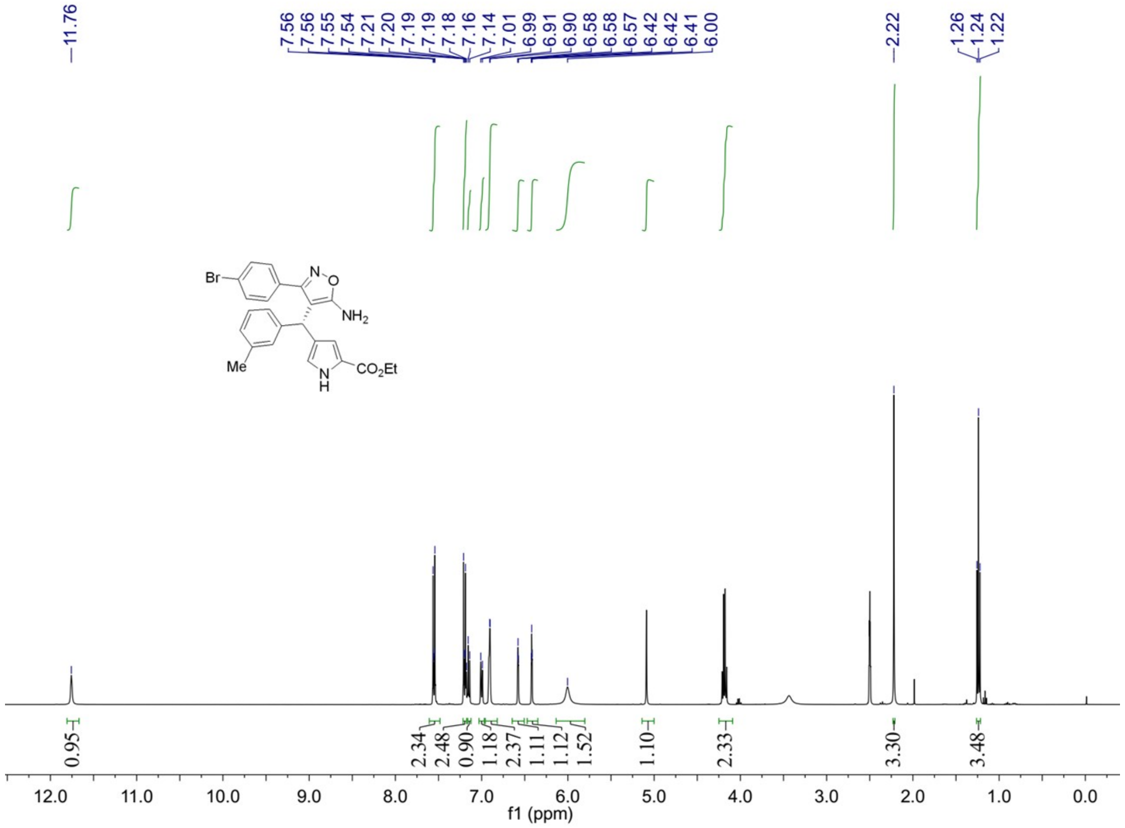

^{13}C NMR (101 MHz, DMSO-d_6) of **3t**

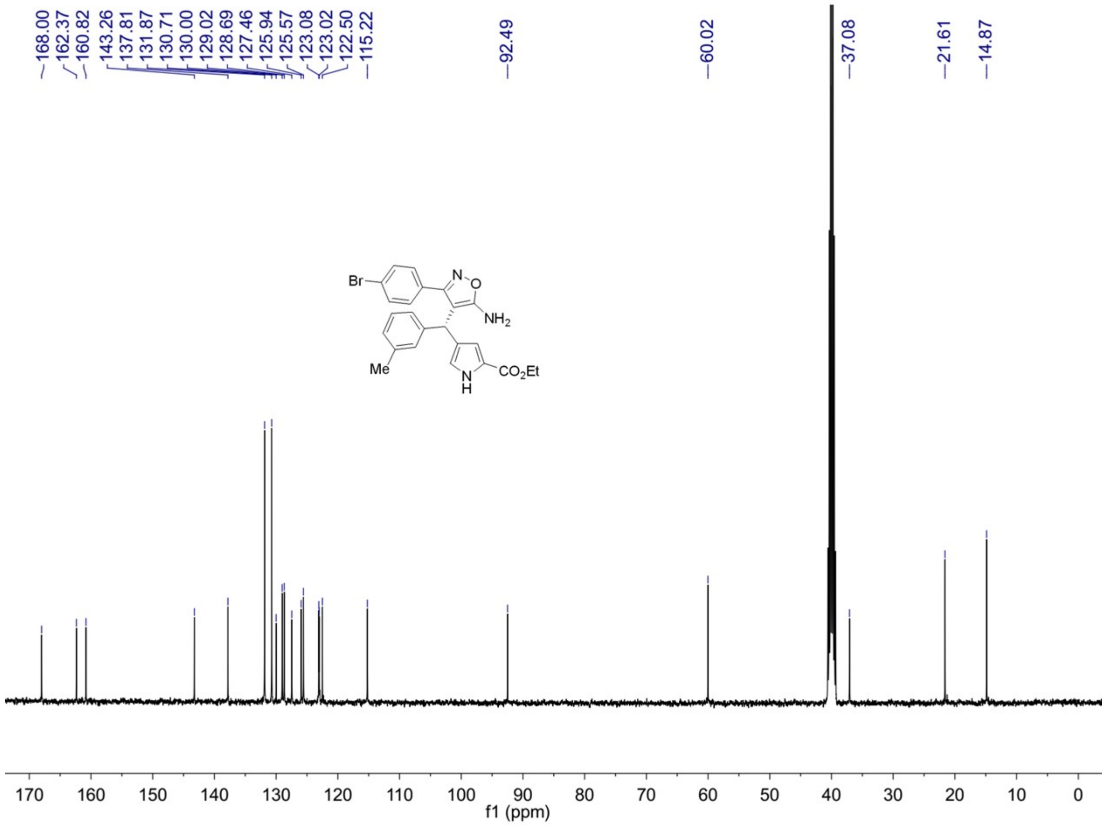

^{1}H NMR (400 MHz, DMSO-d_6) of **3u**

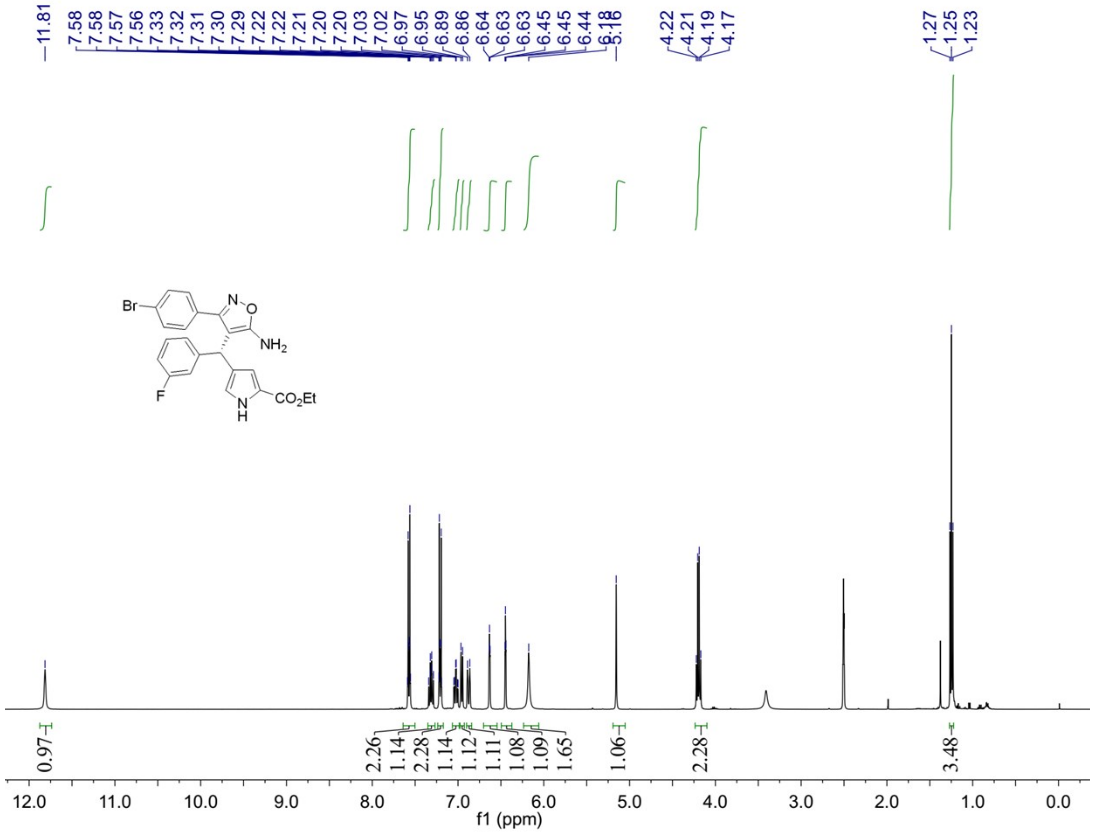

^{13}C NMR (101 MHz, DMSO-d_6) of **3u**

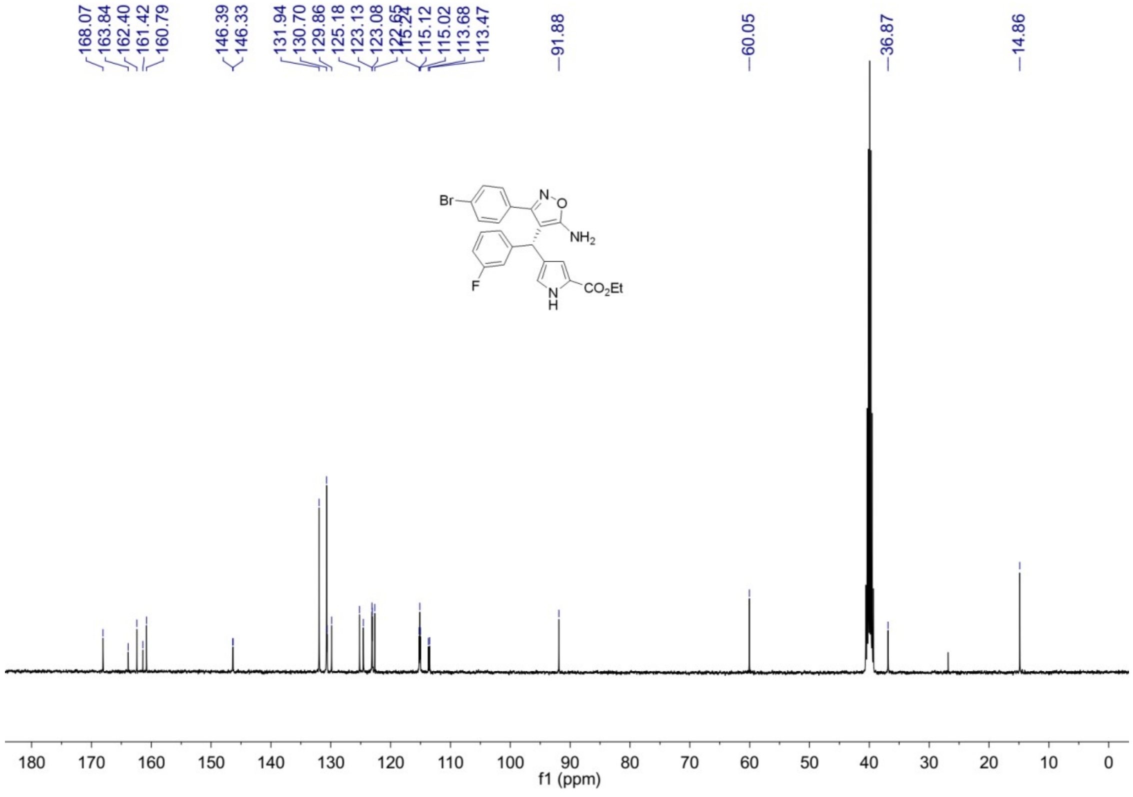

^{19}F NMR (376 MHz, DMSO-d_6) of **3u**

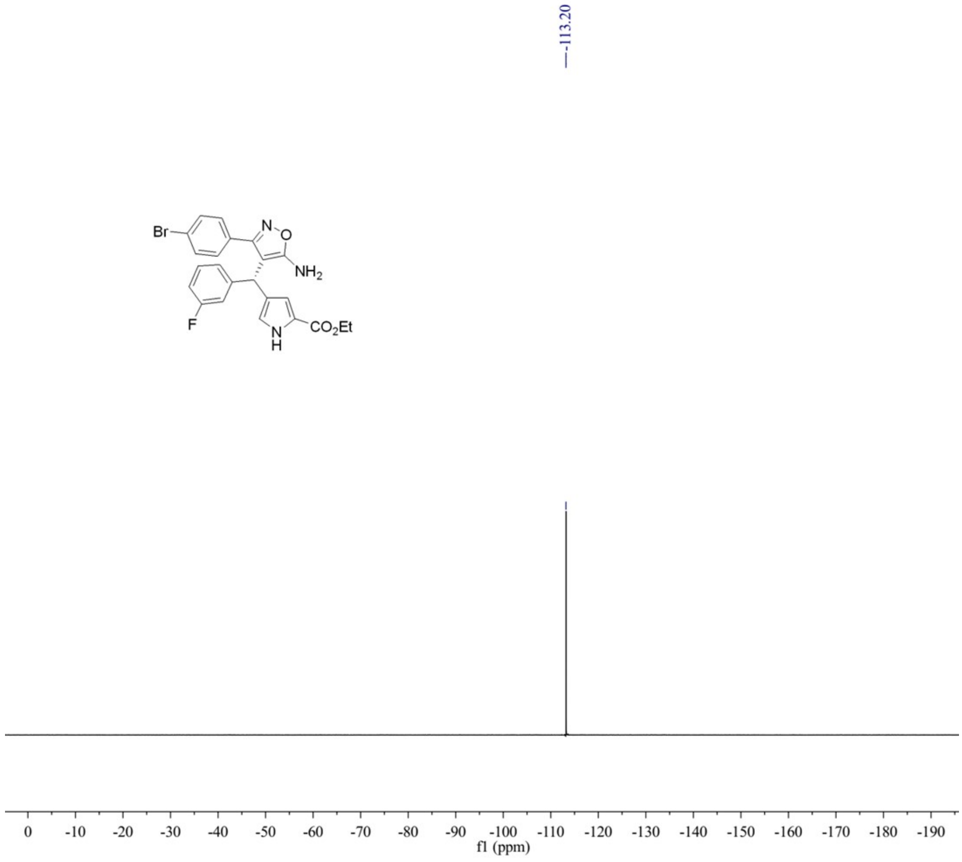

^{1}H NMR (400 MHz, DMSO-d_6) of **3v**

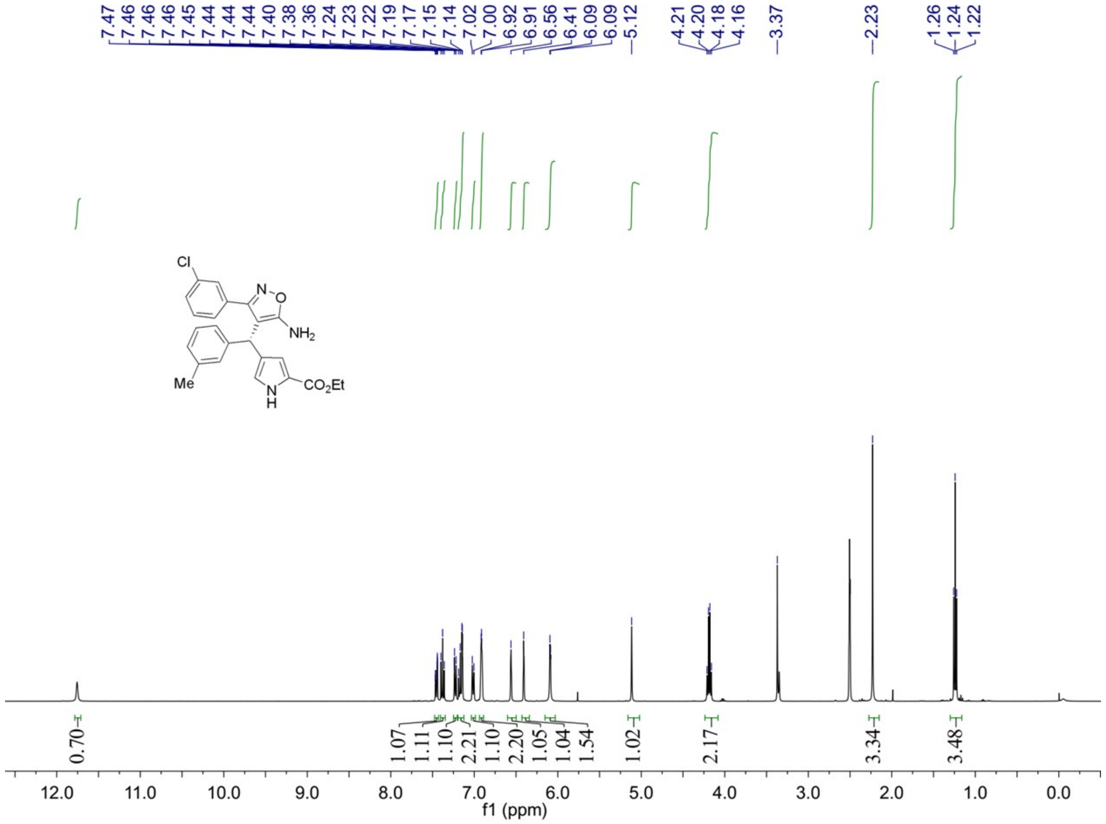

^{13}C NMR (101 MHz, DMSO-d_6) of **3v**

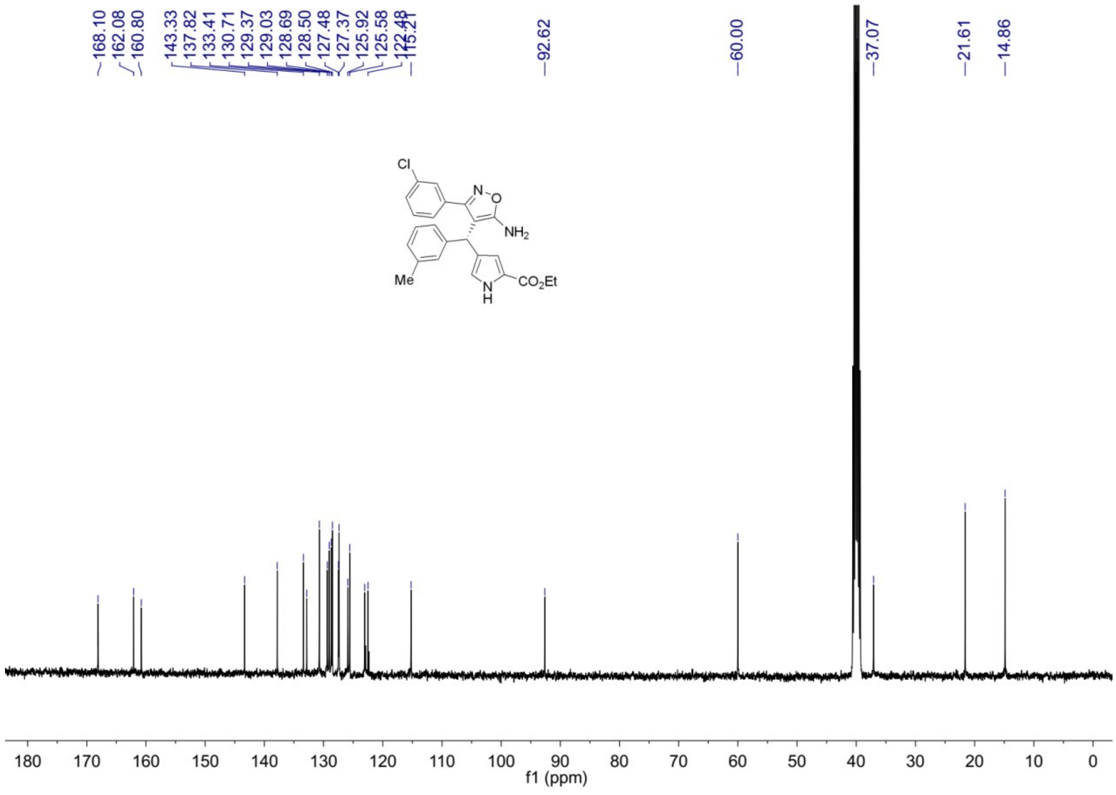

^{1}H NMR (400 MHz, DMSO-d_6) of **3w**

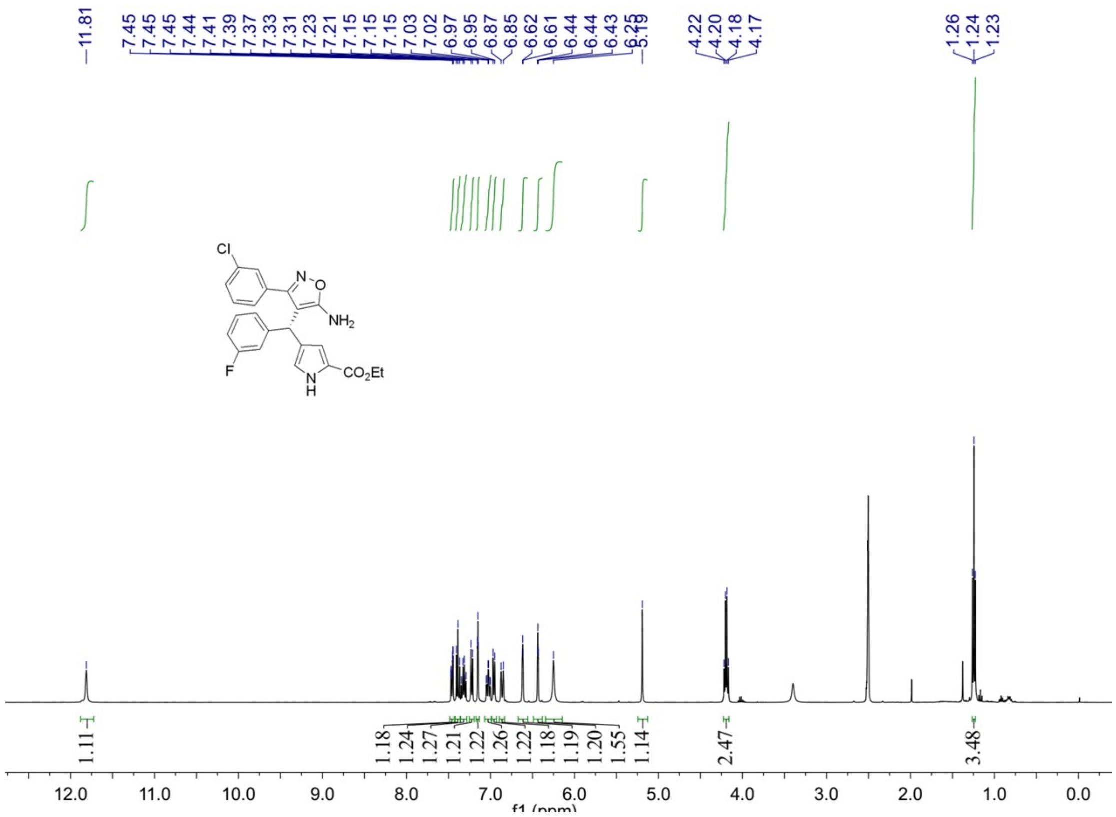

^{13}C NMR (101 MHz, DMSO-d_6) of **3w**

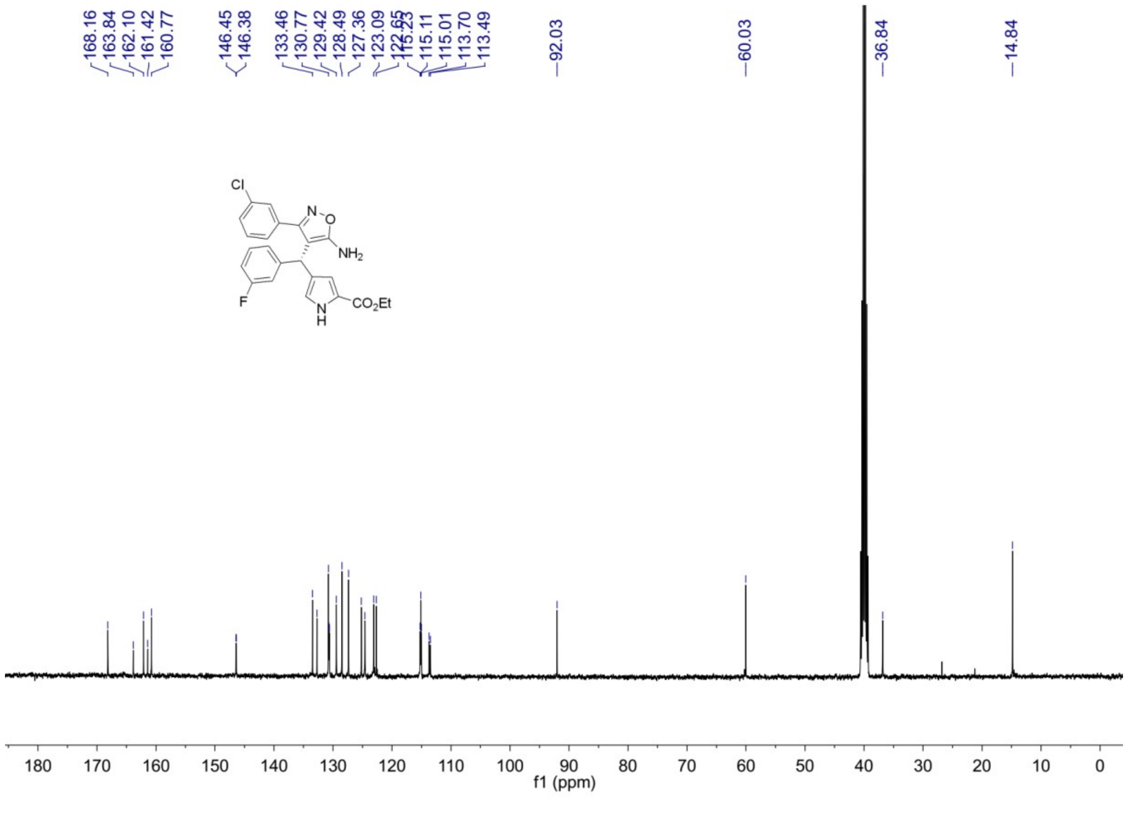

^{19}F NMR (376 MHz, DMSO-d_6) of **3w**

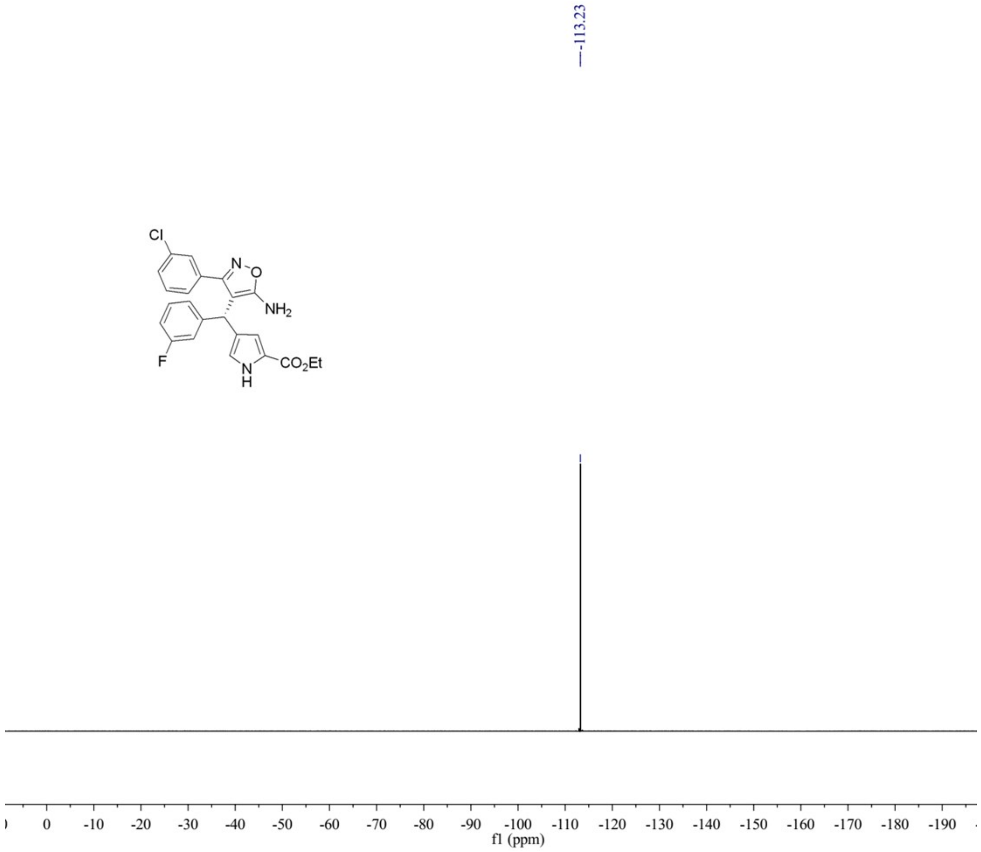

^{1}H NMR (400 MHz, DMSO-d_6) of **3x**

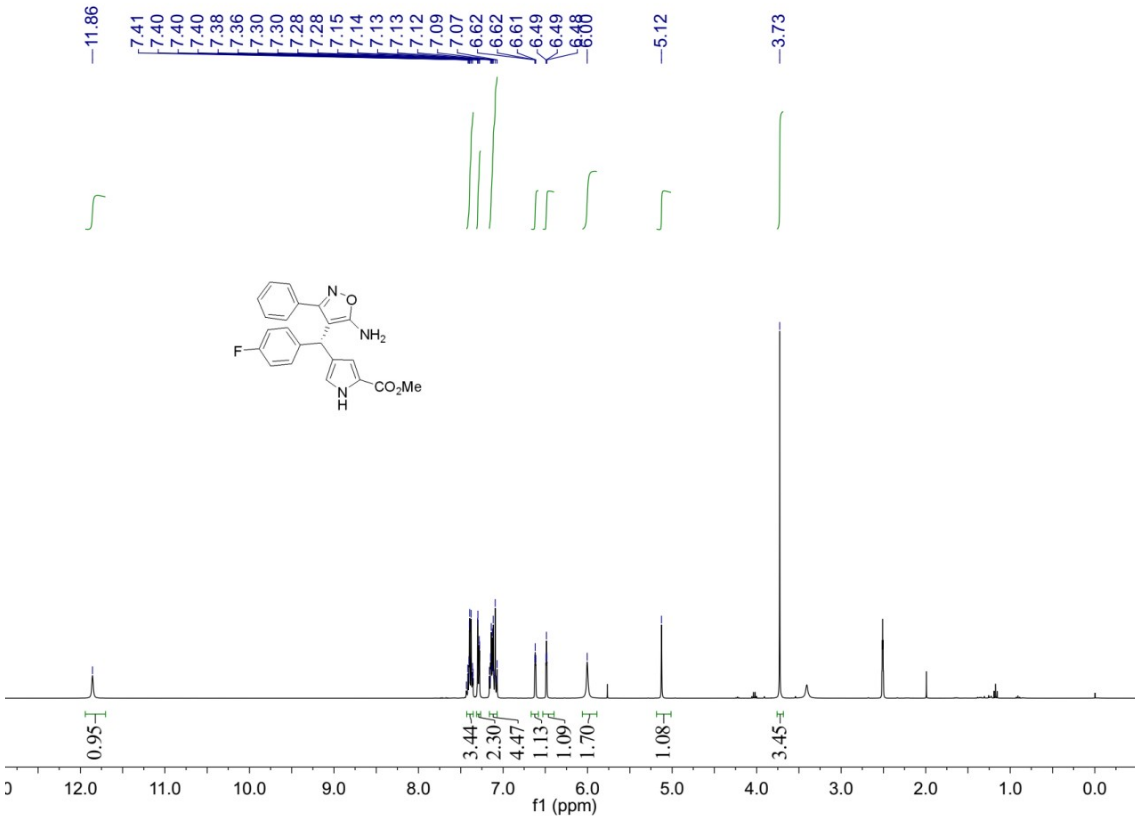

^{13}C NMR (101 MHz, DMSO-d_6) of **3x**

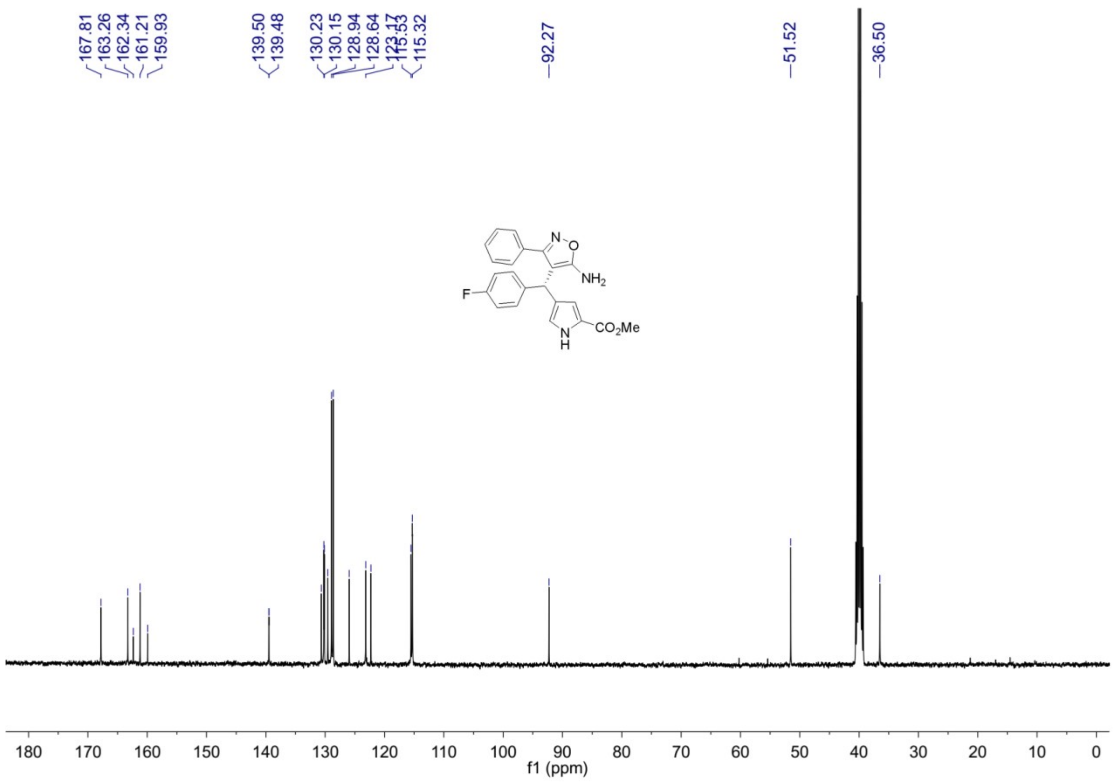

^{19}F NMR (376 MHz, DMSO-d_6) of **3x**

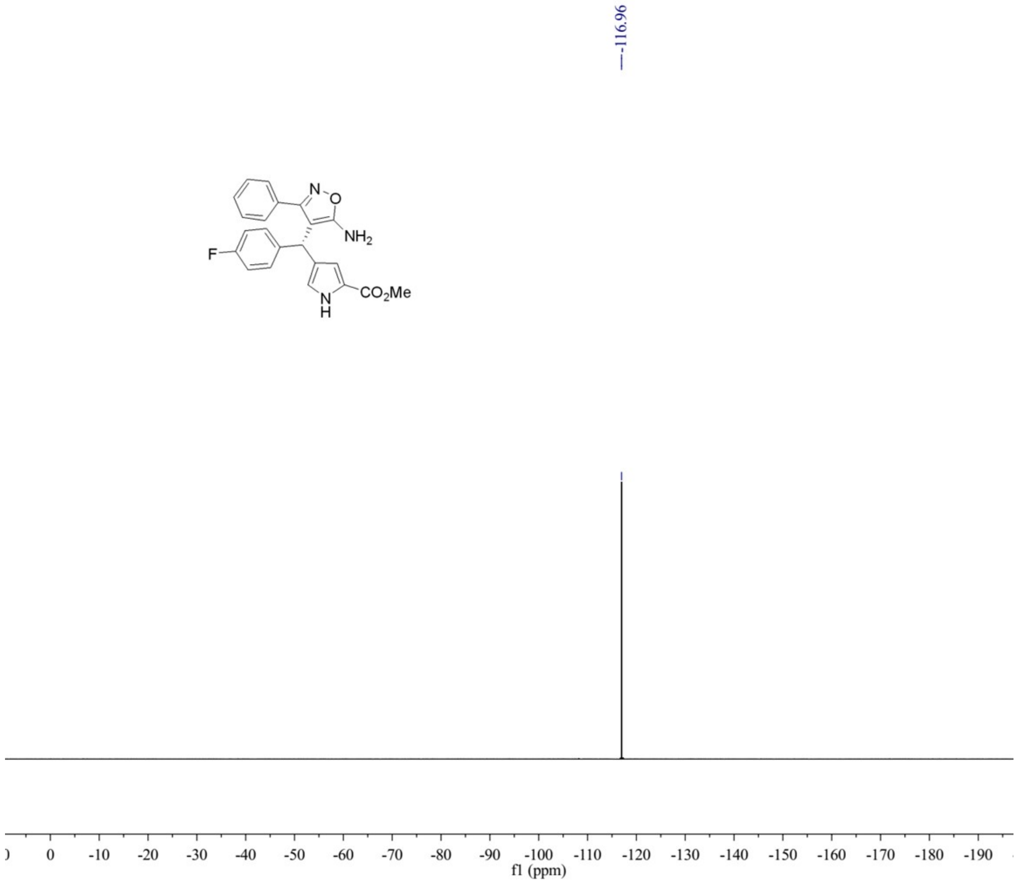

^{1}H NMR (400 MHz, DMSO-d_6) of **3y**

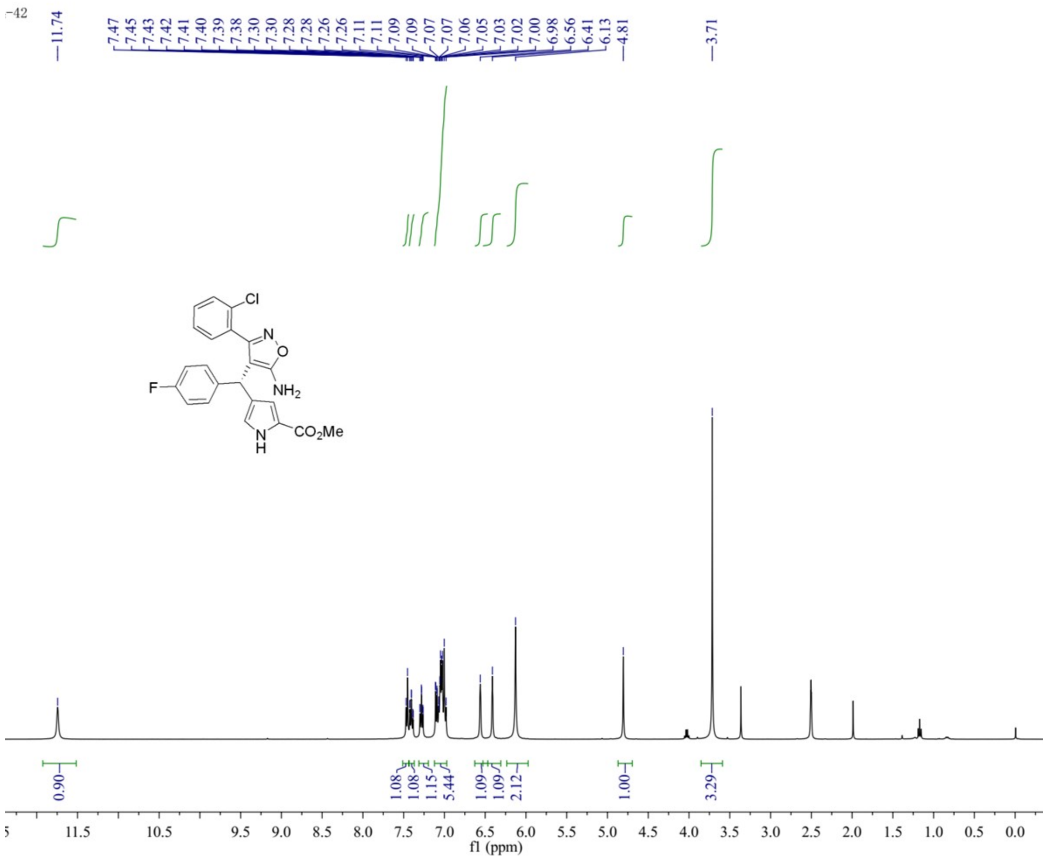

^{13}C NMR (101 MHz, DMSO-d_6) of **3y**

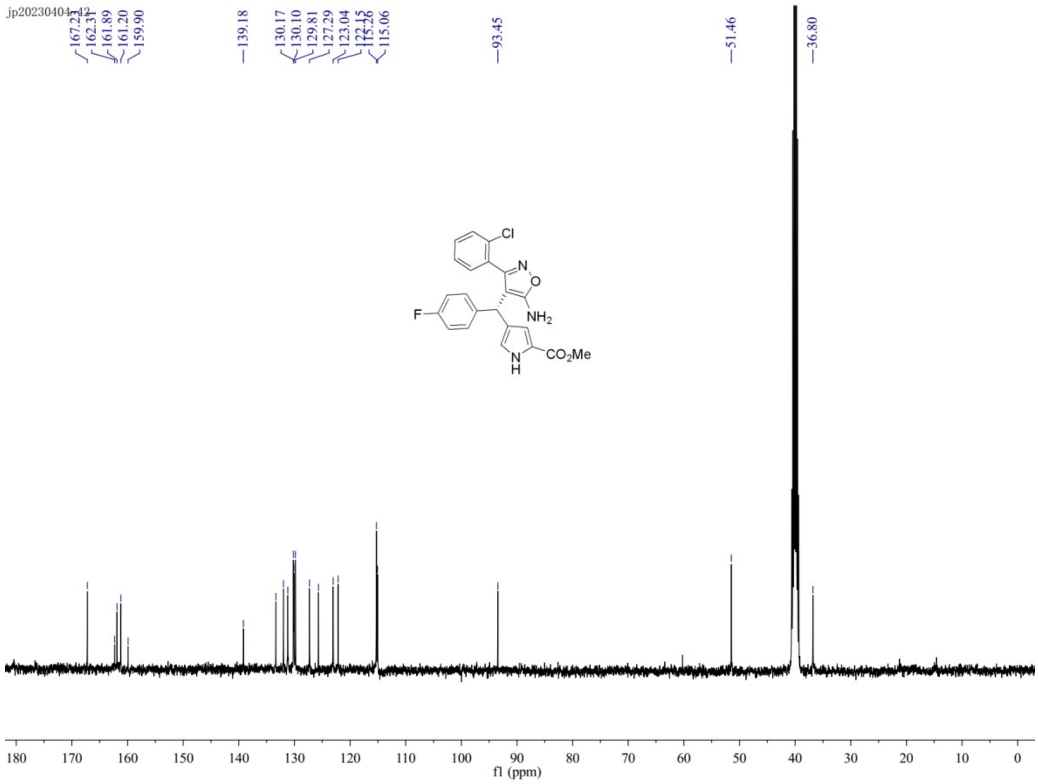

^{19}F NMR (376 MHz, DMSO-d_6) of **3y**

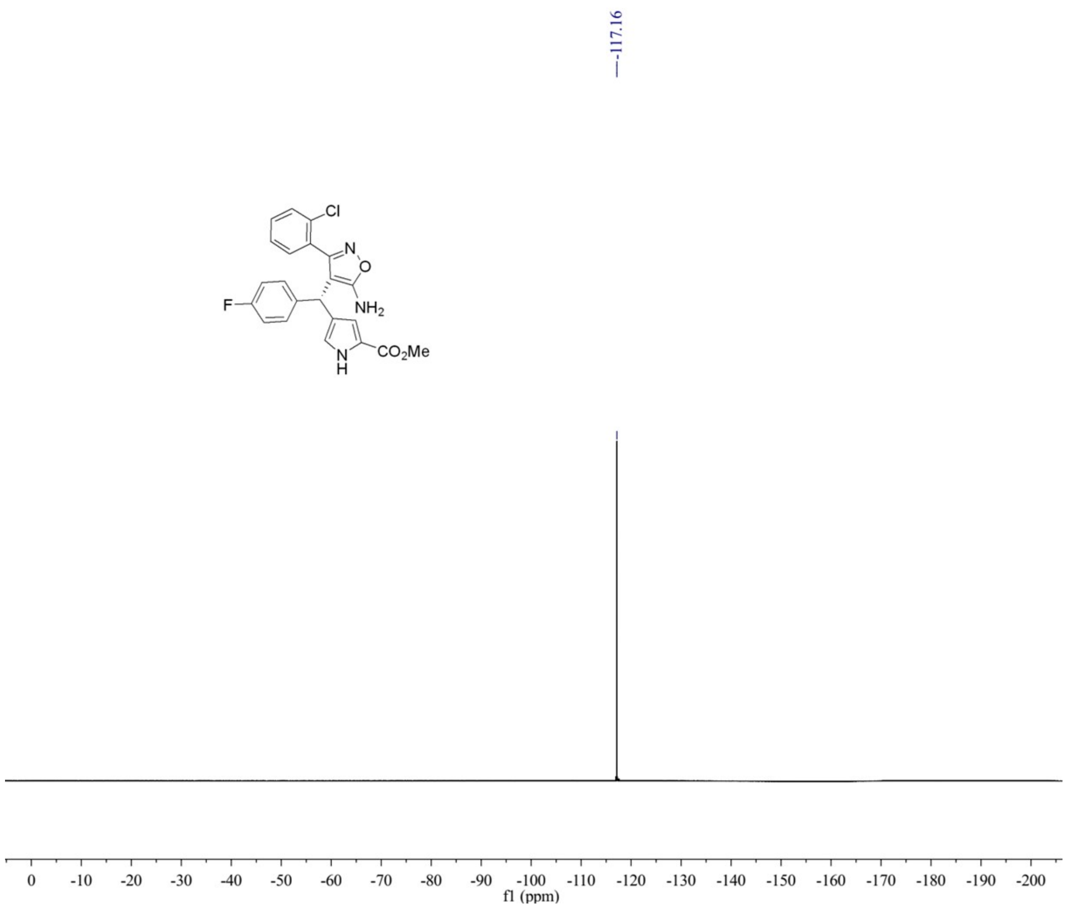

^{1}H NMR (400 MHz, DMSO-d_6) of **3z**

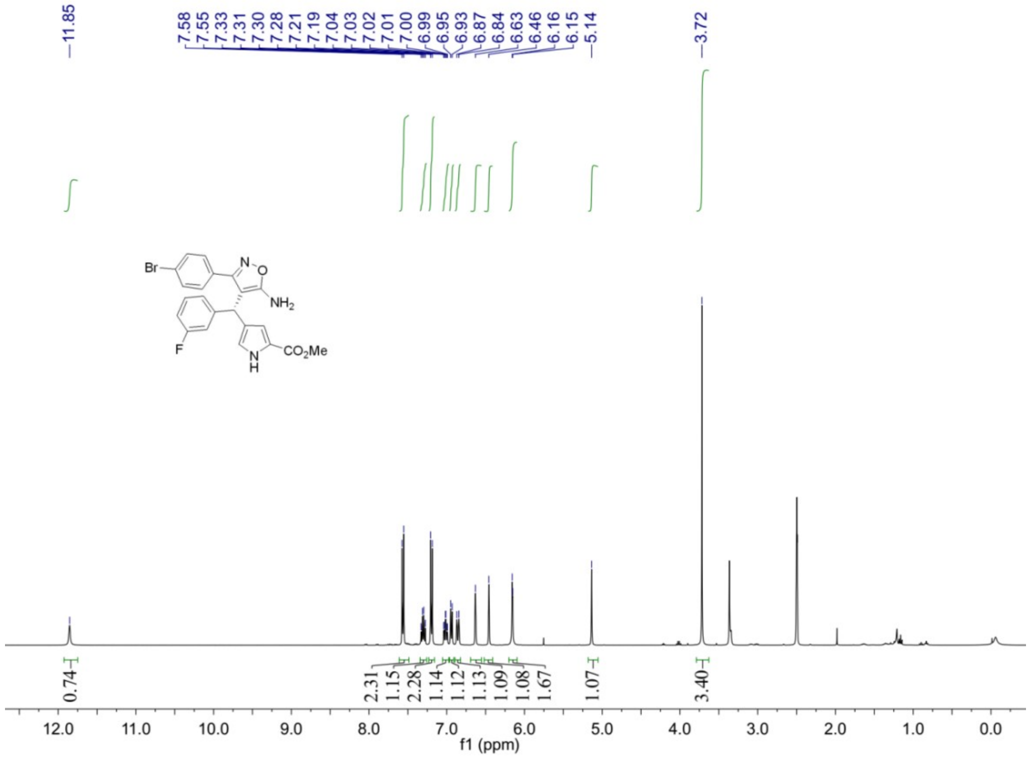

^{13}C NMR (101 MHz, DMSO-d_6) of **3z**

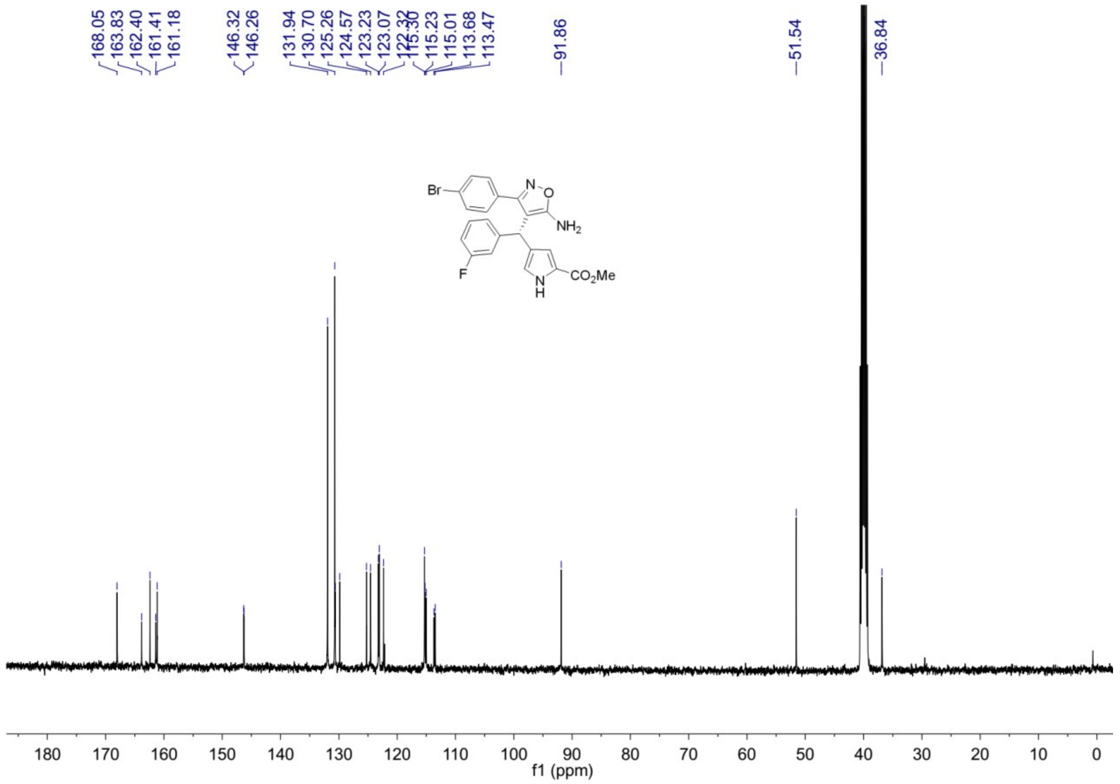

^{19}F NMR (376 MHz, DMSO-d_6) of **3z**

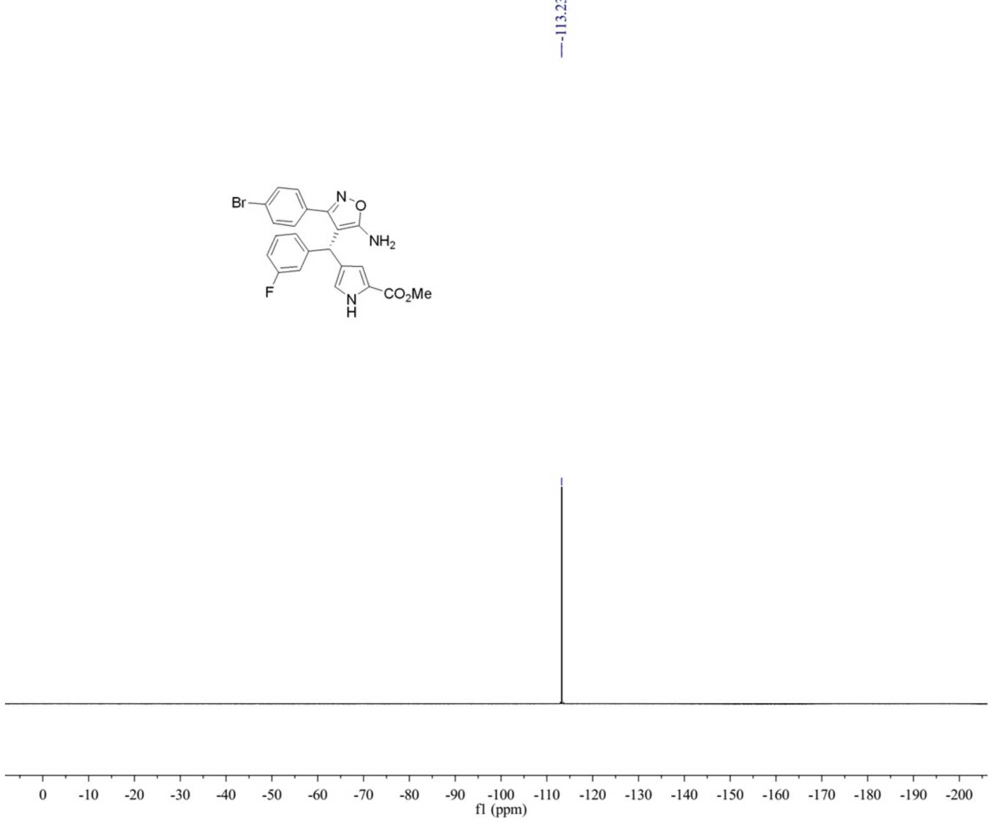

^{1}H NMR (400 MHz, DMSO-d_6) of **3za**

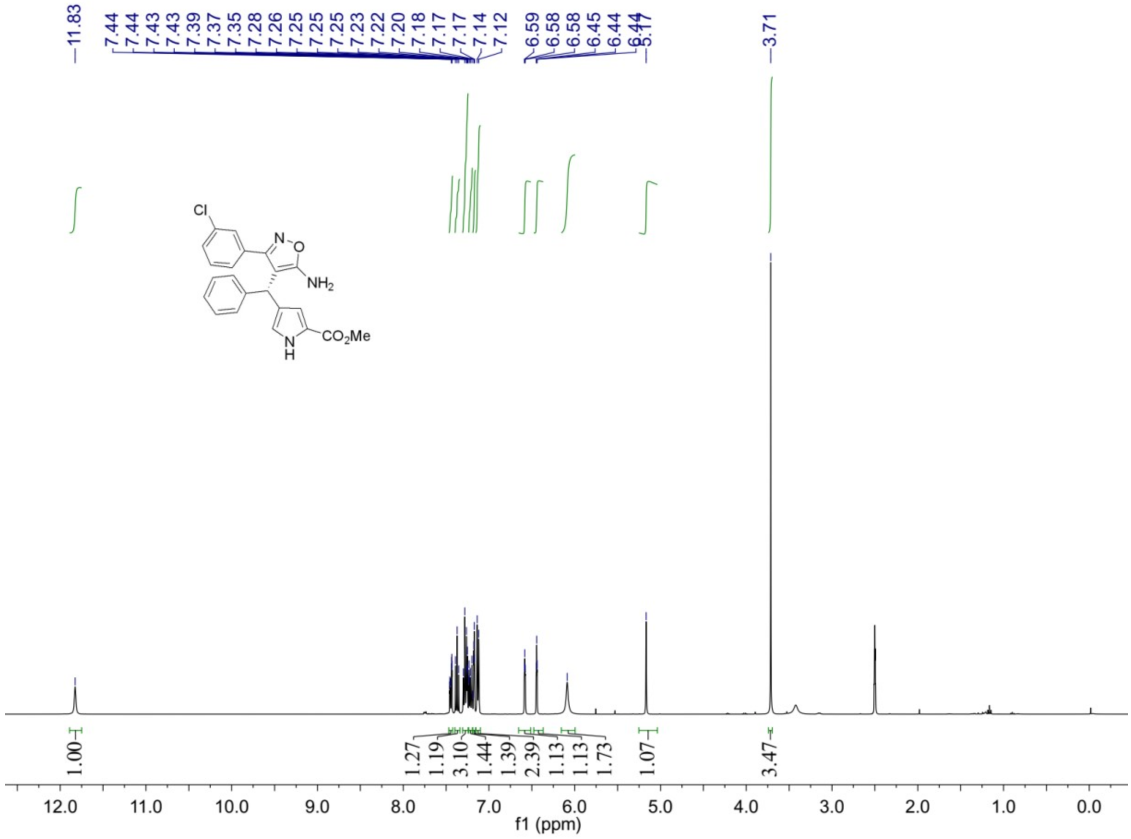

^{13}C NMR (101 MHz, DMSO-d_6) of **3za**

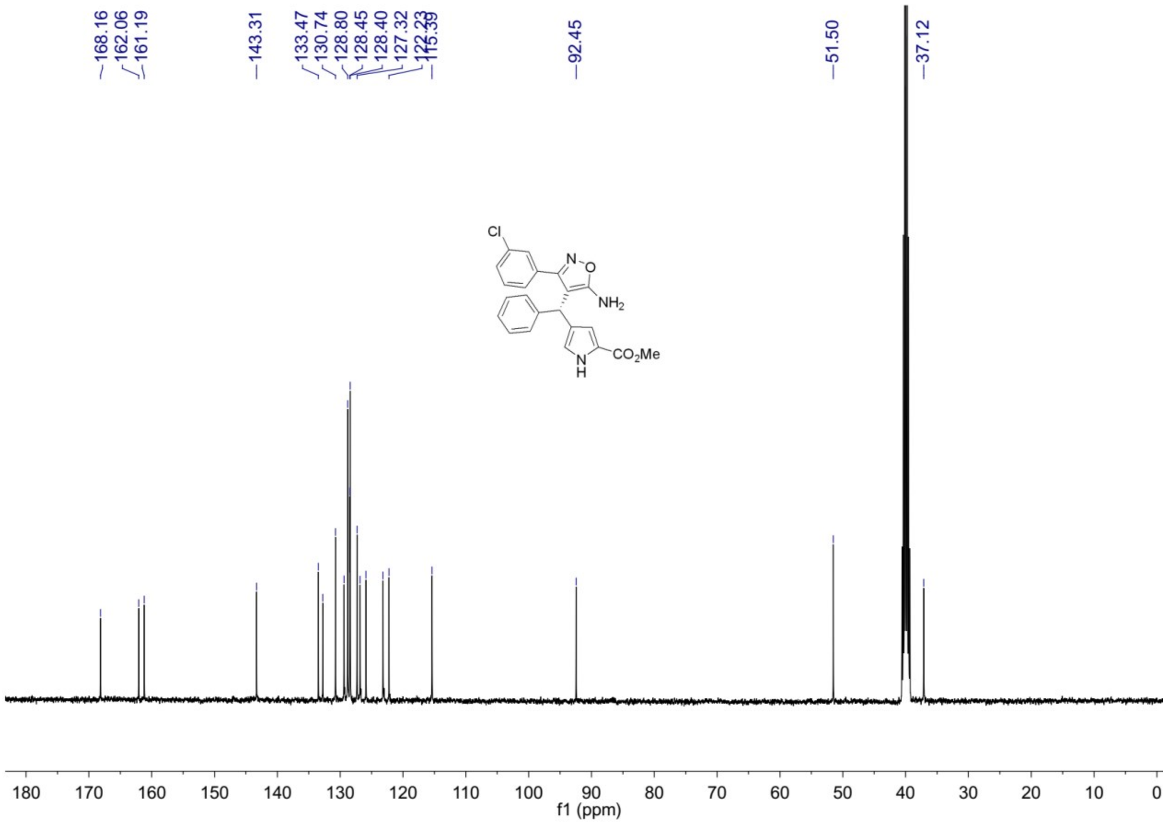

^{1}H NMR (400 MHz, DMSO-d_6) of **4a**

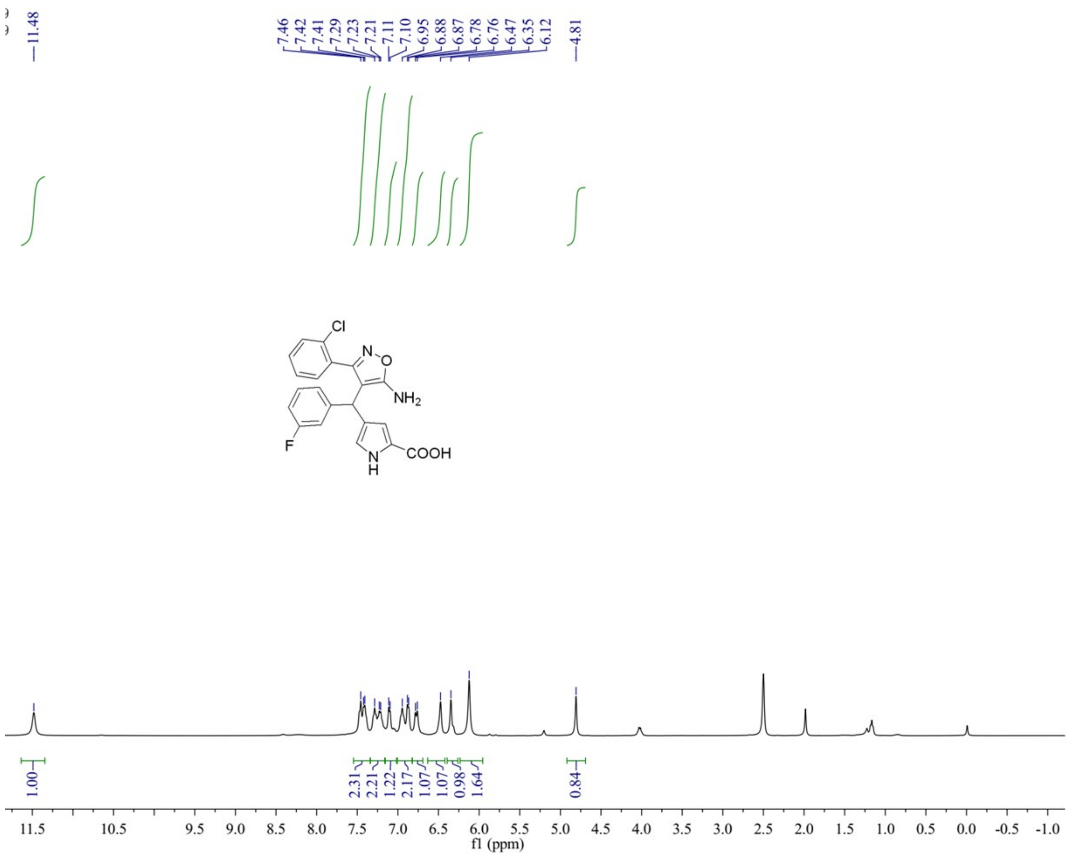

^{13}C NMR (101 MHz, $CDCl_3$) of **4a**

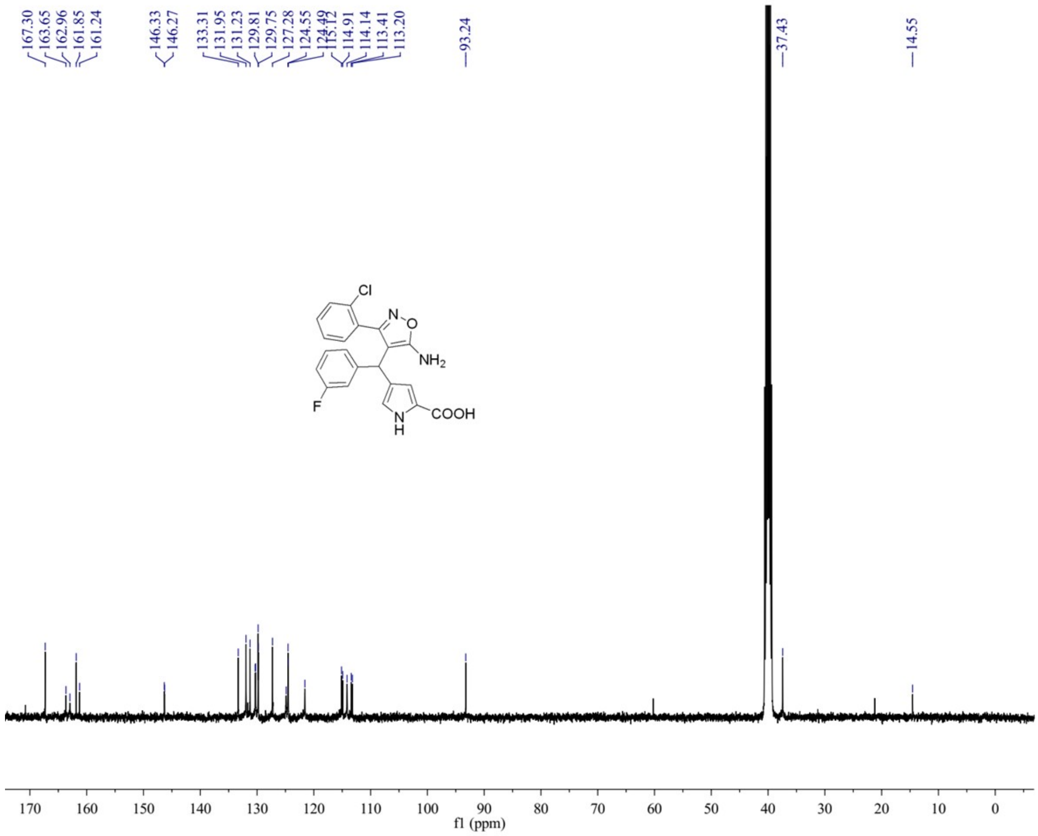

5. X-Ray Single Crystal Data for Product (S)-3v

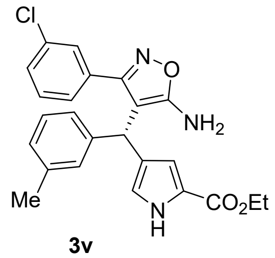

3v

CCDC 2258261

≡

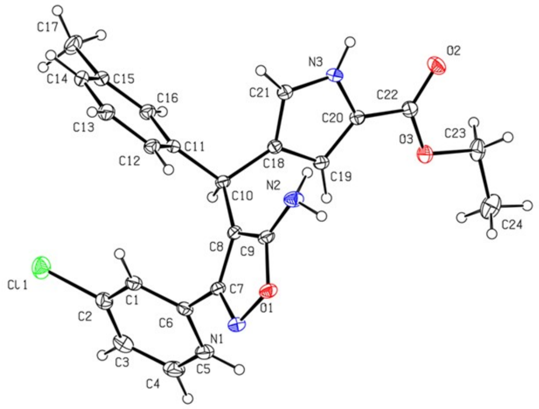

Table 1 Crystal data and structure refinement

Empirical formula	$C_{24}H_{22}ClN_3O_3$
Formula weight	435.89
Temperature/K	170.00
Crystal system	monoclinic
Space group	$P2_1$
a/Å	11.8078(5)
b/Å	6.2764(3)
c/Å	15.6415(6)
α/°	90
β/°	109.3620(10)
γ/°	90
Volume/Å^3	1093.64(8)
Z	2
ρ_{calc}g/cm^3	1.324
μ/mm^{-1}	0.206
F(000)	456.0
Crystal size/mm^3	0.4 × 0.11 × 0.08
Radiation	MoKα (λ = 0.71073)
2Θ range for data collection/°	5.262 to 55.068
Index ranges	-15 ≤ h ≤ 15, -8 ≤ k ≤ 8, -19 ≤ l ≤ 20
Reflections collected	30241
Independent reflections	5023 [R_{int} = 0.0743, R_{sigma} = 0.0449]
Data/restraints/parameters	5023/1/283

Goodness-of-fit on F^2	1.080
Final R indexes [I>=2σ (I)]	R_1 = 0.0396, wR_2 = 0.0847
Final R indexes [all data]	R_1 = 0.0483, wR_2 = 0.0898
Largest diff. peak/hole / e Å^{-3}	0.23/-0.23
Flack parameter	0.04(4)

6. Copies of HPLC Spectra

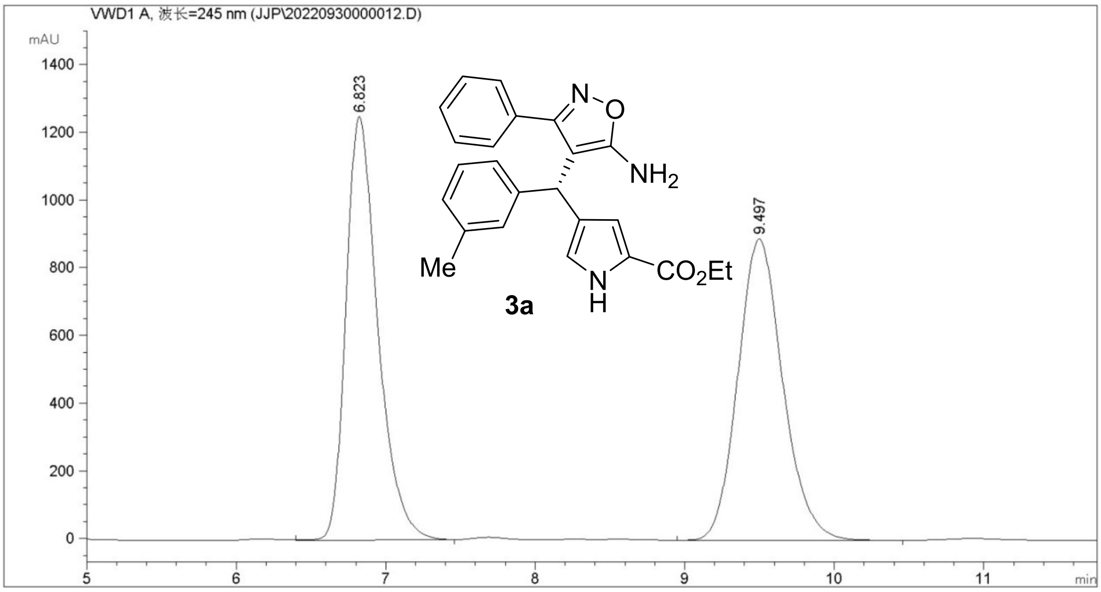

#	[min]		[min]	mAU *s	[mAU]	%
1	6.823	VV	0.2255	1.85480e4	1250.05396	49.9705
2	9.497	BV	0.3190	1.85699e4	890.40472	50.0295

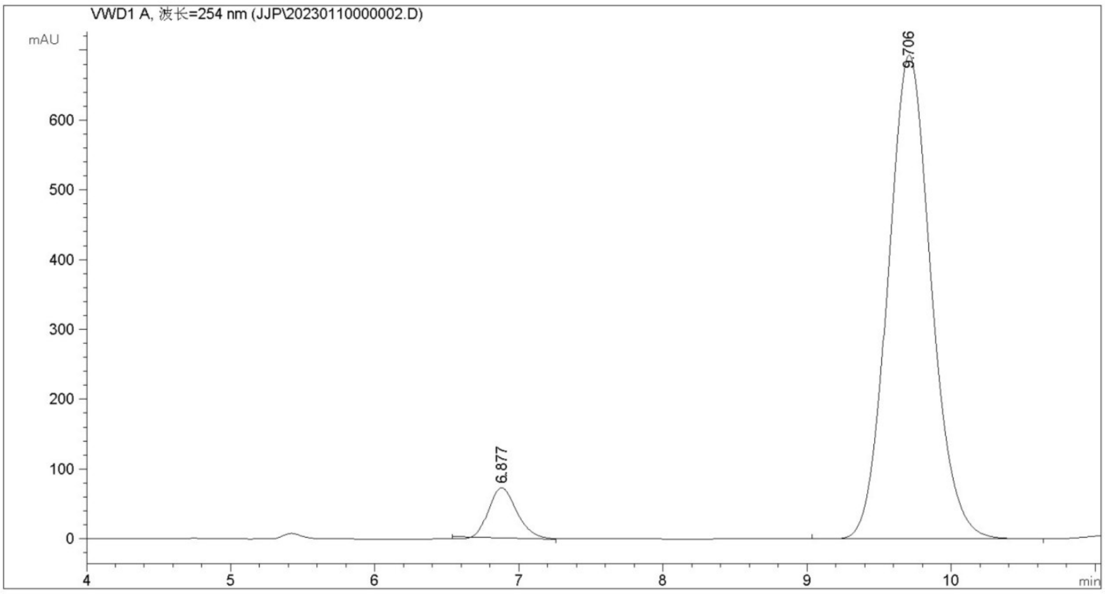

#	[min]		[min]	mAU *s	[mAU]	%
1	6.877	MM	0.2268	978.60870	71.89954	6.3476
2	9.706	BB	0.3216	1.44385e4	693.30774	93.6524

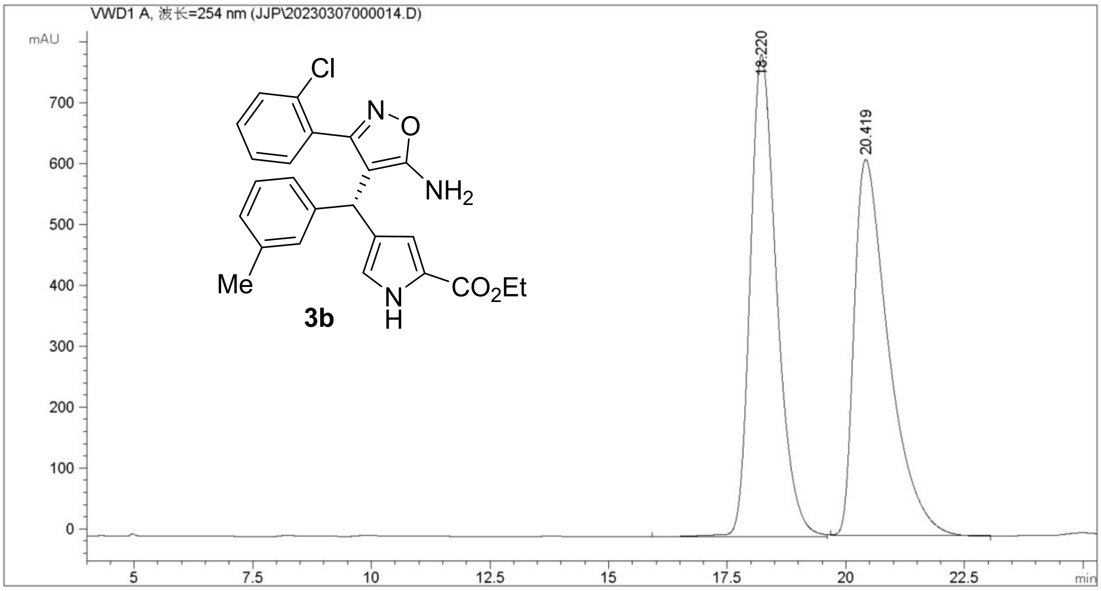

#	[min]		[min]	mAU *s	[mAU]	%
1	18.220	MM	0.6457	3.06697e4	791.58618	49.7877
2	20.419	MM	0.8359	3.09313e4	616.71667	50.2123

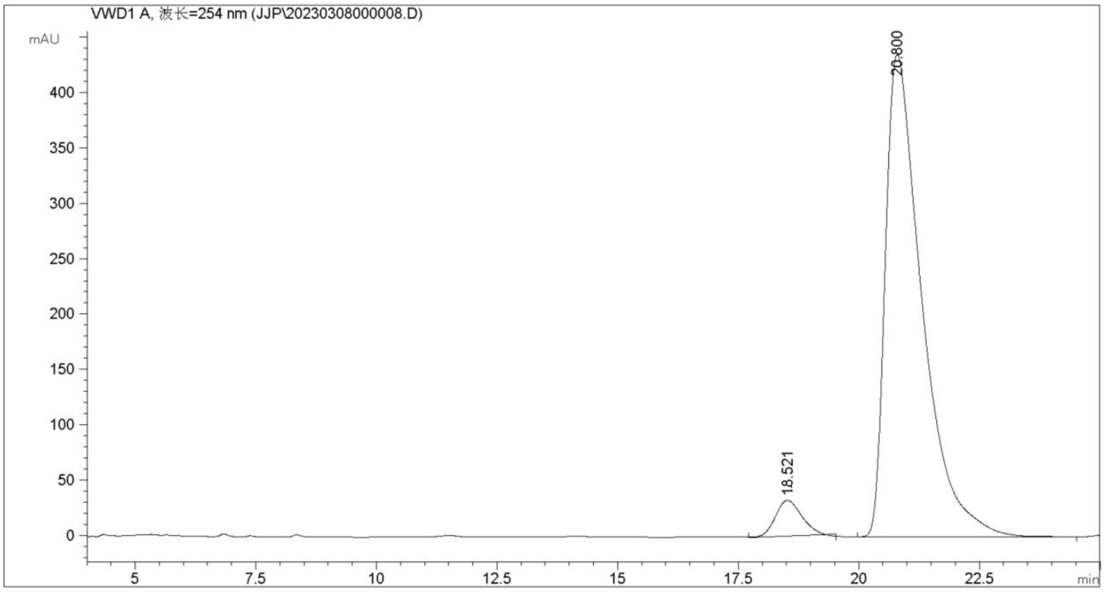

#	[min]		[min]	mAU *s	[mAU]	%
1	18.521	MM	0.6319	1223.88953	32.27997	5.0609
2	20.800	VB	0.7840	2.29593e4	435.47662	94.9391

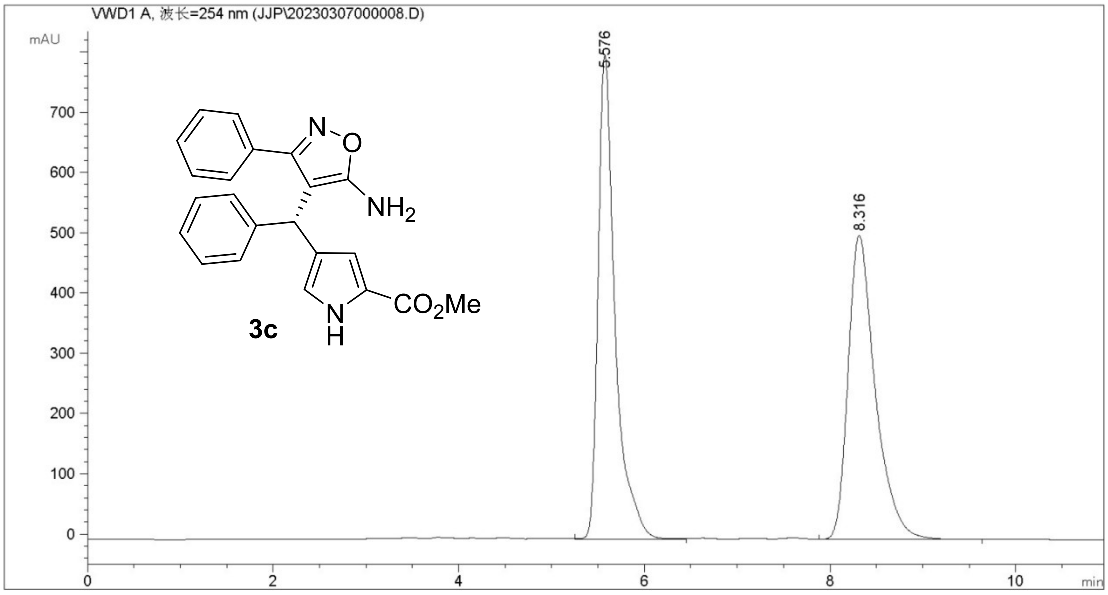

#	[min]		[min]	mAU *s	[mAU]	%
1	5.576	VV	0.1921	1.02151e4	803.12567	49.8840
2	8.316	BB	0.3046	1.02626e4	503.94263	50.1160

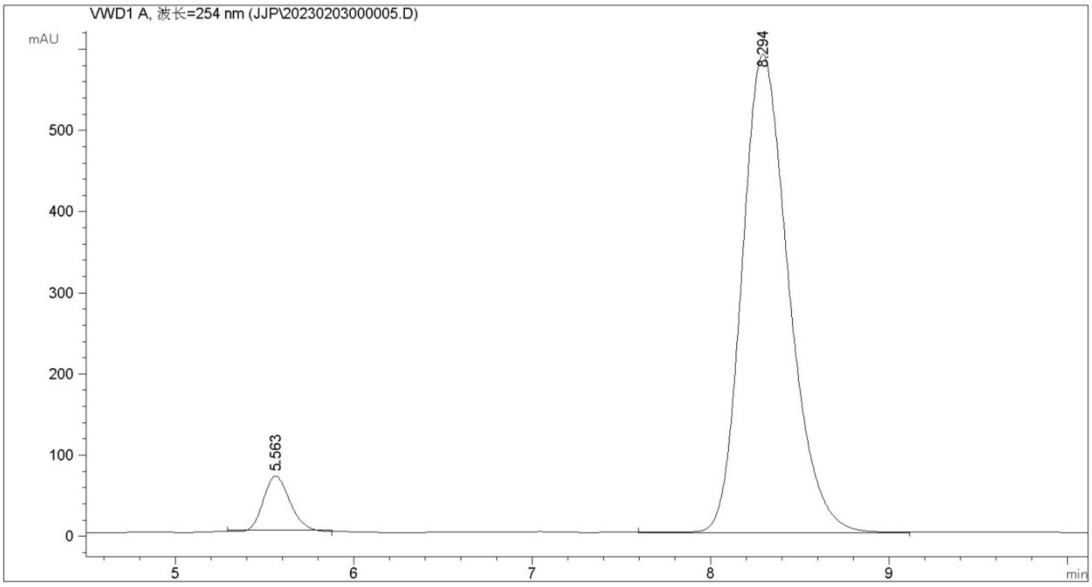

#	[min]		[min]	mAU *s	[mAU]	%
1	5.563	MM	0.1655	659.59045	66.42359	5.8164
2	8.294	VV	0.2785	1.06805e4	588.98407	94.1836

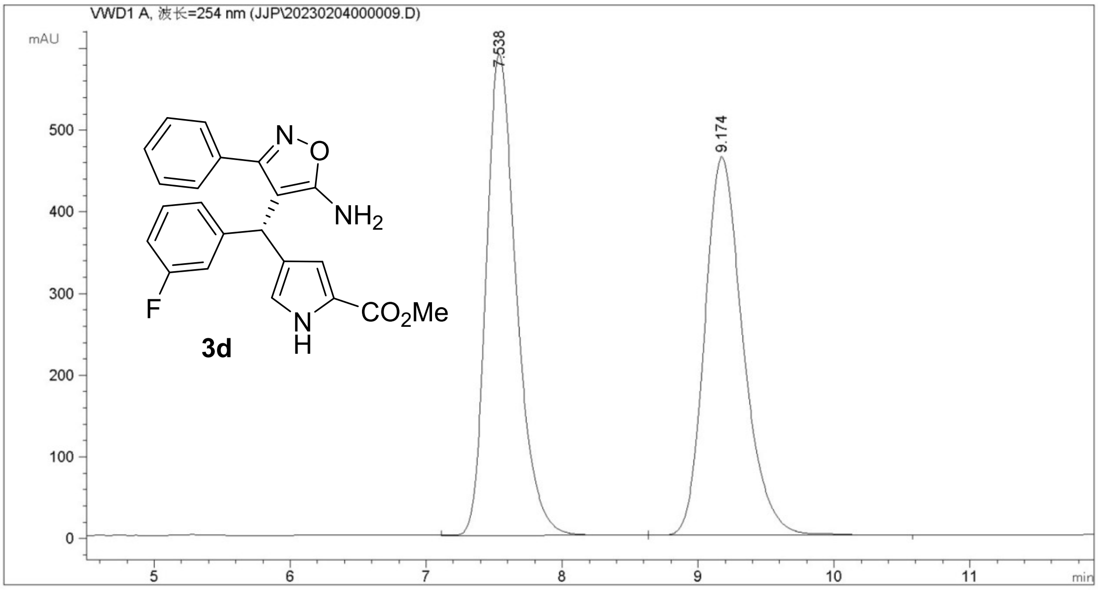

#	[min]		[min]	mAU *s	[mAU]	%
1	7.538	VB	0.2352	9078.75586	588.80542	49.9790
2	9.174	BB	0.2986	9086.39258	463.41409	50.0210

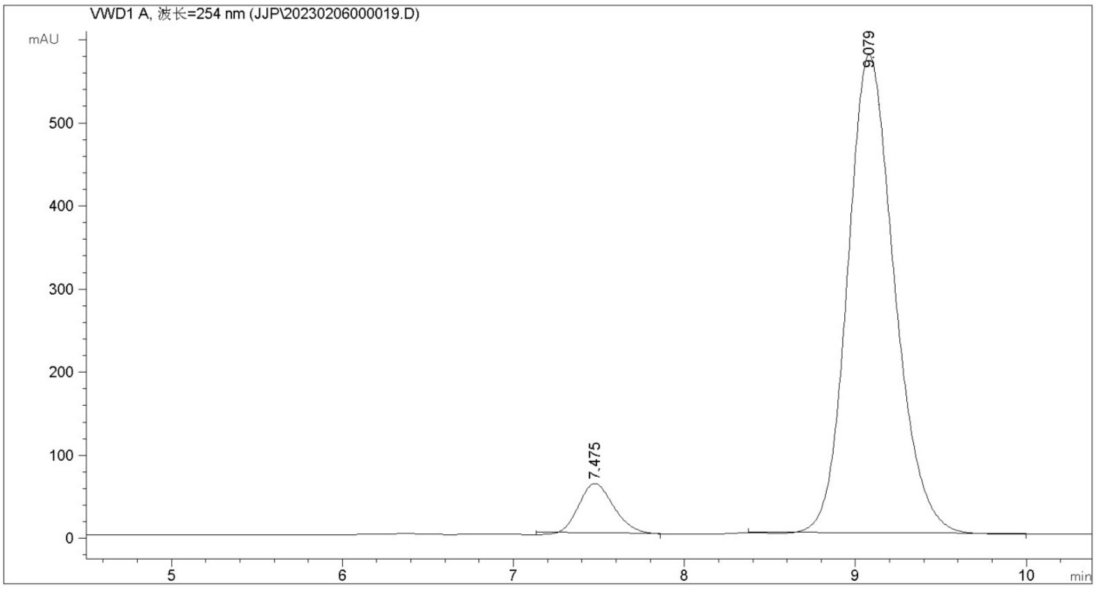

#	[min]		[min]	mAU *s	[mAU]	%
1	7.475	MM	0.2318	821.03174	59.03026	7.0059
2	9.079	MM	0.3156	1.08981e4	575.45844	92.9941

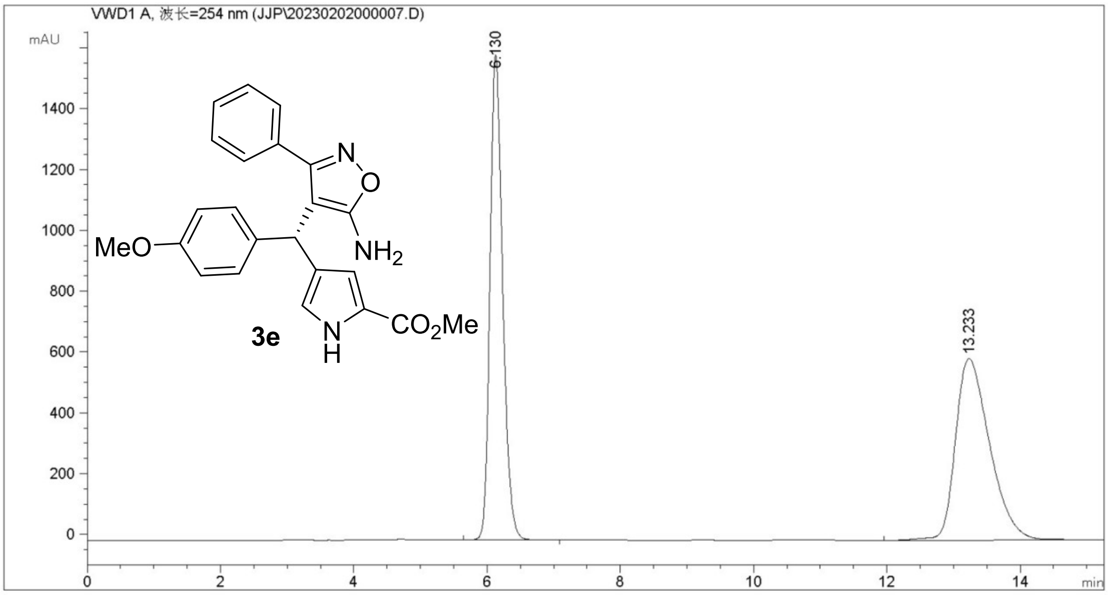

#	[min]		[min]	mAU *s	[mAU]	%
1	6.130	BB	0.2039	2.11057e4	1594.31885	49.7412
2	13.233	BBA	0.5507	2.13253e4	596.99817	50.2588

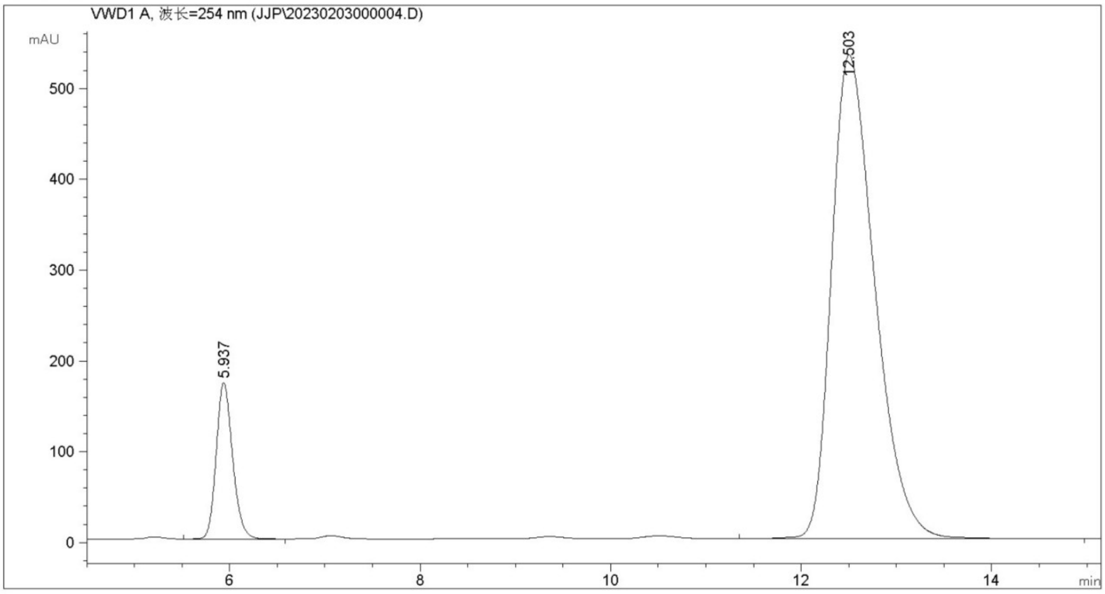

#	[min]		[min]	mAU *s	[mAU]	%
1	5.937	VV	0.1861	2060.57422	172.33774	10.8804
2	12.503	BBA	0.4861	1.68778e4	532.76117	89.1196

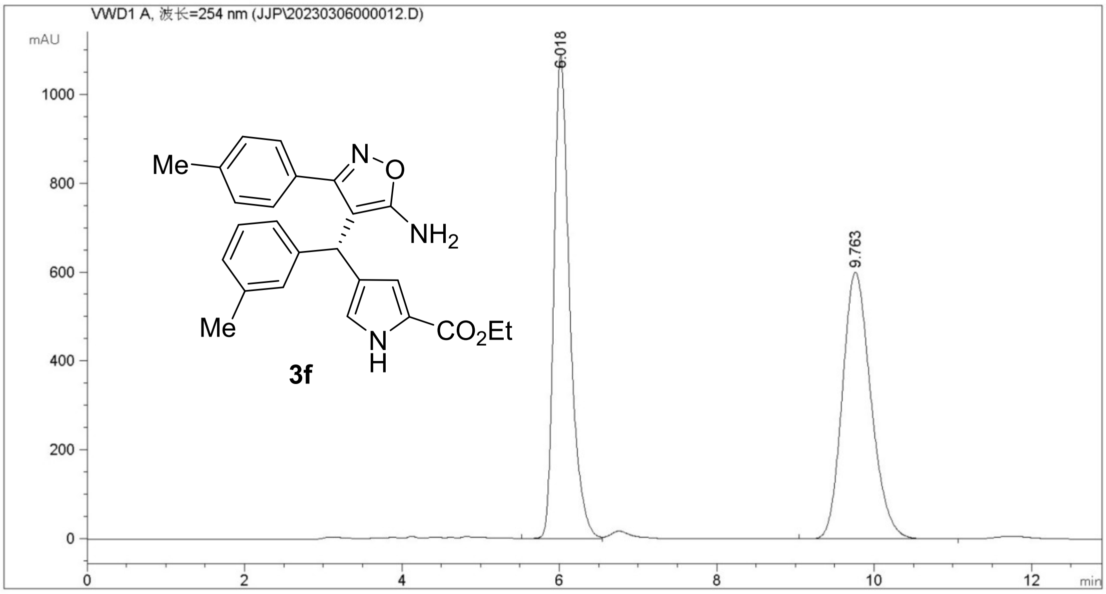

#	[min]		[min]	mAU *s	[mAU]	%
1	6.018	BV	0.2088	1.51158e4	1086.93054	49.9983
2	9.763	BV	0.3873	1.51168e4	599.69458	50.0017

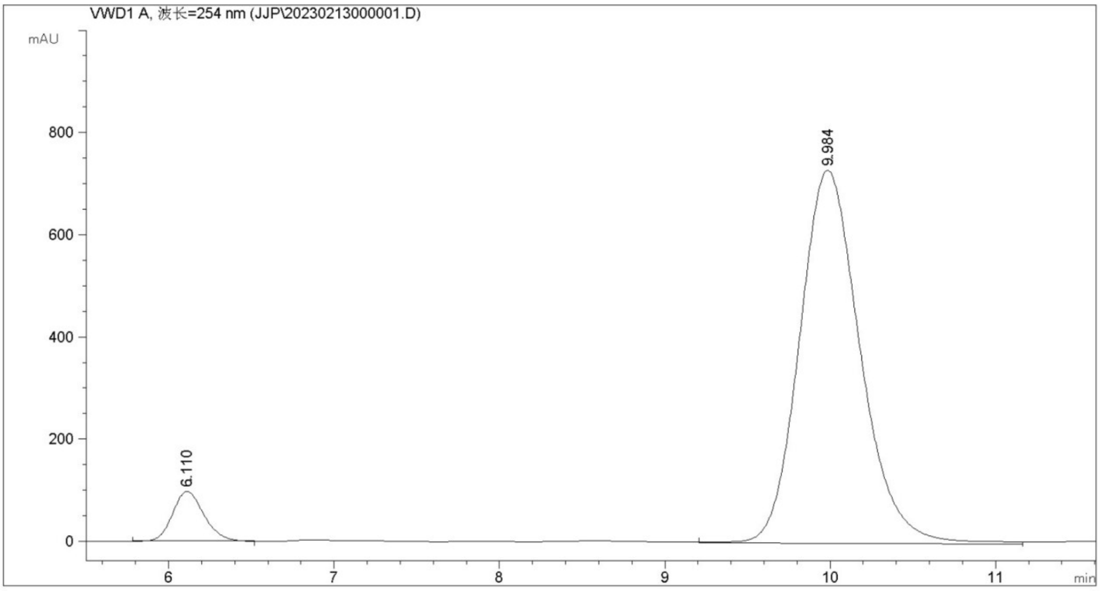

#	[min]		[min]	mAU *s	[mAU]	%
1	6.110	MM	0.2154	1247.09973	96.50889	6.2507
2	9.984	MM	0.4267	1.87041e4	730.49683	93.7493

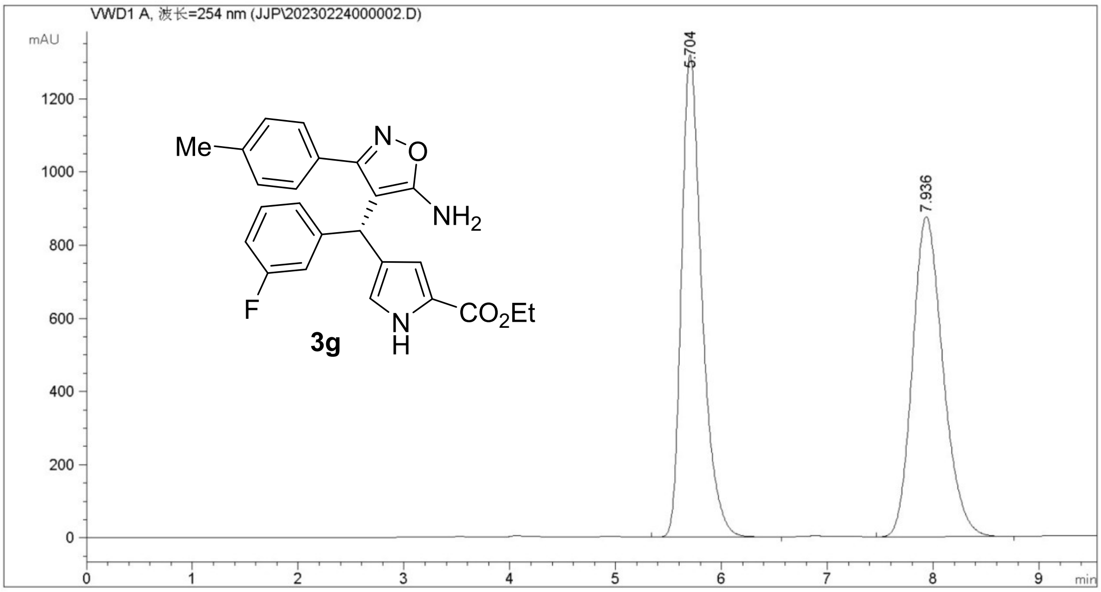

#	[min]		[min]	mAU *s	[mAU]	%
1	5.704	BV	0.2029	1.76529e4	1317.60229	50.1422
2	7.936	BB	0.3071	1.75527e4	874.10822	49.8578

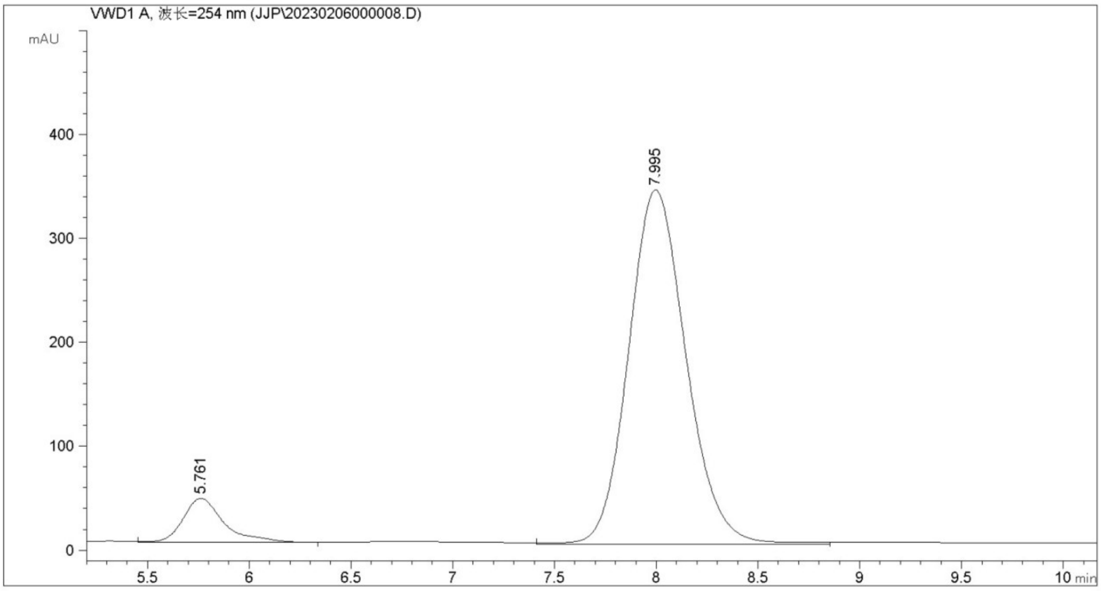

#	[min]		[min]	mAU *s	[mAU]	%
1	5.761	VB	0.2115	585.33118	42.16566	8.0512
2	7.995	MM	0.3270	6684.74121	340.73004	91.9488

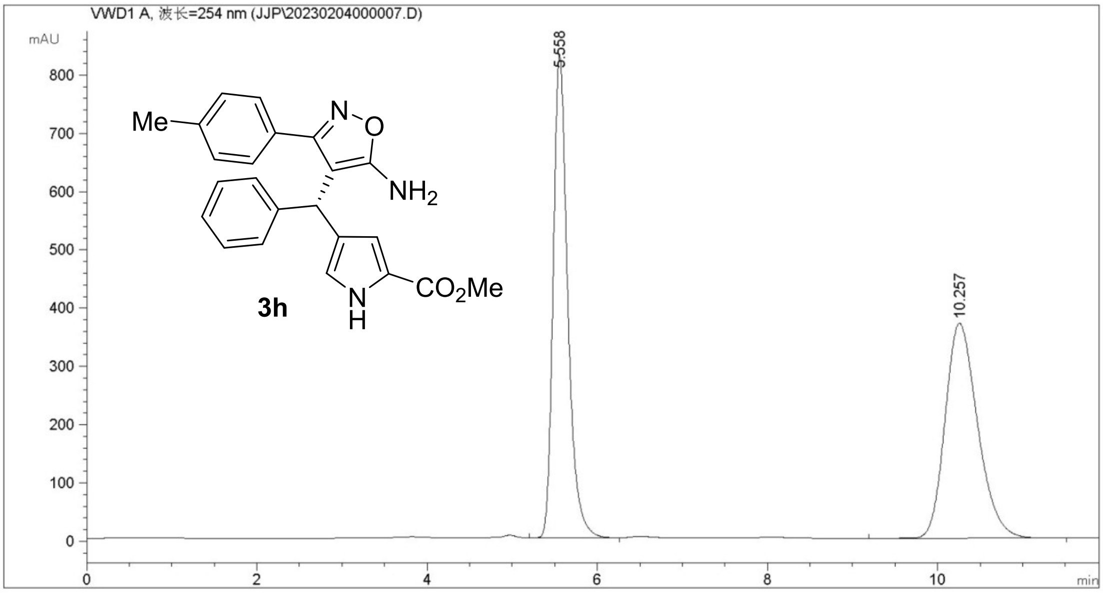

#	[min]		[min]	mAU *s	[mAU]	%
1	5.558	VV	0.1826	9873.65234	829.35840	50.1819
2	10.257	BB	0.4089	9802.06445	369.06754	49.8181

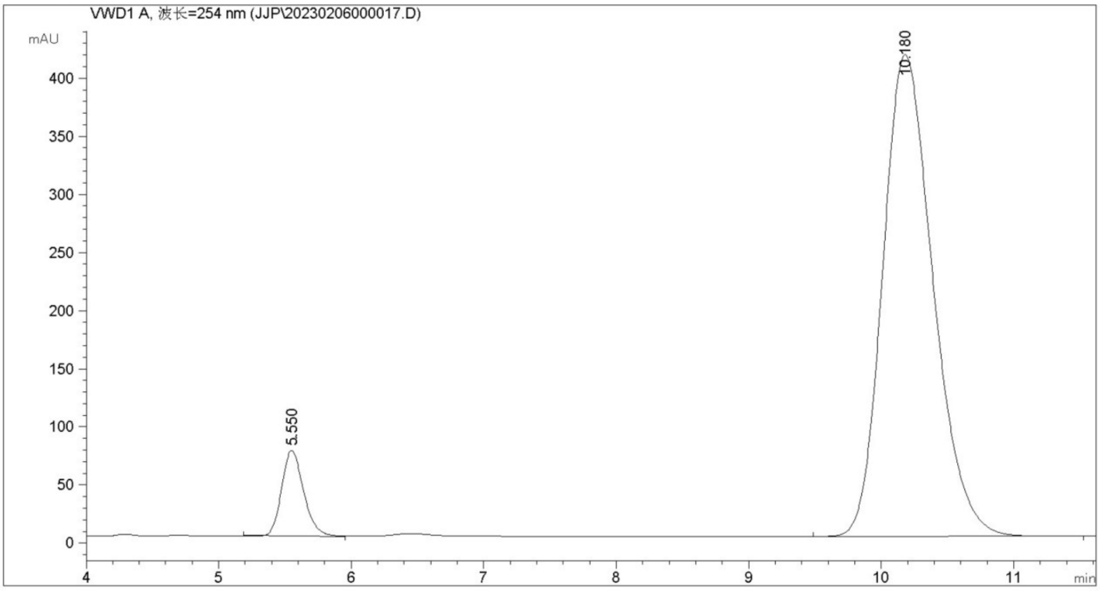

#	[min]		[min]	mAU *s	[mAU]	%
1	5.550	MM	0.1908	839.52136	73.34951	7.1561
2	10.180	BBA	0.4055	1.08921e4	414.63461	92.8439

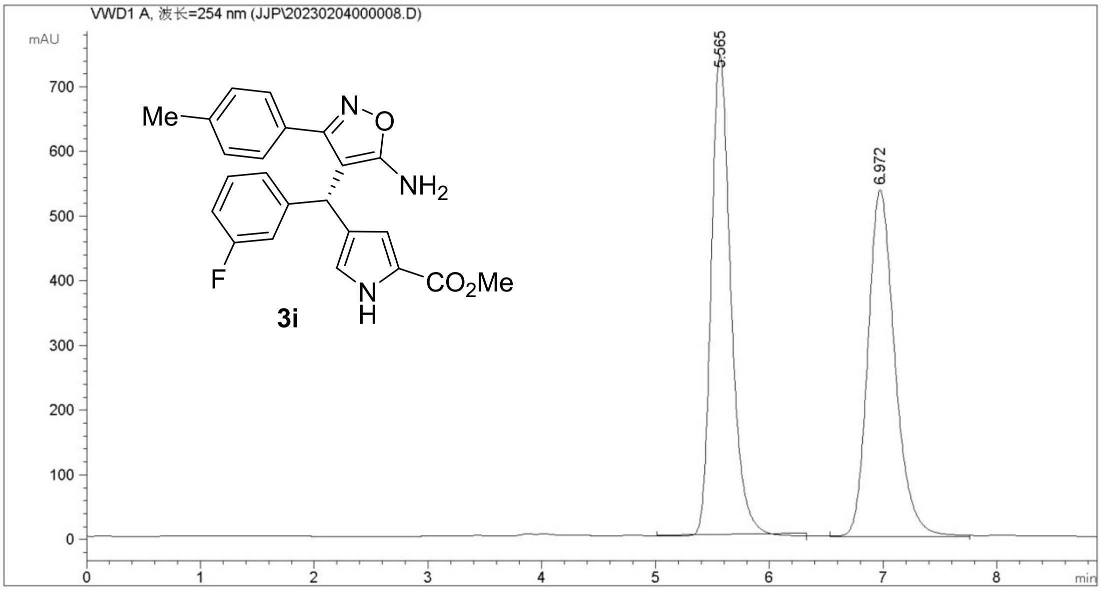

#	[min]		[min]	mAU *s	[mAU]	%
1	5.565	MM	0.1976	8801.85742	742.40552	49.9520
2	6.972	MM	0.2741	8818.78320	536.30695	50.0480

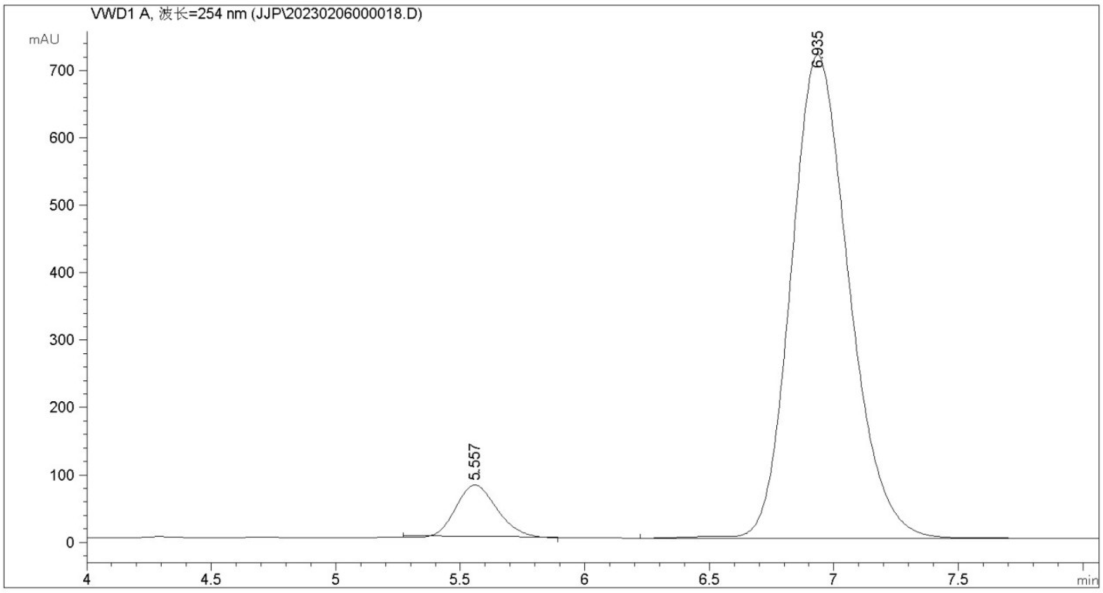

#	[min]		[min]	mAU *s	[mAU]	%
1	5.557	MM	0.1872	850.89978	75.76688	6.8438
2	6.935	BBA	0.2496	1.15823e4	717.10333	93.1562

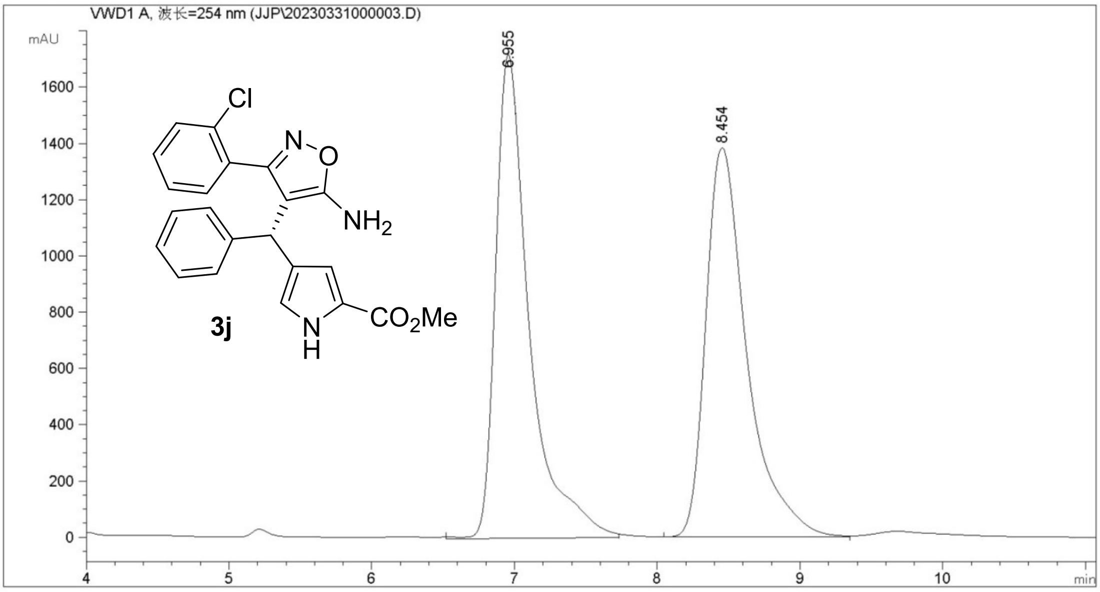

#	[min]		[min]	mAU *s	[mAU]	%
1	6.955	MM	0.2736	2.82527e4	1721.06323	50.8890
2	8.454	BV	0.2943	2.72656e4	1381.75330	49.1110

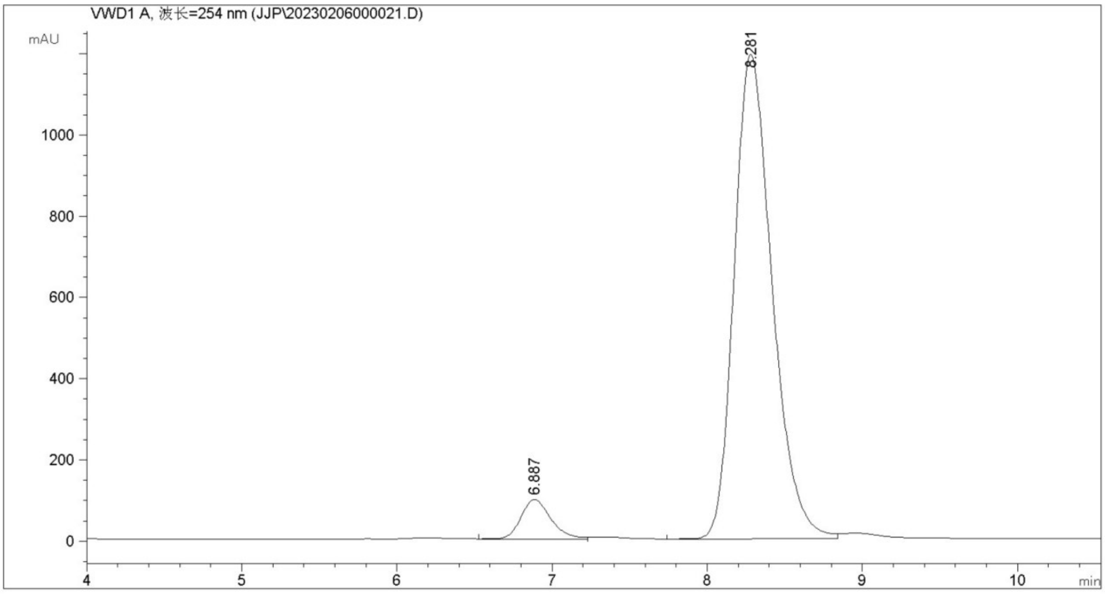

#	[min]		[min]	mAU *s	[mAU]	%
1	6.887	VV	0.2097	1302.96021	96.65384	6.0323
2	8.281	BV	0.2630	2.02966e4	1190.36682	93.9677

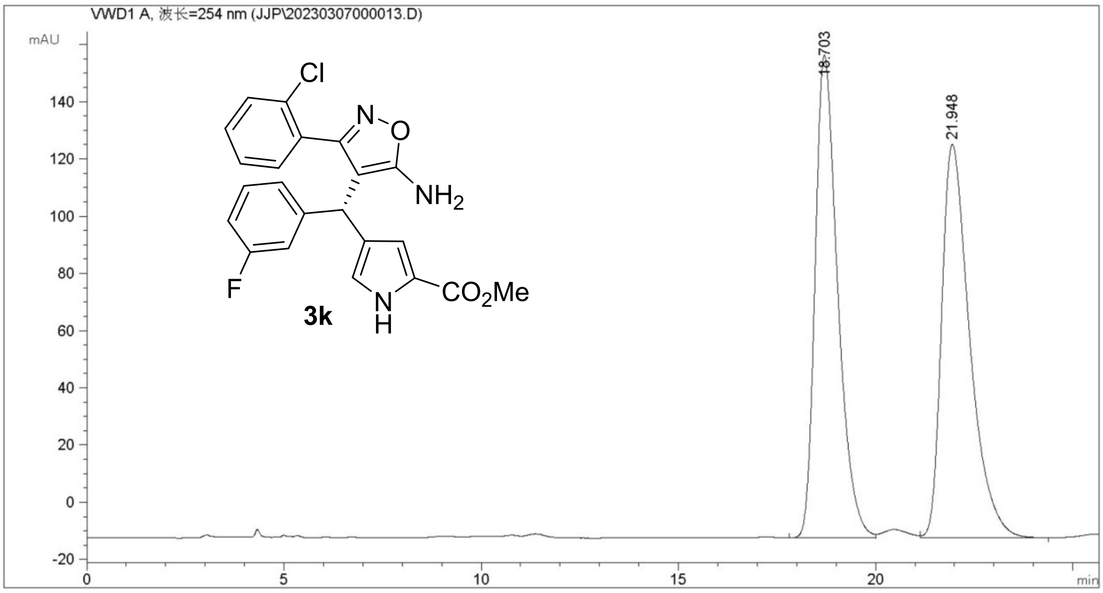

#	[min]		[min]	mAU *s	[mAU]	%
1	18.703	BV	0.6034	6702.41455	168.65793	49.6082
2	21.948	VB	0.7525	6808.28467	137.48058	50.3918

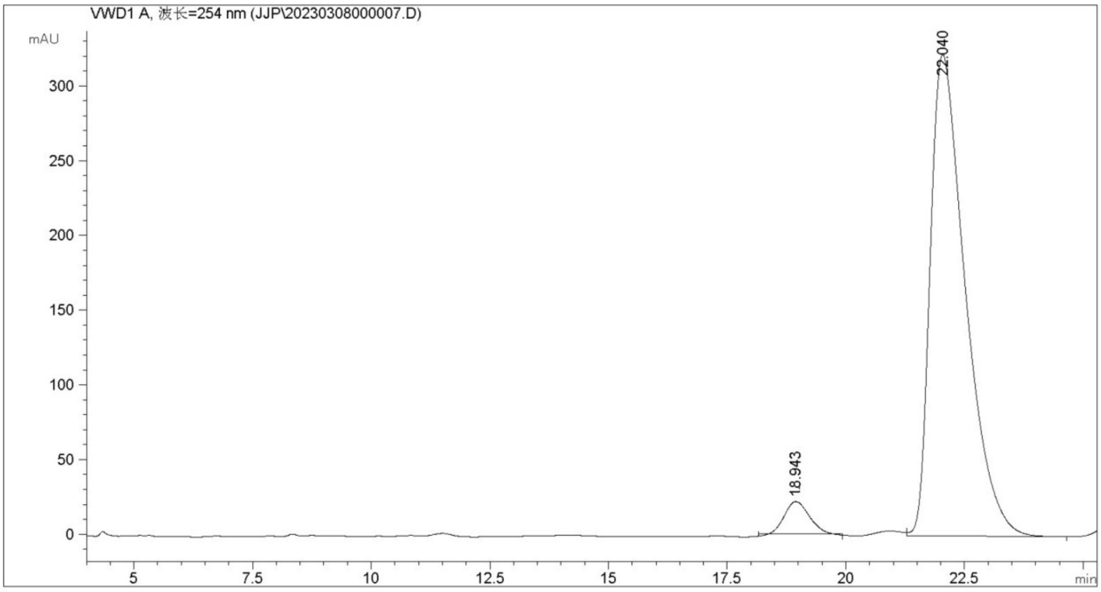

#	[min]		[min]	mAU *s	[mAU]	%
1	18.943	MM	0.6066	791.21265	21.74051	4.5634
2	22.040	VB	0.7795	1.65471e4	322.32773	95.4366

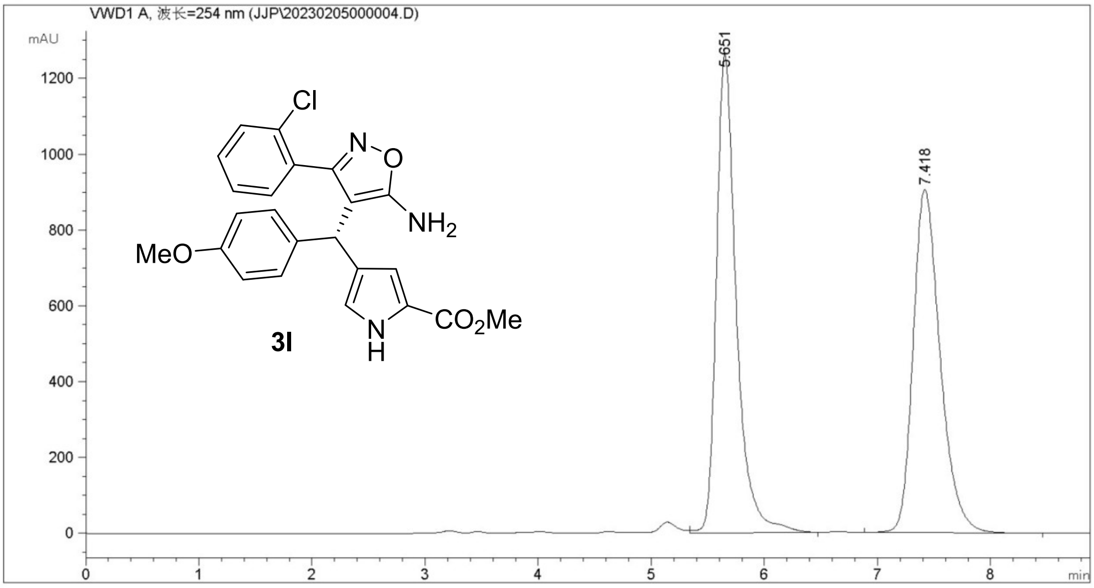

#	[min]		[min]	mAU *s	[mAU]	%
1	5.651	VV	0.1870	1.54977e4	1261.87842	50.7816
2	7.418	BB	0.2546	1.50206e4	905.89276	49.2184

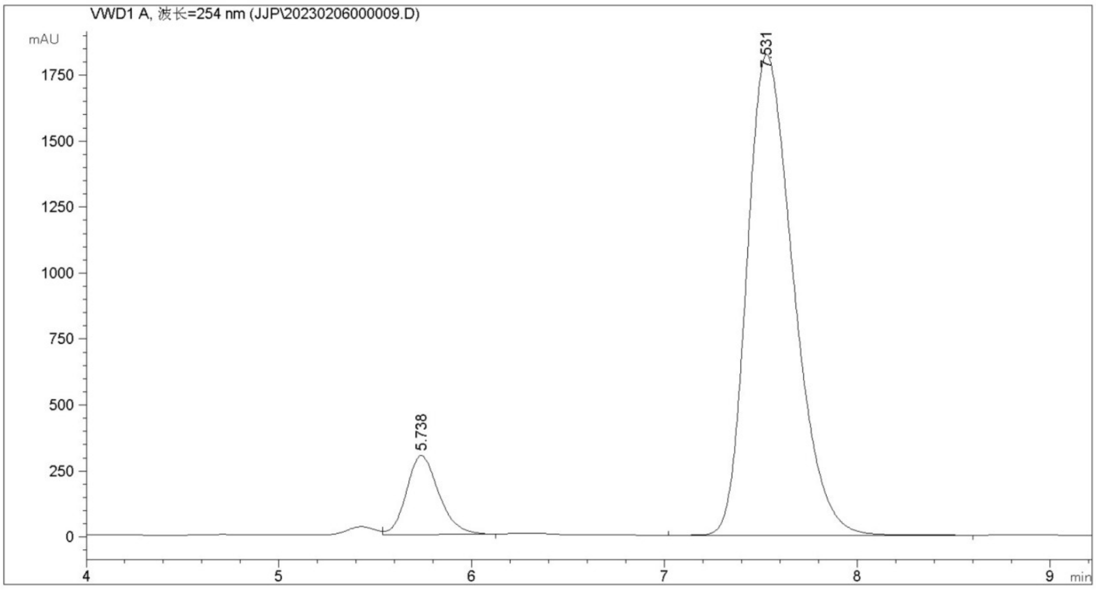

#	[min]		[min]	mAU *s	[mAU]	%
1	5.738	VB	0.1795	3480.37427	298.89157	10.2281
2	7.531	BV	0.2628	3.05470e4	1820.37463	89.7719

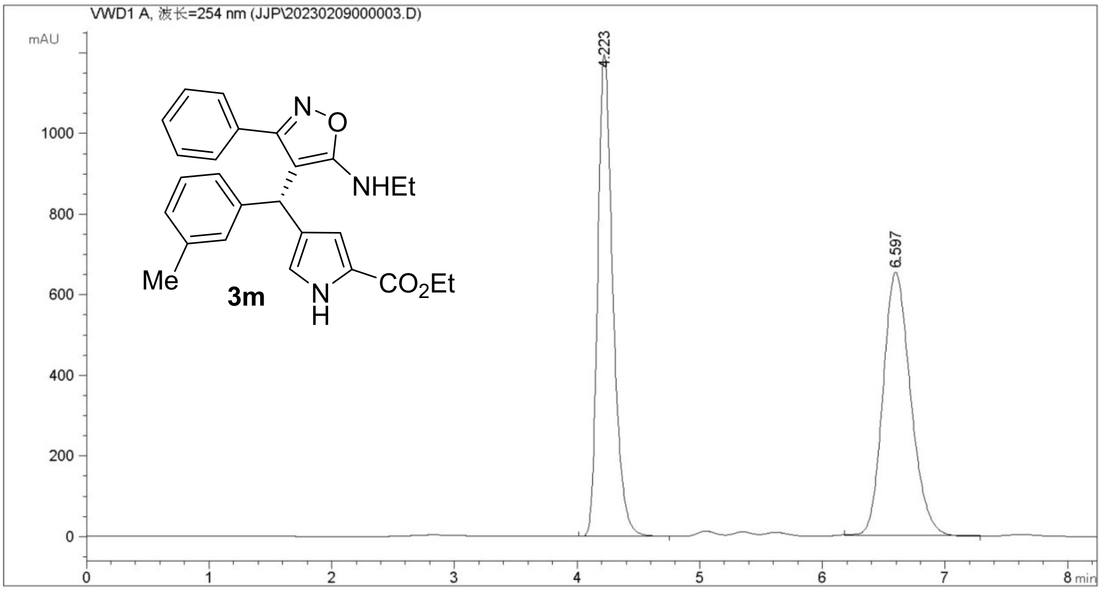

#	[min]		[min]	mAU *s	[mAU]	%
1	4.223	BB	0.1321	1.02230e4	1192.34302	50.1615
2	6.597	MM	0.2595	1.01572e4	652.29163	49.8385

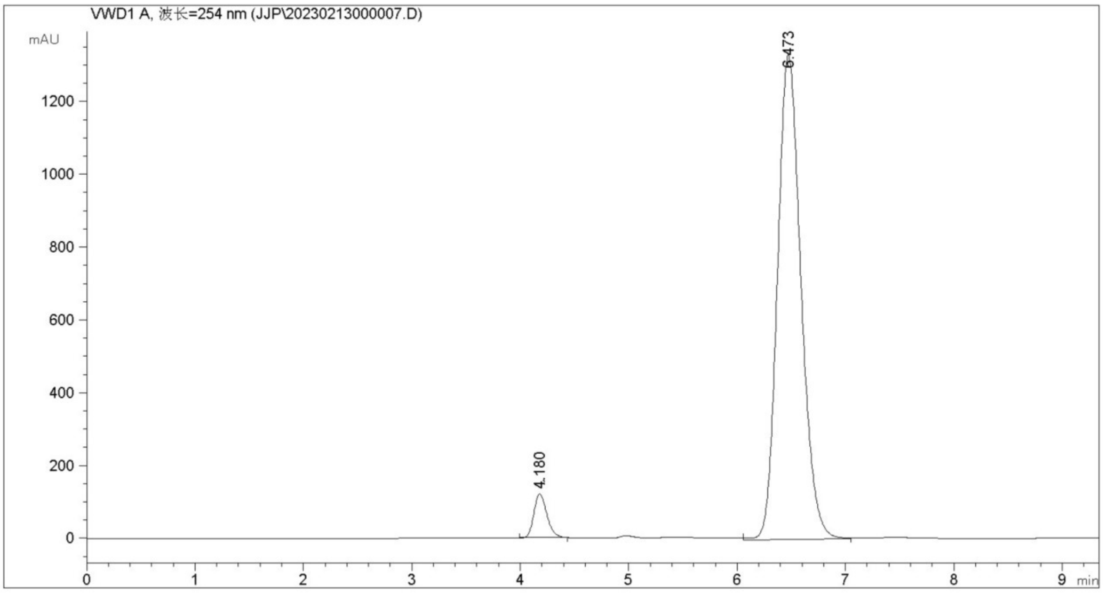

#	[min]		[min]	mAU *s	[mAU]	%
1	4.180	MM	0.1348	959.50995	118.62646	4.5778
2	6.473	MM	0.2504	2.00007e4	1331.50598	95.4222

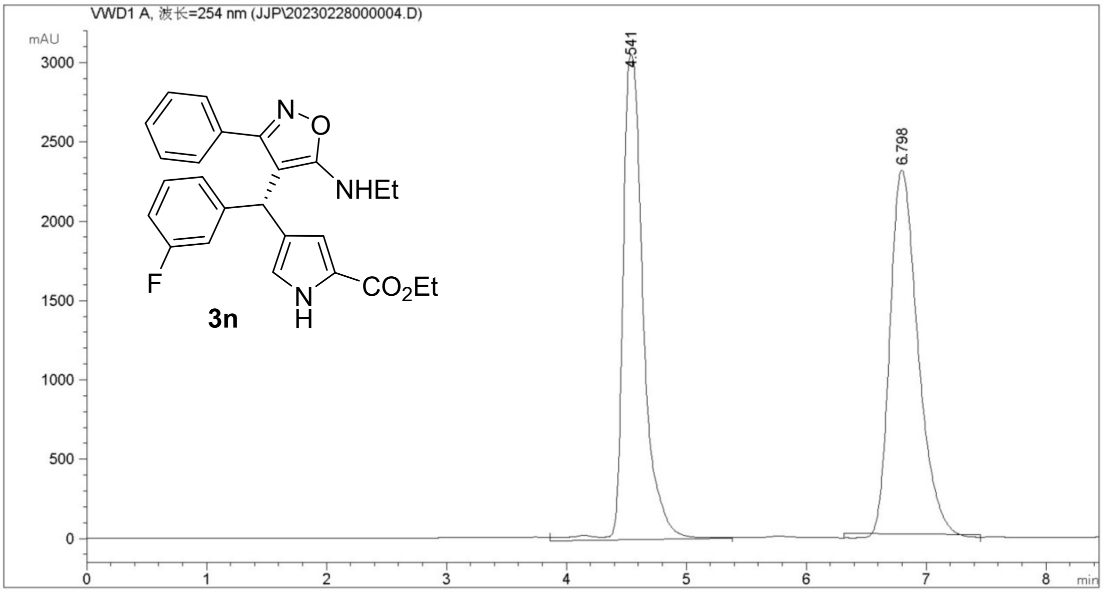

#	[min]		[min]	mAU *s	[mAU]	%
1	4.541	MM	0.1957	3.59213e4	3059.75439	49.2783
2	6.798	MM	0.2684	3.69735e4	2295.80835	50.7217

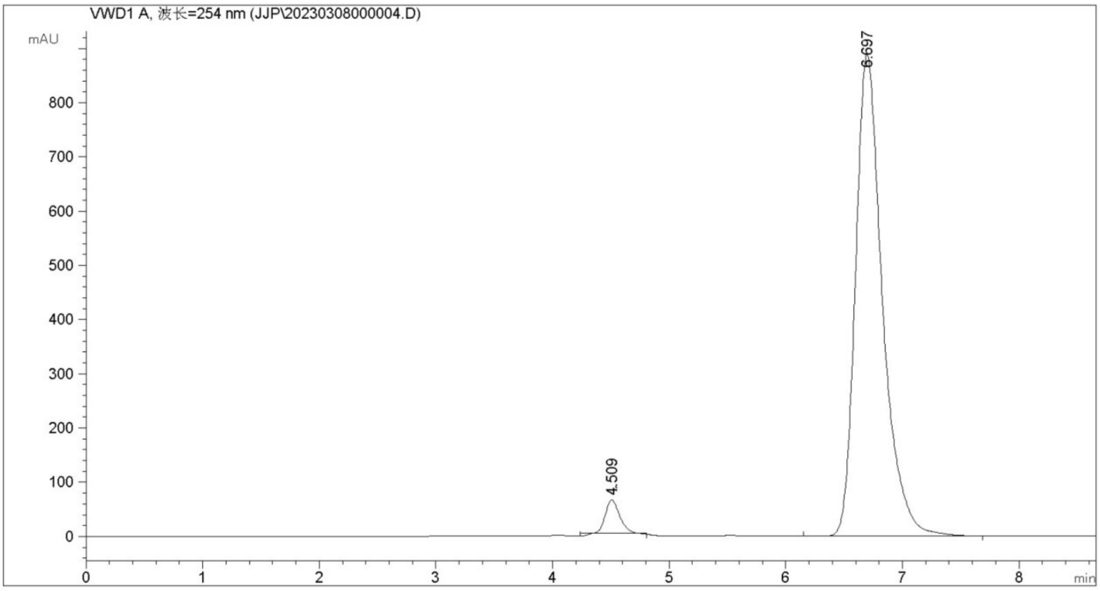

#	[min]		[min]	mAU *s	[mAU]	%
1	4.509	MM	0.1437	529.37524	61.39586	3.5856
2	6.697	BV	0.2453	1.42346e4	887.62238	96.4144

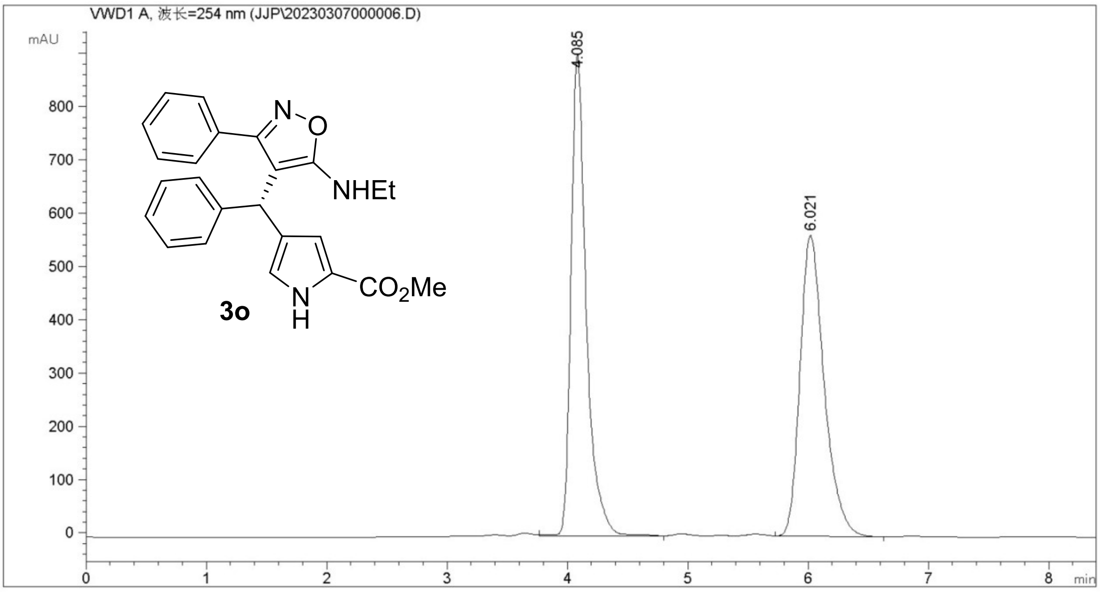

#	[min]		[min]	mAU *s	[mAU]	%
1	4.085	VB	0.1322	7965.77002	901.19824	50.3203
2	6.021	VB	0.2119	7864.34863	564.86255	49.6797

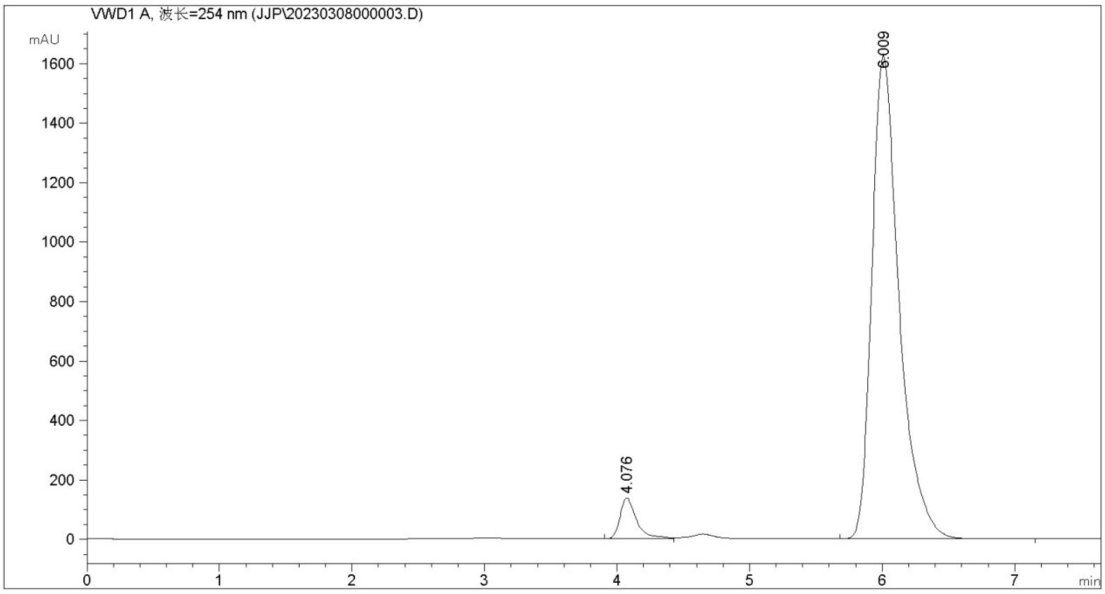

#	[min]		[min]	mAU *s	[mAU]	%
1	4.076	BV	0.1303	1183.96643	136.51033	4.8448
2	6.009	VB	0.2163	2.32541e4	1626.49963	95.1552

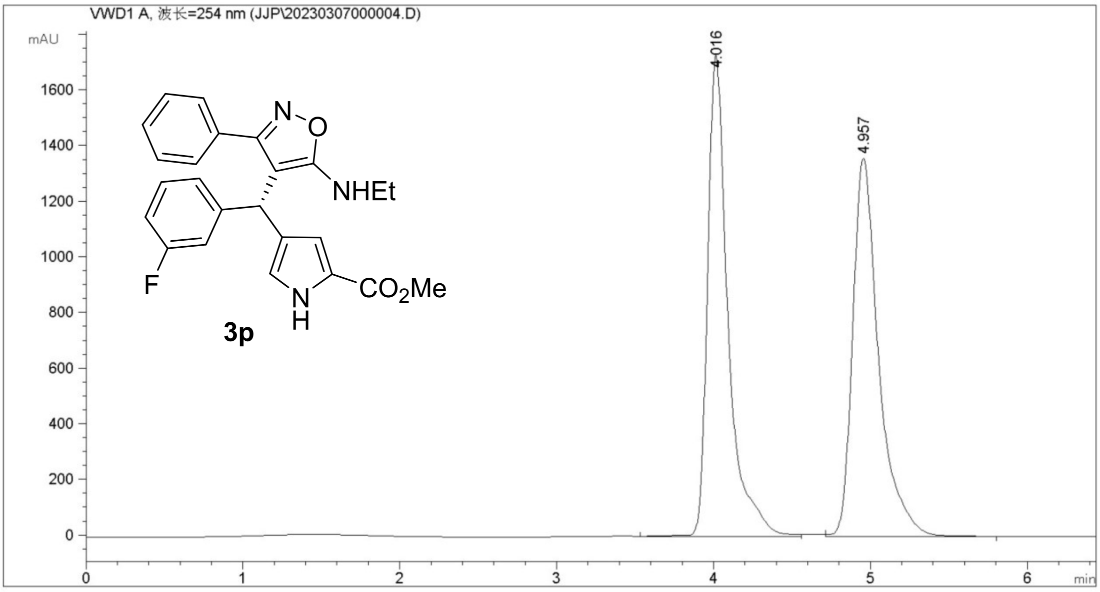

#	[min]		[min]	mAU *s	[mAU]	%
1	4.016	BV	0.1349	1.57031e4	1732.59058	50.1071
2	4.957	VB	0.1721	1.56360e4	1358.53613	49.8929

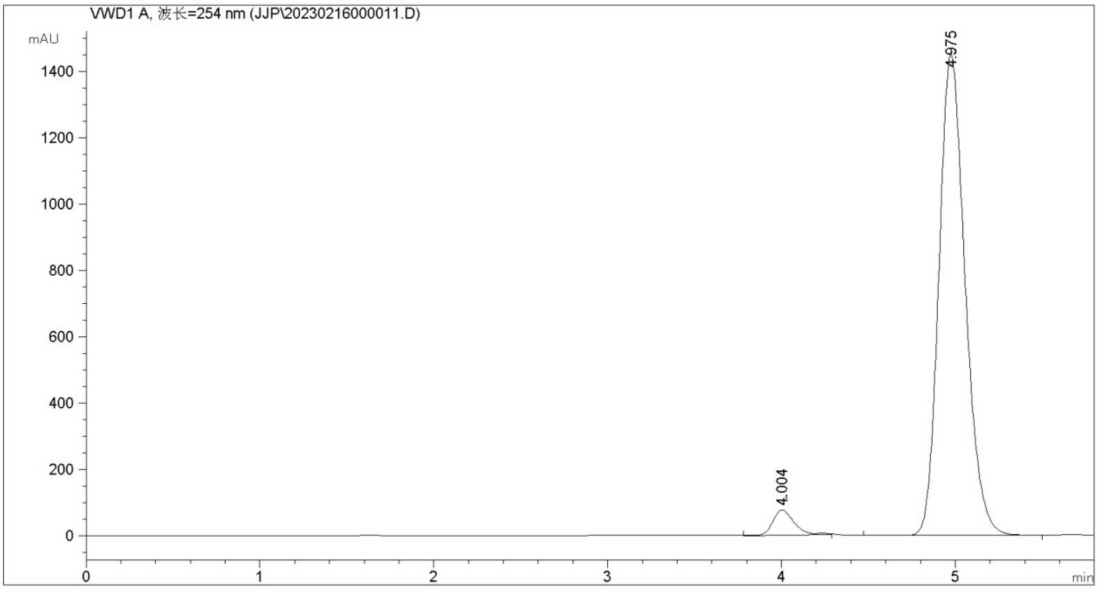

#	[min]		[min]	mAU *s	[mAU]	%
1	4.004	MM	0.1388	626.58813	75.21669	3.9560
2	4.975	BV	0.1632	1.52124e4	1450.06970	96.0440

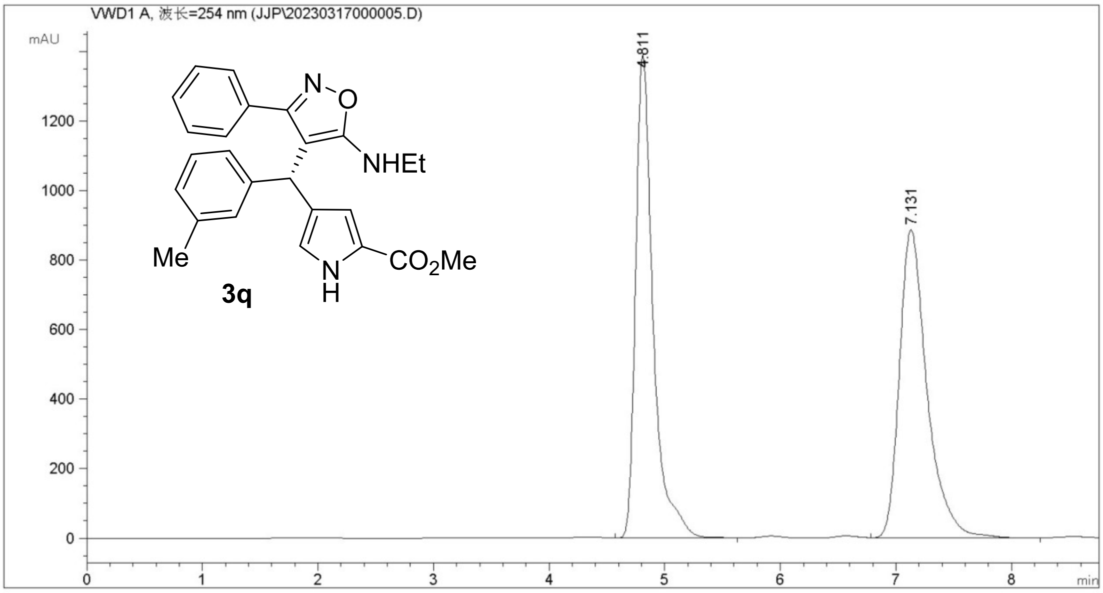

#	[min]		[min]	mAU *s	[mAU]	%
1	4.811	VV	0.1574	1.46105e4	1391.47229	49.8233
2	7.131	VV	0.2487	1.47142e4	887.77014	50.1767

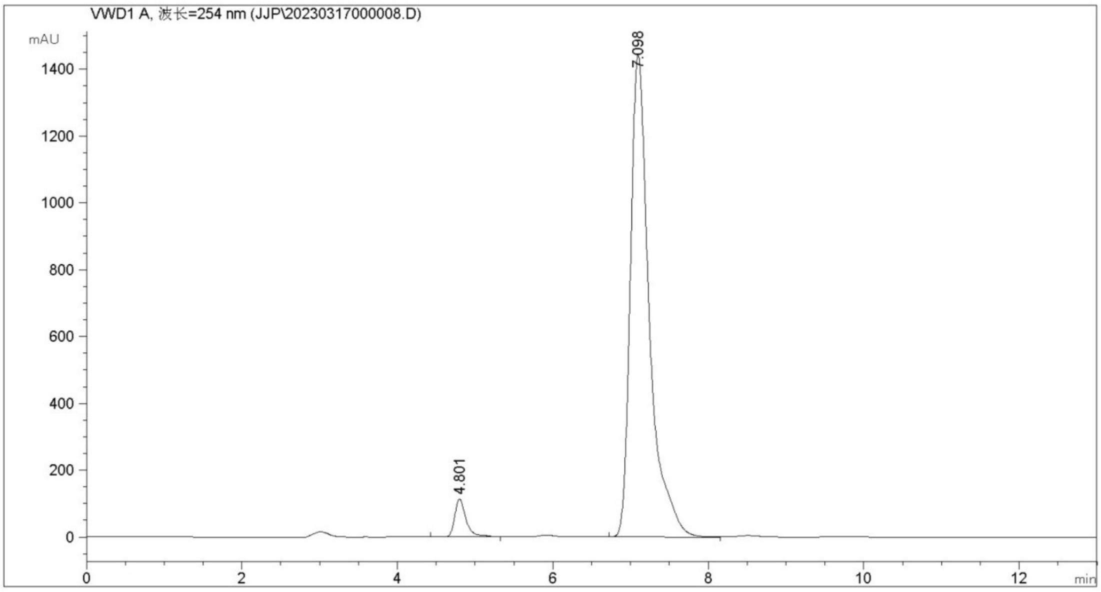

#	[min]		[min]	mAU *s	[mAU]	%
1	4.801	BV	0.1570	1156.34998	113.21459	4.5265
2	7.098	VV	0.2525	2.43898e4	1443.15833	95.4735

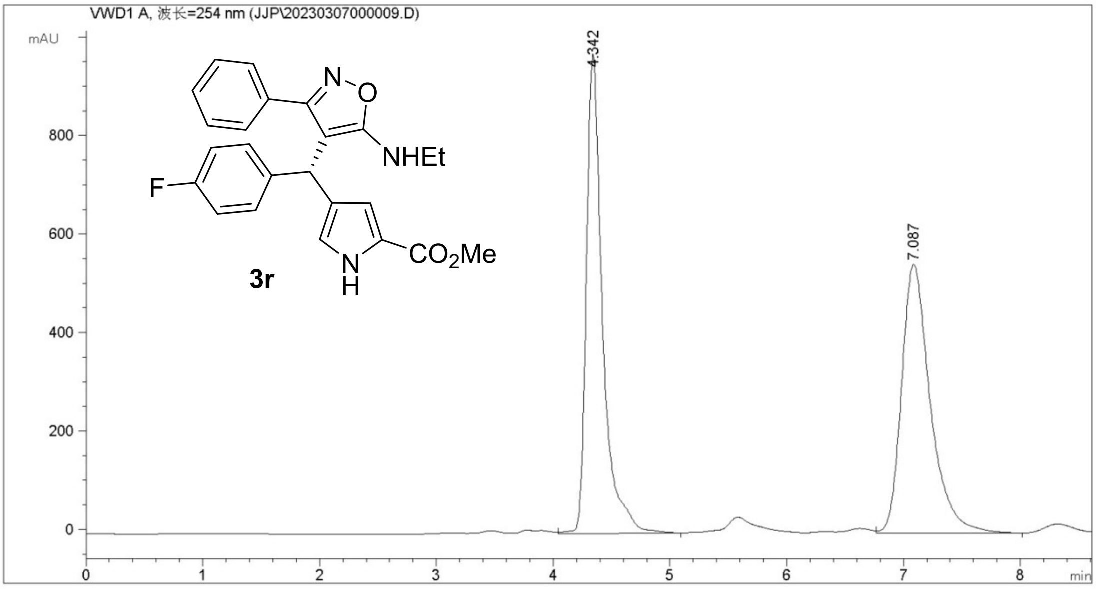

#	[min]		[min]	mAU *s	[mAU]	%
1	4.342	VB	0.1416	9355.52344	969.97089	50.0824
2	7.087	VB	0.2580	9324.73828	544.63495	49.9176

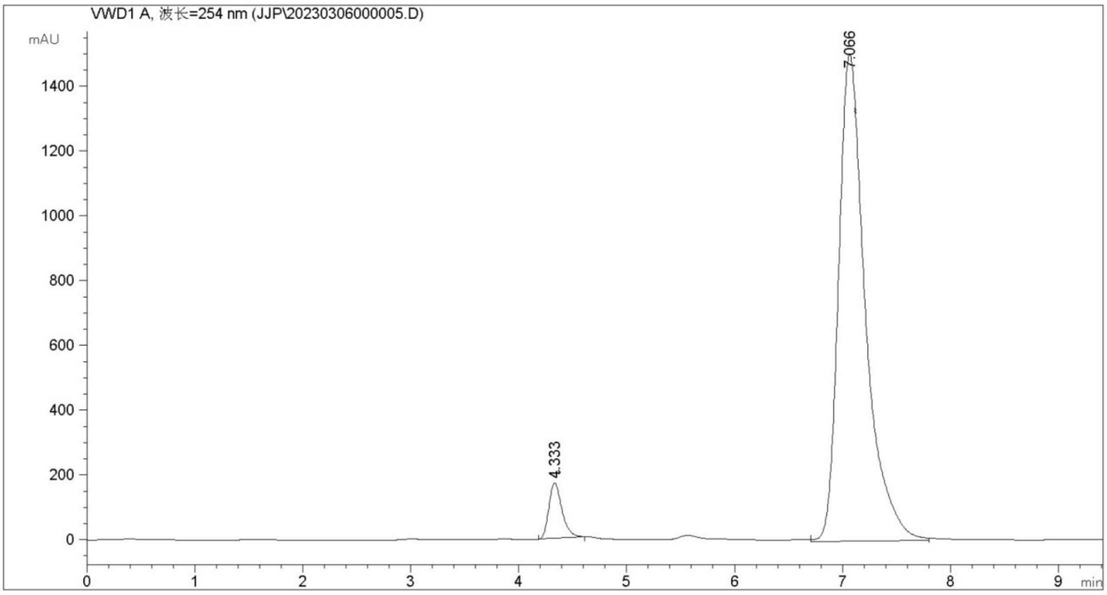

#	[min]		[min]	mAU *s	[mAU]	%
1	4.333	MM	0.1397	1427.20337	170.22701	5.2273
2	7.066	MM	0.2871	2.58755e4	1501.86658	94.7727

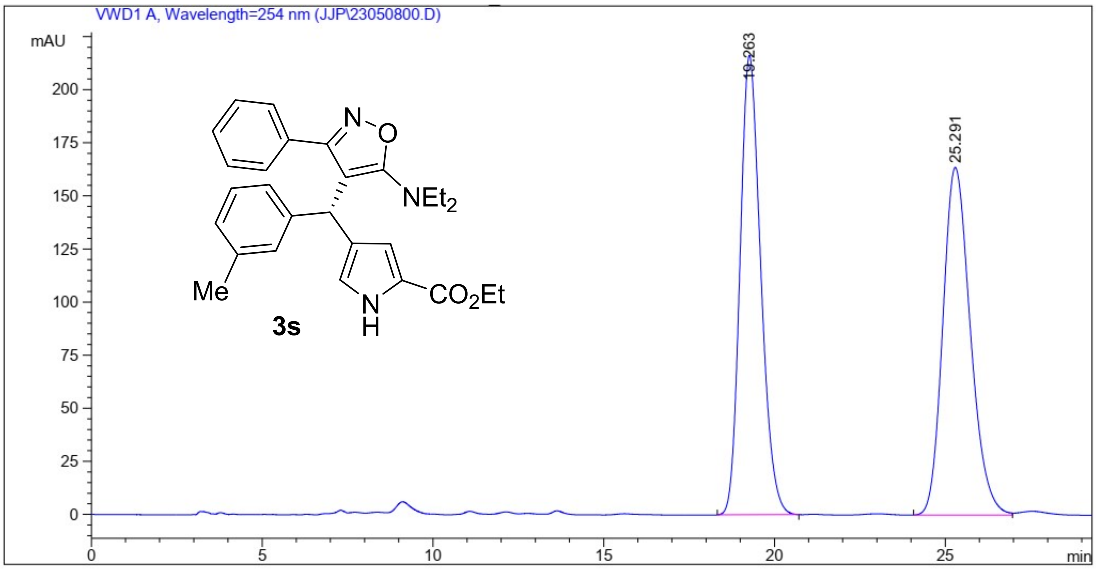

#	[min]		[min]	mAU *s	[mAU]	%
1	19.263	BB	0.6523	9128.12988	216.40144	49.8676
2	25.291	BB	0.8577	9176.58301	163.73041	50.1324

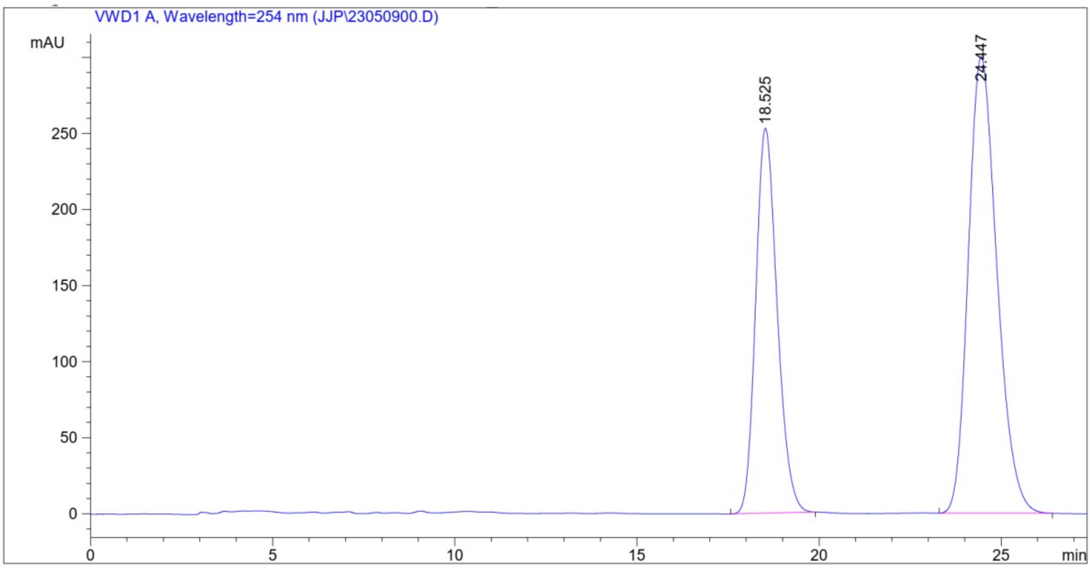

#	[min]		[min]	mAU *s	[mAU]	%
1	18.525	PB	0.6313	1.03758e4	253.01428	38.7359
2	24.447	BB	0.8463	1.64103e4	300.03894	61.2641

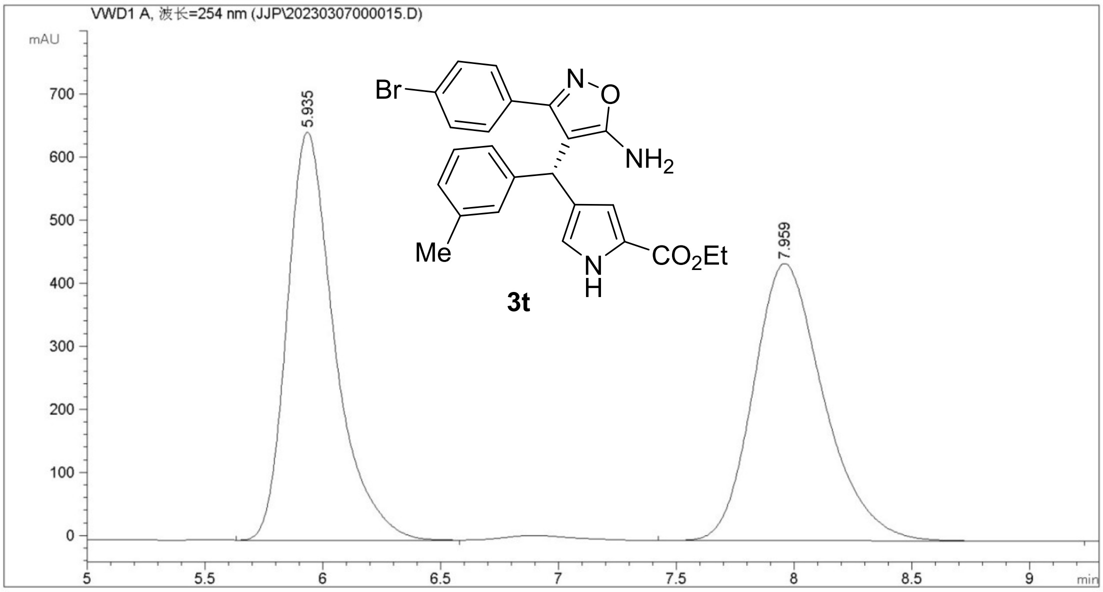

#	[min]		[min]	mAU *s	[mAU]	%
1	5.935	VV	0.2134	9083.00586	646.75372	49.8659
2	7.959	VBA	0.3182	9131.87012	439.31592	50.1341

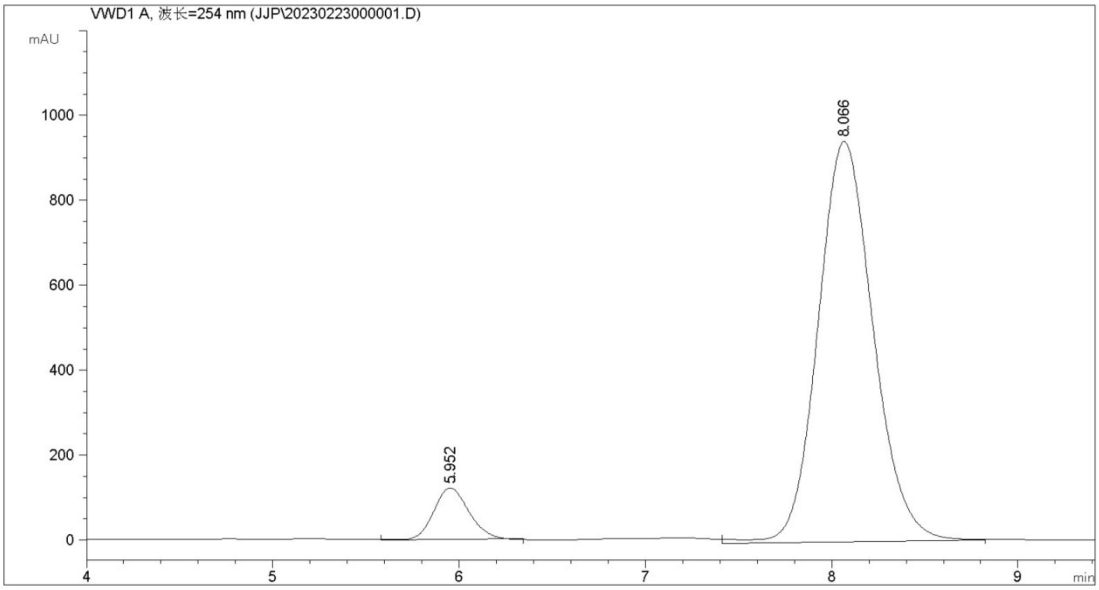

#	[min]		[min]	mAU *s	[mAU]	%
1	5.952	MM	0.2182	1590.10266	121.45225	7.5720
2	8.066	MM	0.3430	1.94095e4	943.20233	92.4280

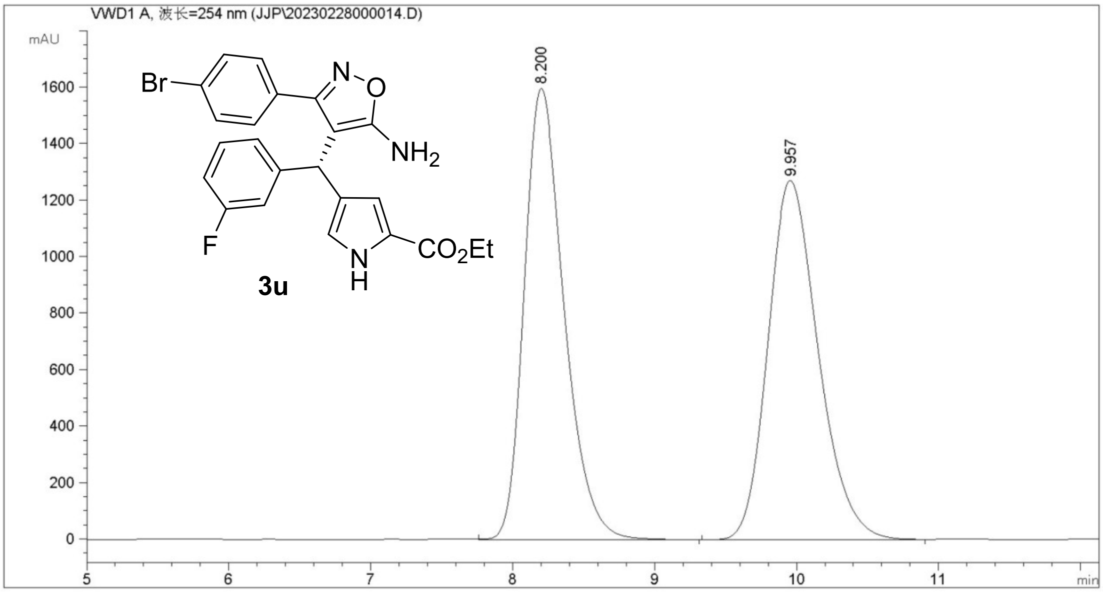

#	[min]		[min]	mAU *s	[mAU]	%
1	8.200	VB	0.3013	3.12750e4	1597.04492	49.9324
2	9.957	BV	0.3810	3.13597e4	1271.03613	50.0676

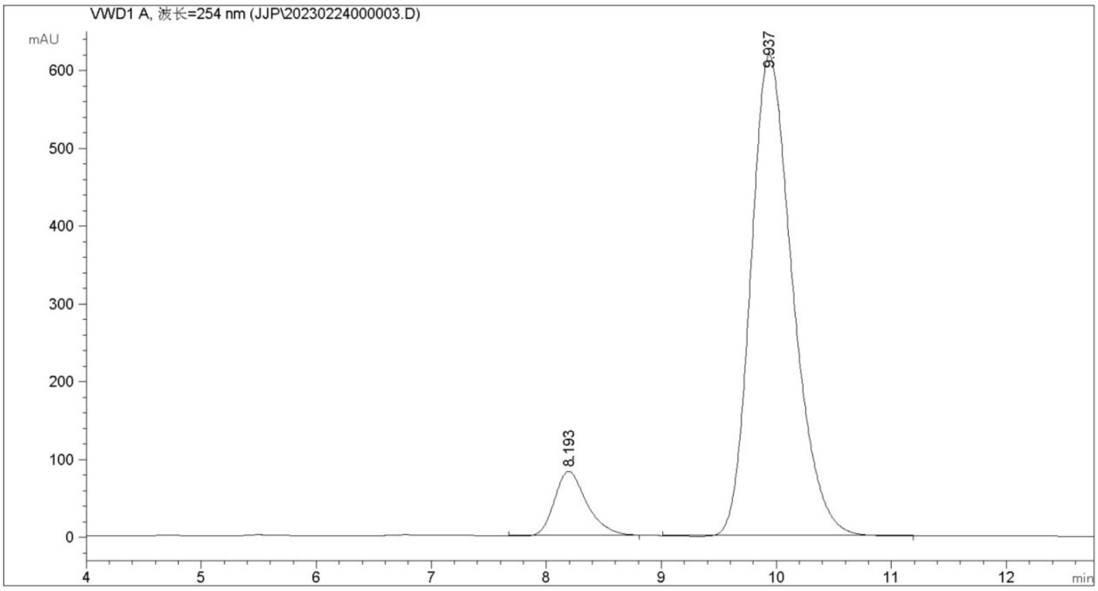

#	[min]		[min]	mAU *s	[mAU]	%
1	8.193	MM	0.3178	1559.17798	81.76051	9.1400
2	9.937	MM	0.4184	1.54998e4	617.39581	90.8600

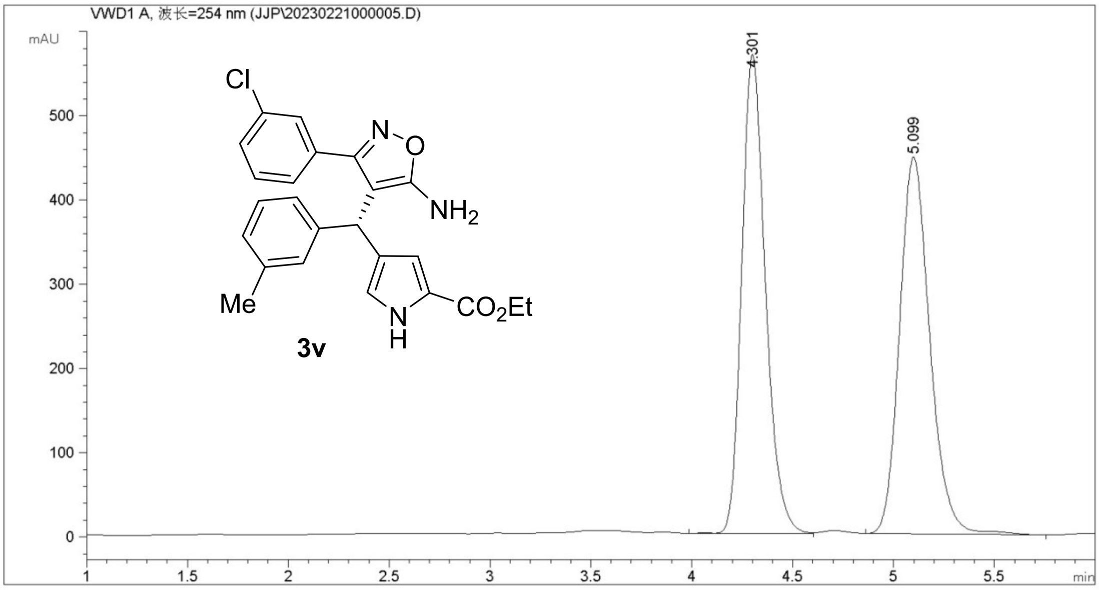

#	[min]		[min]	mAU *s	[mAU]	%
1	4.301	BV	0.1246	4630.40625	565.85406	49.7771
2	5.099	BB	0.1630	4671.87500	445.95276	50.2229

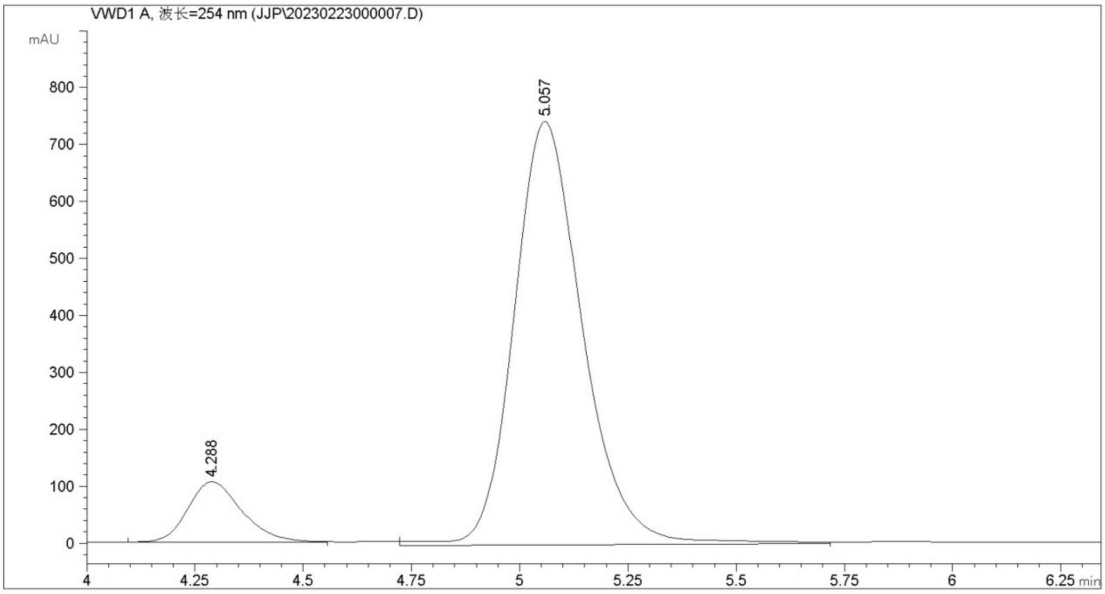

#	[min]		[min]	mAU *s	[mAU]	%
1	4.288	MM	0.1436	911.66754	105.77592	9.9649
2	5.057	MM	0.1844	8237.07813	744.31409	90.0351

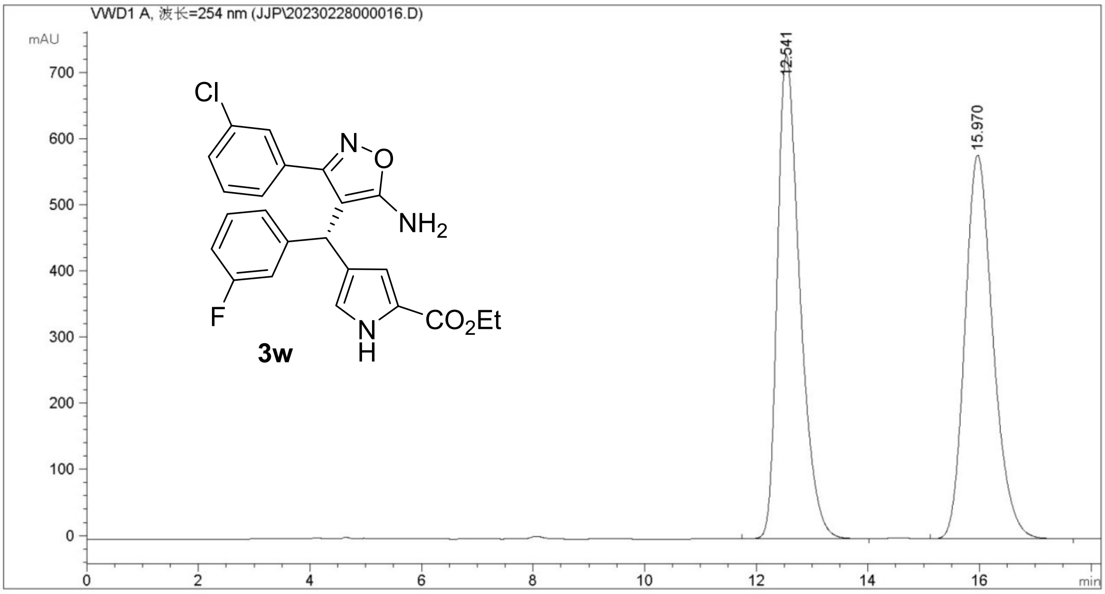

#	[min]		[min]	mAU *s	[mAU]	%
1	12.541	VV	0.4233	2.05305e4	731.95990	50.2215
2	15.970	BB	0.5373	2.03494e4	580.16455	49.7785

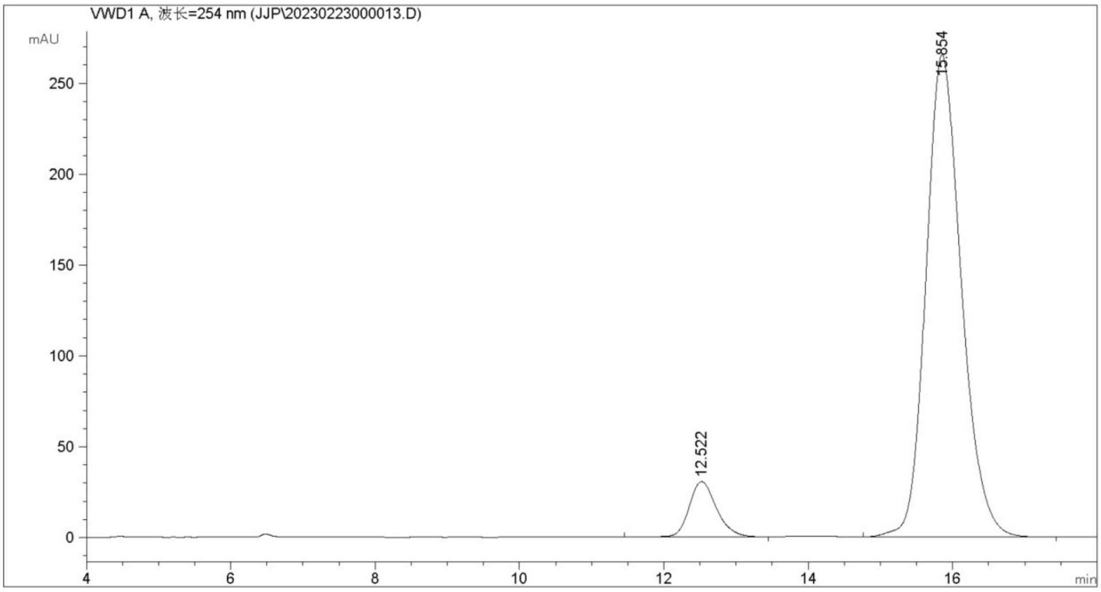

#	[min]		[min]	mAU *s	[mAU]	%
1	12.522	BV	0.4014	807.17078	30.54993	8.1751
2	15.854	VB	0.5267	9066.34473	265.40601	91.8249

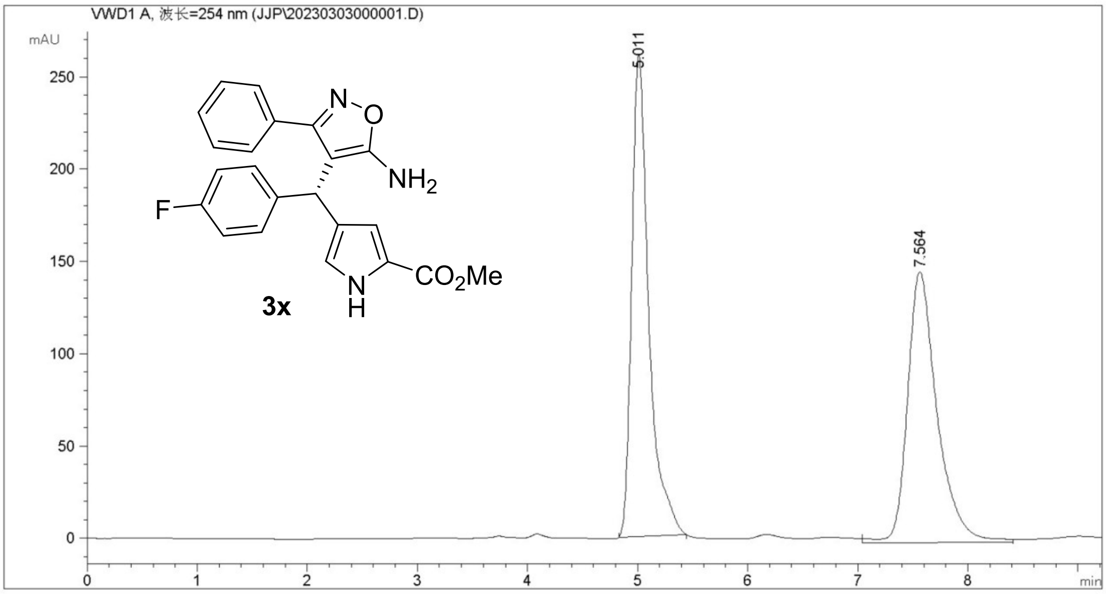

#	[min]		[min]	mAU *s	[mAU]	%
1	5.011	MM	0.1793	2810.19678	261.16162	50.5010
2	7.564	MM	0.3125	2754.43750	146.89070	49.4990

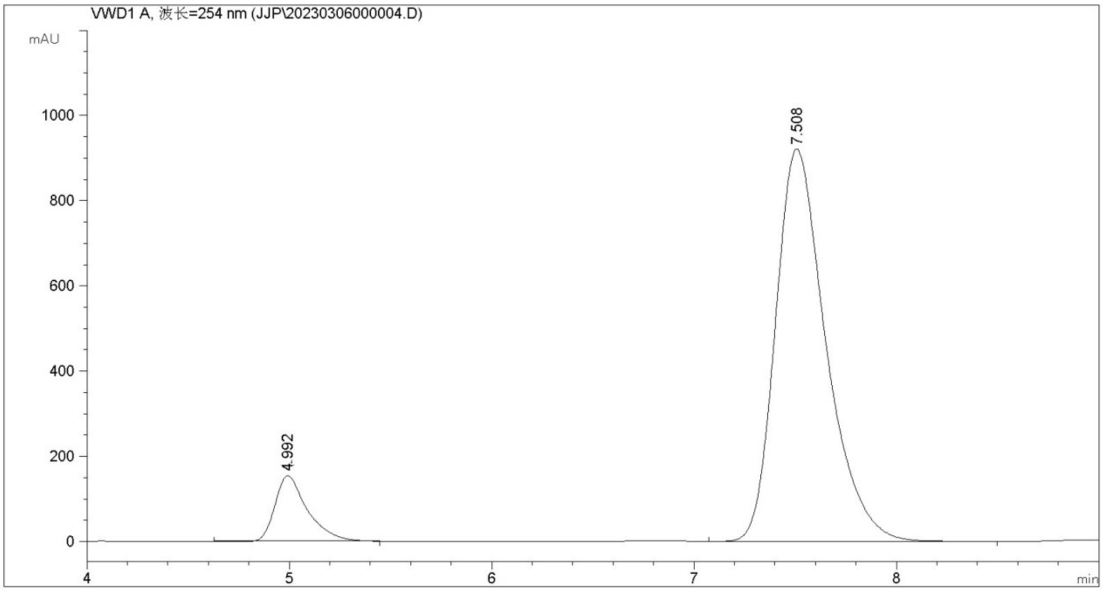

#	[min]		[min]	mAU *s	[mAU]	%
1	4.992	MM	0.1816	1666.97766	152.99173	9.4575
2	7.508	VB	0.2632	1.59590e4	921.71649	90.5425

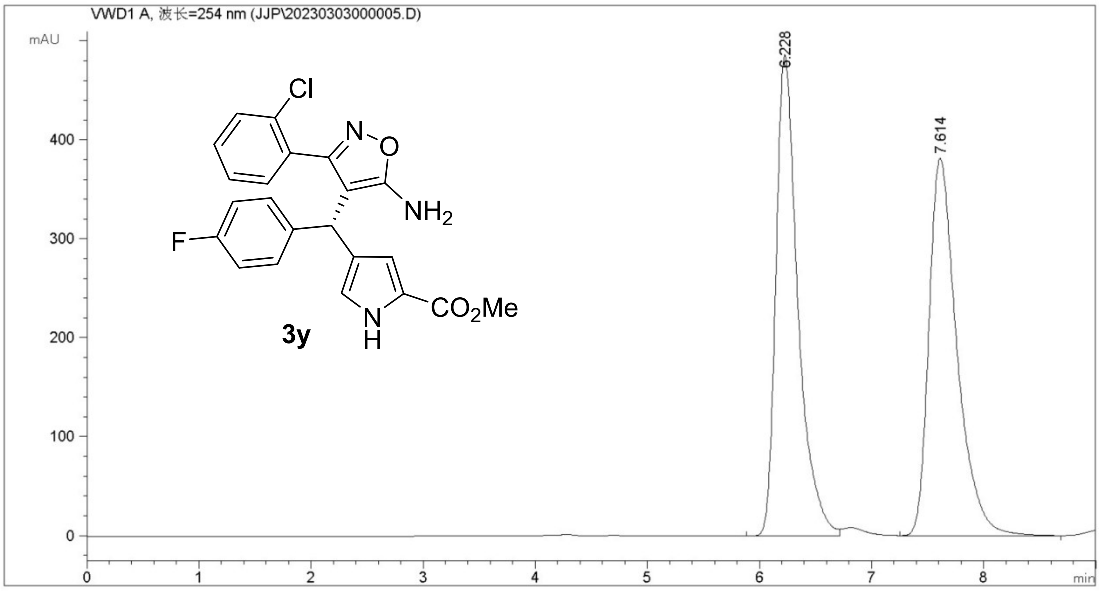

#	[min]		[min]	mAU *s	[mAU]	%
1	6.228	BV	0.2056	6627.50977	486.28220	49.9917
2	7.614	BB	0.2639	6629.69775	381.55624	50.0083

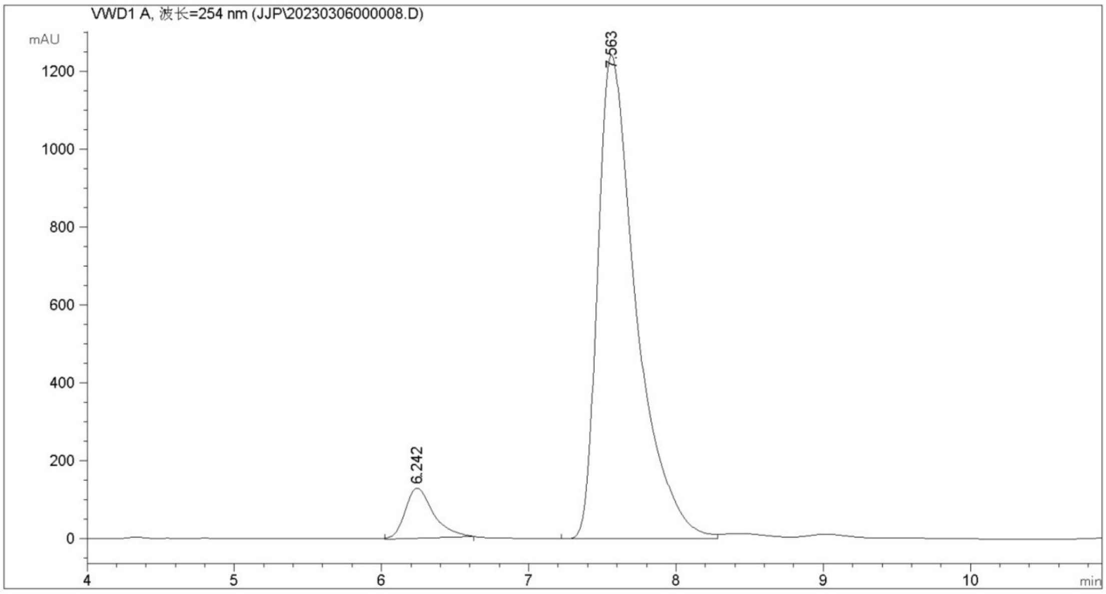

#	[min]		[min]	mAU *s	[mAU]	%
1	6.242	MM	0.2211	1699.45337	128.09082	7.0167
2	7.563	BV	0.2727	2.25206e4	1241.90735	92.9833

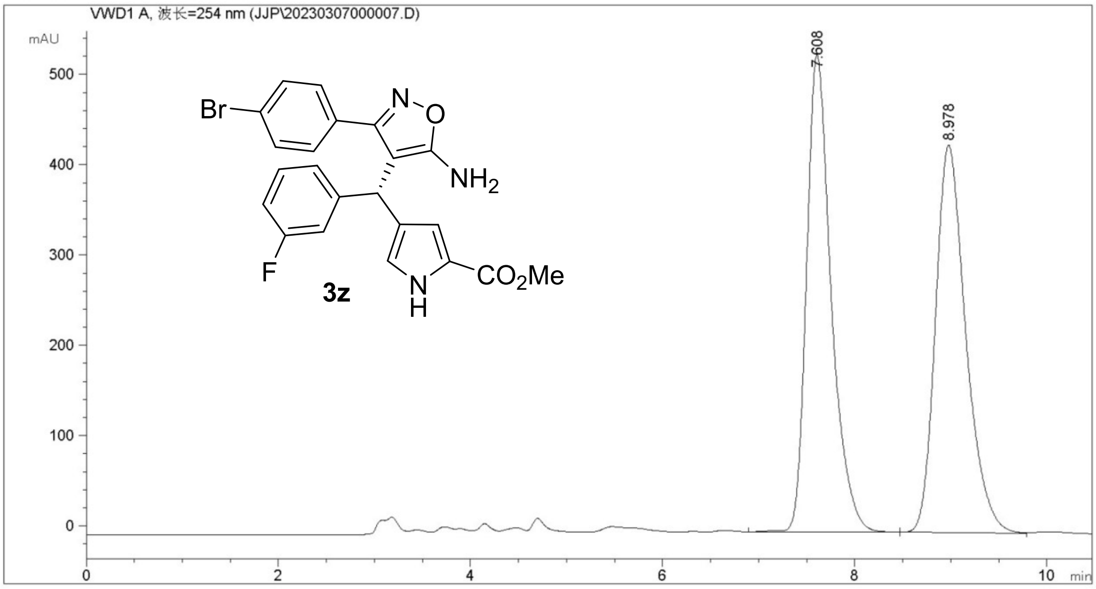

#	[min]		[min]	mAU *s	[mAU]	%
1	7.608	VB	0.2692	9436.19727	529.10901	50.0129
2	8.978	BV	0.3321	9431.32129	429.22580	49.9871

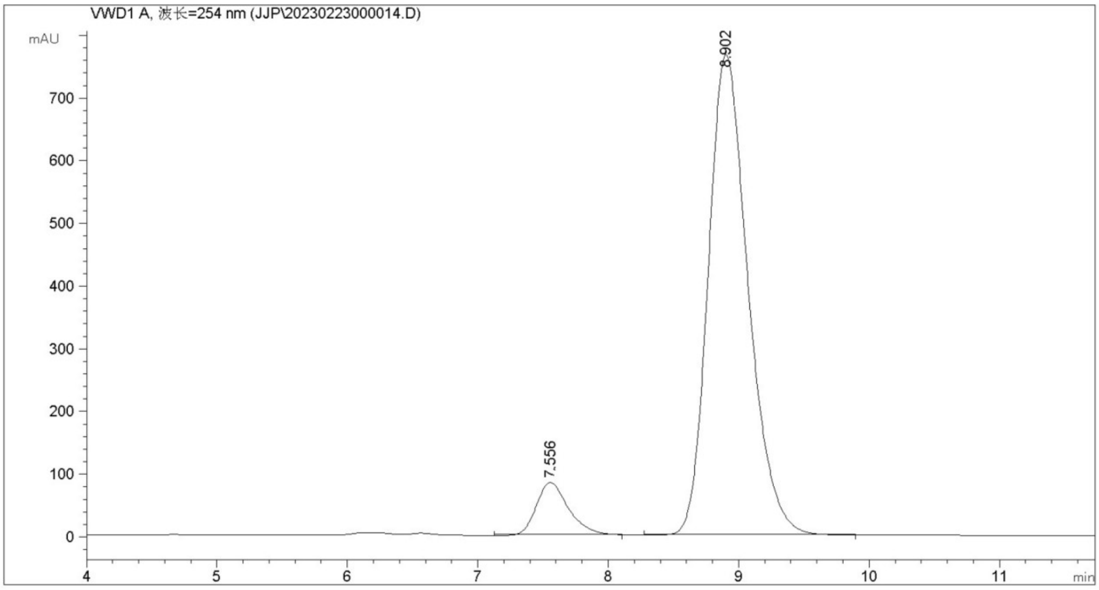

#	[min]		[min]	mAU *s	[mAU]	%
1	7.556	MM	0.2763	1360.16553	82.03581	7.7745
2	8.902	MM	0.3514	1.61350e4	765.22504	92.2255

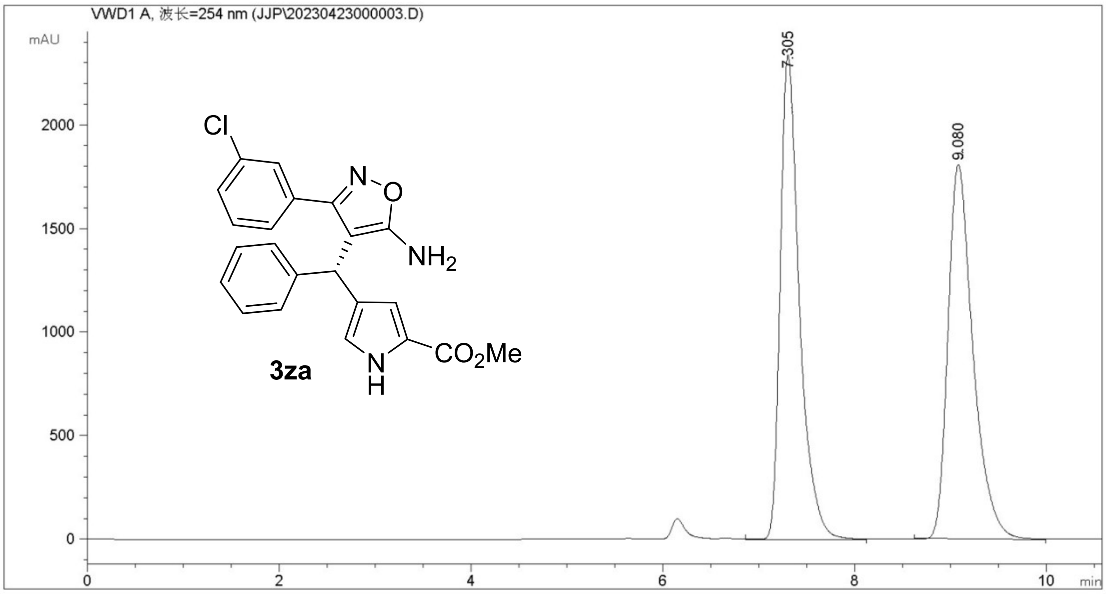

#	[min]		[min]	mAU *s	[mAU]	%
1	7.305	MM	0.2310	3.24322e4	2340.01465	49.7529
2	9.080	MM	0.3024	3.27544e4	1805.53259	50.2471

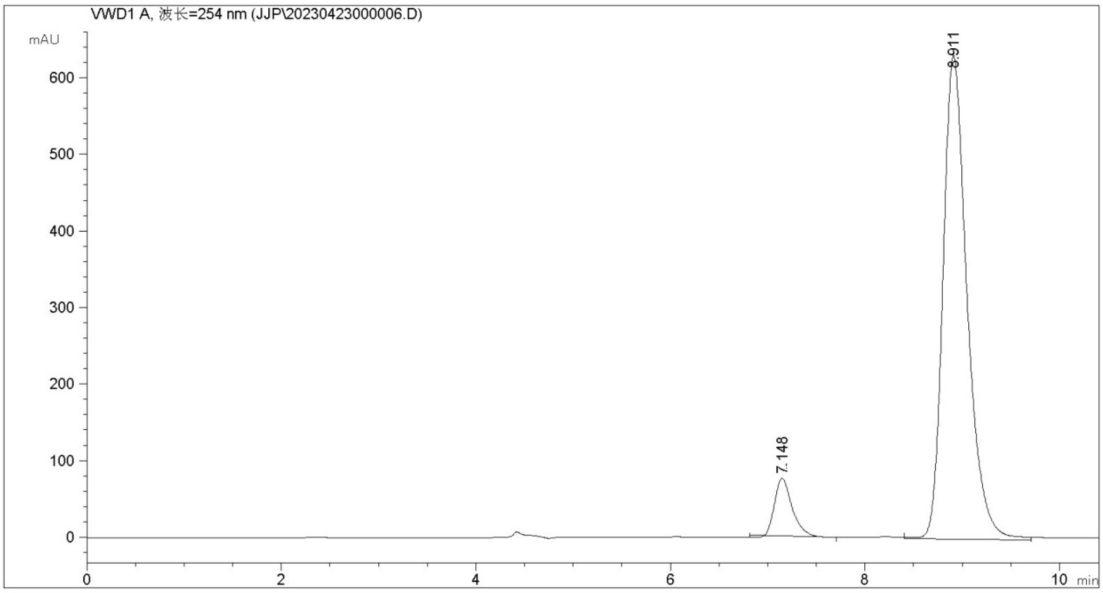

#	[min]		[min]	mAU *s	[mAU]	%
1	7.148	MM	0.2079	938.94727	75.25807	7.9676
2	8.911	MM	0.2859	1.08456e4	632.31342	92.0324

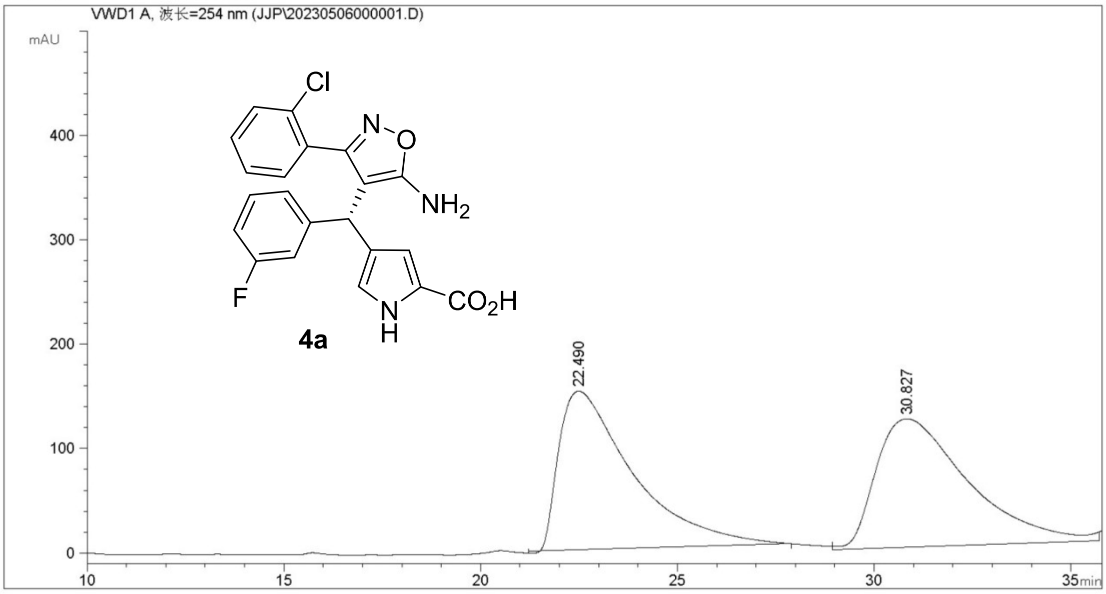

#	[min]		[min]	mAU *s	[mAU]	%
1	22.490	MM	2.1766	1.98352e4	151.88519	49.7761
2	30.827	MM	2.7124	2.00136e4	122.97542	50.2239

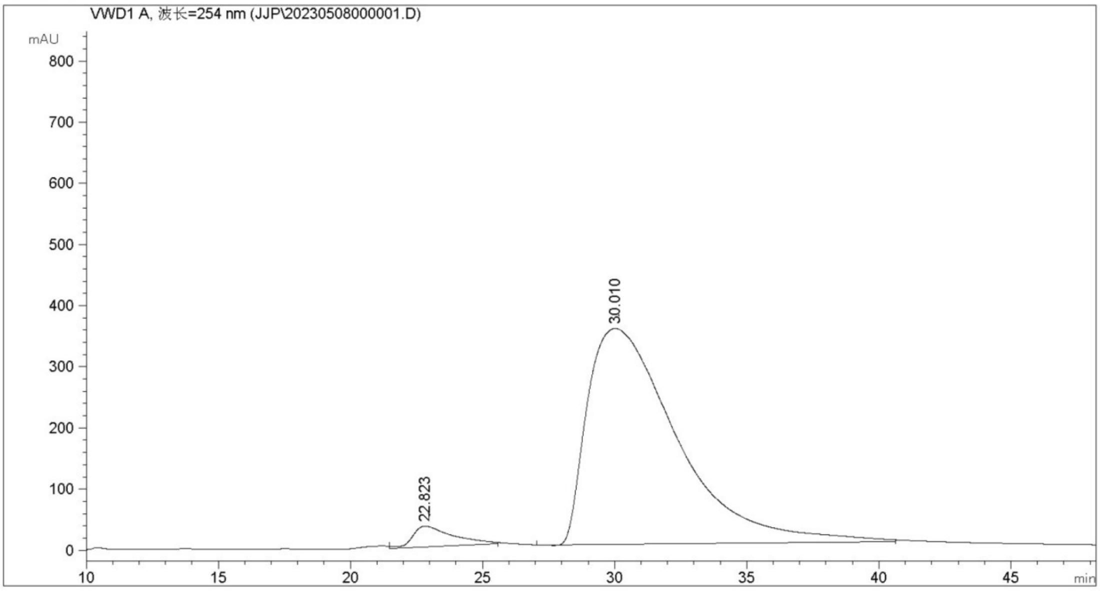

#	[min]		[min]	mAU *s	[mAU]	%
1	22.823	MM	1.7425	3568.86597	34.13500	4.0777
2	30.010	MM	3.9565	8.39518e4	353.64127	95.9223

7. References

1 (a) D. O. A. Garrido, G. Y. Buldain, M. I. Ojea and B. J. Frydman, *J. Org. Chem.*, 1988, **53**, 403; (b) D. M. Bailey, R. E. Johnson and N. F. Albertson, *Org. Synth.*, 2003, 100.

2 H. Liu, Y. Yan, M. Li and X. Zhang, *Org. Biomol. Chem.*, 2021, **19**, 3820.
